# Speciation atlas of polyoxometalates in aqueous solutions

**DOI:** 10.1126/sciadv.adi0814

**Published:** 2023-06-21

**Authors:** Nadiia I. Gumerova, Annette Rompel

**Affiliations:** Universität Wien, Fakultät für Chemie, Institut für Biophysikalische Chemie, Josef-Holaubek-Platz 2, 1090 Wien, Austria.

## Abstract

Speciation is the key parameter in solution chemistry that describes the composition, concentration, and oxidation state of each chemical form of an element present in a sample. The speciation study of complex polyatomic ions has remained challenging because of the large number of factors affecting stability and the limited number of direct methods. To address these challenges, we developed the speciation atlas of 10 polyoxometalates commonly used in catalytic and biological applications in aqueous solutions, where the speciation atlas provides both a species distribution database and a predictive model for other polyoxometalates to be used. Compiled for six different polyoxometalate archetypes with three types of addenda ions based on 1309 nuclear magnetic resonance spectra under 54 different conditions, the atlas has revealed a previously unknown behavior of polyoxometalates that may account for their potency as biological agents and catalysts. The atlas is intended to promote the interdisciplinary use of metal oxides in various scientific fields.

## Teaser

The speciation atlas is the large-scale study of the polyoxometalates’ behavior in solutions.

## INTRODUCTION

Polyoxometalates (POMs) (*1*, *2*), being polyatomic ions, are predominantly anionic metal oxide inorganic compounds that exhibit diverse structures and are soluble in liquid phases and, hence, have many applications in solutions, especially in homogeneous catalysis (*3*–*5*) and as biologically active agents for the benefit of human health (*6*–*8*). Normally, POMs are thoroughly characterized in the solid state, and this structural information often forms the basis on which knowledge of the POMs’ behavior in solution was developed. After dissolution in aqueous media, POM anions, similar to many other compounds, can be protonated and hydrolyzed (*2*) and, sometimes, even undergo redox processes (*9*), all of which affect the presence of the active species. There is a great need for a systematic study of the speciation of POMs encountered in solution (*10*). Here, we expand our knowledge of the speciation of POMs in aqueous solutions by analyzing 10 commonly used POM representatives using a quantitative nuclear magnetic resonance (NMR) spectroscopy–based approach, the most readily available method for understanding processes in solution. Seven phosphorus-containing polyoxotungstates (POTs) [α-PW_12_O_40_]^3−^ (Keggin-type, **PW**_**12**_; fig. S1A) (*11*), [α-P_2_W_18_O_62_]^6−^ (Wells-Dawson–type, **P**_**2**_**W**_**18**_; fig. S1B) (*12*), [α/β-P_2_W_18_O_62_]^6−^ (Wells-Dawson–type, **α**/**β-P**_**2**_**W**_**18**_; fig. S1) (*13*), [NaP_5_W_30_O_110_]^14−^ (Preyssler-type, **P**_**5**_**W**_**30**_; fig. S1C) (*14*), [{α-P^V^W^VI^_11_O_39_Zr^IV^(μ-OH)(H_2_O)}_2_]^8−^ [Keggin-based sandwich, **(ZrPW**_**11**_**)**_**2**_; fig. S1D] (*15*), two polyoxomolybdates (POMos) [α-PMo_12_O_40_]^3−^ (Keggin-type, **PMo**_**12**_; fig. S1A) (*16*) and [α-P_2_Mo_18_O_62_]^6−^ (Wells-Dawson–type, **P**_**2**_**Mo**_**18**_; fig. S1B) (*17*), and two POTs the Anderson-type POT [TeW_6_O_24_]^6−^ (**TeW**_**6**_; fig. S1E) (*18*) and silicotungstic acid [SiW_12_O_40_]^4−^ (**SiW**_**12**_; fig. S1A) (*19*), and the prominent polyoxovanadate representative, decavanadate, [V_10_O_28_]^6−^ (**V**_**10**_; fig. S1F) (*20*) were selected for this study, considering their common use in both catalysis and biology (table S3) and the availability of active NMR nuclei in the POM anion. The atlas is the first comprehensive tool that identifies the species(s) present in buffer solutions, thus providing a framework for understanding POM activity in aqueous solutions. For the POM types not discussed here, as well as for all other metal oxides that transform in aqueous solution, a guide is presented on how to proceed when identifying species that occur in aqueous solution.

## RESULTS

### Speciation atlas of 10 of the most commonly used POMs in aqueous solution

This atlas describes the behavior of 10 of the most commonly used discrete metal oxides in catalysis (*21*, *22*) and biological studies (*6*, *7*, *23*–*26*) (Fig. 1A) under 54 different buffered aqueous conditions (Table 1), resulting in speciation maps based on 1134 ^31^P NMR, 162 ^51^V NMR, and 13 ^183^W NMR spectra (figs. S11 to S148 and tables S5 to S28). The Anderson-type POT [TeW_6_O_24_]^6−^ (**TeW**_**6**_; fig. S149), which is a successful additive for protein crystallization (*26*), and the catalytically active (*3*) [applied in, e.g., esterification (*27*) and acetalization (*28*)], silicotungstic acid [SiW_12_O_40_]^4−^ (**SiW**_**12**_; fig. S30) were only studied for selected conditions because of the long acquisition times encountered in ^183^W NMR.

**Fig. 1.**
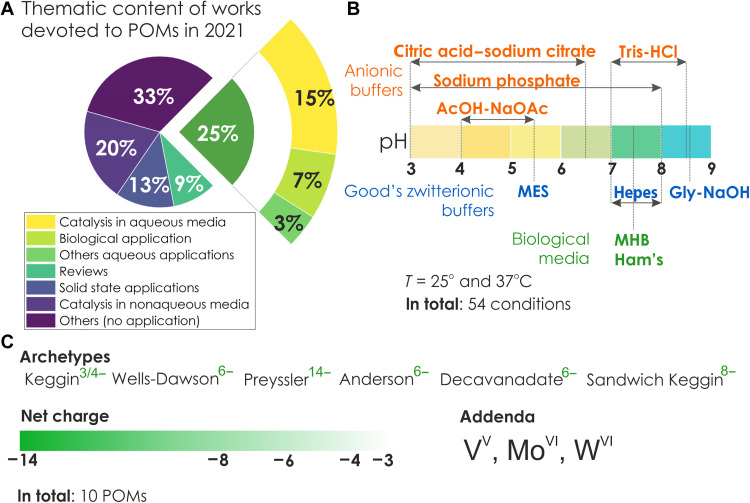
Speciation atlas parameters. (**A**) Distribution of topics in papers about POMs published in 2021. For the Scopus search term “polyoxometalate,” 655 publications were specified and hand-checked to first classify them into five categories: (i) describing any application in aqueous solutions (25%; grass green); (ii) describing the use of POMs in nonaqueous catalysis (20%; violet); (iii) describing the use of POM in the solid state (13%; light violet); (iv) reviews and book chapters (9%; light green); (v) those that do not describe applications (33%; purple). The articles describing the use of POMs in aquatic environments were sorted by their use in catalysis (15%), biology (7%), and other fields (3%). (**B**) The conditions (kind of buffer, pH value, and temperature) chosen for compiling the speciation atlas. Phosphate-buffered saline (PBS) is covered under the general name “phosphate buffer.” (**C**) This study examined the main characteristics (archetype, net charge, and addenda) of the POMs. Archetypes covered in this resource are listed in order of frequency of occurrence in the literature.

**Table 1.** Buffers and media used in the study. List of selected buffers (concentration of 0.1 M) for stability studies of POMs.

Buffer type	Ascending numbering	Buffer	p*K*_a_ at 25°C (*31*, *57*)	pH range	pH values applied for speciation study	Number of pH values at which this buffer was used
Anionic	1	Acetic acid–sodium acetate (*57*)	4.76	3.6–5.6	4, 5, and 5.5	3
	2	Citric acid–sodium citrate (*57*)	3.13, 4.76, and 6.40	3–7.2	3, 4, 5, 6, and 6.5	5
	3	Sodium phosphate (*57*)	2.15, 6.86, and 12.32	2–8	3, 4, 5, 6, 7, and 8	6
	4	Phosphate-buffered saline (PBS) (*57*)	6.86	5.8–7.8	7.4	1
	5	Tris-HCl (*57*) (see structure in fig. S2)	8.06	7.1–8.9	7.5, 8, and 8.5	3
Zwitterionic	6	Glycine-NaOH (*31*)	2.35 and 9.78	8.2–10.6	8.6	3 (this buffer was applied in three concentrations—0.1, 0.2, and 0.5 M)
	7	Hepes (*31*) (see structure in fig. S2)	3 and 7.5	6.8–8.2	7 and 8	2
	8	MES (*31*) (see structure in fig. S2)	6.15	5.5–6.7	6	1
	9	MHB (*32*)			7.4	1
	10	F-12 Ham (*33*)			7.4	1
	11	Double-distilled H_2_O			-	1
Total number of conditions:						Σ 27

For the compilation of the atlas, we applied buffer types under conditions previously used in catalytic and biological applications (table S3). Most commonly, POM-based catalysis has been performed either only in water or in buffer solutions (e.g., acetate, phosphate, and citrate) (*29*) in the pH range from 5 to 8, but some catalysis also runs in a strongly acidic region (e.g., water oxidation electrocatalysis even at pH < 1; table S3) (*5*). For biological experiments, note that the pH varies between 4.5 and 8.0 (*30*) in different cell organelles, so this is the range where the speciation needs to be studied. The pH range from 3 to 9 is often covered by anionic buffers {acetic acid–sodium acetate, citric acid–sodium citrate, sodium phosphate [including phosphate-buffered saline (PBS)], and tris-HCl} and by the so-called organic Good’s buffer (*31*) solutions: Hepes (*31*), MES (*31*) and glycine-NaOH (*31*) (Fig. 1 and see fig. S2 for structural formulas of buffer components). Biological studies often require media of complex composition that support the growth and well-being of the living organisms investigated. Therefore, we studied two media: Mueller-Hinton broth (MHB) (*32*) (a liquid medium for antibiotic susceptibility studies containing dehydrated infusion from beef, casein hydrolysate, and starch) and nutrient mixture F-12 Ham (*33*) (a serum-free medium for mammalian cell growth containing sodium pyruvate, phenol red, and l-glutamine but no NaHCO_3_ and Hepes; for more details, www.sigmaaldrich.com/AT/en/technical-documents/technical-article/cell-culture-and-cell-culture-analysis/mammalian-cell-culture/f-12-ham). The speciation data were recorded at overlapping pH values of different types of buffers (Fig. 1B) to understand not only the effect of the solution pH but also the effect of the buffer/medium components, which are normally considered relatively benign but can play a key role in the stability, speciation, and efficiency of POM applications. To provide buffering capacity, the concentration of the buffering components must be at least an order of magnitude higher than the concentration of the solute (*34*). Considering NMR signal quality and POM concentrations successfully used in previous applications (see references to literature in table S3), a 10 mM POM concentration was selected, and the buffer concentration was adjusted to 0.1 M accordingly. For **TeW**_**6**_ and **SiW**_**12**_, which were examined only by ^183^W NMR spectroscopy, the concentrations of POM and buffer solution were doubled because of the low abundance (~14%) of the ^183^W isotope.

POM investigations are usually performed at room temperature, so, initially, all speciation measurements were done under standard conditions [1 atm (101,325 Pa) and 25°C (298 K)]. To investigate the influence of temperature on POMs’ speciation, we subsequently incubated all solutions measured at room temperature at 37°C for 24 hours and measured them again. This takes the physiological temperature into account and gives the first insights into the speciation of POMs at temperatures above room temperature.

Although pH and temperature are considered the main factors affecting speciation, other parameters such as the incubation time of the solution before its use, ionic strength (*34*) of the solution, the presence of chelating or reducing (*35*) components as part of the buffer content, and the nature of counter cation (*36*) also need to be carefully thought about. The influence of these factors is considered individually below, but it is also important to examine how they act together. In the following sections, we describe the speciation of three of the 10 POMs to give representative examples of the information covered by the atlas. These three flagship POMs, which are the most accessible and widely used (table S3), are the Keggin-type POT **PW**_**12**_ as a low-charge (−3) anion that is hydrolyzed under all the conditions studied here (tables S5 to S7 and figs. S11 to S29); the Wells-Dawson–type POT **P**_**2**_**W**_**18**_ with a net charge of −6, which only degrades at pH ˃ 5 (tables S8, S10, and S11 and figs. S31, S32, S34 to S42, and S53 to S59); and the Preyssler-type POT **P**_**5**_**W**_**30**_ as a highly negatively charged anion of −14, which is very stable over the entire pH range tested (tables S14 to S16 and figs. S67 to S79). For a detailed analysis of the other seven studied POMs, please see supplementary tables and figures—**PMo**_**12**_ (tables S17 to S19 and figs. S80 to S97), ***α/β*-P**_**2**_**W**_**18**_ (tables S9, S12, and S13 and figs. S31, S34, S44 to S52, and S60 to S66), **P**_**2**_**Mo**_**18**_ (tables S20 to S22 and figs. S98 to S114), **(ZrPW**_**11**_**)**_**2**_ (tables S23 to S25 and figs. S115 to S130), **V**_**10**_ (tables S26 to S28 and figs. S131 to S148), **TeW**_**6**_ (fig. S149), and **SiW**_**12**_ (fig. S30).

### pH—The main factor in POM speciation

**Fig. 2.**
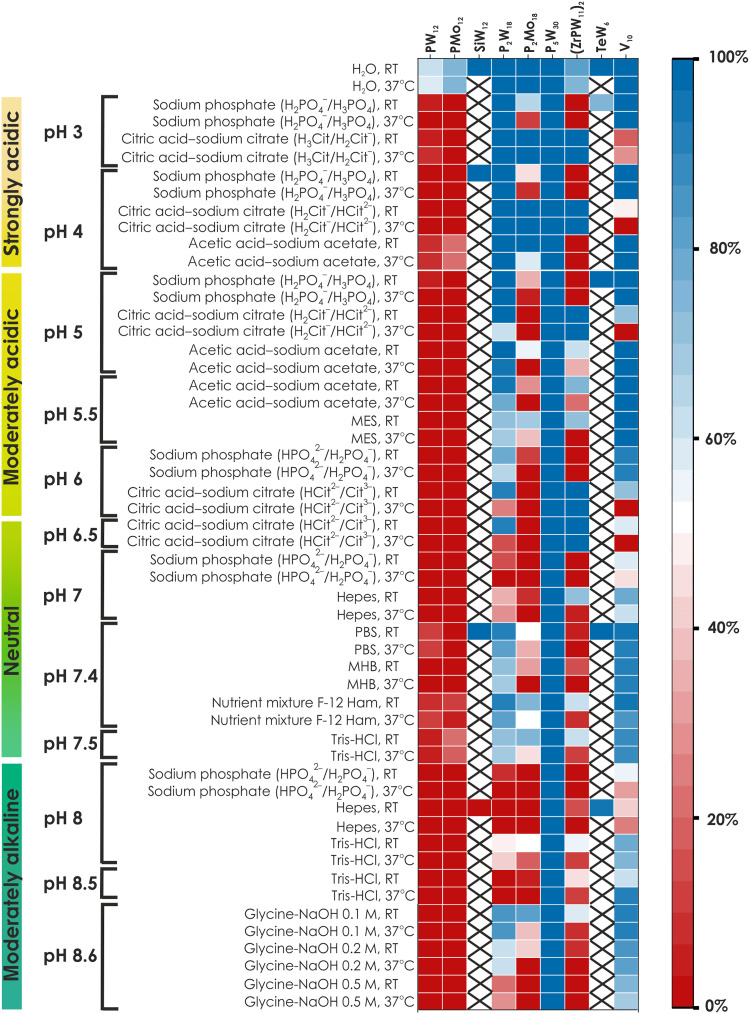
“Heatmap” of the POM stability. The stability heatmap shows the percentage of the initial anion present in solution at room temperature and after 24 hours incubation at 37°C. The map was created on the basis of the integrated data from ^31^P [**PW**_**12**_, **P**_**2**_**W**_**18**_, **P**_**5**_**W**_**30**_, **PMo**_**12**_, **P**_**2**_**Mo**_**18**_, and **(ZrPW**_**11**_**)**_**2**_], ^51^V (**V**_**10**_), and ^183^W (**TeW**_**6**_ and **SiW**_**12**_) NMR spectra (see the Supplementary Materials). The stability of α/β **-P**_**2**_**W**_**18**_ is the same as that of **P**_**2**_**W**_**18**_, only with different isomer distribution; therefore, α/β **-P**_**2**_**W**_**18**_ is not shown additionally on this heatmap. X in columns **TeW**_**6**_ and **SiW**_**12**_ stands for “not determined.” RT, room temperature.

The speciation in POM solutions is strongly dependent on pH (Fig. 2) since the formation of POMs through polycondensation is a function of pH (*1*). It is generally accepted that POMs prefer an acidic environment and their stability toward basic hydrolysis increases with the absolute negative net charge of the POM anion (*1*). Since this is the only general rule for archetype- and addenda metal type–dependent transformations of POM in aqueous solutions, the speciation of each POM must be considered individually. The pH range from 3 to 8.6 examined here is divided into four regions: strongly acidic 3 ≤ pH ≤ 4, moderately acidic 5 ≤ pH ≤ 6, neutral 6.5 ≤ pH ≤ 7.5, and moderately alkaline 8 ≤ pH ≤ 8.6 (Fig. 1). Three anionic buffers that have p*K*_a_ values (where *K*_a_ is the acid dissociation constant) close to the chosen pH (Table 1) that are commonly used (table S3) are 0.1 M sodium phosphate at pH 3 and 4, 0.1 M citric acid–sodium citrate at pH 3 and 4, and 0.1 M acetic acid–sodium acetate at pH 4. The buffer solutions with appropriate p*K*_a_ values (Table 1) in a moderately acidic environment 5 ≤ pH ≤ 6 are the three anionic 0.1 M sodium phosphate at pH 5 and 6, 0.1 M citric acid–sodium citrate at pH 5 and 6, and 0.1 M acetic acid–sodium acetate at pH 5 and 5.5 and the zwitterionic 0.1 M MES (fig. S2) (*31*) at pH 5.5. The neutral pH from 6.5 to 7.5 is often realized under physiological conditions, and seven different buffer solutions (anionic and zwitterionic) and media with appropriate p*K*_a_ values (Table 1) were tested: 0.1 M citric acid–sodium citrate at pH 6.5, 0.1 M sodium phosphate at pH 7, 0.1 M Hepes (fig. S2) (*31*) at pH 7, PBS at pH 7.4, MHB at pH 7.4, nutrient mixture F-12 Ham at pH 7.4, and 0.1 M tris-HCl (fig. S2) (*31*) at pH 7.5. For the moderately alkaline range, four types of buffer solutions were used: 0.1 M sodium phosphate at pH 8; 0.1 M tris-HCl at pH 8.0 and 8.5; 0.1 M Hepes at pH 8; and 0.1, 0.2, and 0.5 M glycine-NaOH at pH 8.6.

### Temperature

Similar to many other chemical reactions, hydrolysis can be accelerated by increasing the temperature. An increase in temperature to 37°C does not play a notable role in the case of completely stable (e.g., Preyssler **P**_**5**_**W**_**30**_) and unstable (e.g., Keggin **PW**_**12**_) POMs that are hydrolyzed already at room temperature. For all other POMs, a 24-hour incubation at 37°С often leads to a several-fold decrease in the concentration of the parent POM. **P**_**2**_**W**_**18**_ as an example is present at about 90% in 0.1 M citric acid–sodium citrate at pH 6.5 and room temperature, and after 24-hour incubation at 37°C, its concentration drops to 15% (tables S10 and S11).

### The speciation of PW_12_, P_2_W_18_, and P_5_W_30_ in a strongly acidic environment 3 ≤ pH ≤ 4

**Fig. 3.**
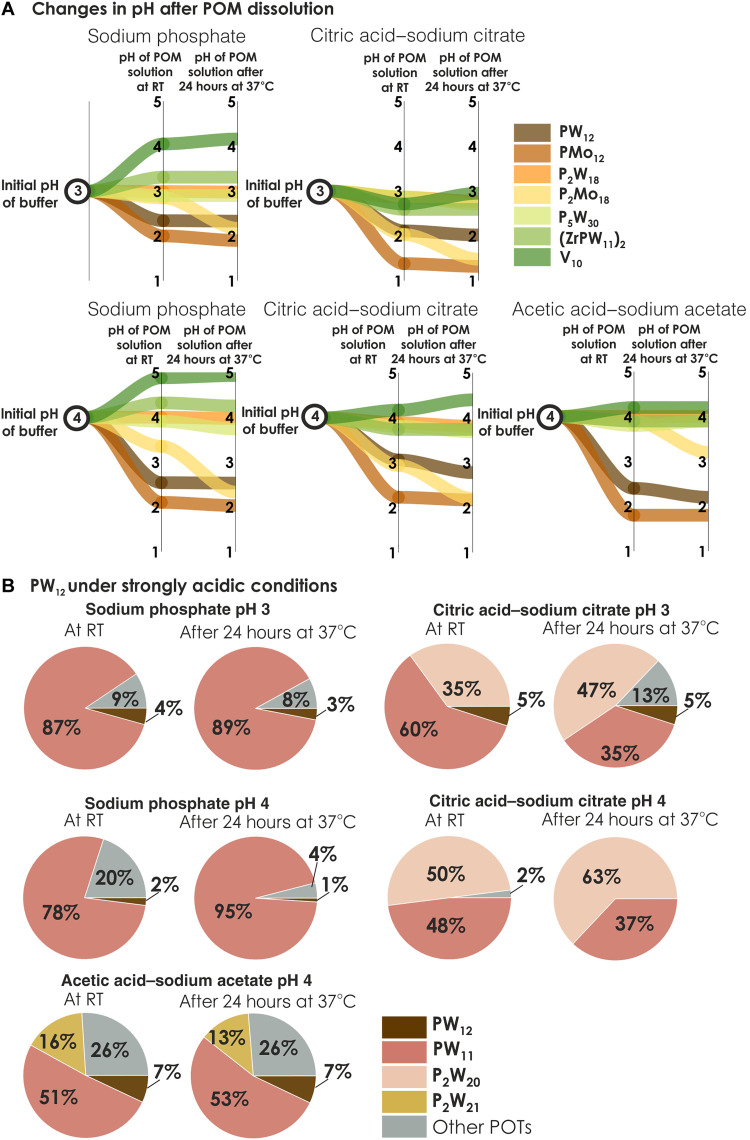
POMs’ behavior in a strongly acidic environment. (**A**) Changes in pH after POM dissolution at room temperature and after 24-hour incubation at 37°C in five buffers applied between pH 3 and 4 (tables S5, S8, S9, S14, S17, S20, S23, and S26). In solutions of stable POMs [**P**_**2**_**W**_**18**_, **P**_**5**_**W**_**30**_, **(ZrPW**_**11**_**)**_**2**_, and **V**_**10**_], the pH change from the starting pH of a buffer itself is minor, while for Keggin POT **PW**_**12**_ and POMo **PMo**_**12**_, the pH value drops by two pH units. The increase in temperature can also lead to a decrease in pH, as can be clearly seen in the case of **P**_**2**_**Mo**_**18**_. (**B**) Pie chart showing percentages of P-containing POT species in 10 mM solutions of **PW**_**12**_ in 0.1 M buffers with pH 3 and 4. POM concentration values are given as mean values; SDs are given in tables S6 and S7. The structures and full formulas of all anions are shown in fig. S11. The percentages for **P**_**2**_**W**_**18**_ and **P**_**5**_**W**_**30**_ tend to be close to 100% (tables S10, S11, S15, and S16) and therefore are not shown in the figure. To identify the individual anions, they are shown in different colors, with the same color code being selected for a specific anion throughout all figures and tables in this manuscript and the Supplementary Materials.

For Keggin-type POT **PW**_**12**_ (charge of −3 in the solid state), the pH immediately decreased by 0.5 to 1 U after its dissolution in all five buffers studied between pH 3 and 4 (Fig. 3A, table S5, and fig. S12), indicating the instability of the anion even under these acidic conditions. **PW**_**12**_ is present in low amounts (<5%; tables S6 and S7 and figs. S23 to S25), and the predominant anion is its monolacunary form [PW_11_O_39_]^7−^ (**PW**_**11**_), the percentage of which is strongly dependent on the type of buffer, where, at pH 4, the content of **PW**_**11**_ is around 78% in phosphate buffer, 48% in citrate buffer, and 51% in acetate buffer (Fig. 3B, table S6, and figs. S23 to S25). The incubation for 24 hours at 37°C does not affect speciation in acetic acid–sodium acetate buffer but leads to an increase in the concentration of **PW**_**11**_ in sodium phosphate solutions at pH 4 from 78 to 95%. In citric acid–sodium citrate buffer at pH 4, the content of **PW**_**11**_ decreases from 48 to 37% after incubation (table S7 and figs. S23 to S25) due to the formation ~60% of [P_2_W_20_O_70_(H_2_O)_2_]^10−^ (**P**_**2**_**W**_**20**_), which is formed in solution after **PW**_**12**_ hydrolysis (Fig. 3B and figs. S11 and S25) (*37*). The Wells-Dawson **P**_**2**_**W**_**18**_ (tables S10 and S11 and figs. S53 to S55) and the Preyssler **P**_**5**_**W**_**30**_ (tables S15 and S16 and fig. S79) anions remain intact and are present in concentrations close to 100% even after 24-hour incubation at 37°C in all five buffers tested between pH 3 and 4 (Fig. 2).

### The speciation of PW_12_, P_2_W_18_, and P_5_W_30_ in a moderately acidic environment 5 ≤ pH ≤ 6

Under moderately acidic conditions, intact **PW**_**12**_ is virtually absent at room temperature and consequently after 24-hour incubation at 37°C, and the monolacunary form **PW**_**11**_ is the predominant species (˃70%; tables S6 and S7), along with newly formed [P_2_W_20_O_70_(H_2_O)_2_]^10−^ (**P**_**2**_**W**_**20**_) and [P_2_W_21_O_71_(H_2_O)_3_]^6−^ (**P**_**2**_**W**_**21**_) (tables S6 and S7).

The Wells-Dawson **P**_**2**_**W**_**18**_ shows stability at pH 5 in sodium phosphate buffer (99%; tables S10 and S11) and acetic acid–sodium acetate (95%; tables S10 and S11) solutions even after incubation at 37°C. At pH 5 in citric acid–sodium citrate buffer and at pH 5.5 in acetic acid–sodium acetate buffer, **P**_**2**_**W**_**18**_ is almost 100% present at room temperature, but after 24 hours at 37°C, about 30% is converted to the monolacunary anion [P_2_W_17_O_61_]^10−^ (**P**_**2**_**W**_**17**_) (tables S10 and S11 and fig. S53). At pH 5.5 in MES buffer and at pH 6 in sodium phosphate, ~20% (table S10) of the intact Wells-Dawson phosphotungstate is hydrolyzed immediately, and after keeping the solution at 37°C for 24 hours, the degree of hydrolysis increases to 35% (table S11).

The Preyssler **P**_**5**_**W**_**30**_ anion remains intact and is present at concentrations approaching 100% (table S15) even after 24-hour incubation at 37°C. It is the most stable POM between pH 5 and 6 under the conditions applied (table S16 and fig. S79).

### The speciation of PW_12_, P_2_W_18_, and P_5_W_30_ in a neutral environment 6.5 ≤ pH ≤ 7.5

No intact **PW**_**12**_ exists in neutral buffer solutions, with the exception of a small amount (~10%) of **PW**_**12**_ in tris-HCl at pH 7.5, PBS, and nutrient mixture F-12 Ham (tables S6 and S7 and figs. S18 and S20). In all **PW**_**12**_ solutions, the monolacunary **PW**_**11**_ is the predominant species at room temperature with a concentration of 50 to 60% in sodium phosphate at pH 7, PBS, nutrient mixture F-12 Ham, and tris-HCl at pH 7.5 and a concentration of 80 to 100% in citric acid–sodium citrate at pH 6.5, Hepes at pH 7, and MHB (tables S6 and S7 and figs. S18 to S20 and S26 to S28). Incubation for 24 hours at 37°C changes speciation only in sodium phosphate at pH 7, increasing the presence of [P_2_W_5_O_23_]^6−^ (**P**_**2**_**W**_**5**_) from 34 to 66% (tables S6 and S7) by decreasing the concentration of **PW**_**11**_.

The Wells-Dawson **P**_**2**_**W**_**18**_ is stable (about 80 to 90% independent of temperature increase) at pH 7.4 in PBS, MHB, and nutrient mixture F-12 Ham solutions, in which the concentration of the buffer components is known and below 0.1 M [e.g., PBS with the standard concentration of HPO_4_^2−^ of 10 mM and H_2_PO_4_^−^ of 1.8 mM (*34*)] or is not fully known because of the complex composition (MHB and nutrient mixture F-12 Ham) but presumably below 0.1 M. In 0.1 M citric acid–sodium citrate at pH 6.5, ~90% of intact anion was detected in freshly prepared solution, but after 24-hour incubation at 37°C, **P**_**2**_**W**_**18**_ almost completely converted to the monolacunary anion **P**_**2**_**W**_**17**_ (70%) (tables S10 and S11 and figs. S37 and S55). In solutions with an initial pH of 7 at room temperature, **P**_**2**_**W**_**18**_ is present at concentrations less than 35% and at even lower concentrations after incubation at 37°C. The tris-HCl solution does not follow this trend and contains 70% of intact **P**_**2**_**W**_**18**_ anions despite its initial pH of 7.5, with or without incubation (tables S10 and S11 and figs. S39 and S56), suggesting a lower ability of tris-HCl to maintain the desired pH of 7.5 (measured values, ~5.5), resulting in less hydrolysis due to the lower pH.

Again, the Preyssler **P**_**5**_**W**_**30**_ anion remains intact and is present at concentrations approaching 100% in all neutral buffer solutions even after 24-hour incubation at 37°C, therewith being the most stable POM at a pH between 6.5 and 7.5 (tables S15 and S16 and fig. S79).

### The speciation of PW_12_, P_2_W_18_, and P_5_W_30_ in a moderately alkaline environment 8 ≤ pH ≤ 8.6

As expected, the pH discrepancy between the pH values of the buffer solution itself and after POM dissolution is largest for unstable anions (tables S5 and S8 and figs. S12D and S32D). Under alkaline conditions, intact **PW**_**12**_ was no longer present at pH 8, 8.5, and 8.6, and the lacunary Keggin anion **PW**_**11**_ predominates (tables S6 and S7). Thus, the Keggin POT in sodium phosphate at pH 8 converts to **PW**_**11**_ (63%) and **P**_**2**_**W**_**5**_ (37%) at room temperature (table S6), and the proportion of the latter anion increases to 63% after 24-hour incubation at 37°C, which is accompanied by a decrease in **PW**_**11**_ content (table S7 and fig. S24). In the solutions of **PW**_**12**_ in tris-HCl at pH 8 and in 0.1 and 0.2 M glycine-NaOH at room temperature and after 24-hour incubation at 37°C, there are two hydrolysis products—**PW**_**11**_ (65 to 82%) and **P**_**2**_**W**_**21**_ (tables S6 and S7). In Hepes at pH 8, tris-HCl at pH 8.5, and 0.5 M glycine-NaOH at pH 8.6, **PW**_**12**_ hydrolyzed completely to **PW**_**11**_ (100%) independent of the temperature applied.

The concentration of the intact Wells-Dawson **P**_**2**_**W**_**18**_ is only above 50% in 0.1 and 0.2 M glycine-NaOH, whereas in all other tested solutions, the monolacunary anion **P**_**2**_**W**_**17**_ predominates (tables S10 and S11). The higher stability of **P**_**2**_**W**_**18**_ in glycine-NaOH may be due to the insufficient buffering capacity (*34*) of this buffer solution, which is mainly used for immunological applications and not in inorganic systems. After the dissolution of **P**_**2**_**W**_**18**_ in sodium phosphate (pH 8) at room temperature, the intact Wells-Dawson anions hydrolyzed completely to a mixture of ~67% of **P**_**2**_**W**_**17**_ and ~33% of **P**_**2**_**W**_**5**_, and their composition does not change even after 24-hour incubation 37°C (tables S10 and S11 and fig. S54). The equilibria between **P**_**2**_**W**_**18**_ and its monolacunary form **P**_**2**_**W**_**17**_ are 1:1 in tris-HCl (pH 8), 6:1 in 0.1 M glycine-NaOH, 3:2 in 0.5 M glycine-NaOH, and 1:3 in 0.5 M glycine-NaOH independent of temperature. In Hepes at pH 8 and tris-HCl at pH 8.5, **P**_**2**_**W**_**18**_ transformed to **P**_**2**_**W**_**17,**_ which is almost exclusively present in solutions at room temperature and after 24-hour incubation at 37°C (table S11). Even under moderately basic conditions, the Preyssler **P**_**5**_**W**_**30**_ anion remains intact and is present at concentrations of almost 100%, again being the most stable POM at pH 8 and 8.6 (tables S15 and S16 and fig. S79).

### Buffer concentration

In catalysis, the type of buffer and its concentration are generally not key factors unless protons, hydroxide anions, or buffer components are directly involved in the catalytic mechanism (*29*). When choosing a buffer concentration in biology, the following rule often applies: “As low as possible as long as the pH value can be adequately controlled” (*34*). Often, POM transformations in aqueous media actively generate hydrogen ions, especially under alkaline conditions, which lead to a change in pH in the system and require higher buffer concentrations. To examine the concentration effect of the buffer itself, we investigated the most alkaline solution in this study, glycine-NaOH with pH 8.6, at three concentrations of 0.1, 0.2, and 0.5 M. Since the ratio of dissolved POM to buffer concentration (rather than absolute concentrations of both POM and buffer) is a key factor, the buffer concentration was varied because the NMR signal deteriorates with decreasing POM concentration and increasing in POM concentration is not possible in many cases due to its solubility limit.

pH values close to the initial pH of buffer were only observed for **P**_**5**_**W**_**30**_ solutions (table S14 and fig. S68), where only intact [NaP_5_W_30_O_114_]^14−^ is present at all concentrations (0.1, 0.2, and 0.5 M) of a glycine-NaOH buffer (tables S15 and S16). In all other cases [**PW**_**12**_, **PMo**_**12**_, **P**_**2**_**W**_**18**_, **α/β-P**_**2**_**W**_**18**_, **P**_**2**_**Mo**_**18**_, **(ZrPW**_**11**_**)**_**2**_, and **V**_**10**_], the decrease in pH is more pronounced the stronger the POM hydrolysis, and a pH decrease in several units is observed even in solutions with a buffer concentration of 0.5 M [e.g., for **PW**_**12**_, pH is 4.8 in 0.5 M glycine-NaOH (table S5 and fig. S12), or for **PMo**_**12**_, pH is 4.0 in 0.5 M glycine-NaOH (table S17 and fig. S81)]. The proportion of hydrolyzed **P**_**2**_**W**_**18**_ anion at room temperature in a 0.1 M glycine-NaOH solution (pH after POM dissolution is 5.8) is 14%, that in 0.2 M (pH after POM dissolution is 6.0) is 40%, and that in 0.5 M (pH after POM dissolution is 6.2) is up to 78% (tables S15 and S16 and fig. S59). An increase in the buffer concentration with alkaline pH values is accompanied by a higher buffer capacity, and as a result, it leads to a greater degree of hydrolysis if POM is subjected to it at a given pH value. To maintain pH under alkaline conditions, a higher concentration buffer can be applied; however, this can entail a greater degree of POMs’ degradation. For acidic pH regions, where POMs are usually more stable, the effect of buffer concentration is less important (fig. S35).

### Ionic strength of buffers

Per definition, the ionic strength (*I*) of a salt solution is half of the total obtained by multiplying the molarity of each ion by its valence squared (eq. S1). (*34*) For zwitterionic buffers, their contribution to ionic strength is small, which has been reported (*38*), although MES [SO_3_^−^; (morpholino)NH^+^], Hepes [SO_3_^−^; (piperizine)NH^+^], and glycine (COO^−^; NH_3_^+^) (fig. S2) have large dipole moments but still behave mostly like uncharged species (*33*). For anionic systems, polyvalent buffers such as phosphate, which can carry multiple negative charges (here from −1 to −3), have a much higher ionic strength than monovalent buffers such as acetic acid–sodium acetate (CHCOO^−^/Na^+^) or tris-HCl (NH_3_^+^/Cl^−^) (*34*). The ionic strength of anionic buffers is pH dependent (*33*), because of the ratio of ionic components according to the Henderson-Hasselbalch equation (*39*) (see Materials and Methods, Eq. 1). If POMs are very stable (e.g., **P**_**5**_**W**_**30**_) or, conversely, very unstable (e.g. **PW**_**12**_), it is practically impossible to trace the influence of ionic strength, as **P**_**5**_**W**_**30**_ is stable under all conditions and **PW**_**12**_ is hydrolyzed independent of the buffer type. The influence of ionic strength is demonstrated in the behavior of two Wells-Dawson anions **P**_**2**_**Mo**_**18**_ and **P**_**2**_**W**_**18**_ at pH 8. Both are more stable (concentration of 53% of [P_2_Mo_18_O_62_]^6−^ and 48% of [P_2_W_18_O_62_]^6−^ at room temperature; tables S10 and S21) in 0.1 M tris-HCl buffer (trisH^+^ and Cl^−^) with low *I* = 0.05 than in 0.1 M sodium phosphate (H_2_PO_4_^−^/HPO_4_^2−^) with higher *I* = 0.23 (concentration of 0% of [P_2_Mo_18_O_62_]^6−^ and 9% of [P_2_W_18_O_62_]^6−^; for *I* calculation, tables S10 and S21). The difference in ionic strength affects the stability of the POM since the more negatively charged components of the buffer (higher *I*) can act as proximal bases and cause a local increase in pH, accelerating decomposition or rearrangement (*29*). To distinguish between effects caused by pH or ionic strength in buffers of different ionic strengths, we add neutral salts to bring the ionic strength to a constant value. Thus, an increase in ionic strength to *I* = 0.20 in a solution of **P**_**2**_**Mo**_**18**_ in 0.1 M tris-HCl buffer (*I* = 0.05) at pH 8 by the addition of a strong electrolyte (0.15 M NaNO_3_) leads to a decrease in the concentration of the intact anion **P**_**2**_**Mo**_**18**_ [P_2_Mo_18_O_62_]^6−^ from 53 to 45% (table S29), slightly offsetting the initial discrepancy with 0.1 M sodium phosphate (H_2_PO_4_^−^/HPO_4_^2−^) at pH 8 and making the comparison of two buffer solutions more accurate.

### Chelation of metal ions and its influence on speciation

Most common buffer (e.g., acetic acid-sodium acetate and phosphate) species have very weak metal ion binding capacity. However, a citrate buffer can be a very good chelating agent for many metal ions, reducing their effective concentration in solution. Since the conjugate acid and base (e.g., Cit^3−^ and HCit^2−^) have different metal ion affinities, their ability to bind the metal ion is pH dependent. The most notable example is the formation of vanadium-citrate (**V-cit**) complexes in decavanadate **V**_**10**_ solutions (tables S27 and S28 and figs. S136 and S144). Thus, **V-cit** complexes are present in 0.1 M citric acid–sodium citrate solutions in the pH range from 3 to 6.5, with the different concentrations at different pH values (fig. S144), which can be explained in a simplified way considering the affinity of Cit^3−^ (pH > 5) to V^V^ compared to its protonated forms HCit^2−^ (4 < pH < 5) and H_2_Cit^−^ (pH < 4). However, the stability of **V**_**10**_ and its decomposition products make the picture more complex and have to be considered. As a result, the use of a citrate buffer for vanadium-containing POMs is not recommended (*40*). Comparison of the Keggin tungstate and molybdate analogs, **PW**_**12**_ and **PMo**_**12**_, shows the higher affinity of Mo^VI^ for citrate (the formation constants for Mo^VI^-Cit complexes logβ are reported in the range of 8.35 up to 77.45) (*41*) compared to W^VI^ (the formation constants for W^VI^-Cit complexes logβ are reported in the range of 10.21 up to 39.30) (*42*), leading to the formation of **Mo-cit** complexes in 0.1 M citric acid–sodium citrate solutions with pH > 4 (figs. S86 and S94). Both examples (**V**_**10**_ and **PW**_**12**_ versus **PMo**_**12**_) show that the chelation of addenda ions has a substantial impact on the stability of POMs. Therefore, the choice of buffer type must consider the affinity of its components to metal (addenda and hetero) ions.

### Redox processes caused by medium and buffer components

Oxidized and reduced POMs usually have the same structure; however, reduced POMos and POTs have a characteristic deep blue color due to intense *d-d *transitions and intervalence charge transfer, which helps to identify and track the reduction immediately (*9*). In inorganic synthesis, strong reducing agents such as B_2_H_6_, NaBH_4_, N_2_H_4_, and NH_2_OH are used to reduce POMs (*9*). However, the reduction of POMs was already observed under “milder” conditions (*35*) [e.g., reduction of **P**_**2**_**W**_**18**_ in methicillin-resistant *Staphylococcus aureus* (*43*)], which is due to the buffer or medium components. Our atlas showed that in MHB medium at pH 7.4, **P**_**2**_**W**_**18**_, **P**_**5**_**W**_**30,**_ and **P**_**2**_**Mo**_**18**_ in MHB medium at pH 7.4 were reduced to [P_2_W^V^*_l_*W^VI^_(18–*l*)_O_62_]^(6+*l*)–^, [NaP_5_W^V^*_m_*W^VI^_(30−*n*)_O_114_]^(6+*m*)–^, and [P_2_Mo^V^*_n_*Mo^VI^_(18–*n*)_O_62_]^(6+*n*)–^ (where *l*, *m*, and *n* are the numbers of accepted electrons), respectively, but the signals in the ^31^P spectra were not strong enough to be detected. To identify the reducing components of MHB medium, we individually investigated the redox activity of its three components—beef extract, casein hydrolysate, and starch—by ultraviolet-visible spectroscopy showing that casein hydrolysate and starch do not exhibit reducing activity (fig. S150), making the complex composition of the beef extract responsible for the POM reduction. In Hepes at pH 7 and 8, **P**_**2**_**Mo**_**18**_ and **PMo**_**12**_ also turned blue, and, in their ^31^P NMR spectra, a low-intensity signal was observed at −4.6 parts per million (tables S18, S19, S21, and S22 and figs. S89 and S106), which corresponds to the reduced derivatives [P_2_Mo^V^*_n_*Mo^VI^_(18–*n*)_O_62_]^(6+*n*)–^ (*n* = 1, 2) (*44*). The piperazine moiety in Hepes (fig. S2) generates nitrogen-centered free radicals that can act as a weak reducing agent (*45*), which, combined with the “electron-sponging” nature of POMos, leads to a reduction in contrast to **PW**_**12**_ and **P**_**5**_**W**_**30**_ (*9*). These examples clearly show that the absence of an additionally added reducing agent does not guarantee the absence of redox processes in the POM solution. Given the importance of redox processes in both biology and catalysis, reduced POM anions can represent the active species, and it is therefore highly recommended to test the ability of a buffer or medium to reduce a POM.

### The influence of counter cations on speciation

The most common choice of cations for POM anions is alkali metal ions, which make them easily soluble in an aqueous medium and usually do not affect the behavior of solutions, mainly interacting with POMs electrostatically (*36*). Here, only POMs with simple monovalent cations (alkali metals and NH_4_^+^) have been investigated. The effect of chemically diverse cations such as dendrimers, polyvalent metals, metal complexes, amphiphiles, and alkaloids has so far been investigated as structural-directed functions for POM assembly (*36*), and their effect on speciation is a topic in its own right.

### Superposition of factors influencing speciation

The analysis of the atlas showed that the pH, temperature, concentration, and ionic strength of a buffer are factors that act in synergy influencing POM degradation. The investigated POMs showed that temperature, increased buffer concentration, and a high ionic strength themselves often do not cause POM degradation but intensify existing hydrolysis processes and this all the more, the higher the pH value of the solution. Factors such as chelating and reducing agents act independently. Reduction processes become more noticeable during incubation, which depends not only on temperature but also on the exposure time.

### Reanalysis of published data based on the speciation atlas

**Fig. 4.**
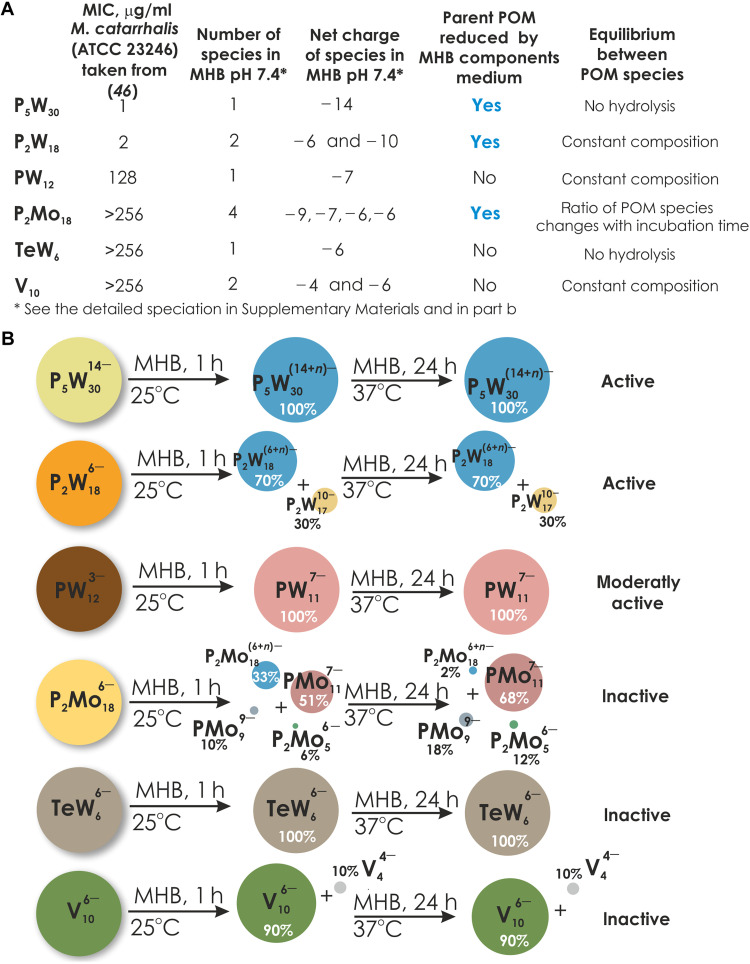
POMs against *M. catarrhalis*: An example of the atlas usage. (**A**) Summary of antibacterial activity experiments for six POMs—**PW**_**12**_, **P**_**2**_**W**_**18**_, **P**_**2**_**Mo**_**18**_, **P**_**5**_**W**_**30**_, **TeW**_**6**_, and **V**_**10**_. The minimum inhibitory concentration (MIC) values are taken from (*46*). The solution characteristics in MHB are the results of this study. “No hydrolysis” means that parent POM does not undergo any transformation in solution; “constant composition” corresponds to a situation when POM has been hydrolyzed, but the ratio between species remains stable over incubation time. (**B**) The simplified speciation schemes for **PW**_**12**_, **P**_**2**_**W**_**18**_, **P**_**2**_**Mo**_**18**_, **P**_**5**_**W**_**30**_, **TeW**_**6**_, and **V**_**10**_. For more details, see tables S6, S7, S10, S11, S15, S16, S21, S22, S27, and S28. A blue color for **P**_**5**_**W**_**30**_, **P**_**2**_**W**_**18**_, and **P**_**2**_**Mo**_**18**_ after their explosion to MHB indicates their reduction with *n* electrons. To identify the individual anions, they are shown in different colors, with the same color code being selected for a specific anion throughout all figures and tables in this manuscript and the Supplementary Materials.

This speciation atlas enables reinterpreting already published data. In 2018 (*46*), our team screened 29 different POMs as antibacterial agents against *Moraxella catarrhalis* in MHB medium after 20- to 22-hour incubation at 37°C. In (*46*), we related the antibacterial activity of active phosphorous-containing POTs against *M. catarrhalis* to an increase in POTs’ total charge density (the ratio between a number of addenda metal ions to a net charge), based only on solid-state structural data, and did not consider for individual speciation in solution. Six (active against *M. catarrhalis*—**PW**_**12**_, **P**_**2**_**W**_**18**_, and **P**_**5**_**W**_**30**_; inactive against *M. catarrhalis*—**P**_**2**_**Mo**_**18**_, **TeW**_**6**_, and **V**_**10**_) of the previously investigated POMs (*46*) are part of our atlas (Fig. 4). Reanalysis now indicates the species present in solutions that may be responsible for activity against *M. catarrhalis* (minimum inhibitory concentration <128 μg/ml). The species present in MHB solutions of **P**_**5**_**W**_**30**_ are the oxidized and reduced forms of [NaP_5_W_30_O_114_]^14−^. In the MHB solution of **P**_**2**_**W**_**18**_, it is a combination of [P_2_W_18_O_62_]^6−^ in its oxidized and reduced form with [P_2_W_17_O_61_]^10−^, and in MHB solutions of **PW**_**12**_, [PW_11_O_39_]^7−^ is the predominant species (Fig. 4 and tables S7, S11, and S16). In two of the most active POM solutions **P**_**5**_**W**_**30**_ and **P**_**2**_**W**_**18**_, the parent species have a higher negative charge (<−6) compared to other tested POMs, which was already concluded in 2018 (*46*). However, the reducibility of POMs appears to be a critical factor that plays an important role in determining their activity against bacteria, which has not often been considered in antibacterial tests but has already been mentioned as an important ability in biological systems (*35*). Because of its infrequent mention in antibacterial studies, it can be challenging to estimate the precise role of reduction in POMs’ antibacterial activity. Hence, it is essential to take it into account when designing bioactive POMs. This is supported by our recent study (*47*), where we observed that the activity trend of Al-containing POMs could be attributed to their varying redox activity. We found that POMs with a higher tendency toward reduction exhibited increased antibacterial activity, resulting in enhanced potency against bacteria (*47*). In the **P**_**2**_**Mo**_**18**_ MHB solution, the equilibrium in this solution is not stable over time, and the concentration of real active species might be too low (Fig. 4), emphasizing how difficult it can be to assign real active species, even when the speciation is known.

## DISCUSSION

**Fig. 5.**
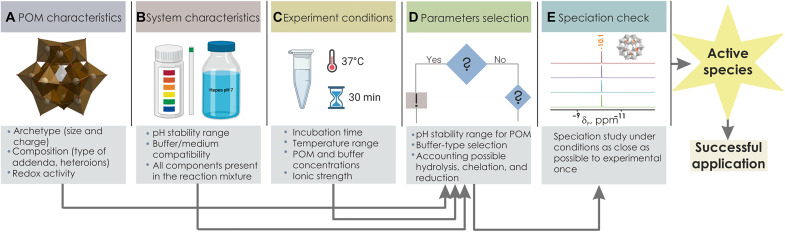
A roadmap presenting the experiment design for POM applications in aqueous solutions. The key steps in experiment planning that include the POM choice and characterization of the application system: (**A**) POM selection; (**B** and **C**) Characterization of the system in which POM is applied; (**D** and **E**) POM speciation check. Created with Biorender.com. ppm, parts per million.

Reporting stability information is becoming a good practice in the POM community from inorganic (anion conversion), catalytic (postcatalytic analysis), and biochemical (active species) perspectives but requires wider dissemination and initial good and detailed planning. To support speciation investigation in future studies, a roadmap for planning and conducting experiments with compounds that tend to transform in aqueous solutions, exemplified here on POMs, is proposed (Fig. 5):

1) POM selection. If the POM properties (e.g., size, charge, and addenda type) required for a particular activity are known in advance, then we select several POM variants that are likely to be stable under the given conditions or hydrolyze (transform) to the desired active species (Fig. 5A). The atlas proves that the following POM characteristics are key points to consider when selecting a POM: (i) The charge. The higher the negative charge (preferably <–6), the more stable the POM at neutral pH (>6.5). POMs with a low negative charge (<−3) are unstable even in acidic regions and often form more than one hydrolysis product. (ii) Addenda ion type and archetype. Two anions of the same archetype with different addenda ions are likely to show different speciations. Thus, the POMos of Keggin and Wells-Dawson archetypes tend to form more complex mixtures after hydrolysis of the intact species as compared to the POT analogs. Wells-Dawson POMo also shows lower stability than the corresponding POT. The more inert the addenda metal ion (inertness increases in a row V^V^-Mo^VI^-W^VI^), the lower the chance of chelation by buffer components. (iii) Redox activity. The intact Wells-Dawson and Preyssler POM archetypes show a high tendency to be reduced in buffer/medium solutions, which can be caused by a combination of reducing buffer components being present and the electron-sponging nature of POMs. When intact POMs are not a high priority, note that lacunary POMs are less likely to be reduced. If your study is designed for screening, then selecting POMs of different archetypes and charges that can be postmodified and have active nuclei (e.g., ^31^P and ^51^V) for NMR analysis is preferable, which will greatly facilitate subsequent speciation studies.

While our findings are based on six archetypes and three types of addenda ions and they can be extrapolated with some accuracy to other POM classes, there are several important POM classes, such as polyoxoniobates and polyoxotantalates that are stable under alkali conditions, noble-metal POMs and molybdenum blues that are not covered in this study. We encourage synthetic specialists in these fields to continue collecting data on their stability, which will eventually expand the atlas and provide more insights into POM solution chemistry. There is still a considerable amount of data to be collected on POM speciation in nonaqueous media, which would greatly benefit applications in various areas, including catalysis, electrochemistry, and materials science.

2) Characterization of the system in which the POM is applied. List all application system parameters, including possible variable buffer types, pH range, incubation time, concentration of buffer, temperature, etc. (Fig. 5, B and C). We have shown that anionic buffers consisting of monovalent anions are the best choice to keep the pH close to the initial pH of the buffer, but the tendency of buffer components to chelate must be considered. So-called Good’s zwitterionic buffers, very common in biology, do not show a high ability to maintain the pH in POM solutions. Among the tested buffers for the acidic region, acetic acid–sodium acetate and sodium phosphate are recommended, whereas citric acid–sodium citrate can only be applied to systems that do not tend to form complexes with citrate. For the moderately acidic and neutral pH region, acetic acid–sodium acetate, MES, and sodium phosphate are the buffers of choice (for more details, see the Supplementary Materials). Under alkaline conditions, all tested buffers do not keep the pH well and promote hydrolysis of unstable (e.g., with net charge of <–6) POMs. As expected, the higher the buffer concentration, the more likely it is to maintain the pH upon the addition of POM; however, in an alkaline environment, we observed enhanced hydrolysis at a higher buffer concentration. Notice that tris itself can act as a binding ligand (*48*), and even be a critical template component for POM assembly (*49*). Hepes and MHB need to be considered as reducing buffers, in which reduction was observed for Wells-Dawson and Preyssler archetypes, where the piperazine moiety (Hepes) and parts of beef extract (MHB) act as reducing agents, respectively. If the aim is to minimize the decomposition of the parent anion, then the temperature and incubation time should be as low as possible. In any case, poststability analysis is of paramount importance (Fig. 5E).

3) POM speciation. The information collected and analyzed in subsections (1) and (2) serves as the basis for selecting the most appropriate POM and buffers for your application. Speciation studies should be carried out under conditions as close as possible to the planned experiments (not only the type of buffer and pH but also the presence of all components of the system) (Fig. 5, D and E). Furthermore, it should be noted that the presence of biomolecules in solution can impact the stability and behavior of POMs (*50*, *51*). In particular, POM-protein and POM-peptide interactions are complex and important areas of research that must be considered in many biological applications (*52*–*54*). These interactions can influence the efficacy and specificity of POMs in various biological contexts, making their study of great importance in the field. Even if incubation is not foreseen by the experiment, the stability of POM should be checked at elevated temperatures (in case of local temperature increase) and over time, which will give an idea of how stable the equilibrium in the system is and how long a POM stock solution can be stored. For the stability check, the choice of method is highly important and sometimes is not so straightforward. NMR spectroscopy is a reliable tool for the qualitative investigation of POMs in solutions and is preferred whenever possible. Electronic or mass spectrometry can also be used for quantitative stability checks. More advanced techniques such as small-angle x-ray scattering, x-ray absorption fine structure, and x-ray absorption near-edge structure, dynamic light scattering, and cyclic voltammetry can provide useful information but are not as widely used as other methods due to their limited availability and complexity of execution. Note that modern computational methods have the ability to predict the speciation map of POMs in solutions with considerable accuracy. The POMSimulator is a notable example of such a tool, which has successfully predicted the states of most POMs in solution (*55*, *56*). This computational method can be particularly useful for cases where experimental measurements are not possible because of low POM concentration or the presence of interfering components.

In conclusion, the speciation atlas contributes to the field of aquatic POM chemistry by first providing a knowledge base and cataloging the speciation profiles of the common POM archetypes to enable the community to access key stability characteristics for biological and catalytic insights into POM applications. Second, the atlas provides a resource of experimental data demonstrating the influence of unmanifest experimental parameters on the behavior of POMs in solution. The speciation atlas aims to improve the interdisciplinarity and multidisciplinary of POM chemistry with other fields, remove barriers to ambiguity about what happens in solution, and stimulate verification of the stability of the compounds in solution.

## MATERIALS AND METHODS

All chemicals were purchased from Sigma-Aldrich (Austria) and used without further purification. MHB (*32*) and nutrient mixture F-12 Ham (*33*) were purchased from Merck (Austria).

### Buffer characteristics and preparation

All buffers have an optimal pH range over which they are able to moderate changes in hydrogen ion concentration without changing the pH. This range is centered around the coefficient of the acid dissociation constant of the buffer (*K*_a_) and is generally defined as the p*K*_a_ (–log*K*_a_) value plus or minus one pH unit (*34*). To study the speciation of POMs in solution, seven different anionic and zwitterionic buffers and two media (MHB and nutrient mixture F-12 Ham) covering a pH range from 3 to 8.6 were selected (see fig. S2 for structural formulas of buffer components). In the acidic range, three anionic buffers: sodium phosphate NaH_2_PO_4_/H_3_PO_4_, and Na_2_HPO_4_/NaH_2_PO_4_ [pH 3 to 6; while phosphate does not buffer in the pH range of 3.5 to 5.5, experiments were conducted at this pH to provide comparisons to previously published studies (*29*)] (*57*); citric acid–sodium citrate Na_3_Cit/H_3_Cit (pH 3 to 6.5) (*57*); acetic acid–sodium acetate NaOAC/HOAc (pH 4 to 5.5) (*57*); and one zwitterionic buffer MES (pH 5.5) (*31*) have been examined. In neutral/basic region, three anionic: sodium phosphate Na_2_HPO_4_/NaH_2_PO_4_ (pH 7 to 8), PBS (pH 7.4), and tris-HCl (pH 7.5 to 8.5); two zwitterionic buffers: Hepes (pH 7 to 8) (*31*) and glycine-NaOH (pH 8.6); and two media: MHB (pH 7.4) and nutrient mixture F-12 Ham (pH 7.4) (Table 1) have been studied. All buffers were prepared at a concentration of 0.1 M using the Henderson-Hasselbalch equation (*39*)

$$\mathrm{pH}={pK}_{a}+\log_{10}\left(\frac{\left[\text{proton acceptor}\right]}{\left[\text{proton donor}\right]}\right)$$ (1)

### POM synthesis and characterization

K_6_[α-P_2_W_18_O_62_]·19H_2_O (**α-P**_**2**_**W**_**18**_) (*12*), (NH_4_)_6_[α/β-P_2_W_18_O_62_]·14H_2_O (**α/β-P**_**2**_**W**_**18**_) (*13*), (NH_4_)_6_[α-P_2_Mo_18_O_62_]·19H_2_O (**P**_**2**_**Mo**_**18**_) (*17*), K_12.5_Na_1.5_[NaP_5_W_30_O_114_]·19H_2_O (**P**_**5**_**W**_**30**_) (*14*), K_4_[SiW_12_O_40_]·8H_2_O (**SiW**_**12**_) (*19*), (Et_2_NH_2_)_8_[{α-P^V^W^VI^_11_O_39_Zr^IV^(μ-OH)(H_2_O)}_2_]·7H_2_O [**(ZrPW**_**11**_**)**_**2**_] (*15*), Na_2_K_4_[V_10_O_28_]·10H_2_O (**V**_**10**_) (*20*), and Na_6_[TeW_6_O_24_]·10H_2_O (**TeW**_**6**_) (*18*) were synthesized according to reported procedures. Na_3_[α-PW_12_O_40_]·8.5H_2_O (**PW**_**12**_) and H_3_[α-PMo_12_O_40_]·9H_2_O (**PMo**_**12**_) were purchased from Merck (Austria) as the commercially available compounds are commonly used in applications (table S3). All synthesized ionic compounds were characterized in solution by NMR spectroscopy (figs. S13 to S21, S30, S34 to S52, S67 to S77, S82 to S91, S100 to S108, S117 to S125, S133 to S141, and S149) and in solid-solid using infrared spectroscopy (figs. S3 to S7 and table S1) and thermogravimetric analysis (TGA) (figs. S8 to S10 and table S2).

### Speciation studies

All solutions were tested with a POM concentration of 10 mM and under 27 different conditions (see Table 1) immediately after the preparation of the solution and then after 24-hour incubation at 37°C. The concentration of buffer solutions was 0.1 M, except for two more concentrated solutions of glycine-NaOH (0.2 and 0.5 M) that are used to study the speciation behavior as a function of the buffer concentration. The pH value was determined on a Thermo Fisher Orion Star A211 benchtop pH meter with a Hamilton Biotrod electrode, which was calibrated before each series of measurements with at least two pH points using standard buffers from Sigma-Aldrich for calibration with pH 2.00 (citric acid/sodium hydroxide/hydrogen chloride), 4.01 (potassium hydrogen phthalate), and 7.00 (disodium hydrogen phosphate/potassium dihydrogen phosphate).

The speciation of phosphorous-containing POMs was investigated using ^31^P NMR spectroscopy. The relative abundance of all species was calculated on the basis of the integration of the ^31^P NMR peaks, considering only signals that can be assigned to POM species, ignoring free phosphate signals. For decavanadate ^51^V NMR and for **TeW**_**6**_ and **SiW**_**12**_, ^183^W NMR has been applied. ^31^P, ^51^V, and ^183^W NMR spectra were recorded on a Avance Neo 500-MHz FT-NMR spectrometer (Bruker, Rheinstetten, Germany) at 25°C. Chemical shifts were measured relative to 85% H_3_PO_4_, VOCl_3,_ and 1 M Na_2_WO_4_, respectively. The ^31^P NMR spectra were recorded at 202.53 MHz, and the ^51^V NMR samples were recorded at 131.60 MHz (2000 scans, accumulation time of 0.05 s, and relaxation delay of 0.01 s). The ^183^W NMR samples were prepared in 2.7 ml of buffer with a POM concentration of 20 mM and measured in 10-mm tubes. The experimental time was about 60 hours, with a standard pulse program at 20.836 MHz and a flip angle of 63° with a 1-s relaxation delay.

## Supplementary Materials

### This PDF file includes:

Figs. S1 to S151 Tables S1 to S29 References
