# Supplementary material for: Speciation atlas of polyoxometalates in aqueous solutions
Source: Sci Adv. 2023 Jun 21;9(25):eadi0814. doi: 10.1126/sciadv.adi0814 (PMC10284552; doi:10.1126/sciadv.adi0814)
Supplement: Supplementary file 1 — Figs. S1 to S151 Tables S1 to S29 References [file sciadv.adi0814_sm.pdf]

Science Advances
AAAS

Supplementary Materials for

Speciation atlas of polyoxometalates in aqueous solutions

Nadiia I. Gumerova and Annette Rompel

Corresponding author: Nadiia I. Gumerova, nadiia.gumerova@univie.ac.at;
Annette Rompel, annette.rompel@univie.ac.at

Sci. Adv. **9**, eadi0814 (2023)
DOI: 10.1126/sciadv.adi0814

This PDF file includes:

Figs. S1 to S151
Tables S1 to S29
References

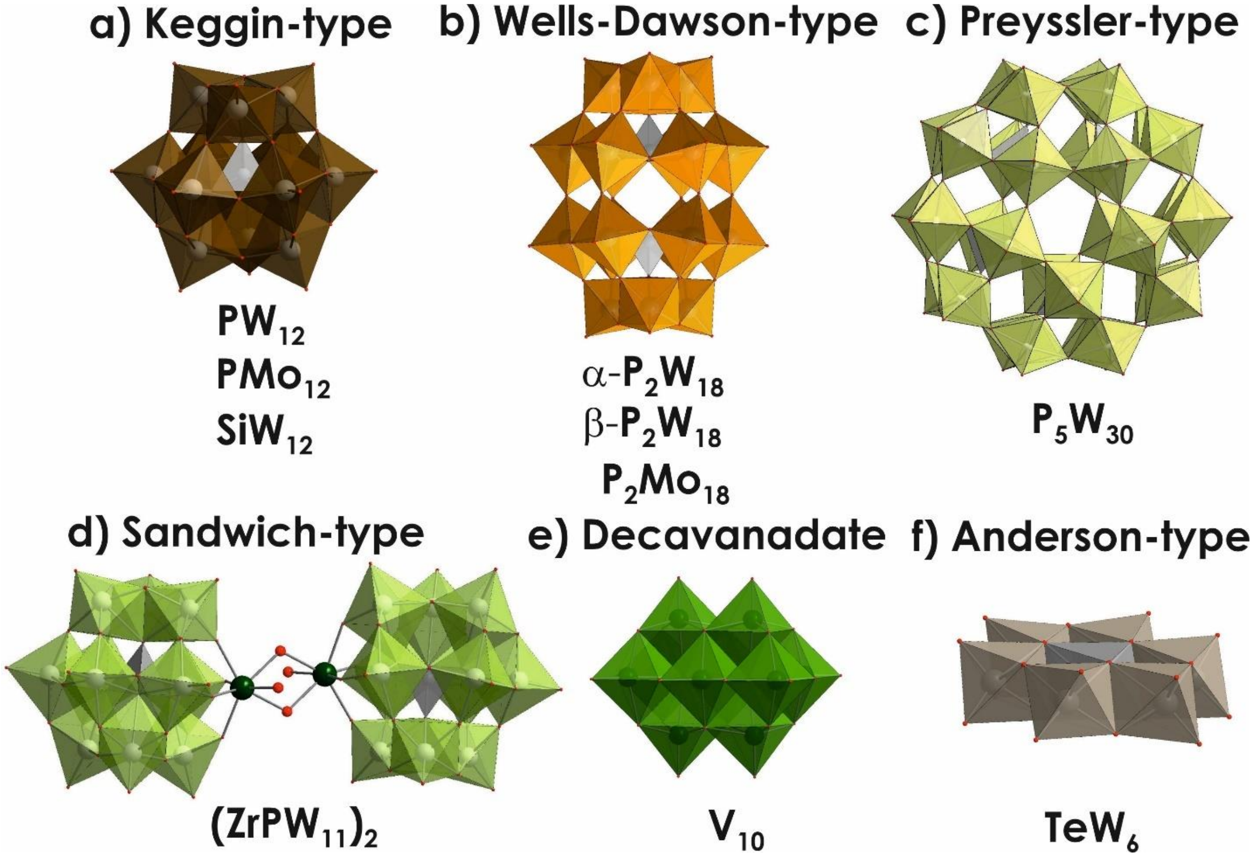

Fig. S1. POM archetypes.

Polyoxometalates used in the study: a) Keggin-type – $[\alpha\text{-}P^{V}W^{VI}_{12}O_{40}]^{3-}$ (**PW12**), $[\alpha\text{-}P^{V}Mo^{VI}_{12}O_{40}]^{3-}$ (**PMo12**), $[\alpha\text{-}Si^{IV}W^{VI}_{12}O_{40}]^{4-}$ (**SiW12**); b) Wells-Dawson-type – $[\alpha\text{-}P^{V}_{2}W^{VI}_{18}O_{62}]^{6-}$ (**P2W18**), $[\alpha/\beta\text{-}P^{V}_{2}W^{VI}_{18}O_{62}]^{6-}$ (***α/β*-P2W18**), $[\alpha\text{-}P^{V}_{2}Mo^{VI}_{18}O_{62}]^{6-}$ (**P2Mo18**); c) Preyssler-type $[NaP^{V}_{5}W^{VI}_{30}O_{110}]^{14-}$ (**P5W30)**; Keggin-based sandwich $[\{\alpha\text{-}P^{V}W^{VI}_{11}O_{39}Zr^{IV}(\mu\text{-}OH)(H_2O)\}_2]^{8-}$ (**(ZrPW11)2**); e) decavanadate, $[V^{V}_{10}O_{28}]^{6-}$ (**V10**); f) $[Te^{VI}W^{VI}_{6}O_{24}]^{6-}$ (**TeW6**). Color code: $\{WO_6\}$, brown, orange, yellow-green, light-green, brown-grey; $\{MoO_6\}$, brown, orange; $\{VO_6\}$, green; $\{PO_4\}$, light green; O, red; Zr, dark green.

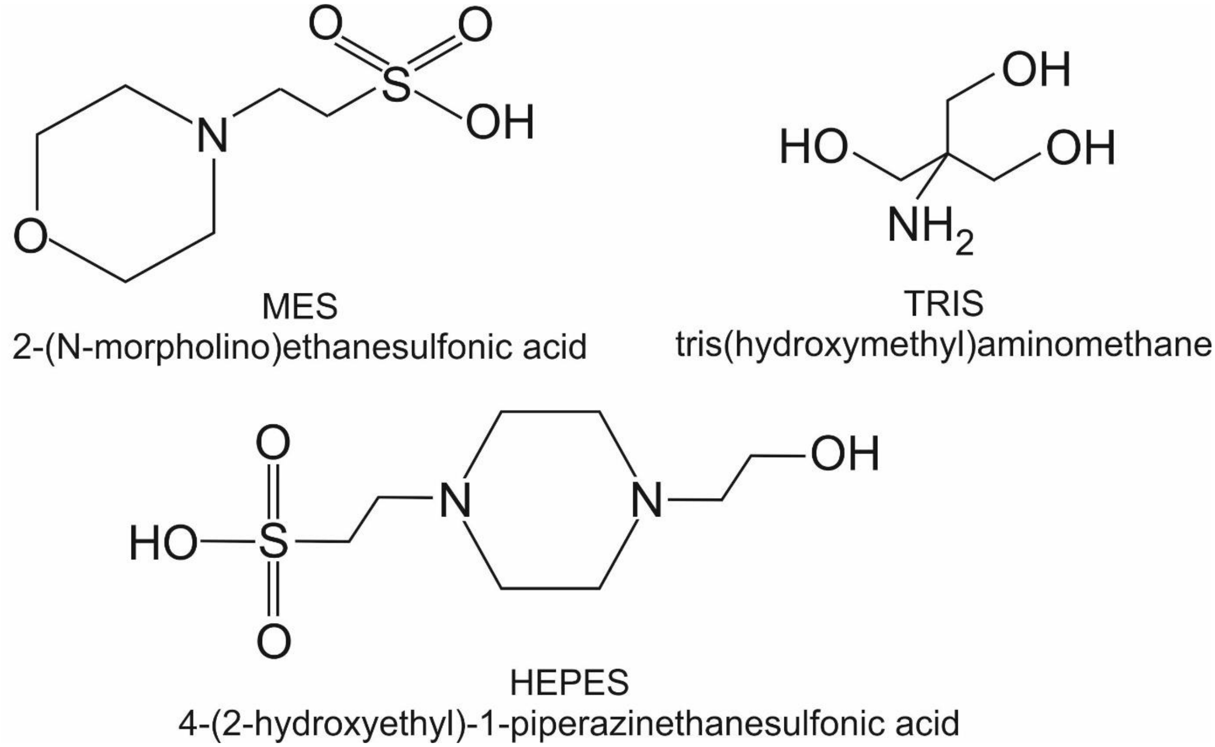

Fig. S2. Buffer components.

Structural formulas of organic buffers components.

2. Synthesis and characterization of polyoxometalates

$K_6[\alpha\text{-}P^{V}_2W^{VI}_{18}O_{62}]\cdot19H_2O$ (**α-P$_2$W$_{18}$**), (*12*) $(NH_4)_6[\alpha/\beta\text{-}P^{V}_2W^{VI}_{18}O_{62}]\cdot14H_2O$ (**α/β-P$_2$W$_{18}$**), (*13*) $(NH_4)_6[\alpha\text{-}P^{V}_2Mo^{VI}_{18}O_{62}]\cdot19H_2O$ (**P$_2$Mo$_{18}$**), (*17*) $K_{12.5}Na_{1.5}[NaP^{V}_5W^{VI}_{30}O_{114}]\cdot19H_2O$ (**P$_5$W$_{30}$**), (*14*) $K_4[\alpha\text{-}Si^{IV}W^{VI}_{12}O_{40}]\cdot8H_2O$ (**SiW$_{12}$**), (*19*) $(Et_2NH_2)_8[\{\alpha\text{-}P^{V}W^{VI}_{11}O_{39}Zr^{IV}(\mu\text{-}OH)(H_2O)\}_2]\cdot7H_2O$ (Et – ethyl, **(ZrPW$_{11}$)$_2$**), (*15*) $Na_2K_4[V^{V}_{10}O_{28}]\cdot10H_2O$ (**V$_{10}$**), (*20*) $Na_6[Te^{VI}W^{VI}_6O_{24}]\cdot10H_2O$ (**TeW$_6$**) (*18*) were synthesized according to reported procedures. $Na_3[\alpha\text{-}P^{V}W^{VI}_{12}O_{40}]\cdot8.5H_2O$ (CAS-Number: 312696-30-3; **PW$_{12}$**) and $H_3[\alpha\text{-}P^{V}Mo^{VI}_{12}O_{40}]\cdot9H_2O$ (CAS-Number: 51429-74-4; **PMo$_{12}$**) were purchased from Sigma-Aldrich, as the commercial versions are commonly used in applications. All compounds were characterized in solution by NMR spectroscopy (Sections 6.2, 7, 8.2, 8.3, 9.2, 10.2, 11.2, 12.2, 13.2, 14) and in the solid-state using infrared (IR, Section 3.1) spectroscopy and thermogravimetric analysis (TGA, Section 3.2).

2.1. IR spectroscopy

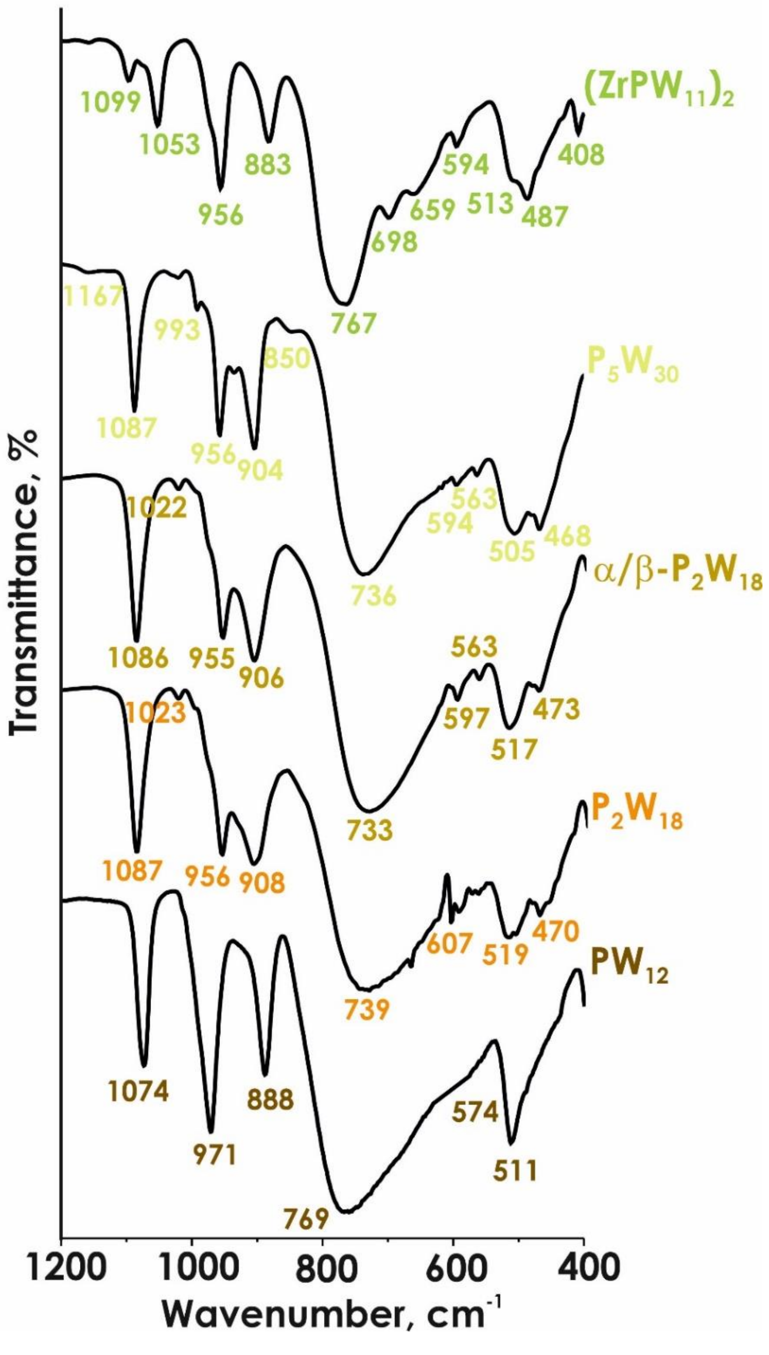

Fig. S3. IR spectra of phosphotungstates.

IR-spectra of five phosphotungstates – $Na_3[\alpha\text{-}P^VW^{VI}_{12}O_{40}]\cdot 8.5H_2O$ (**PW12**), $K_6[\alpha\text{-}P^V_2W^{VI}_{18}O_{62}]\cdot 19H_2O$ (**P2W18**), $(NH_4)_6[\alpha/\beta\text{-}P^V_2W^{VI}_{18}O_{62}]\cdot 14H_2O$ (***α/β*-P2W18**), $K_{12.5}Na_{1.5}[NaP^V_5W^{VI}_{30}O_{114}]\cdot 19H_2O$ (**P5W30**) and $(Et_2NH_2)_8[\{\alpha\text{-}P^VW^{VI}_{11}O_{39}Zr^{IV}(\mu\text{-}OH)(H_2O)\}_2]\cdot 7H_2O$ (**(ZrPW11)2**) – in the fingerprint range of 1200 – 400 cm^{-1} (**Table S1**).

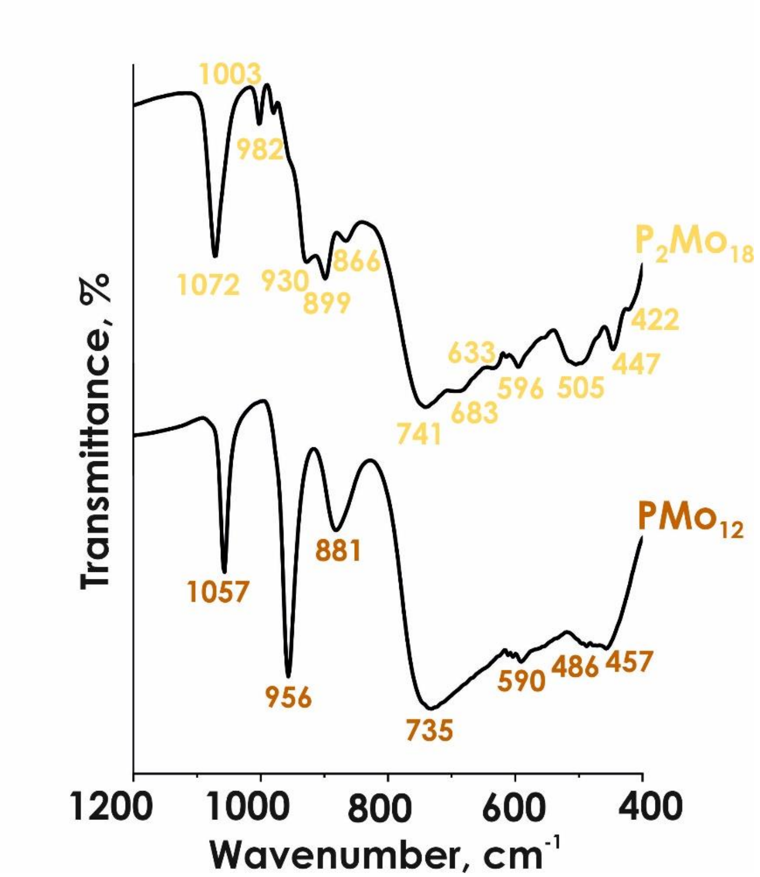

Fig. S4. IR spectra of phosphomolybdates.

IR-spectra of two phosphomolybdates (POMos) – $H_3[\alpha\text{-}P^{V}Mo^{VI}_{12}O_{40}]\cdot 9H_2O$ (**PMo_{12}**) and $(NH_4)_6[\alpha\text{-}P^{V}_2Mo^{VI}_{18}O_{62}]\cdot 19H_2O$ (**P_2Mo_{18}**) – in the fingerprint range of 1200 – 400 cm^{-1} (**Table S1**).

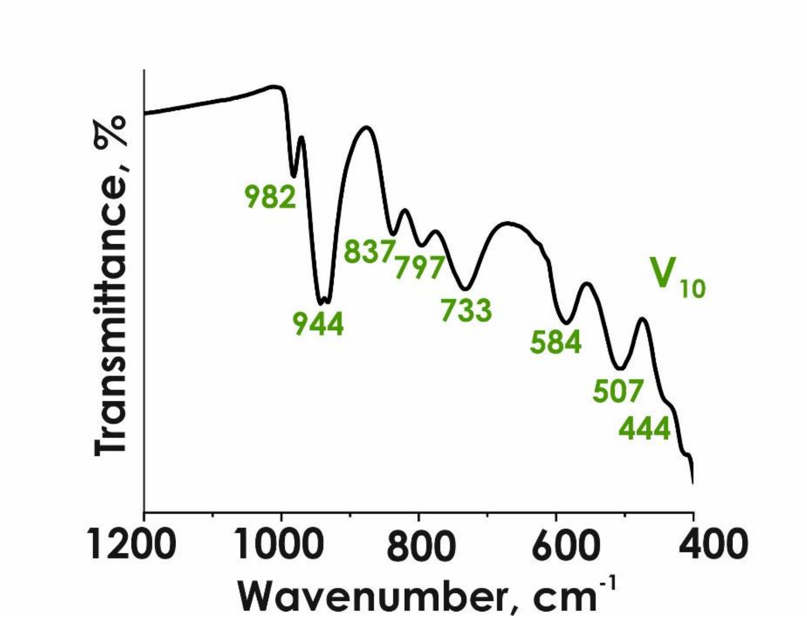

Fig. S5. IR spectrum of decavanadate.
IR-spectrum of $Na_2K_4[V^V_{10}O_{28}]\cdot 10H_2O$ (**V_{10}**) in the fingerprint range of 1200 – 400 cm^{-1} (**Table S1**).

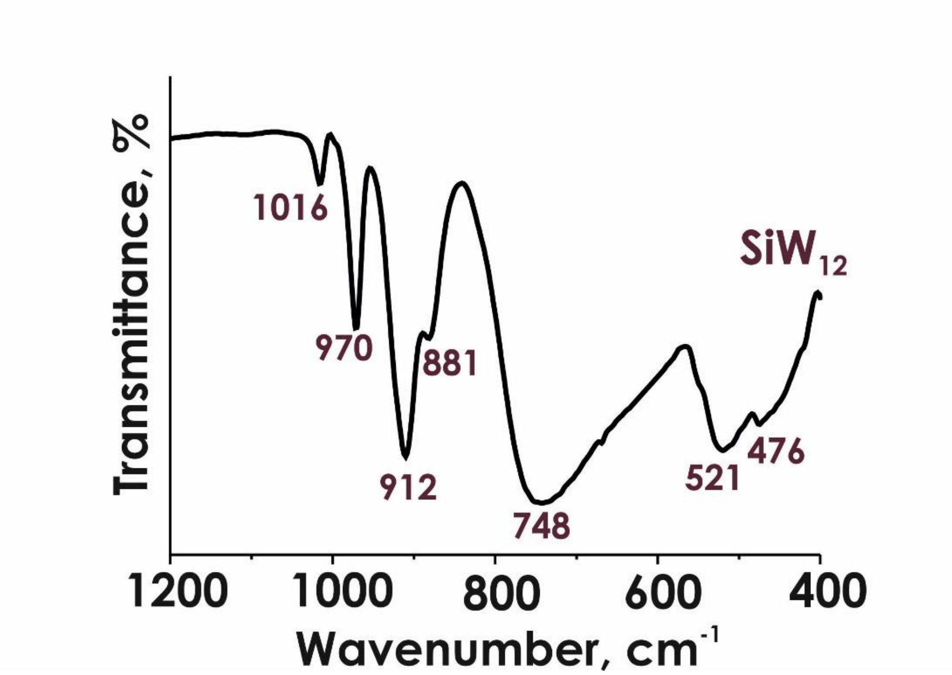

Fig. S6. IR spectrum of Keggin silicotungstate.

IR-spectrum of $K_4[Si^{IV}W^{VI}_{12}O_{40}]\cdot 8H_2O$ (**SiW$_{12}$**) in the fingerprint range of 1200 – 400 cm^{-1} (**Table S1**).

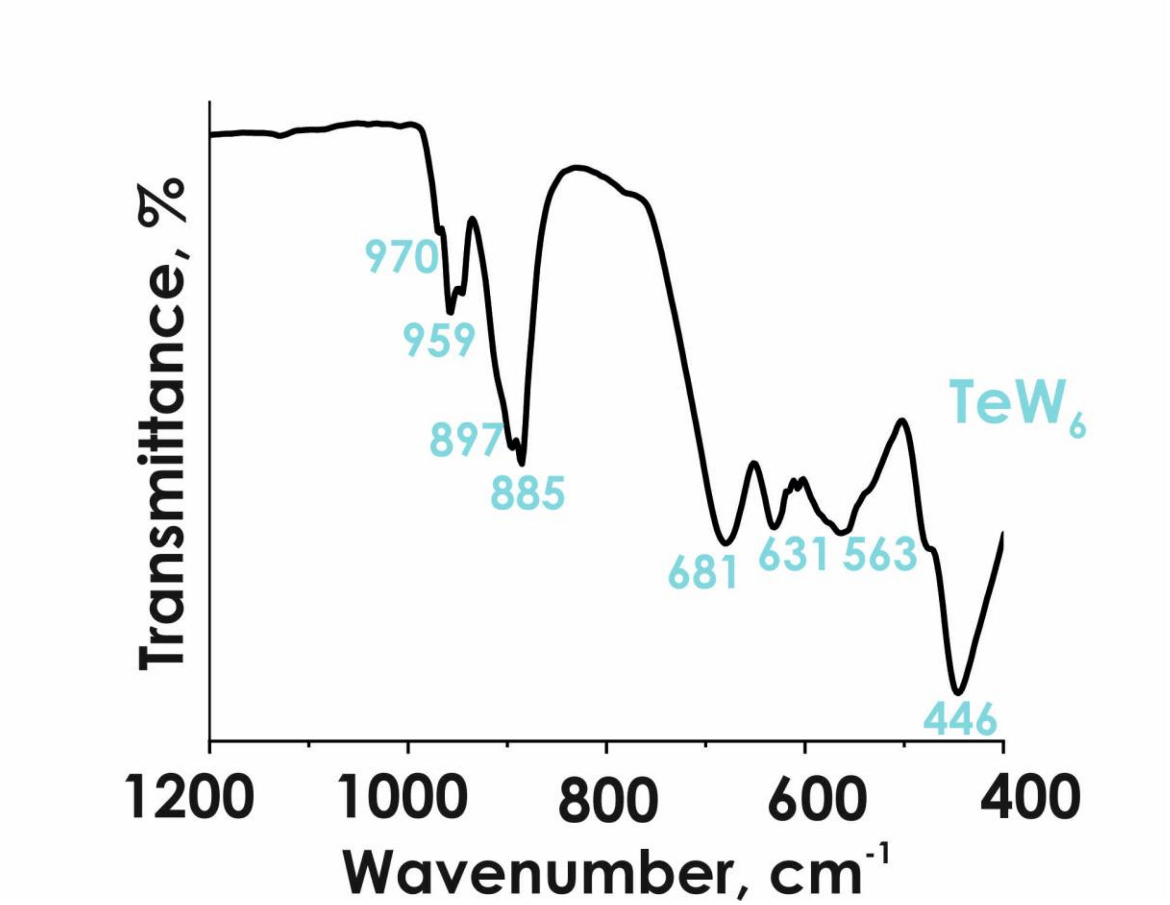

Fig. S7. IR spectrum of Anderson POT TeW_6.

IR-spectrum of $Na_6[Te^{VI}W^{VI}_6O_{24}]\cdot 10H_2O$ (**TeW_6**) in the fingerprint range of 1200 - 400 cm^{-1} (**Table S1**).

Table S1. Positions and attribution of the bands observed in the IR-spectra fingerprint region (1200 – 400 cm^{-1}) of all POMs tested.

Band inconsistencies in some areas of the spectrum may be caused by measurement conditions (in KBr or using ATR-module), different hydrate forms, or not indicating all the bands in the spectrum.

POM	Position and intensity of bands [cm^{-1}]	Position and intensity (if indicated) of bands [cm^{-1}] according to literature	Reference	Attribution
$Na_3[\alpha\text{-}P^VW^{VI}_{12}O_{40}]\cdot 8.5H_2O$	1074 (s)	1080	(*58*)	$^{\nu}$P-O
	971 (s)	987		$^{\nu}$W=O
	888 (s), 769 (s, br), 574 (w, sh), 511 (s)	893, 798, 596, 526		$^{\nu}$W=O, $^{\delta}$W-O-W
$K_6[\alpha\text{-}P^V_2W^{VI}_{18}O_{62}]\cdot 19H_2O$	1087 (s), 1023 (w)	1091, 1020	(*12*)	$^{\nu}$P-O
	956 (s)	961		$^{\nu}$W=O
	908 (s), 739 (s, br), 607 (m), 519 (m), 470 (m)	913, 783, 598, 565, 528, 475		$^{\nu}$W=O, $^{\delta}$W-O-W
$(NH_4)_6[\alpha/\beta\text{-}P^V_2W^{VI}_{18}O_{62}]\cdot 14H_2O$	1086 (s) 1022 (w)	1091, 1020	(*12*)	$^{\nu}$P-O
	955 (s)	961		$^{\nu}$W=O
	906 (s), 733 (s, br), 597 (w), 563 (w), 517 (m), 473 (m)	913, 783, 598, 565, 528, 475		$^{\nu}$W=O, $^{\delta}$W-O-W
$K_{12.5}Na_{1.5}[NaP^V_5W^{VI}_{30}O_{114}]\cdot 19H_2O$	1167 (w), 1087 (s)	1157 (s), 1075 (m)	(14)	$^{\nu}$P-O
	956 (s)	1016 (w)		$^{\nu}$W=O
	904 (s), 850 (w), 736 (s. br), 594 (w), 563 (w), 505 (m), 468 (m)	904 (s), 704 (s)		$^{\nu}$W=O, $^{\delta}$W-O-W
$(Et_2NH_2)_8[\{\alpha\text{-}P^VW^{VI}_{11}O_{39}Zr^{IV}(\mu\text{-}OH)(H_2O)\}_2]\cdot 7H_2O$	1099 (w), 1053 (m)	1098 (m), 1057 (s)	(*59*)	$^{\nu}$P-O
	956 (s)	960 (s)		$^{\nu}$W=O
	883 (m), 767 (s, br), 698 (m, sh), 659 (w), 594 (w), 513 (m, sh), 487 (m), 408 (s)	889 (s), 810 (s), 712 (s), 670 (m), 596 (m), 514 (m)		$^{\nu}$W=O, $^{\delta}$W-O-W
$H_3[\alpha\text{-}P^VMo^{VI}_{12}O_{40}]\cdot 9H_2O$	1057 (s)	1063	(58)	$^{\nu}$P-O
	956 (s)	965, 955		$^{\nu}$Mo=O

	881 (s), 735 (s, br), 590 (w), 486 (w), 457 (w)	880, 805		$^{\nu}$Mo=O, $^{\delta}$Mo-O-Mo
$(NH_4)_6[\alpha\text{-}P^{V}_2Mo^{VI}_{18}O_{62}]\cdot 19H_2O$	1072 (s), 1003 (w)	1077 (s)	(60)	$^{\nu}$P-O
	930 (s)	940 (s)		$^{\nu}$Mo=O
	899 (s), 866 (m), 741 (s, br), 683 (w), 633 (w), 596 (w), 505 (w), 447 (m), 422 (w)	905 (s), 880 (w), 823 (s), 778 (s)		$^{\nu}$Mo=O, $^{\delta}$Mo-O-Mo
$Na_2K_4[V^{V}_{10}O_{28}]\cdot 10H_2O$	982 (m), 944 (s)	966 (s), 941 (s)	(*61*)	$^{\nu}$V=O
	837 (m), 797 (m), 733 (m), 584 (s), 507 (s), 444 (w, sh)	821 (s), 755 (m), 594 (m), 523 (m), 411 (w)		$^{\nu}$V=O, $^{\delta}$V-O-V
$K_4[Si^{IV}W^{VI}_{12}O_{40}]\cdot 8H_2O$	1016 (w)	1020, 999	(58)	$^{\nu}$Si-O
	970 (s)	980, 940		$^{\nu}$W=O
	912 (s), 881 (s, sh), 748 (s, br), 521 (s), 476 (s, sh)	925, 894, 878, 780, 550, 530, 474, 373		$^{\nu}$W=O, $^{\delta}$W-O-W
$Na_6[Te^{VI}W^{VI}_6O_{24}]\cdot 10H_2O$	970 (w, sh), 959 (m)	982	(62) / (63)	$^{\nu}$W=O
	681 (s)	in the range 670 – 700 / 668		$^{\nu}$Te-O
	897 (s, sh), 885 (s), 681 (s), 631 (m) 563 (m), 446 (s)	in the range 350 – 995 / 878, 656		$^{\nu}$W=O, $^{\delta}$W-O-W, $^{\nu}$Te-O

2.2. Thermogravimetric analysis

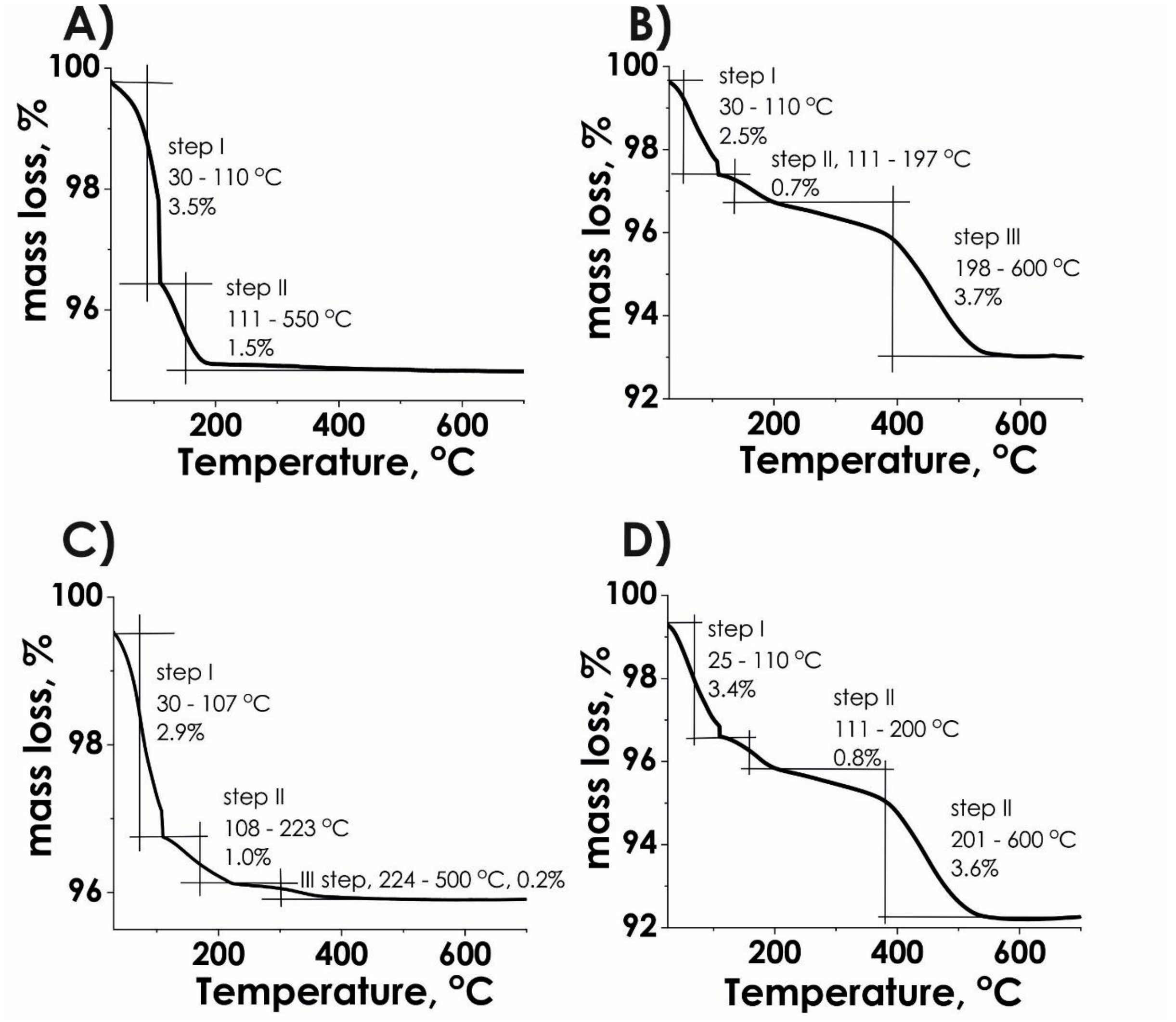

Fig. S8. TGA of phosphotungsatates.

Thermogravimetric curve of phosphotungsatates: **A)** $Na_3[\alpha\text{-}P^{V}W^{VI}_{12}O_{40}]\cdot 8.5H_2O$; **B)** $K_6[\alpha\text{-}P^{V}_2W^{VI}_{18}O_{62}]\cdot 19H_2O$; **C)** $K_{12.5}Na_{1.5}[NaP^{V}_5W^{VI}_{30}O_{114}]\cdot 19H_2O$; **D)** $(NH_4)_6[\alpha/\beta\text{-}P^{V}_2W^{VI}_{18}O_{62}]\cdot 14H_2O$, in the temperature region 30 – 700 ºC with a heating rate of 5 ºC min^{-1}. The sample was equilibrated at 30 ºC and kept isothermally for 10 min at 110 ºC and 1 min at 700 ºC. See **Table S2** for details on weight loss.

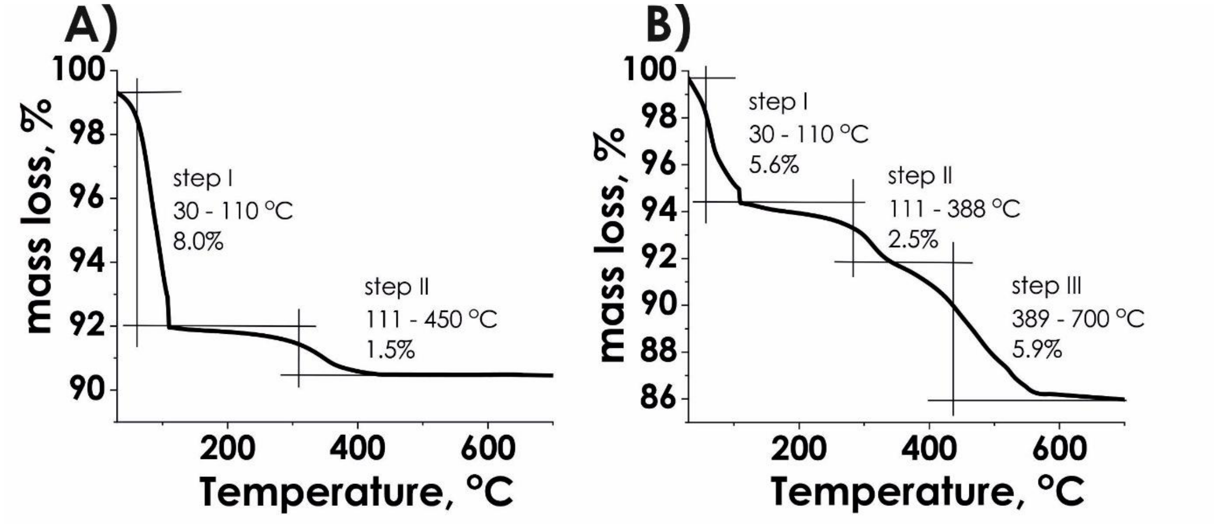

Fig. S9. TGA of phosphomolybdates.

Thermogravimetric curve of phosphomolybdates: **A)** $H_3[\alpha\text{-}P^{V}Mo^{VI}_{12}O_{40}]\cdot 9H_2O$; **B)** $(NH_4)_6[\alpha\text{-}P^{V}_2Mo^{VI}_{18}O_{62}]\cdot 19H_2O$, in the temperature region 30 – 700 ºC with a heating rate of 5 ºC min^{-1}. The sample was equilibrated at 30 ºC and kept isothermally for 10 min at 110 ºC and 1 min at 700 ºC. See **Table S2** for details on weight loss.

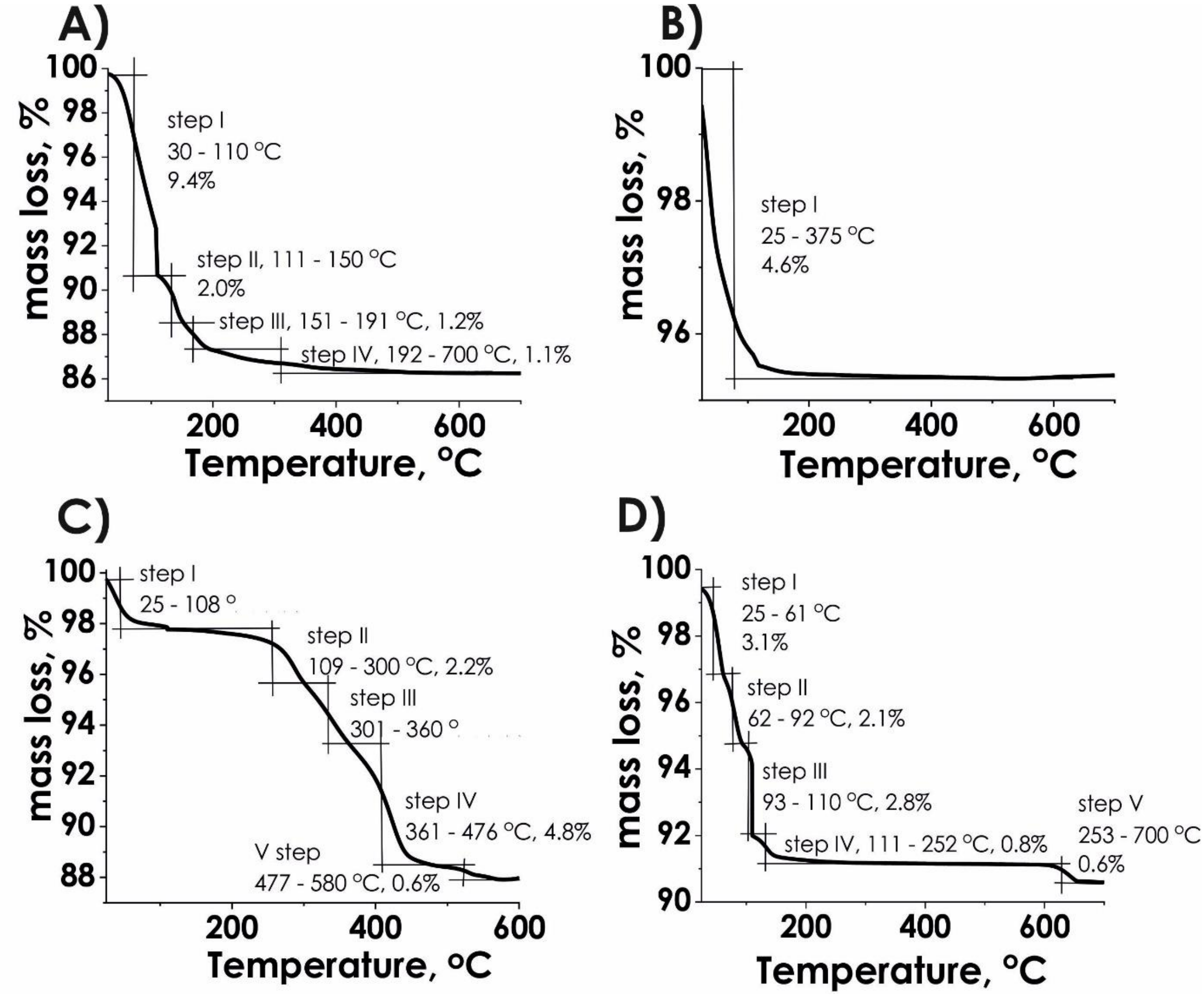

Fig. S10. TGA of decavandate, SiW_{12}, $(ZrPW_{11})_2$ and TeW_6.

Thermogravimetric curve: **A)** $Na_2K_4[V^{V}_{10}O_{28}]\cdot 10H_2O$; **B)** $K_4[Si^{IV}W^{VI}_{12}O_{40}]\cdot 8H_2O$, **C)** $(Et_2NH_2)_8[\{\alpha\text{-}P^{V}W^{VI}_{11}O_{39}Zr^{IV}(\mu\text{-}OH)(H_2O)\}_2]\cdot 7H_2O$; **D)** $Na_6[Te^{VI}W^{VI}_6O_{24}]\cdot 10H_2O$ in the temperature region 30 – 700 ºC with a heating rate of 5 ºC min^{-1}. The sample was equilibrated at 30 ºC and kept isothermally for 10 min at 110 ºC and 1 min at 700 ºC. See **Table S2** for details on weight loss.

Table S2. TGA analysis.

The mass-loss steps with a corresponding assignment to the solvent molecules and /or counter-cations for all POMs tested; n.r. – not reported.

POM	TGA curve displayed in Figure	Step	T, °C	mass-loss, %	Number and kind of molecules corresponding to mass-loss	Number of water molecules reported in the literature
$Na_3[\alpha\text{-}P^{V}W^{VI}_{12}O_{40}]\cdot 8.5H_2O$	S8A	I	30 – 110	3.5	6 H_2O	n.r. (commercial compound)
		II	111 – 500	1.5	2.5 H_2O	
$K_6[P_2W_{18}O_{62}]\cdot 19H_2O$	S8B	I	30 – 110	2.5	7 H_2O	14 H_2O
		II	111 – 197	0.7	2 H_2O	
		III	198 – 600	3.7	10 H_2O	
$K_{12.5}Na_{1.5}[NaP^{V}_5W^{VI}_{30}O_{114}]\cdot 19H_2O$	S8C	I	30 – 107	2.9	13.5 H_2O	15 H_2O
		II	108 – 223	1.0	4.5 H_2O	
		III	224 – 500	0.2	1 H_2O	
$(NH_4)_6[\alpha/\beta\text{-}P^{V}_2W^{VI}_{18}O_{62}]\cdot 14H_2O$	S8D	I	25 – 110	3.4	9 H_2O	14 H_2O + 6 NH_4^+
		II	111 – 200	0.8	2 H_2O	
		III	201 – 600	3.6	3 H_2O + 6 NH_4^+	
$H_3[\alpha\text{-}P^{V}Mo^{VI}_{12}O_{40}]\cdot 9H_2O$	S9A	I	30 – 110	8.0	8.5 H_2O	n.r. (commercial compound)
		II	111 – 450	1.5	2 H_2O	

$(NH_4)_6[\alpha\text{-}P^{V}_2Mo^{VI}_{18}O_{62}]\cdot 19H_2O$	S9B	I	30 – 110	5.6	10 H_2O	14 H_2O + 6 NH_4^+
		II	111 – 388	2.5	4.5 H_2O	
		III	389 – 700	5.9	4.5 H_2O + 6 NH_4	
$Na_2K_4[V^{V}_{10}O_{28}]\cdot 10H_2O$	S10A	I	30 – 110	9.4	7 H_2O	10 H_2O
		II	111 – 150	2.0	1.5 H_2O	
		III	151 – 191	1.2	0.8 H_2O	
		IV	192 – 700	1.1	0.7 H_2O	
$K_4[SiW_{12}O_{40}]\cdot 8H_2O$	S10B	I	25 – 375	4.6	8 H_2O	9 H_2O
$(Et_2NH_2)_8[\{\alpha\text{-}P^{V}W^{VI}_{11}O_{39}Zr^{IV}(\mu\text{-}OH)(H_2O)\}_2]\cdot 7H_2O$	S10C	I	30 – 108	2.1	7 H_2O	7 H_2O
		II	109 – 300	2.2	2 $Et_2NH_2^+$	8 $Et_2NH_2^+$
		III	301 – 360	2.4	2 $Et_2NH_2^+$	
		IV	361 – 476	4.8	4 $Et_2NH_2^+$	
		V	477 – 580	0.6	2 H_2O	2 H_2O
$Na_6[Te^{VI}W^{VI}_6O_{24}]\cdot 10H_2O$	S10D	I	25 – 61	3.1	3 H_2O	22 H_2O
		II	62 – 92	2.1	2 H_2O	
		III	93 – 110	2.8	3 H_2O	

		IV	111 – 252	0.8	H_2O	
		V	253 – 700	0.6	H_2O	

3. Application of POMs in aqueous solutions

Table S3. Applications of POMs in aqueous solutions.

Application of the 10 POMs investigated in aqueous solutions. The search was conducted using the Scopus document search engine and was limited to articles published from 2012 up to 2022. When POM is applied under identical conditions in multiple papers, the table only shows one representative example; n.r. – not reported.

POM formula	Type of application	System / Reaction	Solvent	Stability check	Species detected in solution	Reference
**PW_{12}**						
$H_3[P^VW^{VI}_{12}O_{40}]$	catalysis	hydration of diphenylacetylene	1,4-dioxane:H_2O (4 : 1)	no	n.r.	(64)
$H_3[P^VW^{VI}_{12}O_{40}]$	catalysis	direct conversion of cellulose to glycolic acid	H_2O (at 180°C under 0.6 MPa in autoclave)	no	n.r.	(*65*)
$H_3[P^VW^{VI}_{12}O_{40}]$	catalysis	hydrolysis of cellulose to glucose	H_2O (microwave aided, 400 W)	no	n.r.	(*66*)
$H_3[P^VW^{VI}_{12}O_{40}]$	catalysis	conversion of chitin to 5-hydroxymethylfurfural	DMSO/H_2O	no	n.r.	(*67*)
$H_3[P^VW^{VI}_{12}O_{40}]$	catalysis	removal of SO_2 and NO_x	H_2O	no	n.r.	(*68*)
$H_3[P^VW^{VI}_{12}O_{40}]$	catalysis	conversion of biomass-derived levulinic acid to γ-valerolactone	H_2O for preparation of the catalyst	no	n.r.	(*69*)
(FPDS)$_3[P^VW^{VI}_{12}O_{40}]$, FPDS = (4-formylphenyl)dimethylsulfonium	catalysis	photocatalytic activity toward	H_2O acidified to pH 2.5	no	n.r.	(*70*)

		dichromate reduction				
$(NH_4)_3[P^VW^{VI}_{12}O_{40}]$	catalysis	water purification	solution of Na_2SO_4 (0.1 mol/L)	no	n.r.	(*71*)
$H_3[P^VW^{VI}_{12}O_{40}]$	catalysis	peroxidase mimetics	buffer solution (not specified which one), pH 3.0	no	n.r.	(*72*)
$H_3[P^VW^{VI}_{12}O_{40}]$	catalysis	peroxidase mimetics (POM is combined with glucose oxidase (GOx) generating microsphere structures)	PBS buffer (20 µL, pH 7.4)	no	n.r.	(*73*)
$H_3[P^VW^{VI}_{12}O_{40}]$	catalysis	removal of sialic acid from a glycoprotein	H_2O acidified to pH 2.8	yes, ^{31}P NMR spectrosc opy	mixture of $[P^VW^{VI}_{12}O_{40}]^{3-}$ and $[P^V_2W^{VI}_{20}O_{70}]^{10-}$, ratio is not reperted.	(*74*)
$H_3[P^VW^{VI}_{12}O_{40}]$	biology	potential preventative and therapeutic drug for inflammatory bowel disease	H_2O; PBS buffer pH 7.4; RMPI (Roswell Park Memorial Institute) 1640 medium [10% fetal bovine serum (FBS)], lipid (10% cooking oil aqueous solution with 1% Tween 80), different solution	no	n.r	(*75*)

			temperatures (4, 25 and 37 °C) and pH value (8.0, 7.4, 6.0 and 1.5) with incubation time 4 h			
$H_3[P^VW^{VI}_{12}O_{40}]$	biology	antibacterial activity against *Moraxella catarrhalis*	Mueller–Hinton broth (MHB), pH 7.4	no	n.r.	(*46*)
$(NH_4)_3[P^VW^{VI}_{12}O_{40}]$	biology	antibiotic adsorption	H_2O	no	n.r.	(*76*)
$H_3[P^VW^{VI}_{12}O_{40}]$	biology	anti-cancer effects on glioblastoma cells	HEPES pH 7; potassium phosphate buffer pH 7	yes, UV-Vis spectroscopy	n.r.	(*77*)
**PMo_{12}**						
$H_3[P^VMo^{VI}_{12}O_{40}]$	catalysis	direct conversion of cellulose to glycolic acid	H_2O (at 180°C under 0.6 MPa in autoclave)	no	n.r.	(*65*)
$H_3[P^VMo^{VI}_{12}O_{40}]$	catalysis	conversion of chitin to 5-hydroxymethylfurfural	DMSO/H_2O	no	n.r.	(*67*)
$H_3[P^VMo^{VI}_{12}O_{40}]$	catalysis	removal of SO_2 and NO_x	H_2O	no	n.r.	(*68*)
$(FPDS)_3[P^VMo^{VI}_{12}O_{40}]$, FPDS = (4-formylphenyl)dimethylsulfonium	catalysis	photocatalytic activity toward dichromate reduction	H_2O acidified to pH 2.5	no	n.r.	(*70*)
$H_3[P^VMo^{VI}_{12}O_{40}]$	catalysis	peroxidase mimetics (POM is combined with	PBS buffer (pH 7.4)	no	n.r.	(*73*)

		glucose oxidase (GOx) generating microsphere structures)				
$H_3[P^VMo^{VI}_{12}O_{40}]$	biology	enhancing short-chain fatty acids (SCFAs) production from waste-activated sludge	waste activated sludge	no	n.r.	(*78*)
$H_3[P^VMo^{VI}_{12}O_{40}]$	biology	enhancing methane production from waste-activated sludge	waste activated sludge	no	n.r.	(*79*)
$H_3[P^VMo^{VI}_{12}O_{40}]$	biology	inhibition of α-glucosidase	0.1 M phosphate buffer (Na_2HPO_4-NaH_2PO_4 buffer, pH 6.8) + DMSO + α-glucosidase	no	n.r.	(*80*)
$[Ln^{III}_3(PDA)_3(H_2O)_6(P^VMo^{VI}_{12}O_{40})]$, Ln = La; Ce; Pr; Nd; PDA = 1,10-phenanthroline-2,9-dicarboxylate)	biology	opioid drug detection	H_2O, pH 6	no	n.r.	(*81*)
$(NH_4)_3[P^VMo^{VI}_{12}O_{40}]$	biology	antibiotic adsorption	H_2O	no	n.r.	(*76*)
**P_2W_{18}**						
$H_6[P^V_2W^{VI}_{18}O_{62}]$	catalysis	hydration of diphenylacetylen e	1,4-dioxane : H_2O (4 : 1)	yes	n.r.	(*64*)
$H_6[P^V_2W^{VI}_{18}O_{62}]$	catalysis	hydrolysis of cellulose to glucose	H_2O (microwave aided, 400 W)	no	n.r.	(*66*)

$K_6[\alpha/\beta\text{-}P^{V}_2W^{VI}_{18}O_{62}]$	catalysis	hydrolysis of cellulose to glucose	H_2O (microwave aided, 400 W)	no	n.r.	(*66*)
$K_6[\alpha/\beta\text{-}P^{V}_2W^{VI}_{18}O_{62}]$	catalysis	removal of sialic acid from a glycoprotein	H_2O acidified to pH 2.8	yes, ^{31}P NMR spectroscopy	$[\alpha/\beta\text{-}P^{V}_2W^{VI}_{18}O_{62}]^{6-}$	(*74*)
$K_6[\alpha/\beta\text{-}P^{V}_2W^{VI}_{18}O_{62}]$	biology	anti-quorum sensing, antibiofilm, and antiviral activities	H_2O; Mueller–Hinton broth (MHB), pH 7.4	yes, ^{31}P NMR spectroscopy in MHB	mixture of $[\alpha/\beta\text{-}P^{V}_2W^{VI}_{18}O_{62}]^{6-}$ and $[P^{V}_2W^{VI}_{17}O_{61}]^{10-}$, ratio is not reperted.	(*82*)
$K_6[\alpha/\beta\text{-}P^{V}_2W^{VI}_{18}O_{62}]$	biology	antibacterial activity against *Moraxella catarrhalis*	Mueller–Hinton broth (MHB), pH 7.4	no	n.r.	(*46*)
$K_6[\alpha/\beta\text{-}P^{V}_2W^{VI}_{18}O_{62}]$	biology	inhibition of aquaporin-3 activity, anti-cancer activity against human melanoma	a stock solution of POM was prepared in H_2O; cell line in PBS (pH 7.4)	yes, ^{31}P NMR spectroscopy in H_2O and PBS pH 7.4	mixture of $[\alpha/\beta\text{-}P^{V}_2W^{VI}_{18}O_{62}]^{6-}$ and $[P^{V}_2W^{VI}_{17}O_{61}]^{10-}$, ratio is not reperted.	(*83*)
$Li_6[P^{V}_2W^{VI}_{18}O_{62}]$	energy	electron storage	H_2O	yes, CV and UV-Vis	$[\alpha\text{-}P^{V}_2W^{VI}_{18}O_{62}]^{6-}$	(*84*)
**P_2Mo_{18}**						
$H_6[P^{V}_2Mo^{VI}_{18}O_{62}]$	catalysis	conversion of glucose into glycolic acid	H_2O under hydrothermal reaction	no	n.r.	(*85*)

$K_6[P^V_2Mo^{VI}_{18}O_{62}]$	biology	antibacterial activity against *Moraxella catarrhalis*	Mueller–Hinton broth (MHB), pH 7.4	no	n.r.	(*46*)
$K_6[P^V_2Mo^{VI}_{18}O_{62}]$	biology	antibacterial activity against *Escherichia coli* and *Staphylococcus aureus*	films preparation in H_2O at 80 ºC; antibac. tests in non-specified culture medium with 24 h at 37 °C	no	n.r.	(*86*)
$K_6[P^V_2Mo^{VI}_{18}O_{62}]$	biology	inhibition of α-glucosidase	0.1 M phosphate buffer (Na_2HPO_4-NaH_2PO_4 buffer, pH 6.8) + DMSO + α-glucosidase	no	n.r.	(*87*)
$K_6[P^V_2Mo^{VI}_{18}O_{62}]$	biology	hydration of diphenylacetylene	1,4-dioxane : H_2O (4 : 1)	yes	n.r.	(*64*)
**P_5W_{30}**						
$K_{13}Na[NaP^V_5W^{VI}_{30}O_{110}]$	biology	antibiotic resistance	lysogeny broth (LB, peptone 140, yeast extract, NaCl) at 37°C, Mueller–Hinton broth (MHB), pH 7.4	yes, ^{31}P NMR in tris buffer (0.05 M, pH 7, 25 °C)	$[NaP^V_5W^{VI}_{30}O_{110}]^{14-}$	(*88*)
$(NH_4)_{14}[NaP^V_5W^{VI}_{30}O_{110}]$	biology	Ca^{2+}-ATPase activities	H_2O; 25 mM HEPES (pH 7.0), 100 mM KCl, 5 mM $MgCl_2$, 50 µM $CaCl_2$	yes, ^{31}P NMR spectroscopy in HEPES (pH 7.0) after	$[NaP^V_5W^{VI}_{30}O_{110}]^{14-}$	(*89*)

				incubation for 30 min at 37 °C		
$(NH_4)_{14}[NaP^{V}_5W^{VI}_{30}O_{110}]$	biology	anti-quorum sensing, antibiofilm, and antiviral activities	H_2O; Mueller–Hinton broth (MHB), pH 7.4	yes, ^{31}P NMR spectroscopy in MHB	$[NaP^{V}_5W^{VI}_{30}O_{110}]^{14-}$	(*82*)
$[NaP_5W_{30}O_{110}]^{14-}$ (counter cations are not specified)	biology	inhibition of amyloid-beta protein fibrillization	PBS (pH 7.4, 50 mM phosphate, 0.15 M NaCl)	yes, by UV-vis in PBS pH 7.4	n.r.	(*90*)
$(NH_4)_{14}[NaP^{V}_5W^{VI}_{30}O_{110}]$	biology	antibacterial activity against *Moraxella catarrhalis*	Mueller–Hinton broth (MHB), pH 7.4	no	n.r.	(*46*)
$K_{11}Na_3[NaP^{V}_5W^{VI}_{30}O_{110}]$	biology	antiproliferation of two human cell lines A431 and HEK29, antibacterial activity against *Escherichia coli* and *Bacillus subtilis*	H_2O, Dulbecco's Modified Eagle Medium, (LB, peptone 140, yest extract, NaCl)	yes, ^{31}P NMR spectroscopy only in H_2O	$[NaP^{V}_5W^{VI}_{30}O_{110}]^{14-}$ in H_2O	(*91*)
$(NH_4)_{14}[NaP^{V}_5W^{VI}_{30}O_{110}]$	biology	inhibition of aquaporin-3 activity, anti-cancer activity against human melanoma	a stock solution of POM was prepared in H_2O; cell line in PBS (pH 7.4)	yes, ^{31}P NMR spectroscopy in H_2O and PBS pH 7.4	$[NaP^{V}_5W^{VI}_{30}O_{110}]^{14-}$	(*83*)

$H_{14}[NaP^{V}_{5}W^{VI}_{30}O_{110}]$	biology	*in vitro* inhibitory effects on HepG2 tumor cells	POM was dissolved in NaCl aqueous solution, and then mixed with liposomes; cell experiments were performed in Dulbecco's Modified Eagle Medium	no	n.r.	(*92*)
$K_{12}Na_{2}[NaP^{V}_{5}W^{VI}_{30}O_{110}]$	biology	antibacterial activity against *Escherichia coli* and *Staphylococcus aureus*	Mueller–Hinton broth (MHB), pH 7.4, incubation for 24h at 37 ºC	yes, ^{31}P NMR spectroscopy only in H_2O	$[NaP^{V}_{5}W^{VI}_{30}O_{110}]^{14-}$ in H_2O	(*93*)
$K_{13}Na[NaP^{V}_{5}W^{VI}_{30}O_{110}]$	biology	modulation of bacterial hyperpolarization (*Bacillus subtilis*)	lysogeny broth (LB, peptone 140, yest extract, NaCl) with incubation at 37 ºC	yes, ^{31}P NMR spectroscopy in tris -HCl pH 7 at 25 ºC	$[NaP^{V}_{5}W^{VI}_{30}O_{110}]^{14-}$ in tris-HCl	(*88*)
**SiW_{12}**						
$H_4[Si^{IV}W^{VI}_{12}O_{40}]$	catalysis	direct conversion of cellulose to glycolic acid	H_2O (at 180°C under 0.6 MPa in autoclave)	no	n.r.	(*64*)
$H_4[Si^{IV}W^{VI}_{12}O_{40}]$	catalysis	conversion of chitin to 5-hydroxymethylfurfural	DMSO/H_2O	no	n.r.	(*67*)
$H_4[Si^{IV}W^{VI}_{12}O_{40}]$	catalysis	detection of H_2O_2 and glucose	NaOAc buffer solution, pH 4.0	no	n.r.	(*94*)

$H_4[Si^{IV}W^{VI}_{12}O_{40}]$	catalysis	removal of SO_2 and NO_x	H_2O	no	n.r.	(*68*)
$(FPDS)_4[Si^{IV}W^{VI}_{12}O_{40}]$, FPDS = (4-formylphenyl)dimethylsulfonium	catalysis	photocatalytic activity toward dichromate reduction	H_2O acidified to pH 2.5	no	n.r.	(*69*)
**$(ZrPW_{11})_2$**						
$(Et_2NH_2)_8\{[\alpha\text{-}P^{V}W^{VI}_{11}O_{39}Zr^{IV}(\mu\text{–}OH)(H_2O)]_2\}$	catalysis	amide bond hydrolysis in peptides and cyclic peptides	H_2O pH 5.4	yes, ^{31}P NMR spectroscopy	$[(\alpha\text{-}PW_{11}O_{39}Zr(\mu\text{–}OH)(H_2O))_2]^{8-}$	(15)
$(Et_2NH_2)_8\{[\alpha\text{-}P^{V}W^{VI}_{11}O_{39}Zr^{IV}(\mu\text{–}OH)(H_2O)]_2\}$	catalysis /biology	artificial proteases for phosphoester bond hydrolysis in horse heart myoglobin	10 mM acetate buffer pH 5.0; 10 mM phosphate buffer pH 7	yes, ^{31}P NMR spectroscopy	$[(\alpha\text{-}PW_{11}O_{39}Zr(\mu\text{–}OH)(H_2O))_2]^{8-}$ in acetate buffer; mixture (ratio is not reperted.) of $[(\alpha\text{-}PW_{11}O_{39}Zr(\mu\text{–}OH)(H_2O))_2]^{8-}$ and $[Zr(PW_{11}O_{39})_2]^{10-}$ in phosphate	(*95*)
$(Et_2NH_2)_8\{[\alpha\text{-}P^{V}W^{VI}_{11}O_{39}Zr^{IV}(\mu\text{–}OH)(H_2O)]_2\}$	catalysis /biology	artificial proteases for phosphoester bond hydrolysis	H_2O at pH 6.4	yes, ^{31}P NMR spectroscopy	mixture of $[(\alpha\text{-}PW_{11}O_{39}Zr(\mu\text{–}OH)(H_2O))_2]^{8-}$ and $[Zr(PW_{11}O_{39})_2]^{10-}$	(*96*)
$(Et_2NH_2)_8\{[\alpha\text{-}P^{V}W^{VI}_{11}O_{39}Zr^{IV}(\mu\text{–}OH)(H_2O)]_2\}$	catalysis /biology	artificial proteases for phosphoester	10 mM acetate buffer pH 5.0	yes, ^{31}P NMR	$[(\alpha\text{-}PW_{11}O_{39}Zr(\mu\text{–}OH)(H_2O))_2]^{8-}$	(*97*)

		bond hydrolysis in horse heart myoglobin		spectroscopy	at RT; after 1 day at 60 ºC 15 % of it decomposes to $[Zr(PW_{11}O_{39})_2]^{10-}$	
$(Et_2NH_2)_8\{[\alpha\text{-}P^{V}W^{VI}_{11}O_{39}Zr^{IV}(\mu\text{–}OH)(H_2O)]_2\}$	catalysis /biology	hydrolytic cleavage of DNA	tris-HCl buffer at pH 7.0	yes, ^{31}P NMR spectroscopy	mixture of $[(\alpha\text{-}PW_{11}O_{39}Zr(\mu\text{–}OH)(H_2O))_2]^{8-}$ and $[Zr(PW_{11}O_{39})_2]^{10-}$, ratio is not reperted.	(*98*)
$(Et_2NH_2)_8\{[\alpha\text{-}P^{V}W^{VI}_{11}O_{39}Zr^{IV}(\mu\text{–}OH)(H_2O)]_2\}$	catalysis /biology	Chemical mimics of aspartate-directed proteases	10 mM phosphate buffer (pH 7.4)	no, referred to previous papers	n.r.	(*99*)
**TeW_6**						
$Na_6[Te^{VI}W^{VI}_6O_{24}]$	biology	activity against SARS-CoV-2 target proteins	HEPES pH 7.5, 1 h incubation at 37°C	no	n.r.	(*100*)
$Na_6[Te^{VI}W^{VI}_6O_{24}]$	biology	antibacterial activity against *Moraxella catarrhalis*	Mueller–Hinton broth (MHB), pH 7.4	no	n.r.	(*46*)
$Na_6[Te^{VI}W^{VI}_6O_{24}]$	biology	additive for crystallization of PPO4 mushroom (*Agaricus bisporus*) tyrosinase	25 mM tris–HCl pH 7.5, 10% PEG 4000	no	n.r.	(*101*)

$Na_6[Te^{VI}W^{VI}_6O_{24}]$	biology	additive for crystallization of PPO4 mushroom (*Agaricus bisporus*) tyrosinase	25 mM tris–HCl pH 7.5, 10% PEG 4000	no	n.r.	(*102*)
$Na_6[Te^{VI}W^{VI}_6O_{24}]$	biology	additive for crystallization of hen egg-white lysozyme	NaOAc (0.1 M, pH 4.8), NaCl 5–9 %, w/v	no	n.r.	(*103*)
$Na_6[Te^{VI}W^{VI}_6O_{24}]$	biology	additive for crystallization of aurone synthase (AUS) from *Coreopsis grandiflora*	60 mM Na citrate pH 6.4,12% PEG 4000	no	n.r.	(*104*)
$Na_6[Te^{VI}W^{VI}_6O_{24}]$	biology	additive for crystallization of aurone synthase (AUS) from *Coreopsis grandiflora*	60 mM Na citrate pH 6.4,12% PEG 4000	no	n.r.	(*105*)
$Na_6[Te^{VI}W^{VI}_6O_{24}]$	biology	additive for crystallization of HSP70 nucleotide binding domain	20 mM sodium/potassium phosphate, 300 mM potassium chloride, 0.5 mM tris(2-carboxyethyl)phosphine, 1 mM magnesium chloride, pH 7.5	no	n.r.	(*106*)
**V_{10}**						

$(H$*nicotinamide*$)_2\{[Co^{II}(H_2O)_3($*nicotinamide*$)_2]_2[\mu-V^{V}_{10}O_{28}]\}\cdot 6H_2O$	catalysis	water oxidation catalysis	80 mM aqueous borate buffer (pH 7.5) containing 0.2 mM $[Ru^{II}(bpy)_3]Cl_2$ photosensitizer and 2 mM $Na_2S^{VI}_2O_8$	no	n.r.	(*107*)
$Na_2K_4[V^{V}_{10}O_{28}]$	biology	antibacterial activity against *Moraxella catarrhalis*	Mueller–Hinton broth (MHB), pH 7.4	no	n.r.	(*46*)
$(C_4H_7N_2)_6[V^{V}_{10}O_{28}]$	biology	analysis of hepatic enzymes activities, lipid profile and liver histologic examination	PBS, pH 7.4	no	n.r.	(*108*)
$[C_4H_{14}N_5]_3[V^{V}_{10}O_{28}]\cdot 8H_2O$	biology	Ca^{2+}-ATPase activity; antiproliferative effects on melanoma cells	25 mM HEPES (pH 7.0), 100 mM KCl, 5 mM $MgCl_2$, 50 μM $CaCl_2$, 2.5 mM ATP, 0.42 mM phosphoenolpyruvate, 0.25 mM NADH, 18 IU (international unit) lactate dehydrogenase and 7.5 IU pyruvate kinase; Dulbecco's	no	n.r.	(*109*)

			Modified Eagle's Medium			
$(NH_4)_6[V^V{}_{10}O_{28}]$	biology	cytotoxicity of V_{10} with the SARS - COV2 spike protein	Dulbecco's Modified Eagle's Medium	no	n.r.	(*110*)
$(C_7H_{10}N)_4[H_2V^V{}_{10}O_{28}]$	biology	activity against human MDA-MB231 breast cancer cells	Dulbecco's Modified Eagle's Medium incubated for 3 h at 37 °C	no	n.r.	(*111*)
$Na_6[V^V{}_{10}O_{28}]$	biology	antibactetial activity against *Mycobacterium smegmatis*	Middlebrook 7H9 broth medium, pH values were in the range from 5.5 to 6.3	yes, ^{51}V NMR spectroscopy	$[V^V{}_{10}O_{28}]^{6-}$ in the medium; the presence of the mycobacteria catalyzes the hydrolysis of $\mathbf{V_{10}}$	(*40*)

4. Literature known ^{31}P, ^{51}V and ^{183}W NMR shifts for PW_{12}, P_2W_{18}, P_5W_{30}, PMo_{12}, P_2Mo_{18}, $(ZrPW_{11})_2$, SiW_{12}, TeW_6 and V_{10} and their hydrolysis products

Table S4. ^{31}P, ^{51}V, and ^{183}W NMR shifts of POMs tested in this study.

POM	Chemical shifts in aqueous solutions, ppm	Reference
^{31}P		
POTs		
Keggin type *(Fig. S11)*		
α-$[P^{V}W^{VI}_{12}O_{40}]^{3-}$ $[H^+]$	–14.9	(*112*)
α-$[P^{V}W^{VI}_{12}O_{40}]^{4-}$ (1e$^-$ reduced)	–10.4	(*113*)
α-$[P^{V}W^{VI}_{11}O_{39}]^{7-}$ $[Na^+]$	–11.5 or –10.7	(*37*, *114*)
α-$[P^{V}W^{VI}_{11}O_{39}]^{7-}$ $[K^+]$	–10.95	(*115*)
Wells-Dawson type *(Fig. S31)*		
α-$[P^{V}_2W^{VI}_{18}O_{62}]^{6-}$ $[Na^+]$	–12.7	(*112*, *113*)
β-$[P^{V}_2W^{VI}_{18}O_{62}]^{6-}$ $[Na^+]$	–11 and –11.6 for two non-equivalent P ions	(*112*)
α_1-$[P^{V}_2W^{VI}_{17}O_{61}]^{10-}$	–8.53 and –12.86 for two non-equivalent P ions	(*116*)
α_2-$[P^{V}_2W^{VI}_{17}O_{61}]^{10-}$	–6.8 and –13.63 for two non-equivalent P ions	(*112*)
α_2-$[P^{V}_2W^{VI}_{17}O_{61}]^{10-}$ $[Na^+]$	–6.9 and –13.7 for two non-equivalent P ions	(*114*)
Preyssler type *(Fig. S67)*		
$[NaP^{V}_5W^{VI}_{30}O_{114}]^{14-}$	–9.489	(*14*)
Other phosphotungstates *(Fig. S11)*		
$[H_6P^{V}_2W^{VI}_{21}O_{71}]^{4-}$	–13.2	(*37*)
$[P^{V}_2W^{VI}_{20}O_{70}(H_2O)_2]^{10-}$ $[Na^+]$	–14.1	(*37*)
$[P^{V}_2W^{VI}_5O_{23}]^{6-}$	–2.4	(*37*)
Zr-containing phosphotungstates *(Fig. S115)*		
$[Zr(\alpha\text{-}P^{V}W^{VI}_{11}O_{39})_2]^{10-}$	–14.5 and –14.6 for two non-equivalent P ions	(*15*)
$[\{\alpha\text{-}P^{V}W^{VI}_{11}O_{39}Zr(\mu\text{-}OH)(H_2O)\}_2]^{8-}$	–13.49	(*15*)
$[\{\alpha\text{-}P^{V}W^{VI}O_{39}Zr(\mu\text{-}OH)(H_2O)\}_2]^{8-}$	–13.86	(*59*)
$[\alpha\text{-}P^{V}W^{VI}O_{39}Zr(OH)(H_2O)]^{4-}$	–13.7	(*117*)
POMos		
Keggin type *(Fig. S80)*		
α-$[P^{V}Mo^{VI}_{12}O_{40}]^{3-}$ $[H^+]$	–3.9	(*112*)
	one signal in the range from –3.76 to –3.98	(*118*)
α-$[P^{V}Mo^{VI}_{12}O_{40}]^{3-}$ $[Na^+]$	–3.20	(*119*, *120*)

α-$[P^{V}Mo^{VI}_{11}O_{39}]^{7-}$	one signal in the range from –0.78 to -1.20	(*119*)
	one signal in the range from -1.05 to -0.79	(*120*)
α-$[P^{V}Mo^{VI}_{11}O_{39}]^{7-}$ $[H^{+}]$	one signal in the range from –1.69 to –1.73	(*118*)
α-$[P^{V}Mo^{VI}_{9}O_{31}(OH)_{3}]^{6-}$	one signal in the range from –1.15 to 1.00	(*119*)
	–0.85	(*120*)
	–0.65	(*118*)
	Wells-Dawson type *(Fig. S98)*	
α-$[P^{V}_{2}Mo^{VI}_{18}O_{62}]^{6-}$ $[Na^{+}]$	–3.4	(*112*)
	–2.4	(*120*)
α-$[P^{V}_{2}Mo^{VI}_{18}O_{62}]^{6-}$	–2.53	(*121*)
	–2.95	(*114*)
α-$[P^{V}_{2}Mo^{VI}_{18}O_{62}]^{6-}$ $[H^{+}]$	one signal in the range from –3.24 to –3.27	(*118*)
α-$[P^{V}_{2}Mo^{VI}_{16}Mo^{V}_{2}O_{62}]^{8-}$ $2e^{-}$ red	–4.6	(*121*)
β-$[P^{V}_{2}Mo^{VI}_{18}O_{62}]^{6-}$	–2.47 and –1.95 for two non-equivalent P ions	(*114*)
γ-$[P^{V}_{2}Mo^{VI}_{18}O_{62}]^{6-}$	–1.513	(*114*)
	Other phosphomolybdates *(Fig. S80 and S98)*	
$[P^{V}_{2}Mo^{VI}_{5}O_{23}]^{6-}$	one signal in the range from 2.35 to 1.86	(*119*)
$[P^{V}_{2}Mo^{VI}O_{23}]^{6-}$	2.0	(*120*)
$[P^{V}Mo^{VI}_{6}O_{25}]^{9-}$	0.5	(*120*)
	0.65	(*120*)
^{51}V		
	POVs *(Fig. S131)*	
$[V^{V}O_{4}]^{-}$	–541	(*122*, *123*)
$[V^{V}_{2}O_{7}]^{4-}$	–561	(*122*, *123*)
$H[V^{V}_{3}O_{10}]^{4-}$	–570	(*122*, *123*)
$[V^{V}_{4}O_{12}]^{4-}$	–578	(*122*, *123*)
$[V^{V}_{5}O_{15}]^{5-}$	–586	(*122*, *123*)
$[V^{V}_{10}O_{28}]^{6-}$	–422, –496, –513	(*122*, *123*)
$H[V^{V}_{10}O_{28}]^{5-}$	–424, –500, –516	(*122*, *123*)
$H_{2}[V^{V}_{10}O_{28}]^{4-}$	–425, –506, –524	(*122*, *123*)
$H_{3}[V^{V}_{10}O_{28}]^{3-}$	–427, –515, –534	(*122*, *123*)
^{183}W		
	POTs	
$[Te^{VI}W^{VI}_{6}O_{24}]^{6-}$ (*Fig. S148*)	–115.8	(*62*)
α-$[Si^{IV}W^{VI}_{12}O_{40}]^{4-}$ $[H^{+}]$ (*Fig. S30*)	–104.0	(*124*)

α-$[Si^{IV}W^{VI}_{11}O_{39}]^{8-}$ [Na^+] (*Fig. S30*)	–100.9, –116.2, –121.3, –127.9, –143.2, –176.2	(*124*)
α-$[P^{V}W^{VI}_{12}O_{40}]^{3-}$ [H^+] (*Fig. S11*)	–99.4	(*125*)
α-$[P^{V}W^{VI}_{11}O_{39}]^{7-}$ [Na^+] (*Fig. S11*)	–97.3; –102; –108.9; –116.6; –132.0; –152.1	(*125*)
$[NaP^{V}_{5}W^{VI}_{30}O_{114}]^{14-}$ (*Fig. S67*)	–207.6; −209.7; −275.5; −287.8	(*125*)
α-$[P^{V}_{2}W^{VI}_{18}O_{62}]^{6-}$ [Na^+] (*Fig. S31*)	−128.1; −173.8	(*125*)
α_2-$[P^{V}_{2}W^{VI}_{17}O_{61}]^{10-}$ [Na^+] (*Fig. S31*)	−127.8, −140.8, −159.6, −175.6, −179.6, −218.9, −222.7, −225.0, −242.3	(*125*)

5. The Keggin PW_{12} POT

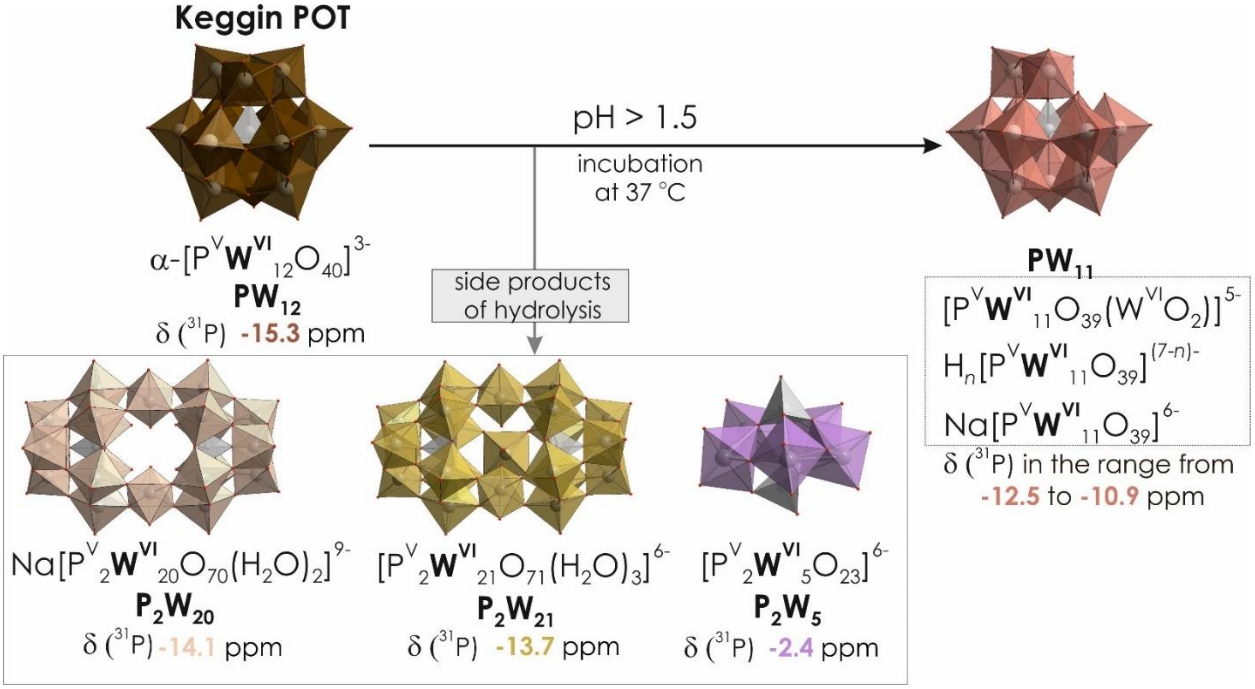

Fig. S11. Hydrolysis of PW_{12}.

Structure of the Keggin POT $[\alpha\text{-}P^{V}W^{VI}_{12}O_{40}]^{3-}$ (**PW_{12}**) and its hydrolysis scheme to the $[P^{V}W^{VI}_{11}O_{39}]^{7-}$ (**PW_{11}**, n = 2 or 3 in the protonated form) monolacunary anion based on the here presented investigation, which is in accordance with the literature data (*36*). The polyanions formed during hydrolysis in the pH range from 1.5 to 7 are shown and named as side products of the hydrolysis. Color code: $\{WO_6\}$, brown, pink, beige, yellow, and lavender; $\{PO_4\}$, grey; O, red. To identify the individual anions, they are shown in different colors, with the same color code being selected for a specific anion throughout all figures and tables in the main manuscript and the supporting information.

5.1. pH of Keggin POT solutions

Table S5. pH in PW_{12} solutions.

pH measured in triplicate of 10 mM **$Na_3[\alpha\text{-}P^{V}W^{VI}_{12}O_{40}]$** dissolved in D_2O and 0.1 M buffers (acetic acid – sodium acetate pH 4 – 5.5; sodium phosphate pH 3 – 8 (while phosphate does not buffer at pH range from 3.5 – 5.5, experiments were conducted at this pH to provide comparisons to previously published studies (*29*)); citric acid – sodium citrate pH 3 – 6.5; MES pH 5.5; PBS pH 7.4; tris-HCl pH 7.5 – 8.5; HEPES pH 7 – 8; MHB pH 7.4; Nutrient mixture F-12 Ham pH 7.4 and glycine-NaOH pH 8.6). The glycine-NaOH buffer was used at two additional concentrations of 0.2 and 0.5 M.

**pH**	**$Na_3[\alpha\text{-}PW_{12}O_{40}]$** (10 mM) in Solvent / Buffer / Medium	pH after dissolving **$Na_3[\alpha\text{-}P^{V}W^{VI}_{12}O_{40}]$** (10 mM) at RT				pH after 24 h incubation of **$Na_3[\alpha\text{-}P^{V}W^{VI}_{12}O_{40}]$** (10 mM) at 37 °C			
		Sample			Mean of 1 to 3 ± SD[a]	Sample			Mean of 1 to 3 ± SD[a]
		#1	#2	#3		#1	#2	#3	
-	D_2O	2.02	2.00	1.99	2.00 ± 0.02	1.80	1.92	1.89	1.87 ± 0.06
Strongly acidic environment 3 ≤ pH ≤ 4									
3	0.1 M Sodium phosphate ($H_2PO_4^-/H_3PO_4$) pH 3	2.40	2.45	2.42	2.42 ± 0.03	2.30	2.33	2.35	2.33 ± 0.03
	0.1 M Citric acid – sodium citrate (H_3Cit/H_2Cit^-) pH 3	2.12	2.10	2.12	2.11 ± 0.01	2.10	1.99	1.98	2.02 ± 0.07
4	0.1 M Sodium phosphate ($H_2PO_4^-/H_3PO_4$) pH 4	2.60	2.64	2.63	2.62 ± 0.02	2.50	2.55	2.53	2.53 ± 0.03
	0.1 M Citric acid – sodium citrate ($H_2Cit^-/HCit^{2-}$) pH 4	3.04	3.10	3.05	3.06 ± 0.03	2.50	2.63	2.62	2.58 ± 0.07
	0.1 M Acetic acid – sodium acetate ($OAc^-/HOAc$) pH 4	2.45	2.10	2.50	2.35 ± 0.22	2.00	2.34	2.40	2.25 ± 0.22
Moderately acidic environment 5 ≤ pH ≤ 6									
5	0.1 M Sodium phosphate ($H_2PO_4^-/H_3PO_4$) pH 5	2.60	2.59	2.59	2.59 ± 0.01	2.44	2.54	2.49	2.49 ± 0.05
	0.1 M Citric acid – sodium citrate ($H_2Cit^-/HCit^{2-}$) pH 5	4.32	4.20	4.32	4.28 ± 0.07	4.20	4.12	4.10	4.14 ± 0.05
	0.1 M Acetic acid – sodium acetate ($OAc^-/HOAc$) pH 5	4.23	4.20	4.19	4.21 ± 0.02	3.20	4.16	4.12	3.83 ± 0.54
5.5	0.1 M Acetic acid – sodium acetate ($OAc^-/HOAc$) pH 5.5	4.64	4.30	4.48	4.47 ± 0.17	4.00	4.58	4.40	4.33 ± 0.30
	0.1 M MES[b] pH 5.5	2.05	1.97	1.97	2.00 ± 0.05	1.85	1.85	1.94	1.88 ± 0.05
6	0.1 M Sodium phosphate ($HPO_4^{2-}/H_2PO_4^-$) pH 6	2.97	2.98	2.89	2.95 ± 0.05	2.87	2.91	2.47	2.75 ± 0.24
	0.1 M Citric acid – sodium citrate ($HCit^{2-}/Cit^{3-}$) pH 6	5.40	5.20	5.36	5.32 ± 0.11	5.20	5.64	5.52	5.45 ± 0.23
Neutral environment 6.5 ≤ pH ≤ 7.5									
6.5	0.1 M Citric acid – sodium citrate ($HCit^{2-}/Cit^{3-}$) pH 6.5	5.71	5.60	5.63	5.65 ± 0.06	5.60	5.55	5.92	5.77 ± 0.64
7	0.1 M Sodium phosphate ($HPO_4^{2-}/H_2PO_4^-$) pH 7	6.49	6.56	6.43	6.49 ± 0.07	6.46	6.48	6.36	6.43 ± 0.06
	0.1 M HEPES[c] pH 7	4.77	4.00	4.78	4.52 ± 0.45	4.30	4.66	4.71	4.56 ± 0.22

7.4	PBS[d] pH 7.4	2.18	2.19	2.16	2.17 ± 0.02	2.11	2.15	2.11	2.12 ± 0.02
	MHB[e] pH 7.4	3.79	3.40	4.15	3.78 ± 0.38	3.4	3.56	3.75	3.57 ± 0.18
	Nutrient mixture F-12 Ham[f]	2.15	2.10	2.20	2.15 ± 0.05	2.12	2.07	2.18	2.12 ± 0.06
7.5	0.1 M tris-HCl[g] pH 7.5	2.36	2.00	2.32	2.22 ± 0.20	2.38	2.31	2.29	2.33 ± 0.05
Moderately alkaline environment 8 ≤ pH ≤ 8.6									
8	0.1 M Sodium phosphate ($HPO_4^{2-}/H_2PO_4^{-}$) pH 8	6.85	6.83	6.78	6.82 ± 0.04	6.79	6.84	6.73	6.79 ± 0.06
	0.1 M HEPES pH 8	6.90	6.42	6.86	6.73 ± 0.26	6.83	6.91	6.85	6.86 ± 0.04
	0.1 M tris-HCl pH 8	3.11	3.20	3.08	3.13 ± 0.06	2.98	3.06	3.06	3.03 ± 0.05
8.5	0.1 M tris-HCl pH 8.5	6.60	6.10	6.76	6.49 ± 0.34	5.94	6.53	6.74	6.40 ± 0.41
8.6	0.1 M glycine-NaOH pH 8.6	3.15	2.90	3.19	3.08 ± 0.16	3.14	3.07	3.02	3.08 ± 0.06
	0.2 M glycine-NaOH pH 8.6	3.57	3.40	3.91	3.63 ± 0.26	3.61	3.48	3.61	3.57 ± 0.08
	0.5 M glycine-NaOH pH 8.6	4.66	4.70	4.95	4.77 ± 0.16	4.48	4.46	4.59	4.51 ± 0.07

[a]SD – standard deviation; [b]MES – 2-(N-morpholino)ethanesulfonic acid, $C_6H_{13}NO_4S$ (Figure S1); [c]HEPES – 4-(2-hydroxyethyl)-1-piperazineethanesulfonic acid, $C_8H_{18}N_2O_4S$ (Figure S1); [d]PBS – phosphate buffer saline; [e]MHB – Mueller-Hinton broth, for more detailed information about composition see https://labmal.com/2019/11/20/mueller-hinton-agar-and-mueller-hinton-broth/; [f]Nutrient mixture F-12 Ham contains sodium pyruvate (0.11 g/L), phenol red, L-glutamine, and does not contain $NaHCO_3$ and HEPES, for more details please see https://www.sigmaaldrich.com/AT/en/technical-documents/technical-article/cell-culture-and-cell-culture-analysis/mammalian-cell-culture/F-12-ham; [g]tris – tris(hydroxymethyl)aminomethane, $C_4H_{11}NO_3$ (Figure S1).

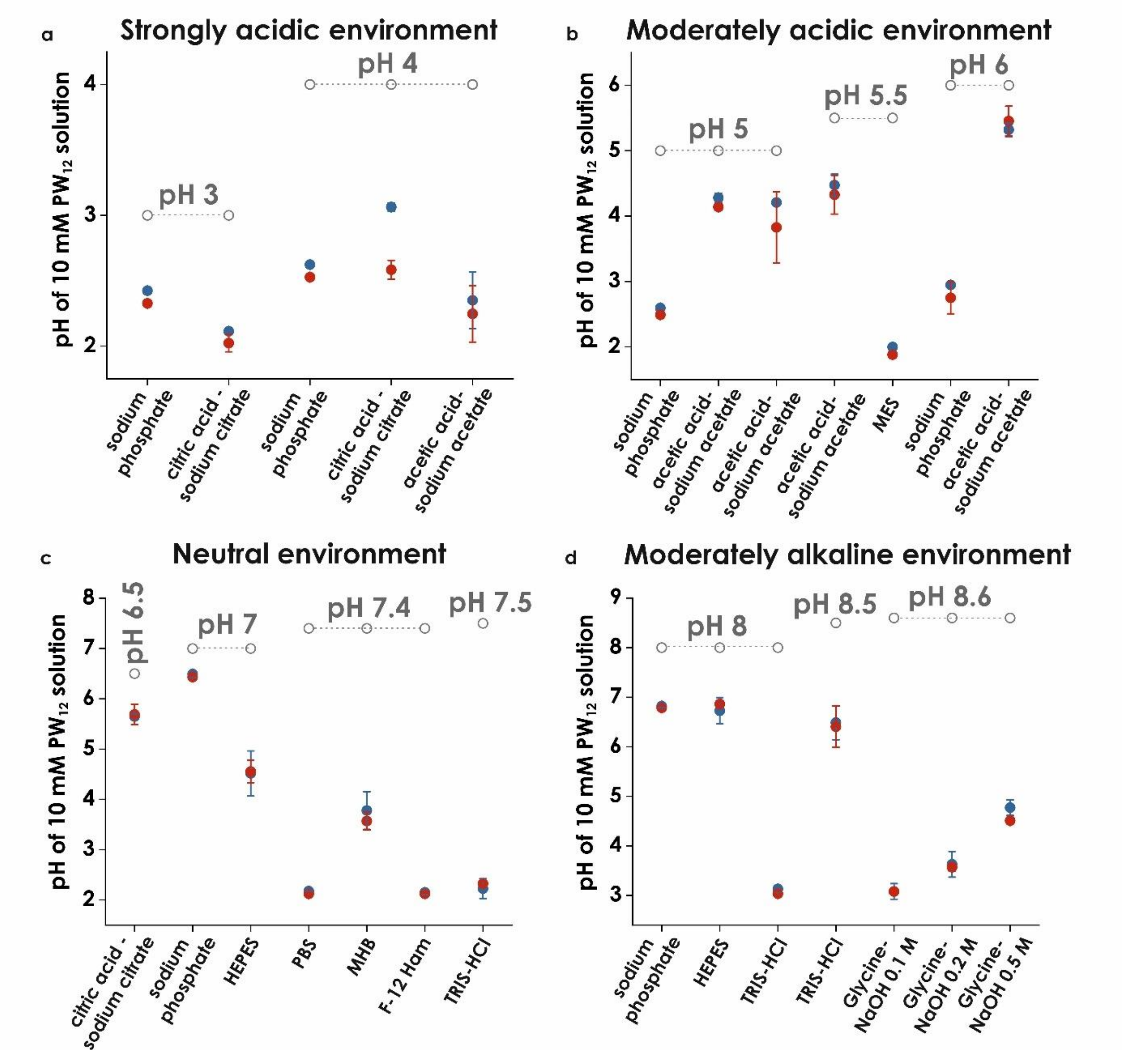

Fig. S12. pH in PW_{12} solutions.

The mean of the pH values measured in triplicates for 10 mM solutions of **$Na_3[\alpha\text{-}P^{V}W^{VI}_{12}O_{40}]$** in different buffers in a) strongly acidic, b) moderately acidic, c) neutral, and d) moderately alkaline environments. The pH of the starting buffers is shown with a gray dashed line; the plots of the measured pH values immediately after the preparation of the solutions are shown in blue and after 24 h incubation at 37 °C in red. The error bar shows standard deviations (**Table S5**).

5.2. ^{31}P NMR and ^{183}W spectroscopic studies of Keggin POT solutions

All ^{31}P and ^{183}W peaks with the highest intensity were unambiguously assigned based on the literature data from **Table S4**. In some ^{31}P spectra, chemical shifts for peaks of lower intensity have not yet been described in the literature and therefore can not be assigned in this work.

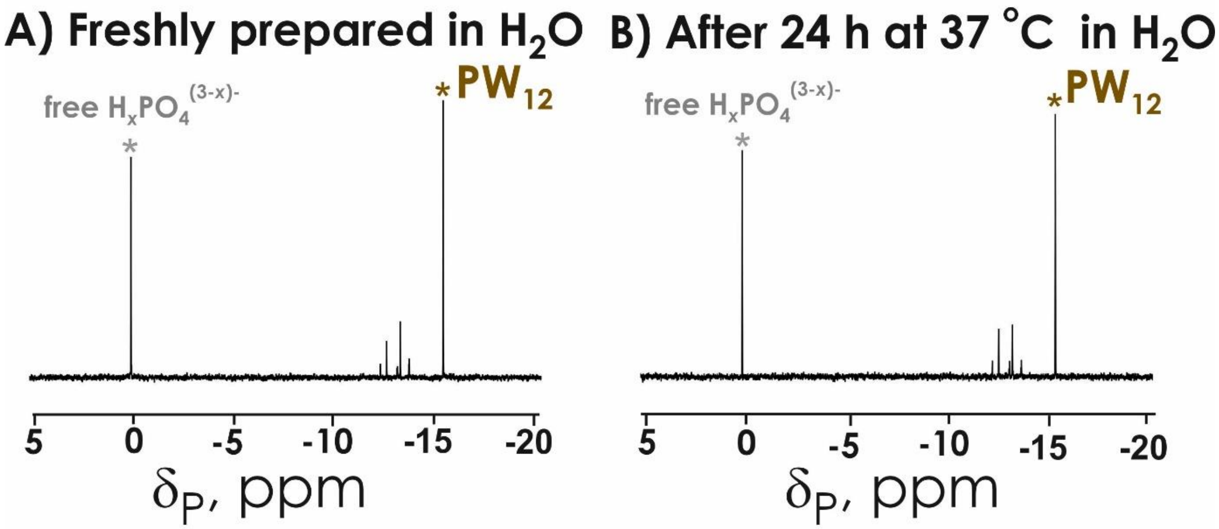

Fig. S13. ^{31}P NMR spectra of PW_{12} in H_2O.

^{31}P NMR spectra for 10 mM solutions of **$Na_3[\alpha\text{-}P^{V}W^{VI}_{12}O_{40}]$** in H_2O that were recorded approximately one hour after preparation (**A**) and after incubation for 24 h at 37 °C (**B**). The structures of all POMs are shown in **Figure S11**. The chemical shifts and percentages of parent and formed species are given in **Tables S6** and **S7**. To identify the individual anions, they are shown in different colors, with the same color code being selected for a specific anion throughout all figures and tables in the main manuscript and the supporting information.

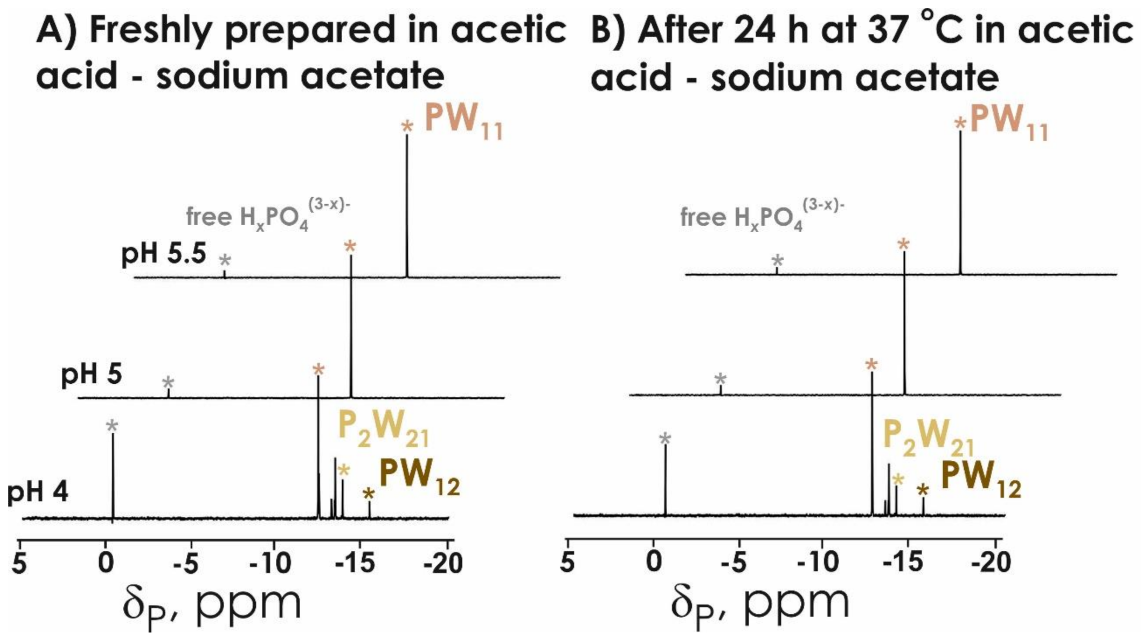

Fig. S14. ^{31}P NMR spectra of PW_{12} in acetic acid – sodium acetate buffer.

^{31}P NMR spectra for 10 mM solutions of **$Na_3[\alpha\text{-}P^{V}W^{VI}_{12}O_{40}]$** in 0.1 M acetic acid – sodium acetate buffer (pH 4 – 5.5) that were recorded approximately one hour after preparation (**A**) and after incubation for 24 h at 37 °C (**B**). The structures of all POMs are shown in **Figure S11**. The chemical shifts and percentages of parent and formed species are given in **Tables S6** and **S7**. To identify the individual anions, they are shown in different colors, with the same color code being selected for a specific anion throughout all figures and tables in the main manuscript and the supporting information.

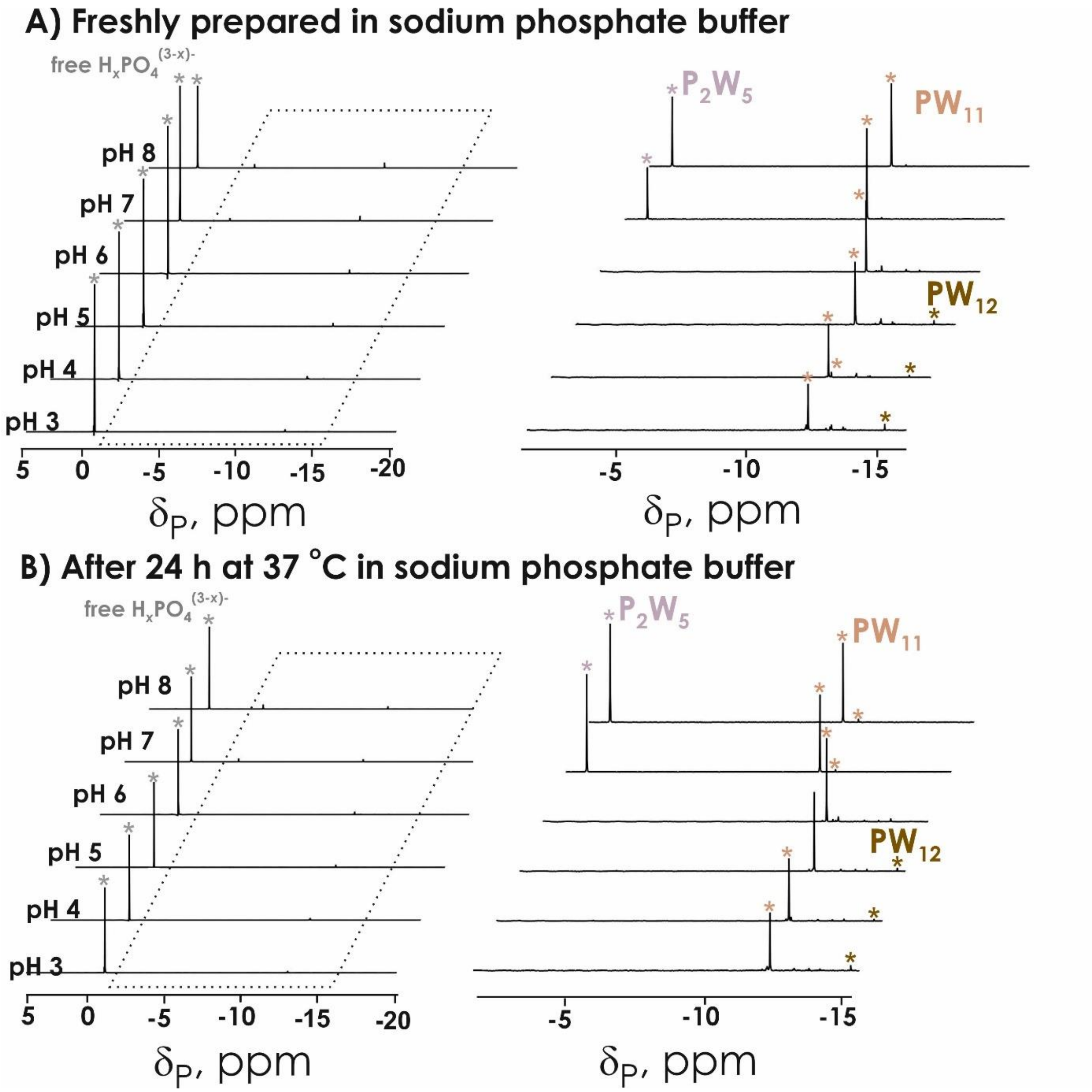

Fig. S15. ^{31}P NMR spectra of PW_{12} in sodium phosphate buffer.

^{31}P NMR spectra for 10 mM solutions of **$Na_3[\alpha\text{-}P^VW^{VI}_{12}O_{40}]$** in 0.1 M sodium phosphate buffer (pH 3 – 8) that were recorded approximately one hour after preparation (**A**) and after incubation for 24 h at 37 °C (**B**). The structures of all POMs are shown in **Figure S11**. The chemical shifts and percentages of parent and formed species are given in **Tables S6** and **S7**. To identify the individual anions, they are shown in different colors, with the same color code being selected for a specific anion throughout all figures and tables in the main manuscript and the supporting information.

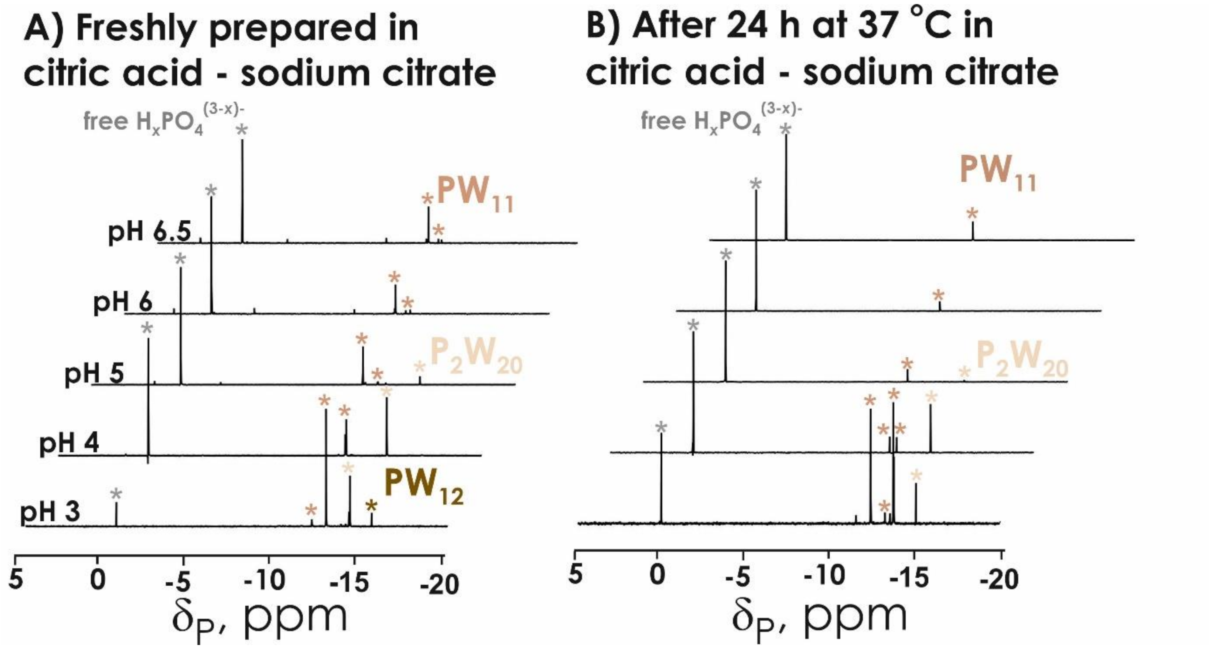

Fig. S16. ^{31}P NMR spectra of PW_{12} in citric acid – sodium citrate buffer.

^{31}P NMR spectra for 10 mM solutions of **$Na_3[\alpha\text{-}P^VW^{VI}_{12}O_{40}]$** in 0.1 M citric acid – sodium citrate buffer (pH 3 – 6.5) that were recorded approximately one hour after preparation (**A**) and after incubation for 24 h at 37 °C (**B**). The structures of all POMs are shown in **Figure S11**. The chemical shifts and percentages of parent and formed species are given in **Tables S6** and **S7**. To identify the individual anions, they are shown in different colors, with the same color code being selected for a specific anion throughout all figures and tables in the main manuscript and the supporting information.

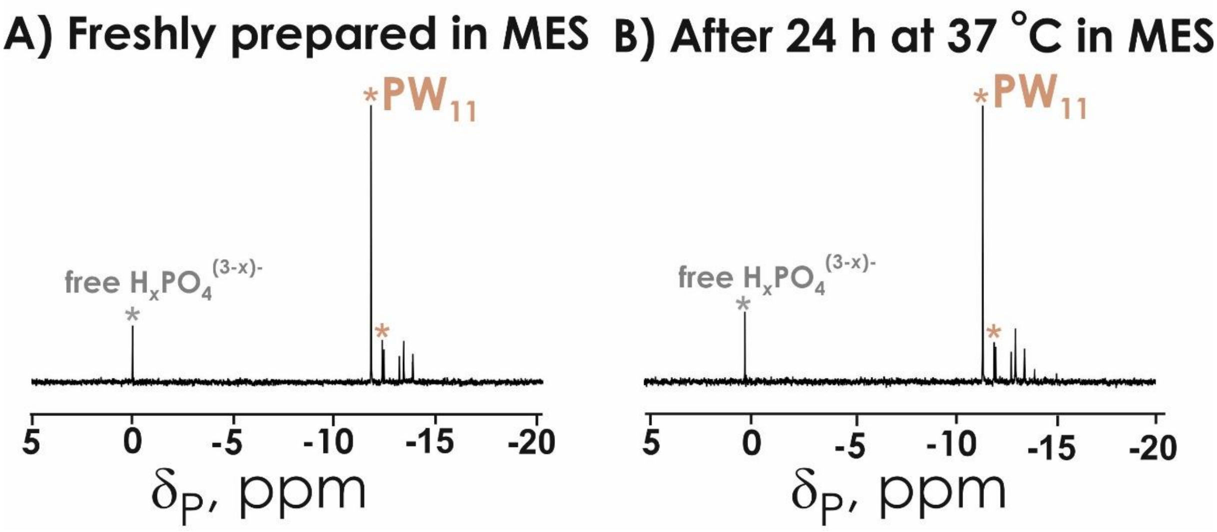

Fig. S17. ^{31}P NMR spectra of PW_{12} in MES buffer.

^{31}P NMR spectra for 10 mM solutions of **$Na_3[\alpha\text{-}P^{V}W^{VI}_{12}O_{40}]$** in 0.1 M MES buffer pH 5.5 that were recorded approximately one hour after preparation (**A**) and after incubation for 24 h at 37 °C (**B**). The structures of all POMs are shown in **Figure S11**. The chemical shifts and percentages of parent and formed species are given in **Tables S6** and **S7**. To identify the individual anions, they are shown in different colors, with the same color code being selected for a specific anion throughout all figures and tables in the main manuscript and the supporting information.

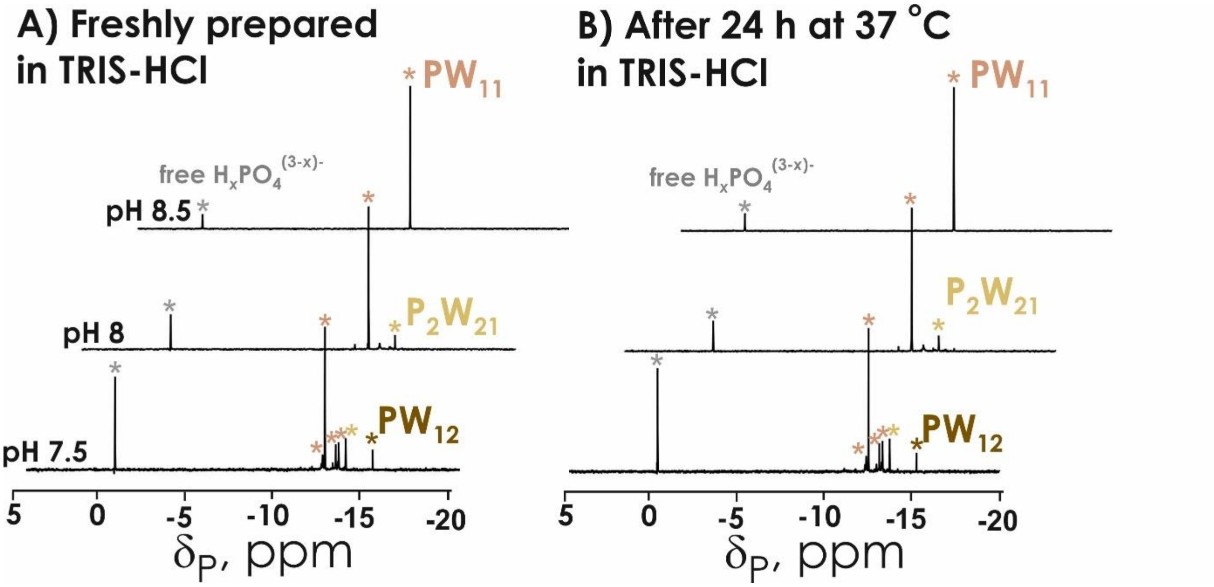

Fig. S18. ^{31}P NMR spectra of PW_{12} in tris-HCl buffer.

^{31}P NMR spectra for 10 mM solutions of **$Na_3[\alpha\text{-}P^VW^{VI}_{12}O_{40}]$** in 0.1 M tris-HCl buffer (pH 7.5 – 8.5) that were recorded approximately one hour after preparation (**A**) and after incubation for 24 h at 37 °C (**B**). The structures of all POMs are shown in **Figure S11**. The chemical shifts and percentages of parent and formed species are given in **Tables S6** and **S7**. To identify the individual anions, they are shown in different colors, with the same color code being selected for a specific anion throughout all figures and tables in the main manuscript and the supporting information.

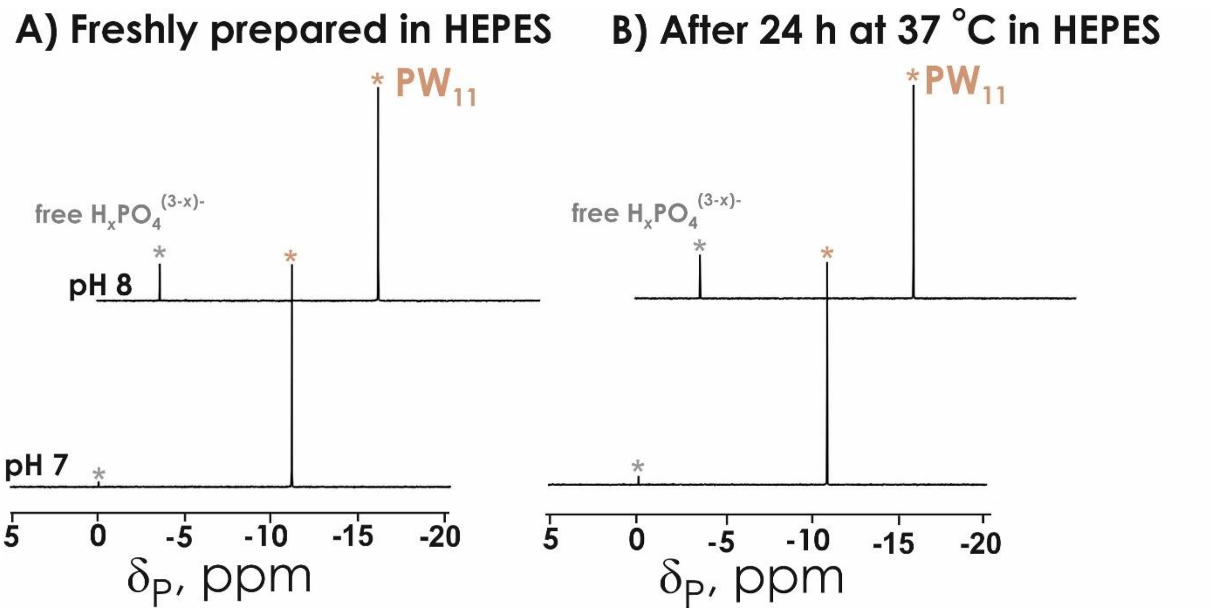

Fig. S19. ^{31}P NMR spectra of PW$_{12}$ in HEPES buffer.

^{31}P NMR spectra for 10 mM solutions of **$Na_3[\alpha\text{-}P^VW^{VI}_{12}O_{40}]$** in 0.1 M HEPES buffer (pH 7 and 8) that were recorded approximately one hour after preparation (**A**) and after incubation for 24 h at 37 °C (**B**). The structures of all POMs are shown in **Figure S11**. The chemical shifts and percentages of parent and formed species are given in **Tables S6** and **S7**. To identify the individual anions, they are shown in different colors, with the same color code being selected for a specific anion throughout all figures and tables in the main manuscript and the supporting information.

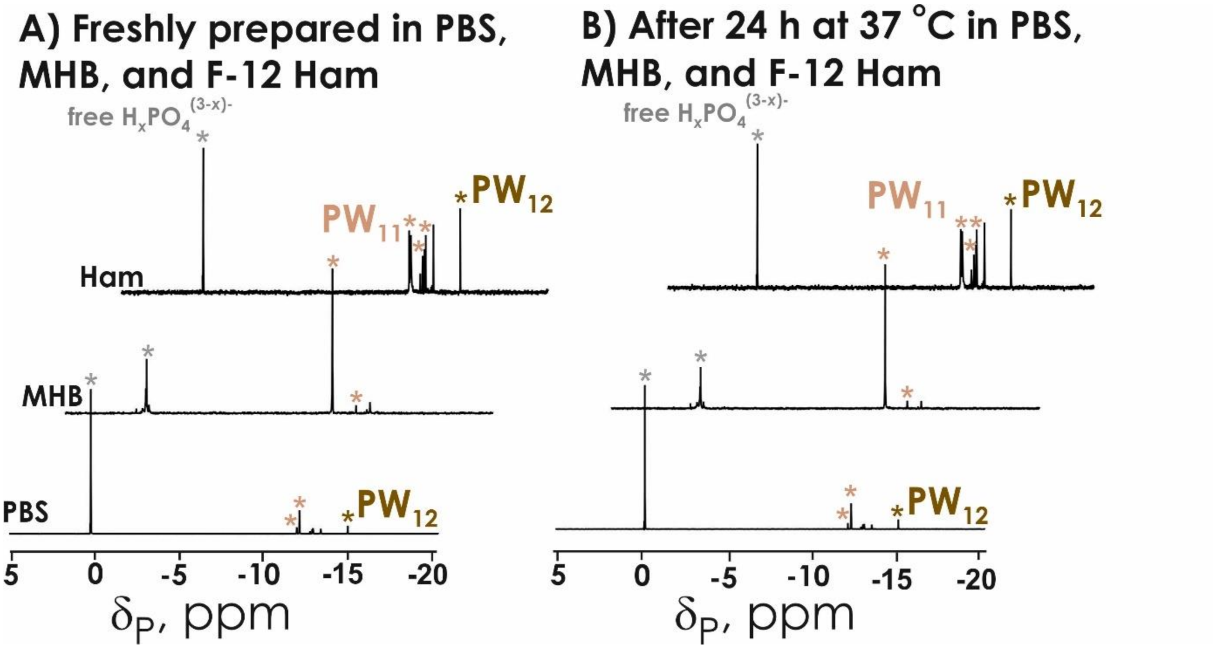

Fig. S20. ^{31}P NMR spectra of PW_{12} in PBS, MHB, and nutrient mixture F-12 Ham.

^{31}P NMR spectra for 10 mM solutions of **$Na_3[\alpha\text{-}P^{V}W^{VI}_{12}O_{40}]$** in 0.1 M PBS, MHB and nutrient mixture F-12 Ham (pH 7.4) that were recorded approximately one hour after preparation (**A**) and after incubation for 24 h at 37 °C (**B**). The structures of all POMs are shown in **Figure S11**. The chemical shifts and percentages of parent and formed species are given in **Tables S6** and **S7**. To identify the individual anions, they are shown in different colors, with the same color code being selected for a specific anion throughout all figures and tables in the main manuscript and the supporting information.

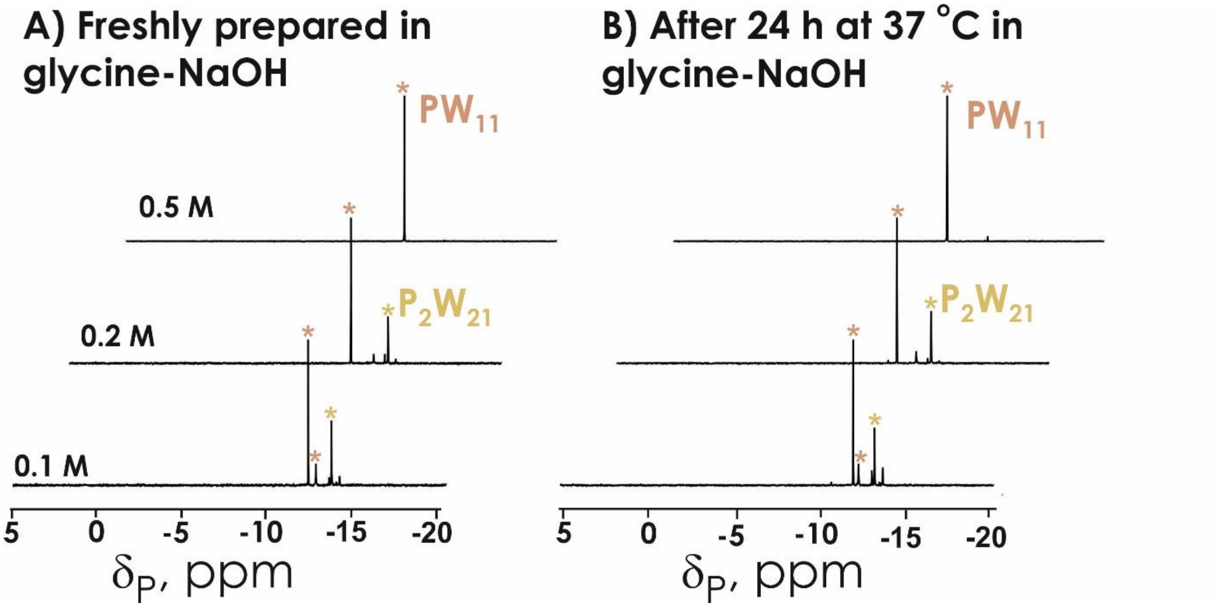

Fig. S21. ^{31}P NMR spectra of PW_{12} in glycine-NaOH buffer

^{31}P NMR spectra for 10 mM solutions of **$Na_3[\alpha\text{-}P^VW^{VI}{}_{12}O_{40}]$** in glycine-NaOH (pH 8.6) with concentrations of 0.1. 0.2 and 0.5 M that were recorded approximately one hour after preparation (**A**) and after incubation for 24 h at 37 °C (**B**). The structures of all POMs are shown in **Figure S11**. The chemical shifts and percentages of parent and formed species are given in **Tables S6** and **S7**. To identify the individual anions, they are shown in different colors, with the same color code being selected for a specific anion throughout all figures and tables in the main manuscript and the supporting information.

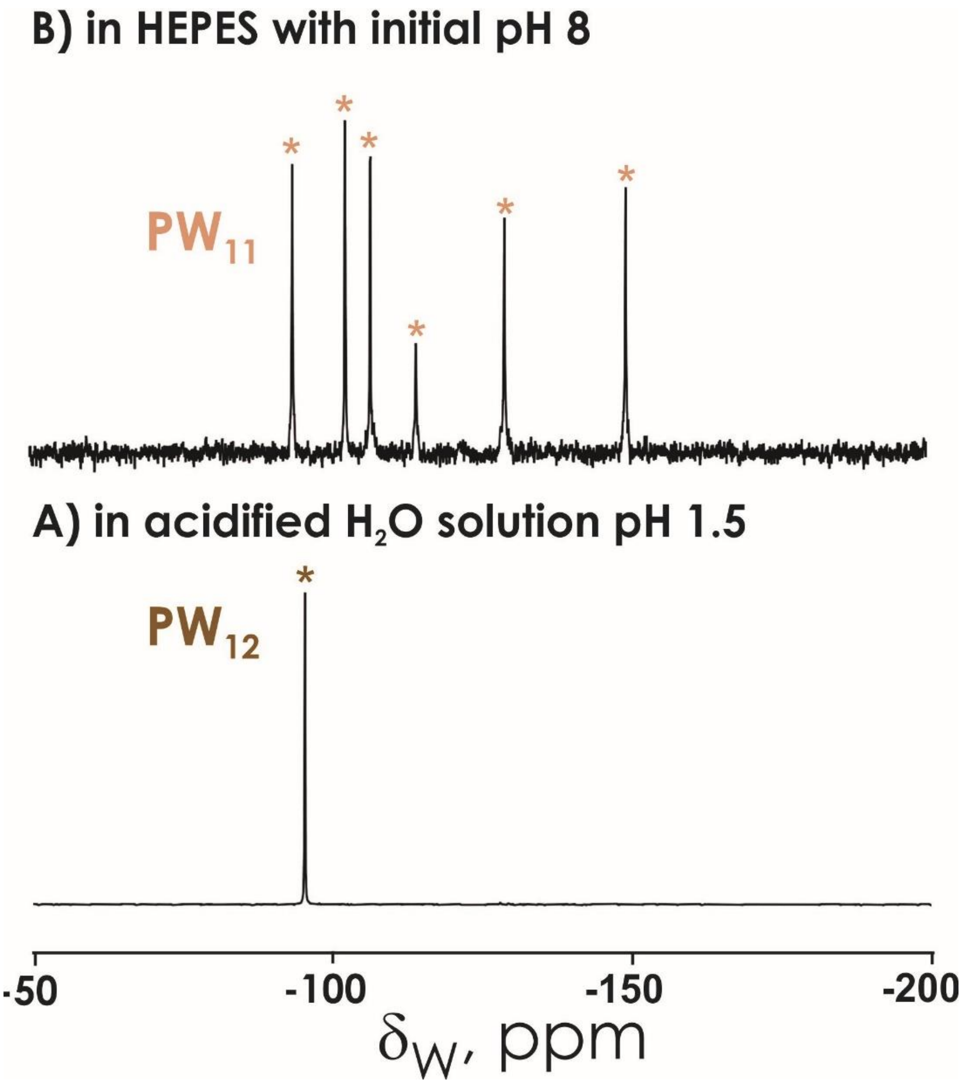

Fig. S22. ^{183}W NMR spectra of PW_{12}.

^{183}W NMR spectra (acquisition details in the method section in the main text) for 20 mM solutions of $Na_3[\alpha\text{-}PW_{12}O_{40}]$ in (**A**) acidified to pH 1.5 aqueous solution showing one signal at –95.5 ppm that corresponds to twelve equivalent W ions in $[\alpha\text{-}PW_{12}O_{40}]^{3-}$; (**B**) in 0.2 M HEPES pH 8 showing 6 signals at –94.4 (2W), – 103.2 (2W), –107.4 (2W), –115.0 (1W), –129.8 (2W), and –150.0 (2W) ppm that correspond to six types of W ions in $[\alpha\text{-}PW_{11}O_{39}]^{7-}$ (**Table S4**). The structures of all POMs are shown in **Figure S11**. To identify the individual anions, they are shown in different colors, with the same color code being selected for a specific anion throughout all figures and tables in the main manuscript and the supporting information.

Table S6. Analysis of NMR spectroscopic data recorded in PW_{12} solutions at room temperature.

Chemical shifts in ^{31}P NMR spectra measured in triplicate of **$Na_3[\alpha\text{-}P^{V}W^{VI}_{12}O_{40}]$** (10 mM) dissolved in D_2O and 0.1 M buffers (acetic acid – sodium acetate pH 4 – 5.5; sodium phosphate pH 3 – 8 (while phosphate does not buffer at pH range from 3.5 – 5.5, experiments were conducted at this pH to provide comparisons to previously published studies (*29*)); citric acid – sodium citrate pH 3 – 6.5; MES pH 5.5; PBS pH 7.4; tris-HCl pH 7.5 – 8.5; HEPES pH 7 – 8; MHB pH 7.4; Nutrient mixture F-12 Ham pH 7.4 and glycine-NaOH pH 8.6) (**Figures S13** – **S21**). The glycine-NaOH buffer was used in two additional concentrations of 0.2 and 0.5 M. The content of species was calculated based on integration of ^{31}P signals considering only signals associated with POTs. Signals were assigned based on the literature data summarized in **Table S4**.

pH	**$Na_3[PW_{12}O_{40}]$** (10 mM) in Solvent / Buffer / Medium	Chemical shifts δ ^{31}P [ppm]	% of **PW_{12}** at RT in **$Na_3[\alpha\text{-}P^{V}W^{VI}_{12}O_{40}]$** (10 mM) solution				% of **PW_{11}** at RT in **$Na_3[\alpha\text{-}P^{V}W^{VI}_{12}O_{40}]$** (10 mM) solution				% of **P_2W_{21}** at RT in **$Na_3[\alpha\text{-}P^{V}W^{VI}_{12}O_{40}]$** (10 mM) solution				% of P_2W_{20} at RT in **$Na_3[\alpha\text{-}P^{V}W^{VI}_{12}O_{40}]$** (10 mM) solution$_0$				% of **P_2W_5** at RT in **$Na_3[\alpha\text{-}P^{V}W^{VI}_{12}O_{40}]$** (10 mM) solution				Average % of other POTs present in solution
			Sample			Mean of 1 to 3 ± SD[a]	Sample			Mean of 1 to 3 ± SD[a]	Sample			Mean of 1 to 3 ± SD	Sample			Mean of 1 to 3 ± SD	Sample			Mean of 1 to 3 ± SD	
			#1	#2	#3		#1	#2	#3		#1	#2	#3		#1	#2	#3		#1	#2	#3		
-	D_2O	0; **–12.2**; **–12.5**; –13.0; –13.2; –13.6; **–13.7**; **–15.3**	63	66	60	63 ± 3	18	10	15	14 ± 4	10	15	7	11 ± 4	0	0	0	0	0	0	0	0	12
Strongly acidic environment 3 ≤ pH ≤ 4																							
3	0.1 M Sodium phosphate ($H_2PO_4^-$ /H_3PO_4) pH 3	0; **–12.3**; **–12.4**; –13.0; –13.3; **–13.7**; –13.8; **–15.3**	5	2	6	4 ±2	80	94	85	86 ± 7	10	4	8	7 ± 3	0	0	0	0	0	0	0	0	3
3	0.1 M Citric acid – sodium citrate (H_3Cit/H_2Cit^-) pH 3	0; **–11.8**; –12.6; –13.5; –13.7; **–14.0**; **–15.3**	4	5	5	5 ± 1	65	53	61	60 ± 6	0	0	0	0	31	37	31	33 ± 3	0	0	0	0	4
4	0.1 M Sodium phosphate	1.3; –0; **–12.2**; –	4	1	1	2 ± 2	71	75	88	78 ± 9	0	0	0	0	0	0	0	0	0	0	0	0	20

	$(H_2PO_4^-/H_3PO_4)$ pH 4	12.3; –13.2; –13.3; –13.7; –13.8; –15.3																					
	0.1 M Citric acid – sodium citrate $(H_2Cit^-/HCit^{2-})$ pH 4	1.3; 0; –11.4; –11.8; –12.5; –14.2	0	0	0	0	51	45	47	48 ± 3	0	0	0	0	47	55	48	50 ± 4	0	0	0	0	2
	0.1 M Acetic acid – sodium acetate $(OAc^-/HOAc)$ pH 4	0; –12.2; –12.3; –13.0; –13.2; –13.3; –13.7; –15.3	10	5	5	7 ± 3	49	51	53	51 ± 2	16	16	15	16 ± 1	0	0	0	0	0	0	0	0	27
Moderately acidic environment 5 ≤ pH ≤ 6																							
5	0.1 M Sodium phosphate $(H_2PO_4^-/H_3PO_4)$ pH 5	1.3; 0; –12.3; –13.0; –13.2; –13.3; –13.7; –13.8; –15.3	6	2	3	4 ± 2	73	85	81	80 ± 6	6	2	4	4 ± 2	0	0	0	0	0	0	0	0	12
	0.1 M Citric acid – sodium citrate $(H_2Cit^-/HCit^{2-})$ pH 5	1.6; 0; –2.4; –10.9; –11.0; –11.7; –11.8; –12.2; –14.3	0	0	0	0	77	86	81	81 ± 5	0	0	0	0	19	14	14	16 ± 3	0	0	0	0	3
	0.1 M Acetic acid – sodium acetate $(OAc^-/HOAc)$ pH 5	0; –10.9	0	0	0	0	100	100	100	100 ± 0	0	0	0	0	0	0	0	0	0	0	0	0	0
5.5	0.1 M Acetic acid – sodium acetate $(OAc^-/HOAc)$ pH 5.5	0; –10.9	0	0	0	0	100	100	100	100 ± 0	0	0	0	0	0	0	0	0	0	0	0	0	0

	0.1 M MES[b] pH 5.5	0; –11.6; –12.1; –12.2; –13.0; –13.2; –13.6; –15.2	2	0	0	1 ± 1	69	72	71	71 ± 2	9	8	9	9 ± 1	0	0	0	0	0	0	0	0	20
6	0.1 M Sodium phosphate (HPO_4^{2-} /$H_2PO_4^-$) pH 6	1.3; –0.7; 0; –11.8; –12.1; –12.2; –12.4; –13.3; –13.8	2	0	0	1 ± 1	89	95	82	89 ± 7	0	0	0	0	0	0	0	0	0	0	0	0	10
	0.1 M Citric acid – sodium citrate ($HCit^{2-}$ /Cit^{3-}) pH 6	2.4; –0; –2.4; –8.4; –10.8; –11.4; –11.7	0	0	0	0	89	92	90	90 ± 2	0	0	0	0	0	0	0	0	11	8	10	10 ± 2	0
Neutral environment 6.5 ≤ pH ≤ 7.5																							
6.5	0.1 M Citric acid – sodium citrate ($HCit^{2-}$ /Cit^{3-}) pH 6.5	2.8; 0.3; 0; –2.4; –8.3; –10.7; –10.8; –11.4; –11.6	0	0	0	0	92	94	95	93 ± 2	0	0	0	0	0	0	0	0	8	6	5	6 ± 2	0
7	0.1 M Sodium phosphate (HPO_4^{2-} /$H_2PO_4^-$) pH 7	3.3; 0.8; –2.4; –10.8; –11.4	0	0	0	0	67	64	66	66 ± 2	0	0	0	0	0	0	0	0	33	36	33	34 ± 2	0
	0.1 M HEPES[c] pH 7	0; –10.9	0	0	0	0	100	100	100	100 ± 0	0	0	0	0	0	0	0	0	0	0	0	0	0
7.4	PBS[d] pH 7.4	0; –12.2; –12.4; –13.0; –13.2; –13.7; –15.3	10	10	12	11 ± 1	49	54	56	53 ± 4	17	16	13	15 ± 2	0	0	0	0	0	0	0	0	36
	MHB[e] pH 7.4	0.6; 0; –0.2; –11.0; –12.4; –	0	0	0	0	72	85	91	83 ± 10	0	0	0	0	0	0	0	0	0	0	0	0	17

		13.1; –13.3																					
	Nutrient mixture F-12 Ham[f]	0; –12.2; –12.3; –12.9; –13.0; –13.2; –13.2; –13.7; –15.3	10	12	9	10 ± 2	54	52	56	54 ± 2	13	11	12	12 ± 1	0	0	0	0	0	0	0	0	24
7.5	0.1 M tris-HCl[g] pH 7.5	0; –11.7; –12.2; –12.3; –12.5; –12.9; –13.1; –13.2; –13.3; –13.7; –15.3	8	6	5	6 ± 2	43	55	55	51 ± 7	11	11	11	11 ± 0	0	0	0	0	0	0	0	0	32
Moderately alkaline environment 8 ≤ pH ≤ 8.6																							
8	0.1 M Sodium phosphate (HPO_4^{2-}/$H_2PO_4^{-}$) pH 8	3.5; 1.2; 0; –2.4; –10.8; –11.4	0	0	0	0	67	61	61	63 ± 3	0	0	0	0	0	0	0	0	33	39	39	37 ± 3	0
8	0.1 M HEPES pH 8	1.4; –10.8	0	0	0	0	100	100	100	100 ± 0	0	0	0	0	0	0	0	0	0	0	0	0	0
8	0.1 M tris-HCl pH 8	0; –10.9; –11.7; –12.4; –13.0; –13.3; –13.7	2	0	0	1 ± 1	74	79	87	80 ± 7	0	0	0	0	0	0	0	0	0	0	0	0	19
8.5	0.1 M tris-HCl pH 8.5	1.42; –10.9	0	0	0	0	100	100	100	100 ± 0	0	0	0	0	0	0	0	0	0	0	0	0	0
8.6	0.1 M glycine-NaOH pH 8.6	–11.9; –12.3; –13.1; –13.3; –13.5; –13.7	0	0	0	0	56	61	60	59 ± 3	7	6	5	6 ± 1	0	0	0	0	0	0	0	0	35
8.6	0.2 M glycine-NaOH pH 8.6	–11.1; –12.4; –13.0; –	0	0	0	0	70	79	75	75 ± 5	5	4	4	4 ± 1	0	0	0	0	0	0	0	0	21

		13.2; –13.7																					
	0.5 M glycine-NaOH pH 8.6	–10.9	0	0	0	0	100	100	100	100 ± 0	0	0	0	0	0	0	0	0	0	0	0	0	0

[a]SD – standard deviation; [b]MES – 2-(N-morpholino)ethanesulfonic acid, $C_6H_{13}NO_4S$ (Figure S1); [c]HEPES – 4-(2-hydroxyethyl)-1-piperazineethanesulfonic acid, $C_8H_{18}N_2O_4S$ (Figure S1); [d]PBS – phosphate buffer saline; [e]MHB – Mueller-Hinton broth, for more detailed information about composition see https://labmal.com/2019/11/20/mueller-hinton-agar-and-mueller-hinton-broth/; [f]Nutrient mixture F-12 Ham contains sodium pyruvate (0.11 g/L), phenol red, L-glutamine, and does not contain $NaHCO_3$ and HEPES, for more details please see https://www.sigmaaldrich.com/AT/en/technical-documents/technical-article/cell-culture-and-cell-culture-analysis/mammalian-cell-culture/F-12-ham; [G]tris – tris(hydroxymethyl)aminomethane, $C_4H_{11}NO_3$ (Figure S1).

Table S7. Analysis of NMR spectroscopic data of PW_{12} solutions investigated after 24 h incubation at 37 °C.

Chemical shifts in ^{31}P NMR spectra measured in triplicate of **$Na_3[\alpha\text{-}P^{V}W^{VI}_{12}O_{40}]$** (10 mM) dissolved D_2O and 0.1 M buffers (acetic acid – sodium acetate pH 4 – 5.5; sodium phosphate pH 3 – 8 (while phosphate does not buffer at pH range from 3.5 – 5.5, experiments were conducted at this pH to provide comparisons to previously published studies (*29*)); citric acid – sodium citrate pH 3 – 6.5; MES pH 5.5; PBS pH 7.4; tris-HCl pH 7.5 – 8.5; HEPES pH 7 – 8; MHB pH 7.4; Nutrient mixture F-12 Ham pH 7.4 and glycine-NaOH pH 8.6) and investigated after 24 h incubation at 37 °C (**Figures S13 – S21**). glycine-NaOH buffer was used in two additional concentrations of 0.2 and 0.5 M. The content of species was calculated based on the integration of ^{31}P signals considering only signals associated with POTs. Signals were assigned based on the literature data summarized in **Table S4**.

pH	$Na_3[PW_{12}O_{40}]$ (10 mM) in Solvent / Buffer / Medium	Chemical shifts δ ^{31}P [ppm]	% of PW_{12} in $Na_3[\alpha\text{-}P^{V}W^{VI}_{12}O_{40}]$ (10 mM) solution after 24 h incubation at 37 °C				% of PW_{11} in $Na_3[\alpha\text{-}P^{V}W^{VI}_{12}O_{40}]$ (10 mM) solution after 24 h incubation at 37 °C				% of P_2W_{21} in $Na_3[\alpha\text{-}P^{V}W^{VI}_{12}O_{40}]$ (10 mM) solution after 24 h incubation at 37 °C				% of P_2W_{20} in $Na_3[\alpha\text{-}P^{V}W^{VI}_{12}O_{40}]$ (10 mM) solution after 24 h incubation at 37 °C				% of P_2W_5 in $Na_3[\alpha\text{-}P^{V}W^{VI}_{12}O_{40}]$ (10 mM) solution after 24 h incubation at 37 °C				Average % of other POTs
			Sample			Mean of 1 to 3 ± SD[a]	Sample			Mean of 1 to 3 ± SD[a]	Sample			Mean of 1 to 3 ± SD[a]	Sample			Mean of 1 to 3 ± SD[a]	Sample			Mean of 1 to 3 ± SD[a]	
			#1	#2	#3		#1	#2	#3		#1	#2	#3		#1	#2	#3		#1	#2	#3		
-	D_2O	0; –12.2; –12.5; –13.0; –13.2; –13.6; –15.3	58	60	56	58 ± 2	14	16	19	16 ± 3	8	5	6	6 ± 2	0	0	0	0	0	0	0	0	19
Strongly acidic environment 3 ≤ pH ≤ 4																							
3	0.1 M Sodium phosphate ($H_2PO_4^-$ /H_3PO_4) pH 3	1.3; 0; –12.1; –12.2; –12.3; –12.4; –13.2; –13.8; –14.2; –15.3	2	5	3	3 ±2	89	91	88	89 ± 2	2	0	1	1 ± 1	0	0	0	0	0	0	0	0	7
	0.1 M Citric acid – sodium citrate (H_3Cit/H_2Cit^-) pH 3	0; –11.7; –12.6; –13.5; –13.8; –14.0; –15.3	5	5	6	5 ± 1	37	37	33	36 ± 2	2	4	4	3 ± 1	43	50	48	47 ± 4	0	0	0	0	9

4	0.1 M Sodium phosphate ($H_2PO_4^-$ /H_3PO_4) pH 4	1.3; 0; –5.2; –11.8; –12.2; –12.3; –13.3; –13.8; –14.2; –15.3	1	1	1	1 ± 0	95	93	96	95 ± 2	1	1	1	1 ± 0	0	2	0	1 ± 1	0	0	0	0	3
	0.1 M Citric acid – sodium citrate (H_2Cit^- /$HCit^{2-}$) pH 4	0; –11.8; –12.2; –14.2	0	0	0	0	39	35	38	37 ± 2	0	0	0	0	61	65	62	63 ± 2	0	0	0	0	0
	0.1 M Acetic acid – sodium acetate (OAc^- /HOAc) pH 4	0; –12.3; –13.0; –13.2; –13.3; –13.7; –15.3	10	6	5	7 ± 3	52	51	57	53 ± 3	16	12	12	13 ± 2	0	0	0	0	0	0	0	0	26
Moderately acidic environment 5 ≤ pH ≤ 6																							
5	0.1 M Sodium phosphate ($H_2PO_4^-$ /H_3PO_4) pH 5	1.3; –0.3; 0; –12.1; –12.2; –12.3; –13.3; –13.8; –14.2; –15.3	1	1	1	1 ± 0	95	92	92	93 ± 2	1	1	1	1 ± 0	1	1	1	1 ± 0	0	0	0	0	4
	0.1 M Citric acid – sodium citrate (H_2Cit^- /$HCit^{2-}$) pH 5	0; –10.9; –11.8; –14.3	0	0	0	0	96	98	91	95 ± 4	0	0	0	0	4	2	9	5 ± 3	0	0	0	0	0
	0.1 M Acetic acid – sodium acetate (OAc^- /HOAc) pH 5	0; –10.9	0	0	0	0	100	100	100	100± 0	0	0	0	0	0	0	0	0	0	0	0	0	0
5.5	0.1 M Acetic acid – sodium acetate (OAc^- /HOAc) pH 5.5	0; –10.9	0	0	0	0	100	100	100	100 ± 0	0	0	0	0	0	0	0	0	0	0	0	0	0
	0.1 M MES[b] pH 5.5	0; –11.6; –12.1; –12.2; –	1	2	0	2 ± 1	58	62	67	62 ± 5	10	12	9	10 ± 2	0	0	0	0	0	0	0	0	26

		13.0; –13.2; –13.6; –14.1; –15.2																					
6	0.1 M Sodium phosphate (HPO_4^{2-}/$H_2PO_4^{-}$) pH 6	2; 1.3; 0; –11.8; –11.9; –12.1; –12.2; –12.3; –13.3; –13.4; –13.8; –14.2	0	0	0	0	95	99	88	94 ± 6	0	0	0	0	2	1	3	2 ± 1	0	0	0	0	4
	0.1 M Citric acid – sodium citrate ($HCit^{2-}$/Cit^{3-}) pH 6	0.2; –10.8	0	0	0	0	100	100	100	100 ± 0	0	0	0	0	0	0	0	0	0	0	0	0	0
Neutral environment 6.5 ≤ pH ≤ 7.5																							
6.5	0.1 M Citric acid – sodium citrate ($HCit^{2-}$/Cit^{3-}) pH 6.5	0.3; –10.8	0	0	0	0	100	100	100	100 ± 0	0	0	0	0	0	0	0	0	0	0	0	0	0
7	0.1 M Sodium phosphate (HPO_4^{2-}/$H_2PO_4^{-}$) pH 7	3.3; –0.8; –2.4; –10.8: –11.4	0	0	0	0	30	33	37	33 ± 4	0	0	0	0	0	0	0	0	69	66	63	66 ± 3	0
	0.1 M HEPES[c] pH 7	0.2; –10.8	0	0	0	0	100	100	100	100 ± 0	0	0	0	0	0	0	0	0	0	0	0	0	0
7.4	PBS[d] pH 7.4	0; –12.2; –12.4; –13.0; –13.1; –13.2; –13.7; –15.3	12	11	14	12 ± 2	59	60	57	59 ± 2	12	11	11	11 ± 1	0	0	0	0	0	0	0	0	18
	MHB[e] pH 7.4	0.6; 0; –0.2; –11.1; –12.4; –13.3	0	0	0	0	94	90	96	93 ± 3	0	0	0	0	0	0	0	0	0	0	0	0	7

	Nutrient mixture F-12 Ham[f]	0; –12.2; –12.3; –12.9; –13.0; –13.2; –13.2; –13.6; –13.7; –15.3	10	12	8	10 ± 2	52	53	55	53 ± 2	13	12	12	11 ± 1	0	0	0	0	0	0	0	0	25
7.5	0.1 M tris-HCl[g] pH 7.5	0; –12.2; –12.3; –12.5; –12.9; –13.1; –13.2; –13.3; –13.7; –15.3	4	5	4	4 ± 1	59	59	53	57 ± 3	10	12	11	11 ± 1	0	0	0	0	0	0	0	0	28
Moderately alkaline environment 8 ≤ pH ≤ 8.6																							
8	0.1 M Sodium phosphate (HPO_4^{2-}/$H_2PO_4^-$) pH 8	3.5; 1.2; 0; –2.4; –10.8; –11.4	0	0	0	0	30	30	40	33 ± 6	0	0	0	0	0	0	0	0	70	70	60	63 ± 6	0
	0.1 M HEPES pH 8	1.46; –10.8	0	0	0	0	100	100	100	100 ± 0	0	0	0	0	0	0	0	0	0	0	0	0	0
	0.1 M tris-HCl pH 8	0; –10.9; –11.7; –12.4; –13.0; –13.3; –14.2	0	0	0	0	81	81	85	82 ± 2	0	0	0	0	5	2	1	3 ± 2	0	0	0	0	15
8.5	0.1 M tris-HCl pH 8.5	1.42; –10.9	0	0	0	0	100	100	100	100 ± 0	0	0	0	0	0	0	0	0	0	0	0	0	0
8.6	0.1 M glycine-NaOH pH 8.6	–10.8; –12.0; –12.3; –13.1; –13.3; –13.5; –13.7	0	0	0	0	62	68	64	65 ± 3	2	3	4	3 ± 1	0	0	0	0	0	0	0	0	32
	0.2 M glycine-NaOH pH 8.6	–10.8; –11.3; –13.4; –	0	0	0	0	80	89	77	82 ± 6	3	4	4	4 ± 1	0	0	0	0	0	0	0	0	14

		13.0; –13.2; **–13.7**																					
	0.5 M glycine-NaOH pH 8.6	**-10.9**; –13.2	0	0	0	0	100	100	100	100 ± 0	0	0	0	0	0	0	0	0	0	0	0	0	0

[a]SD – standard deviation; [b]MES – 2-(N-morpholino)ethanesulfonic acid, $C_6H_{13}NO_4S$ (Figure S1); [c]HEPES – 4-(2-hydroxyethyl)-1-piperazineethanesulfonic acid, $C_8H_{18}N_2O_4S$ (Figure S1); [d]PBS – phosphate buffer saline; [e]MHB – Mueller-Hinton broth, for more detailed information about composition see https://labmal.com/2019/11/20/mueller-hinton-agar-and-mueller-hinton-broth/; [f]Nutrient mixture F-12 Ham contains sodium pyruvate (0.11 g/L), phenol red, L-glutamine, and does not contain $NaHCO_3$ and HEPES, for more details please see https://www.sigmaaldrich.com/AT/en/technical-documents/technical-article/cell-culture-and-cell-culture-analysis/mammalian-cell-culture/f-12-ham; [g]tris – tris(hydroxymethyl)aminomethane, $C_4H_{11}NO_3$ (Figure S1).

5.2. The Keggin PW_{12} POT

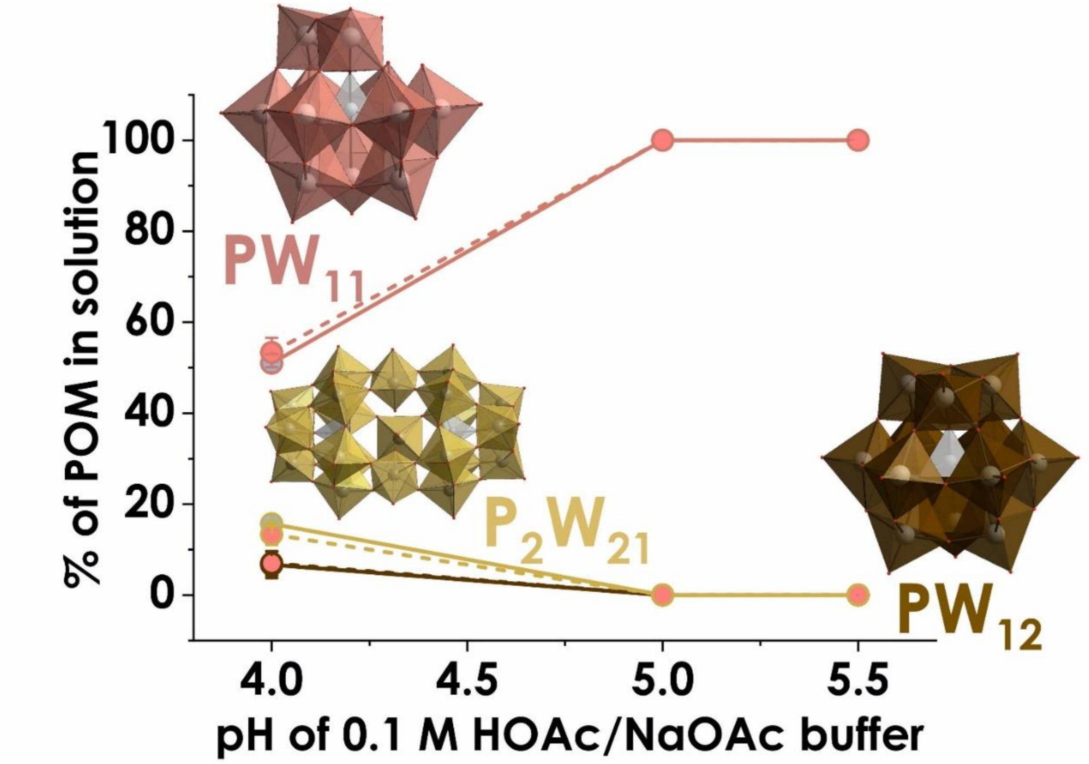

Fig. S23. Speciation of PW_{12} in acetic acid – sodium acetate buffer.

POM concentration curves in **$Na_3[\alpha\text{-}P^{V}W^{VI}_{12}O_{40}]$** (10 mM) in 0.1 M acetic acid – sodium acetate buffer solutions before (solid line, blue dot in the middle) and after incubation (dash line, red dot in the middle) for 24 h at 37 °C. The exact percentage of all POM species present is given in **Tables S6** and **S7**.

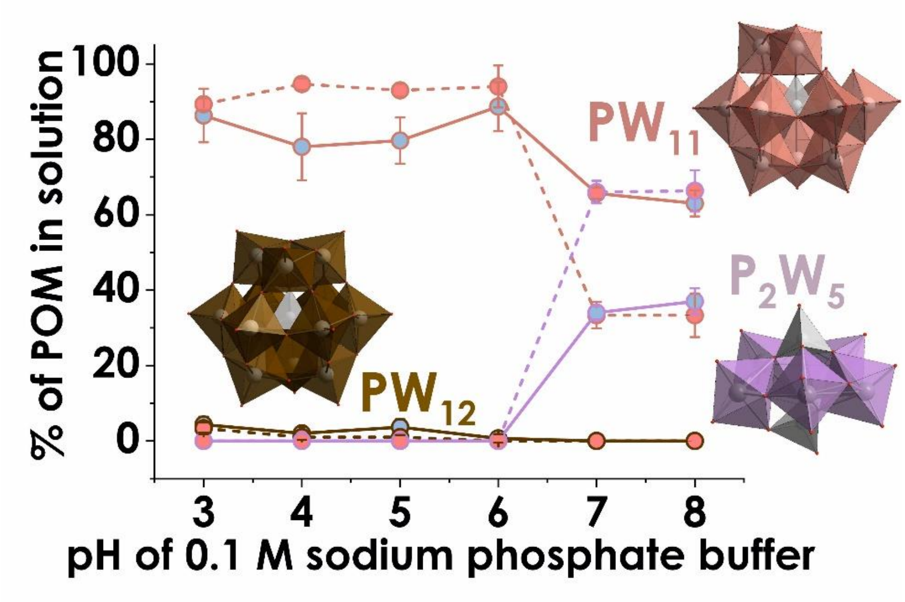

Fig. S24. Speciation of PW_{12} in sodium phosphate buffer.

POM concentration curves in **$Na_3[\alpha\text{-}P^{V}W^{VI}_{12}O_{40}]$** (10 mM) in 0.1 M sodium phosphate buffer solutions before (solid line, blue dot in the middle) and after incubation (dash line, red dot in the middle) for 24 h at 37 °C. The exact percentage of all POM species present is given in **Tables S6** and **S7**.

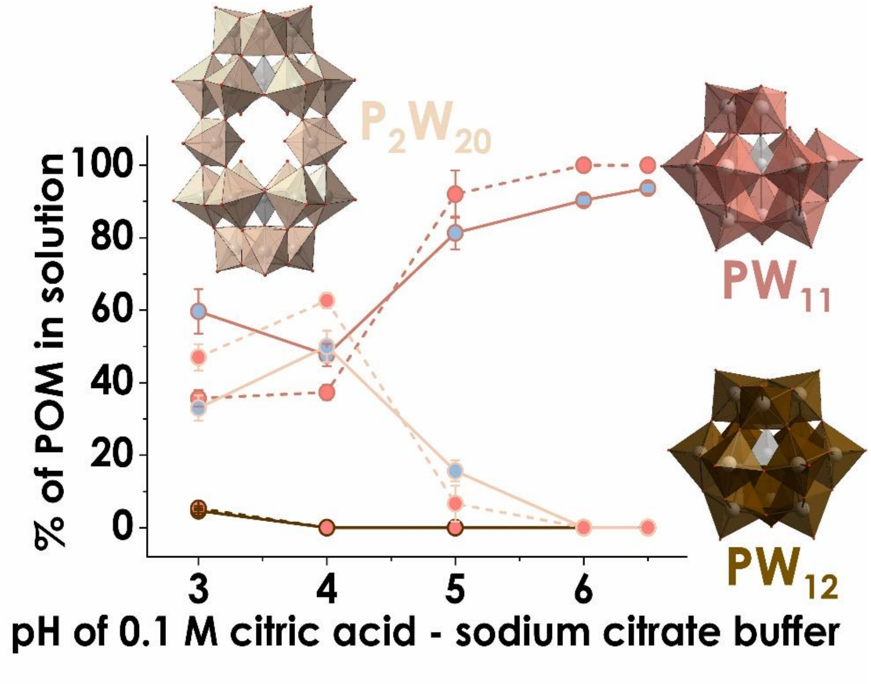

Fig. S25. Speciation of PW_{12} in citric acid – sodium citrate buffer.

POM concentration curves in **$Na_3[\alpha\text{-}P^{V}W^{VI}_{12}O_{40}]$** (10 mM) in 0.1 M citric acid – sodium citrate buffer solutions before (solid line, blue dot in the middle) and after incubation (dash line, red dot in the middle) for 24 h at 37 °C. The exact percentage of all POM species present is given in **Tables S6** and **S7**.

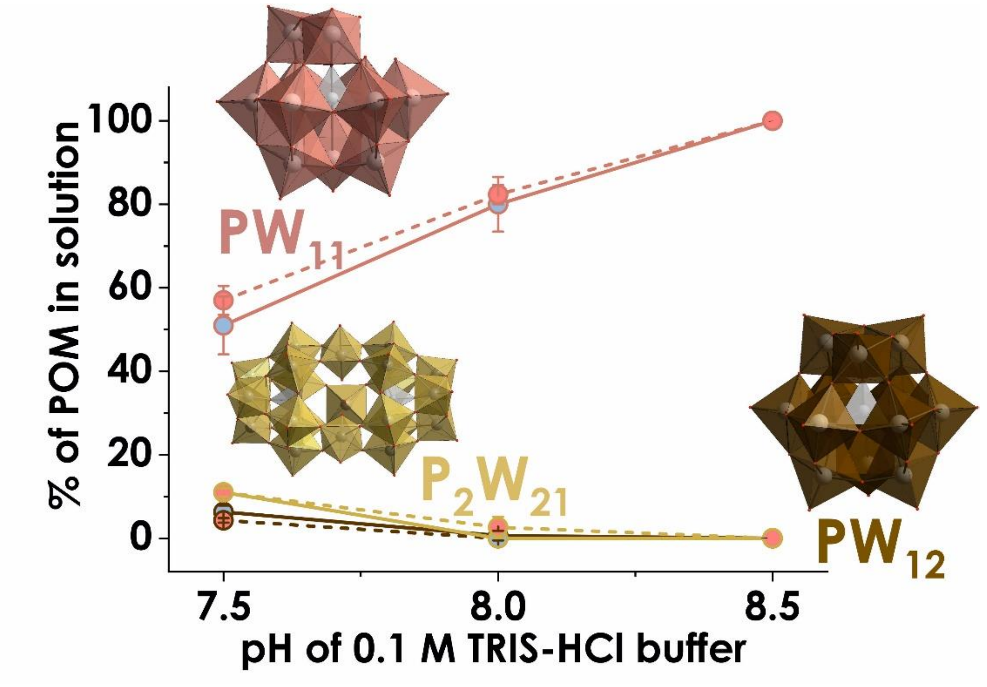

Fig. S26. Speciation of PW12 in tris-HCl buffer.

POM concentration curves in **$Na_3[\alpha\text{-}P^{V}W^{VI}_{12}O_{40}]$** (10 mM) in 0.1 M tris-HCl buffer solutions before (solid line, blue dot in the middle) and after incubation (dash line, red dot in the middle) for 24 h at 37 °C. The exact percentage of all POM species present is given in **Tables S6** and **S7**.

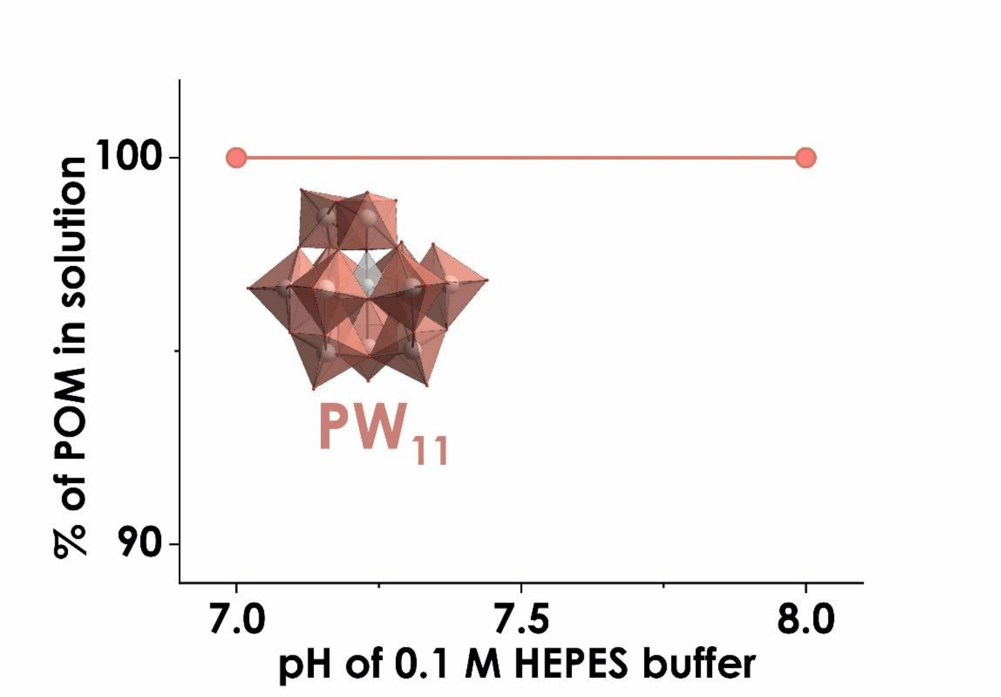

Fig. S27. Speciation of PW_{12} in HEPES buffer.

POM concentration curves in **$Na_3[\alpha\text{-}P^{V}W^{VI}_{12}O_{40}]$** (10 mM) in 0.1 M HEPES buffer solutions before (solid line, blue dot in the middle) and after incubation (dash line, red dot in the middle) for 24 h at 37 °C. The exact percentage of all POM species present is given in **Tables S6** and **S7**.

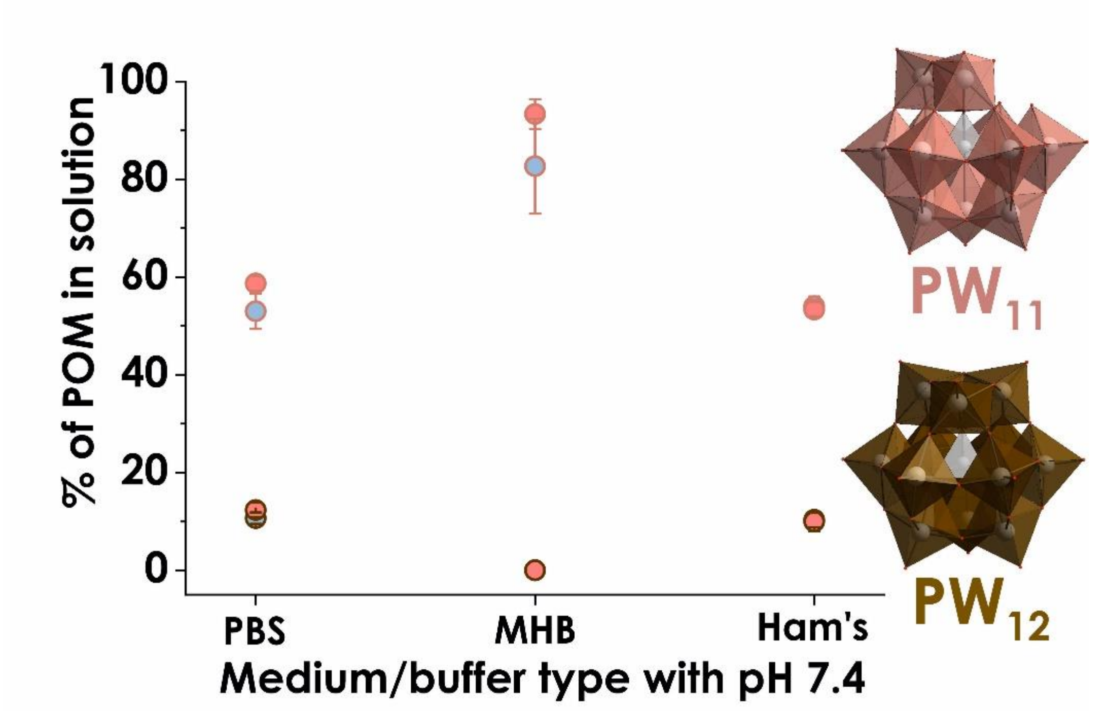

Fig. S28. Speciation of PW_{12} in solutions with pH 7.4.

POM concentration curves in **$Na_3[\alpha\text{-}P^{V}W^{VI}_{12}O_{40}]$** (10 mM) in 0.1 M PBS, MHB, and nutrient mixture F-12 Ham solutions before (blue dot in the middle) and after incubation (red dot in the middle) for 24 h at 37 °C. The exact percentage of all POM species present is given in **Tables S6** and **S7**.

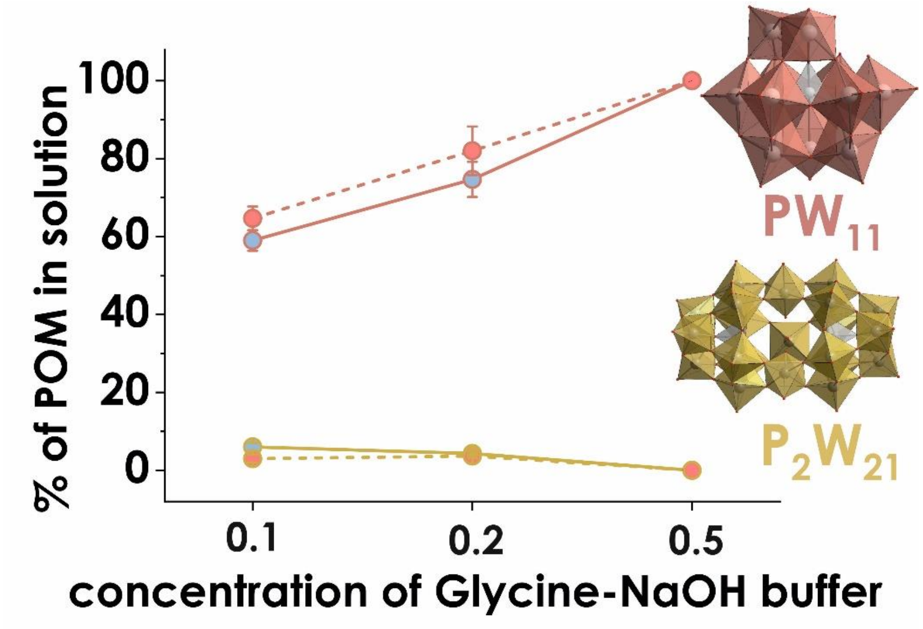

Fig. S29. Speciation of PW_{12} in glycine-NaOH buffer.

POM concentration curves in **$Na_3[\alpha\text{-}P^{V}W^{VI}_{12}O_{40}]$** (10 mM) in glycine-NaOH (pH 8.6) with concentrations of 0.1. 0.2 and 0.5 M solutions before (solid line, blue dot in the middle) and after incubation (dash line, red dot in the middle) for 24 h at 37 °C. The exact percentage of all POM species present is given in **Tables S6** and **S7**.

6. The SiW_{12} Keggin POT

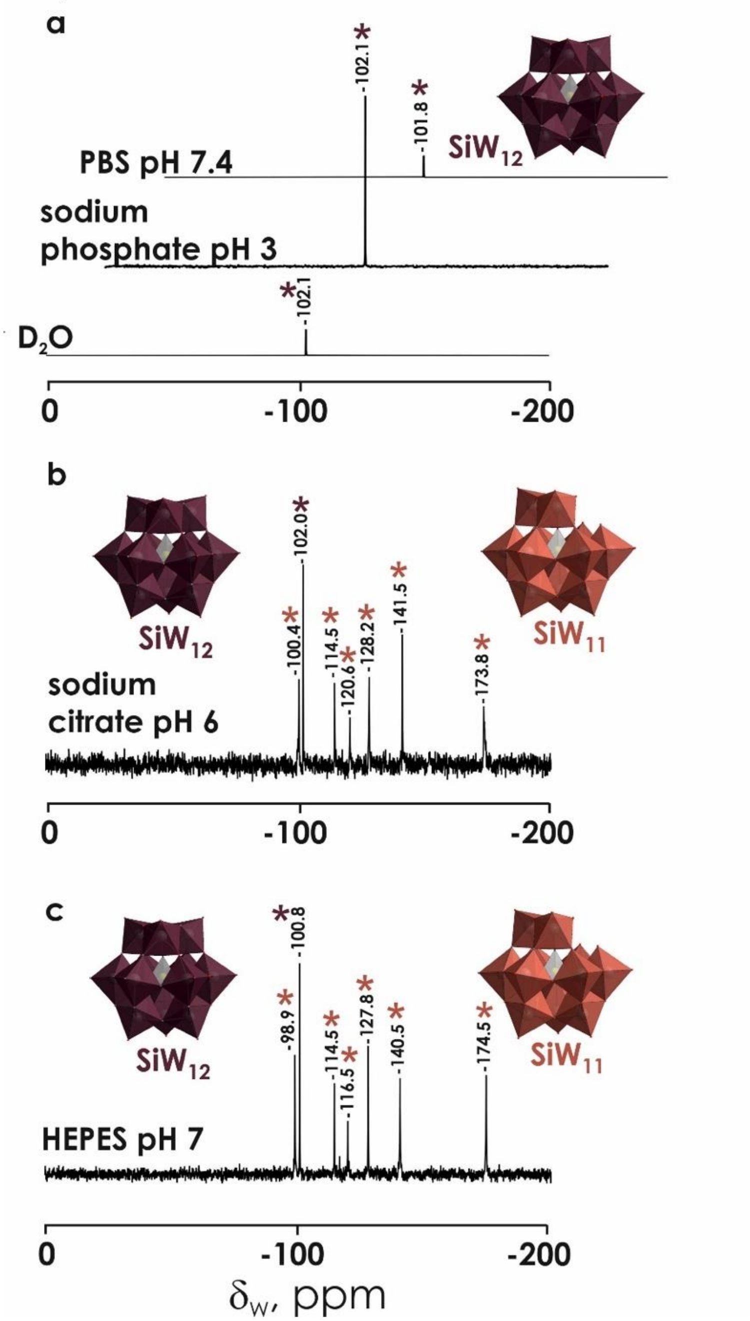

Fig. S30. ^{183}W NMR spectra of SiW_{12} solutions.

^{183}W NMR spectra for 20 mM solutions of **$K_4[Si^{IV}W^{VI}_{12}O_{40}]$** in (**a**) D_2O (pH after POM dissolving is 5.4), 0.2 M sodium phosphate pH 3 (pH 2.85 after POM dissolution), and PBS pH 7.4 (pH 7.1 after POM dissolving); (**b**) in 0.2 M sodium citrate pH 6 (pH 5.7 after POM dissolving); (**c**) in 0.2 M HEPES pH 7 (pH 6.3 after POM dissolution). The chemical shift around –102 ppm corresponds to twelve equivalent W ions in $[Si^{IV}W^{VI}_{12}O_{40}]^{4-}$, and six signals in the range from –98 to –175 ppm arose? from six ions types in monolacunary $[Si^{IV}W^{VI}_{11}O_{39}]^{8-}$ (**Table S4**). Color code: $\{WO_6\}$, burgundy or pink-arange; $\{SiO_4\}$, grey; O, red.

7. The Wells-Dawson α-P_2W_{18} and α/β-P_2W_{18} POTs

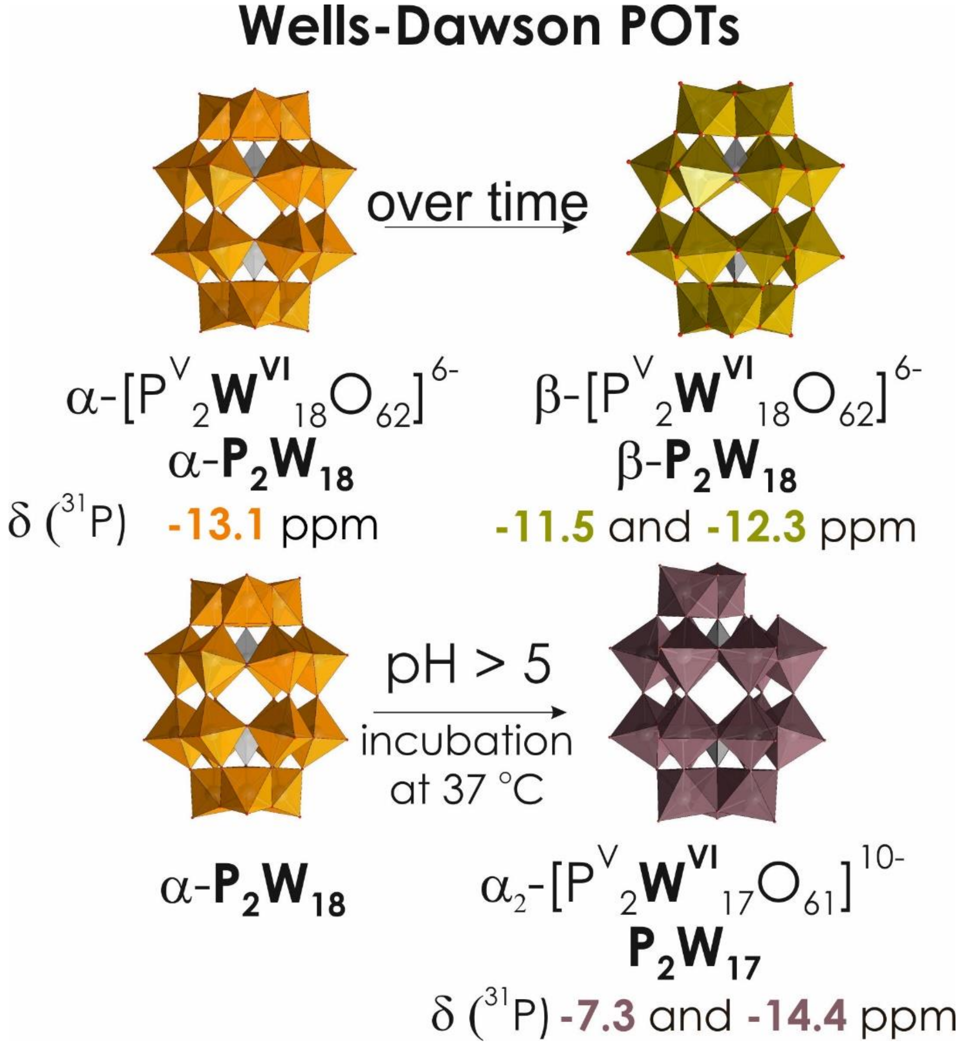

Fig. S31. Wells-Dawson POTs.

Structures of two Wells-Dawson POT isomers $[\alpha\text{-}P^{V}_2W^{VI}_{18}O_{62}]^{6-}$ (***α*-P₂W₁₈**) and $[\beta\text{-}P^{V}_2W^{VI}_{18}O_{62}]^{6-}$ (***β*-P₂W₁₈**) with indication of ^{31}P NMR chemical shifts. The two main transformations that take place in aqueous solutions: 1) hydrolysis of intact Wells-Dawson POT (shown on the example of $[\alpha\text{-}P^{V}_2W^{VI}_{18}O_{62}]^{6-}$ isomer) to the monolacunary anion $[\alpha_2\text{-}P^{V}_2W^{VI}_{17}O_{61}]^{10-}$ (**P_2W_{17}**); 2) rearrangement of $[\beta\text{-}P^{V}_2W^{VI}_{18}O_{62}]^{6-}$ to $[\alpha\text{-}P_2W_{18}O_{62}]^{6-}$ over time in $(NH_4)_6[\alpha/\beta\text{-}P^{V}_2W^{VI}_{18}O_{62}]$ (***α/β*-P_2W_{18}**) solutions. Color code: $\{WO_6\}$, orange, yellow-green and taupe; $\{PO_4\}$, grey; O, red. To identify the individual anions, they are shown in different colors, with the same color code being selected for a specific anion throughout all figures and tables in the main manuscript and the supporting information.

7.1. pH of Wells-Dawson POTs solutions

Table S8. pH in *α*-P_2W_{18} solutions.

pH values measured in triplicate of **$K_6[\alpha\text{-}P^{V}_2W^{VI}_{18}O_{62}]$** (10 mM) dissolved in D_2O and different buffers (acetic acid – sodium acetate pH 4 – 5.5; sodium phosphate pH 3 – 8 (while phosphate does not buffer at pH range from 3.5 – 5.5, experiments were conducted at this pH to provide comparisons to previously published studies (*29*)); citric acid – sodium citrate pH 3 – 6.5; MES pH 5.5; PBS pH 7.4; tris-HCl pH 7.5 – 8.5; HEPES pH 7 – 8; MHB pH 7.4; Nutrient mixture F-12 Ham pH 7.4 and glycine-NaOH pH 8.6) with concentration 0.1 M. The glycine-NaOH buffer was used at two additional concentrations of 0.2 and 0.5 M.

**pH**	**$K_6[\alpha\text{-}P_2W_{18}O_{62}]$** (10 mM) in Solvent / Buffer / Medium	pH after dissolving **$K_6[\alpha\text{-}P^{V}_2W^{VI}_{18}O_{62}]$** (10 mM) at RT				pH after 24 h incubation of **$K_6[\alpha\text{-}P^{V}_2W^{VI}_{18}O_{62}]$** (10 mM) solution at 37 °C			
		Sample			Mean of 1 to 3 ± SD[a]	Sample			Mean of 1 to 3 ± SD[a]
		#1	#2	#3		#1	#2	#3	
-	D_2O	5.87	5.94	6.03	5.94 ± 0.08	4.63	4.57	4.48	4.56 ± 0.08
Strongly acidic environment 3 ≤ pH ≤ 4									
3	0.1 M Sodium phosphate ($H_2PO_4^-/H_3PO_4$) pH 3	2.93	2.96	3.08	2.99 ± 0.08	3.05	2.95	2.95	2.98 ± 0.06
	0.1 M Citric acid – sodium citrate (H_3Cit/H_2Cit^-) pH 3	2.90	2.84	2.85	2.86 ± 0.03	2.82	2.80	2.70	2.77 ± 0.06
4	0.1 M Sodium phosphate ($H_2PO_4^-/H_3PO_4$) pH 4	4.00	3.94	4.05	4.00 ± 0.06	4.03	3.92	3.95	3.97 ± 0.06
	0.1 M Citric acid – sodium citrate ($H_2Cit^-/HCit^{2-}$) pH 4	3.90	3.89	3.88	3.89 ± 0.01	3.81	3.82	3.80	3.81 ± 0.02
	0.1 M Acetic acid – sodium acetate ($OAc^-/HOAc$) pH 4	3.93	3.98	4.00	3.97 ± 0.04	4.02	3.92	4.00	3.98 ± 0.05
Moderately acidic environment 5 ≤ pH ≤ 6									
5	0.1 M Sodium phosphate ($H_2PO_4^-/H_3PO_4$) pH 5	4.97	4.85	4.99	4.94 ± 0.08	4.78	4.65	4.68	4.70 ± 0.07
	0.1 M Citric acid – sodium citrate ($H_2Cit^-/HCit^{2-}$) pH 5	5.00	4.91	4.91	4.94 ± 0.05	4.68	4.80	4.68	4.72 ± 0.07
	0.1 M Acetic acid – sodium acetate ($OAc^-/HOAc$) pH 5	4.97	4.96	4.99	4.98 ± 0.02	5.00	4.94	5.00	4.98 ± 0.03

5.5	0.1 M Acetic acid – sodium acetate (OAc^-/HOAc) pH 5.5	5.40	5.48	5.51	5.46 ± 0.06	5.27	5.20	5.30	5.26 ± 0.05
	0.1 M MES[b] pH 5.5	5.25	4.91	5.16	5.10 ± 0.18	4.53	4.41	4.43	4.45 ± 0.06
6	0.1 M Sodium phosphate (HPO_4^{2-}/$H_2PO_4^-$) pH 6	5.94	5.84	5.93	5.90 ± 0.06	5.01	5.00	4.97	4.99 ± 0.02
	0.1 M Citric acid – sodium citrate ($HCit^{2-}$/Cit^{3-}) pH 6	6.00	5.91	5.93	5.95 ± 0.05	5.50	5.45	5.45	5.47 ± 0.03
Neutral environment 6.5 ≤ pH ≤ 7.5									
6.5	0.1 M Citric acid – sodium citrate ($HCit^{2-}$/Cit^{3-}) pH 6.5	6.39	6.60	6.45	6.48 ± 0.11	5.63	5.80	5.71	5.71 ± 0.08
7	0.1 M Sodium phosphate (HPO_4^{2-}/$H_2PO_4^-$) pH 7	6.89	6.73	6.88	6.83 ± 0.09	6.58	6.53	6.54	6.55 ± 0.03
	0.1 M HEPES[c] pH 7	6.63	6.20	6.44	6.42 ± 0.22	5.91	5.84	5.90	5.88 ± 0.04
7.4	PBS[d] pH 7.4	6.82	6.64	6.77	6.74 ± 0.09	4.74	4.78	4.82	4.78 ± 0.04
	MHB[e] pH 7.4	5.98	5.94	6.06	5.99 ± 0.06	5.18	5.19	5.17	5.18 ± 0.01
	Nutrient mixture F-12 Ham[f]	6.58	6.57	6.56	6.56 ±0.01	4.76	4.81	4.80	4.79 ± 0.02
7.5	0.1 M tris-HCl[g] pH 7.5	5.50	5.50	5.64	5.55 ± 0.08	5.49	5.47	5.50	5.49 ± 0.02
Moderately alkaline environment 8 ≤ pH ≤ 8.6									
8	0.1 M Sodium phosphate (HPO_4^{2-}/$H_2PO_4^-$) pH 8	7.20	7.06	7.25	7.17 ± 0.10	6.76	6.83	7.20	6.93 ± 0.24
	0.1 M HEPES pH 8	7.18	7.00	7.25	7.14 ± 0.13	7.08	7.04	7.10	7.07 ± 0.03
	0.1 M tris-HCl pH 8	5.60	5.82	6.12	5.85 ± 0.26	5.62	5.64	5.70	5.65 ± 0.04
8.5	0.1 M tris-HCl pH 8.5	6.40	6.89	6.91	6.73 ± 0.29	6.17	6.89	6.60	6.55 ± 0.36
8.6	0.1 M glycine-NaOH pH 8.6	5.50	5.84	5.99	5.78 ± 0.25	5.35	5.38	5.40	5.38 ± 0.03
	0.2 M glycine-NaOH pH 8.6	5.61	6.12	6.15	5.96 ± 0.31	5.45	5.51	5.59	5.52 ± 0.07
	0.5 M glycine-NaOH pH 8.6	5.70	6.23	6.36	6.10 ± 0.35	5.48	5.34	5.47	5.43 ± 0.07

[a]SD – standard deviation; [b]MES – 2-(N-morpholino)ethanesulfonic acid, $C_6H_{13}NO_4S$ (Figure S1); [c]HEPES – 4-(2-hydroxyethyl)-1-piperazineethanesulfonic acid, $C_8H_{18}N_2O_4S$ (Figure S1); [d]PBS – phosphate buffer saline; [e]MHB – Mueller-Hinton broth, for more detailed information about composition see https://labmal.com/2019/11/20/mueller-hinton-agar-and-mueller-hinton-broth/; [f]Nutrient mixture F-12 Ham contains sodium pyruvate (0.11 g/L), phenol red, L-glutamine, and does not contain $NaHCO_3$ and HEPES, for more details please see https://www.sigmaaldrich.com/AT/en/technical-documents/technical-article/cell-culture-and-cell-culture-analysis/mammalian-cell-culture/F-12-ham; [g]tris – tris(hydroxymethyl)aminomethane, $C_4H_{11}NO_3$ (Figure S1).

Table S9. pH in *α/β* -P_2W_{18} solutions.

pH values measured in triplicate of **$(NH_4)_6$[*α/β*-$P^{V}_2W^{VI}_{18}O_{62}$]** (10 mM) dissolved in D_2O and different buffers (acetic acid – sodium acetate pH 4 – 5.5; sodium phosphate pH 3 – 8 (while phosphate does not buffer at pH range from 3.5 – 5.5, experiments were conducted at this pH to provide comparisons to previously published studies (*29*)); citric acid – sodium citrate pH 3 – 6.5; MES pH 5.5; PBS pH 7.4; tris-HCl pH 7.5 – 8.5; HEPES pH 7 – 8; MHB pH 7.4; Nutrient mixture F-12 Ham pH 7.4 and glycine-NaOH pH 8.6) with concentration 0.1 M. Glycine-NaOH buffer was used in two additional concentrations 0.2 and 0.5 M.

**pH**	**$(NH_4)_6$[*α/β*-$P_2W_{18}O_{62}$]** (10 mM) in Solvent / Buffer / Medium	pH after dissolving **$(NH_4)_6$[*α/β*-$P^{V}_2W^{VI}_{18}O_{62}$]** (10 mM) at RT				pH after 24 h incubation of **$(NH_4)_6$[*α/β*-$P^{V}_2W^{VI}_{18}O_{62}$]** (10 mM) solution at 37 °C			
		Sample			Mean of 1 to 3 ± SD[a]	Sample			Mean of 1 to 3 ± SD[a]
		#1	#2	#3		#1	#2	#3	
-	D_2O	2.71	2.57	2.53	2.61 ± 0.11	2.59	2.72	2.52	2.61 ± 0.10
Strongly acidic environment 3 ≤ pH ≤ 4									
3	0.1 M Sodium phosphate ($H_2PO_4^-/H_3PO_4$) pH 3	2.88	2.89	2.88	2.88 ± 0.01	2.86	2.82	2.87	2.85 ± 0.03
	0.1 M Citric acid – sodium citrate (H_3Cit/H_2Cit^-) pH 3	2.84	2.87	2.81	2.84 ± 0.03	2.81	2.79	2.78	2.79 ± 0.02
4	0.1 M Sodium phosphate ($H_2PO_4^-/H_3PO_4$) pH 4	3.54	3.64	3.42	3.53 ± 0.11	3.52	3.59	3.41	3.51 ± 0.09
	0.1 M Citric acid – sodium citrate ($H_2Cit^-/HCit^{2-}$) pH 4	3.85	3.87	3.83	3.85 ± 0.02	3.80	3.81	3.78	3.80 ± 0.02
	0.1 M Acetic acid – sodium acetate (OAc^-/HOAc) pH 4	3.83	3.92	3.88	3.88 ± 0.05	3.79	3.83	3.89	3.84 ± 0.05
Moderately acidic environment 5 ≤ pH ≤ 6									
5	0.1 M Sodium phosphate ($H_2PO_4^-/H_3PO_4$) pH 5	4.00	4.34	4.02	4.12 ± 0.19	4.00	4.28	4.01	4.10 ± 0.16
	0.1 M Citric acid – sodium citrate ($H_2Cit^-/HCit^{2-}$) pH 5	4.83	4.88	4.87	4.86 ± 0.03	4.56	4.61	4.54	4.57 ± 0.04
	0.1 M Acetic acid – sodium acetate (OAc^-/HOAc) pH 5	4.96	4.89	4.91	4.92 ± 0.04	4.85	4.86	4.85	4.85 ± 0.01
5.5	0.1 M Acetic acid – sodium acetate (OAc^-/HOAc) pH 5.5	5.41	5.43	5.40	5.41 ± 0.02	5.04	5.06	5.09	5.06 ± 0.03
	0.1 M MES[b] pH 5.5	5.24	4.71	5.32	5.09 ± 0.33	4.44	4.19	4.20	4.28 ± 0.14

6	0.1 M Sodium phosphate ($HPO_4^{2–}/H_2PO_4^–$) pH 6	5.78	5.85	5.71	5.78 ± 0.07	4.94	4.94	4.91	4.93 ± 0.02
	0.1 M Citric acid – sodium citrate ($HCit^{2–}/Cit^{3–}$) pH 6	5.83	5.83	5.95	5.87 ± 0.07	5.25	5.36	5.52	5.38 ± 0.14
Neutral environment 6.5 ≤ pH ≤ 7.5									
6.5	0.1 M Citric acid – sodium citrate ($HCit^{2–}/Cit^{3–}$) pH 6.5	6.27	6.02	6.23	6.17 ± 0.13	5.51	5.55	5.57	5.54 ± 0.03
7	0.1 M Sodium phosphate ($HPO_4^{2–}/H_2PO_4^–$) pH 7	6.78	6.82	6.76	6.79 ± 0.03	6.52	6.41	6.43	6.45 ± 0.06
	0.1 M HEPES[c] pH 7	6.59	6.60	6.56	6.58 ± 0.02	5.61	5.67	5.54	5.61 ± 0.07
7.4	PBS[d] pH 7.4	5.87	5.48	5.79	5.71 ± 0.21	4.71	4.71	4.72	4.71 ± 0.01
	MHB[e] pH 7.4	6.19	5.45	5.95	5.86 ± 0.37	5.10	5.08	5.13	5.10 ± 0.03
	Nutrient mixture F-12 Ham[f]	5.93	5.81	5.86	5.87 ± 0.38	4.76	4.71	4.76	4.74 ± 0.03
7.5	0.1 M tris-HCl[g] pH 7.5	5.95	6.10	6.05	6.03 ± 0.08	5.23	5.26	5.24	5.24 ± 0.02
Moderately alkaline environment 8 ≤ pH ≤ 8.6									
8	0.1 M Sodium phosphate ($HPO_4^{2–}/H_2PO_4^–$) pH 8	6.94	7.07	6.98	7.00 ± 0.07	6.68	6.73	6.75	6.72 ± 0.03
	0.1 M HEPES pH 8	7.12	7.21	7.06	7.13 ± 0.08	7.01	7.00	7.01	7.01 ± 0.01
	0.1 M tris-HCl pH 8	6.35	6.40	6.40	6.38 ± 0.03	5.60	5.51	5.48	5.53 ± 0.06
8.5	0.1 M tris-HCl pH 8.5	6.87	6.95	6.94	6.92 ± 0.04	6.55	6.78	6.59	6.64 ± 0.12
8.6	0.1 M glycine-NaOH pH 8.6	5.94	5.22	5.54	5.57 ± 0.36	5.16	5.11	5.12	5.13 ± 0.03
	0.2 M glycine-NaOH pH 8.6	6.31	5.65	6.02	5.99 ± 0.33	5.30	5.37	5.25	5.31 ± 0.06
	0.5 M glycine-NaOH pH 8.6	6.55	6.06	6.19	6.27 ± 0.25	5.31	5.38	5.30	5.33 ± 0.04

[a]SD – standard deviation; [b]MES – 2-(N-morpholino)ethanesulfonic acid, $C_6H_{13}NO_4S$ (Figure S1); [c]HEPES – 4-(2-hydroxyethyl)-1-piperazineethanesulfonic acid, $C_8H_{18}N_2O_4S$ (Figure S1); [d]PBS – phosphate buffer saline; [e]MHB – Mueller-Hinton broth, for more detailed information about composition see https://labmal.com/2019/11/20/mueller-hinton-agar-and-mueller-hinton-broth/; [f]Nutrient mixture F-12 Ham contains sodium pyruvate (0.11 g/L), phenol red, L-glutamine, and does not contain $NaHCO_3$ and HEPES, for more details please see https://www.sigmaaldrich.com/AT/en/technical-documents/technical-article/cell-culture-and-cell-culture-analysis/mammalian-cell-culture/F-12-ham; [g]tris – tris(hydroxymethyl)aminomethane, $C_4H_{11}NO_3$ (Figure S1).

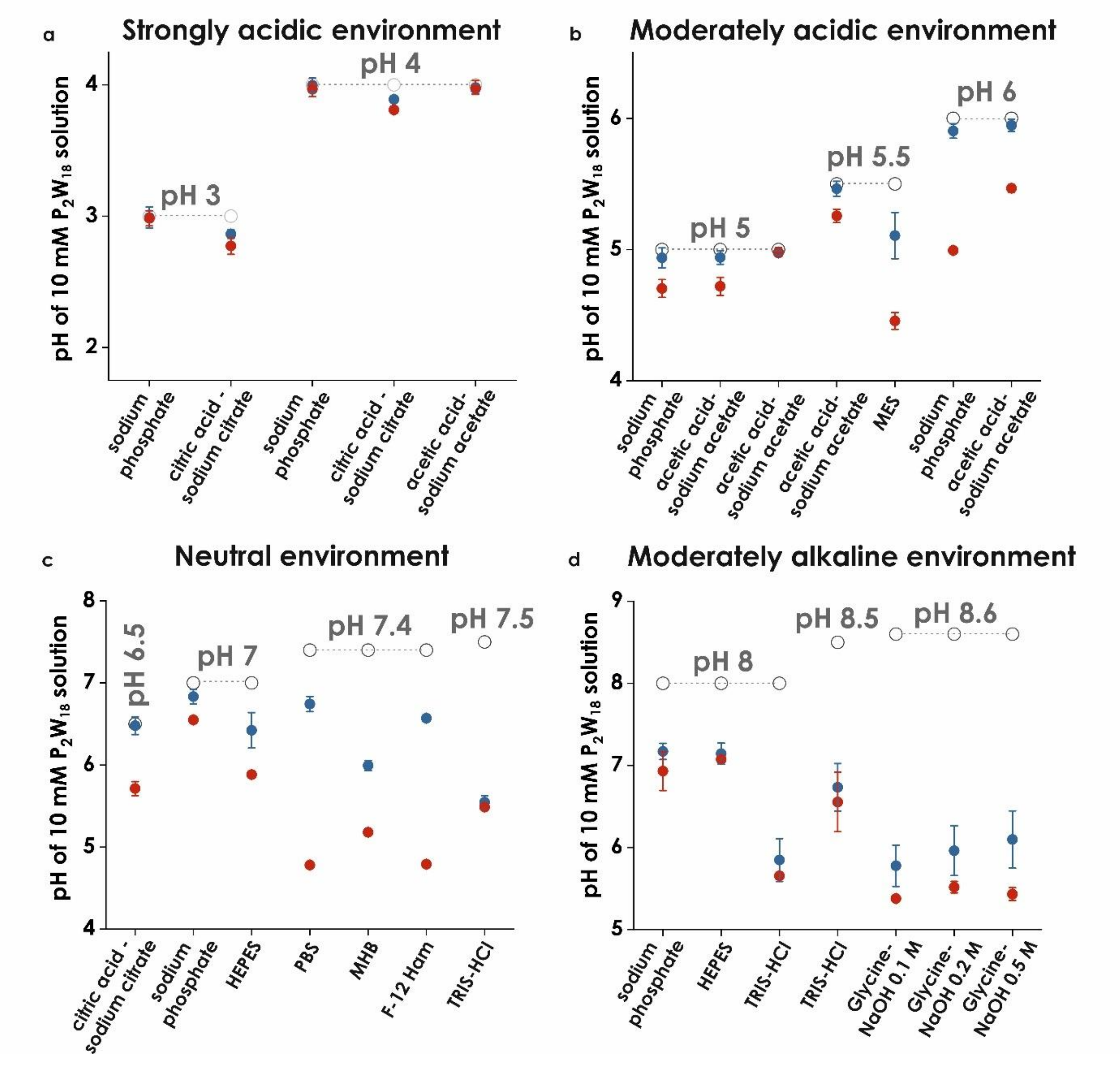

Fig. S32. pH in α-P_2W_{18} solutions.

The mean of pH values measured in triplicates for 10 mM solutions of **$K_6[\alpha\text{-}P^{V}_2W^{VI}_{18}O_{62}]$** in different buffers in a) strongly acidic, b) moderately acidic, c) neutral, and d) moderately alkaline environments. The pH of the starting buffers is shown with a gray dashed line; the plots of the measured pH values immediately after the preparation of the solutions are shown in blue and after 24 h incubation at 37 °C in red. The error bar shows standard deviation (**Table S8**).

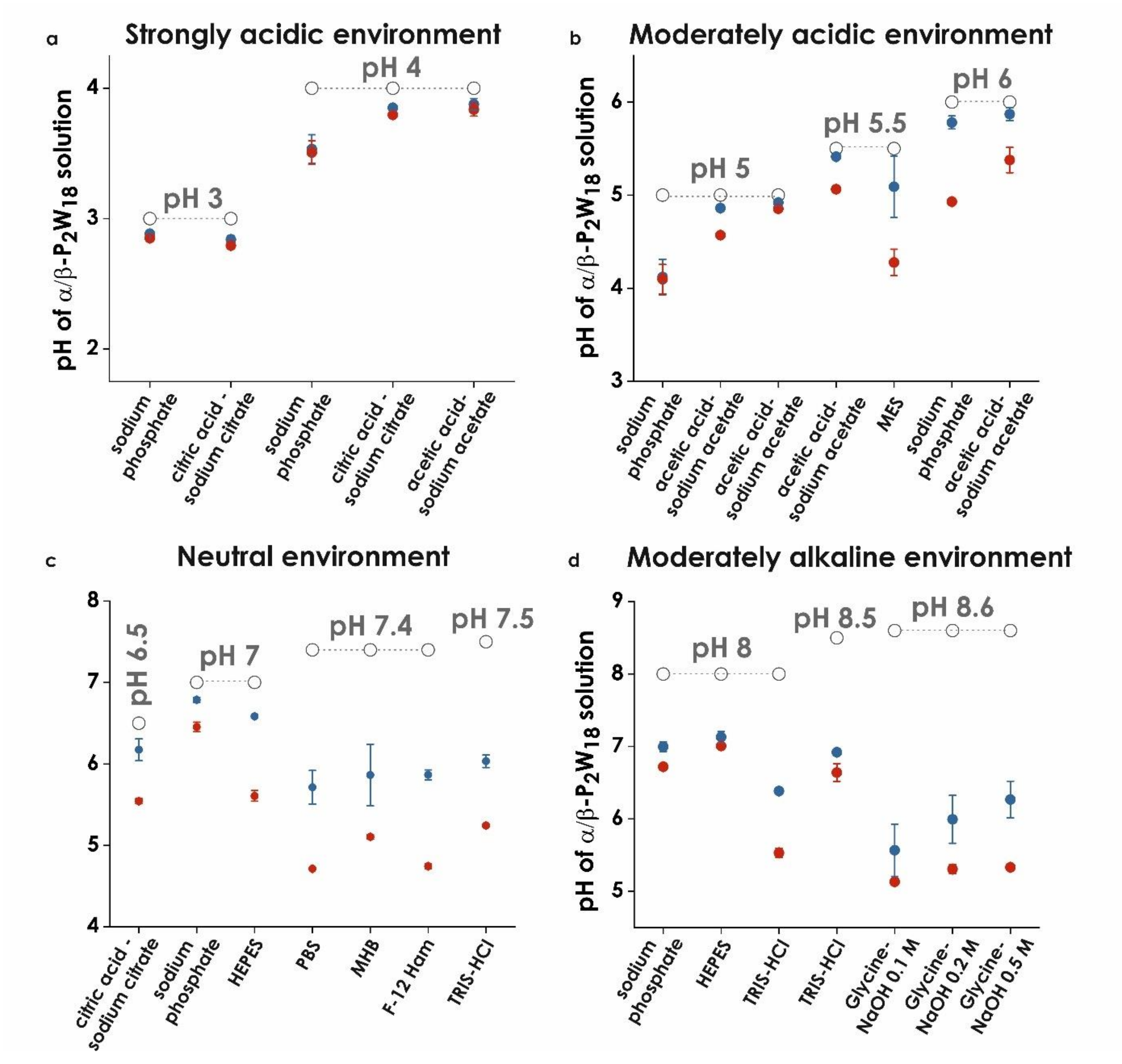

Fig. S33. pH in *α/β*-P_2W_{18} solutions.

The mean of pH values measured in triplicates for 10 mM solutions of **$(NH_4)_6$[*α/β*-$P^V_2W^{VI}_{18}O_{62}$]** in different buffers in a) strongly acidic, b) moderately acidic, c) neutral, and d) moderately alkaline environments. The pH of the starting buffers is shown with a gray dashed line; the plots of the measured pH values immediately after the preparation of the solutions are shown in blue and after 24 h incubation at 37 °C in red. The error bar shows standard deviation (**Table S9**).

7.2. ^{31}P and ^{183}W NMR spectroscopic studies of Wells-Dawson POT $K_6[\alpha\text{-}P^{V}_2W^{VI}_{18}O_{62}]$ solutions

All ^{31}P and ^{183}W peaks with the highest intensity were unambiguously assigned based on the literature data from **Table S4**. In some ^{31}P spectra, chemical shifts for peaks of lower intensity have not yet been described in the literature and therefore can not be assigned in this work.

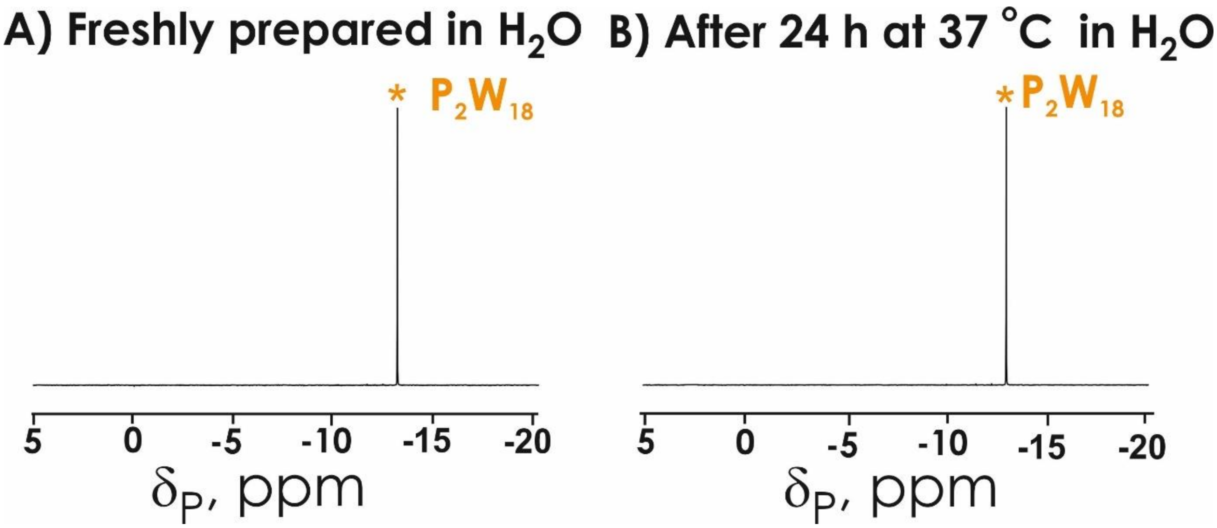

Fig. S34. ^{31}P NMR spectra of α-P_2W_{18} in H_2O.

^{31}P NMR spectra for 10 mM solutions of $\mathbf{K_6[\alpha\text{-}P^{V}_2W^{VI}_{18}O_{62}]}$ in H_2O that were recorded approximately one hour after preparation (**A**) and after incubation for 24 h at 37 °C (**B**). The structures of all POMs are shown in **Figure S31**. The chemical shifts and percentages of parent and formed species are given in **Tables S10** and **S11**. To identify the individual anions, they are shown in different colors, with the same color code being selected for a specific anion throughout all figures and tables in the main manuscript and the supporting information.

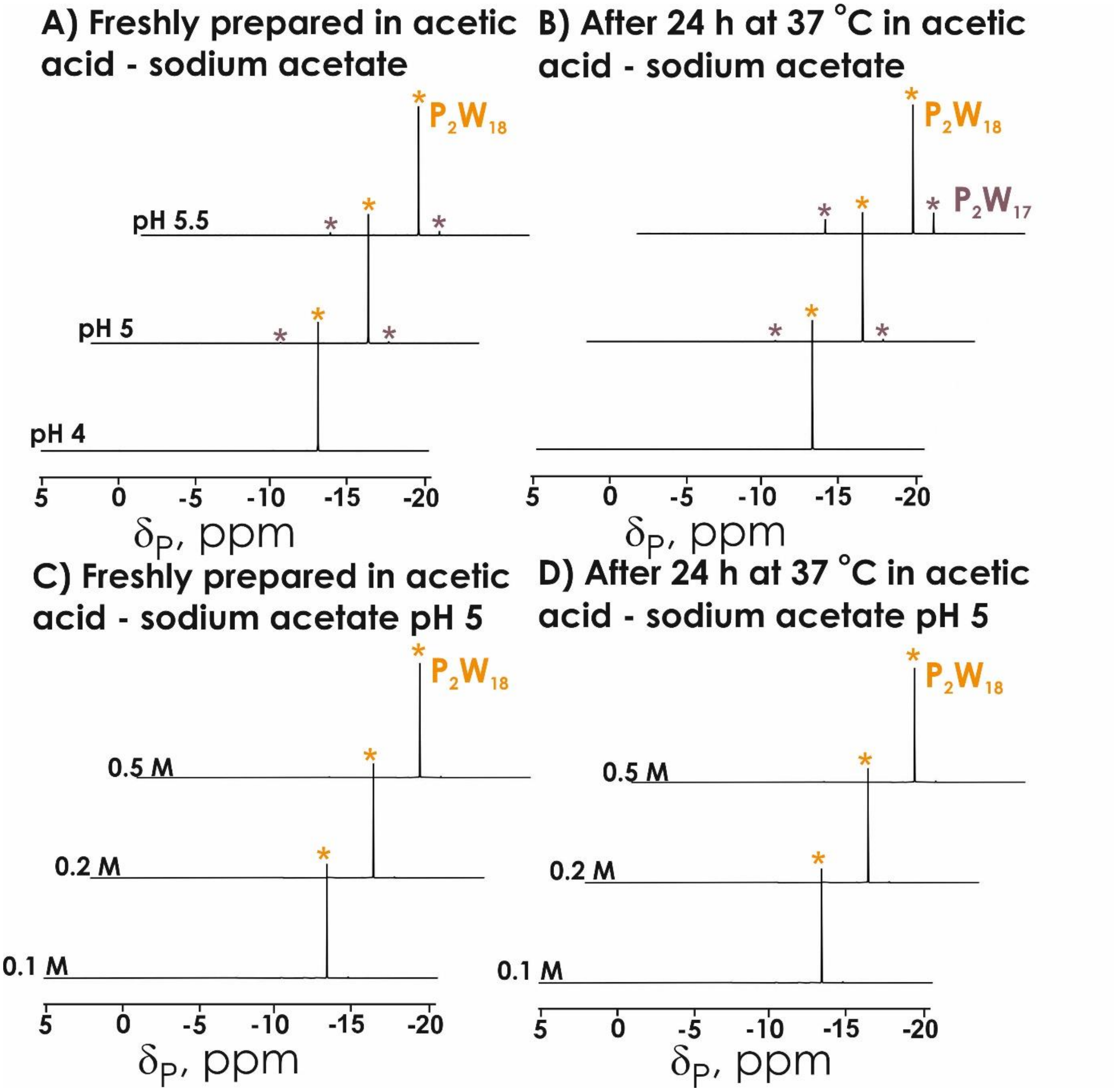

Fig. S35. ^{31}P NMR spectra of α-P_2W_{18} in NaOAc/HOAc buffer.

^{31}P NMR spectra for 10 mM solutions of **$K_6[\alpha\text{-}P^{V}_2W^{VI}_{18}O_{62}]$** in 0.1 M NaOAc/HOAc buffer (pH 4 – 5.5) that were recorded approximately one hour after preparation (**A**) and after incubation for 24 h at 37 °C (**B**). The spectra recorded in 0.1, 0.2, and 0.5 M NaOAc/HOAc buffer pH 5 are shown in **C** and **D**. The structures of all POMs are shown in **Figure S31**. The chemical shifts and percentages of parent and formed species are given in **Tables S10** and **S11**. To identify the individual anions, they are shown in different colors, with the same color code being selected for a specific anion throughout all figures and tables in the main manuscript and the supporting information.

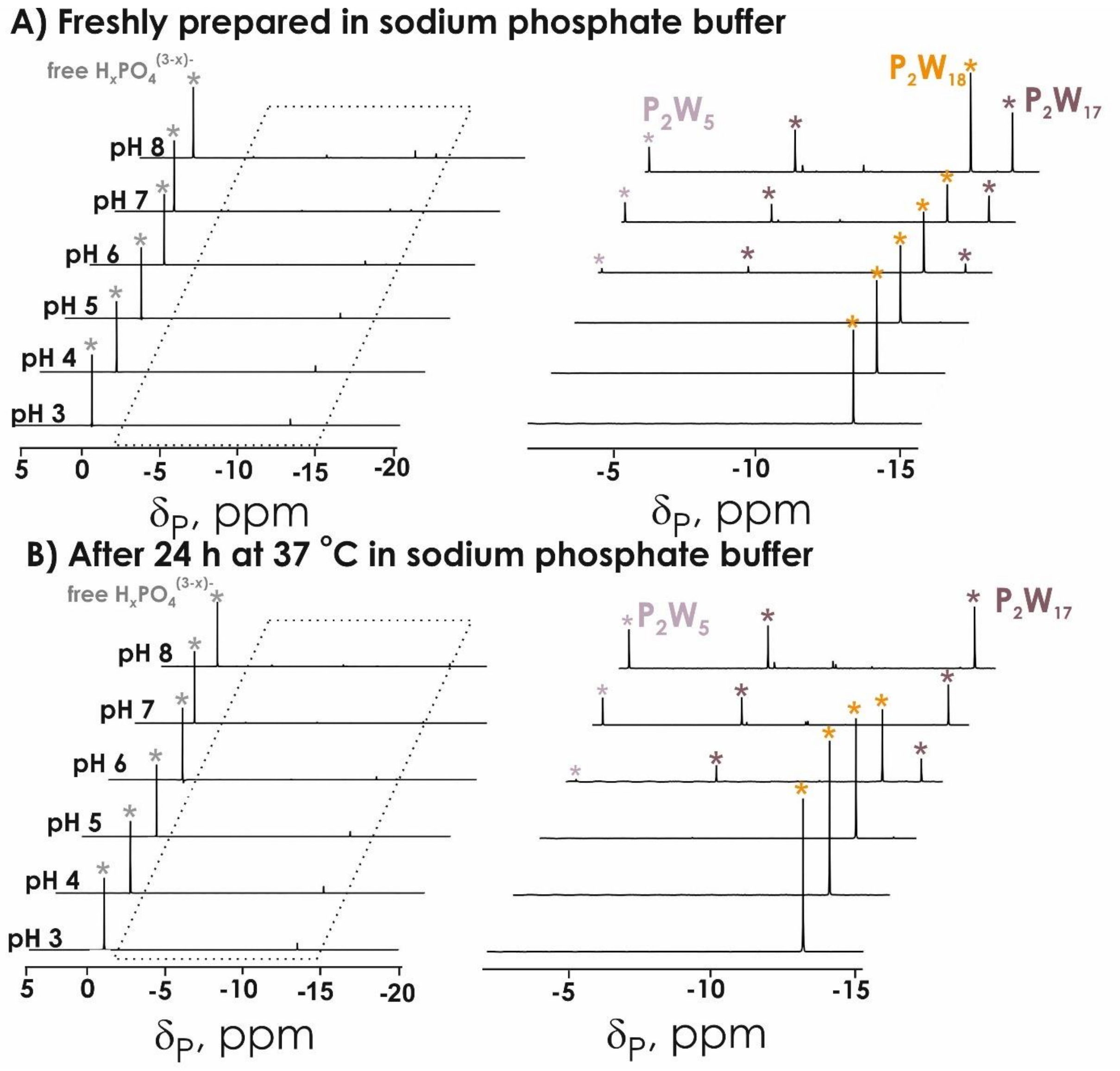

Fig. S36. ^{31}P NMR spectra of α-P_2W_{18} in sodium phosphate buffer.

^{31}P NMR spectra for 10 mM solutions of **$K_6[\alpha\text{-}P^V_2W^{VI}_{18}O_{62}]$** in 0.1 M sodium phosphate buffer (pH 3 – 8) that were recorded approximately one hour after preparation (**A**) and after incubation for 24 h at 37 °C (**B**). The structures of all POMs are shown in **Figure S31**. The chemical shifts and percentages of parent and formed species are given in **Tables S10** and **S11**. To identify the individual anions, they are shown in different colors, with the same color code being selected for a specific anion throughout all figures and tables in the main manuscript and the supporting information.

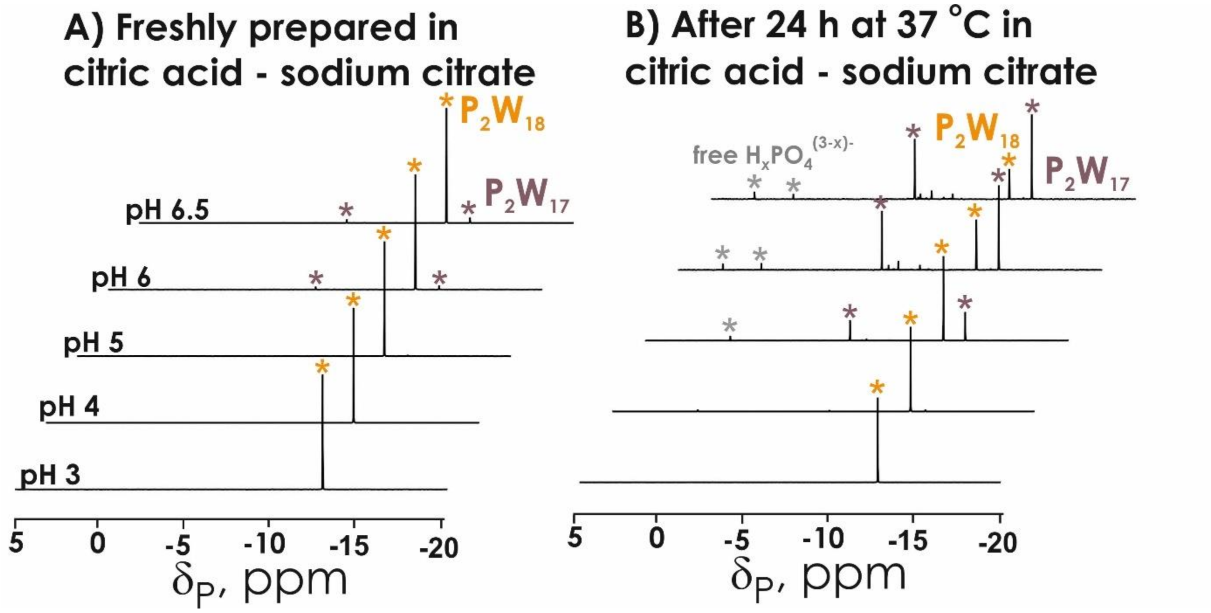

Fig. S37. ^{31}P NMR spectra of α-P_2W_{18} in citric acid – sodium citrate buffer

^{31}P NMR spectra for 10 mM solutions of **$K_6[\alpha\text{-}P^{V}_2W^{VI}_{18}O_{62}]$** in 0.1 M citric acid – sodium citrate buffer (pH 3 – 6.5) that were recorded approximately one hour after preparation (**A**) and after incubation for 24 h at 37 °C (**B**). The structures of all POMs are shown in **Figure S31**. The chemical shifts and percentages of parent and formed species are given in **Tables S10** and **S11**. To identify the individual anions, they are shown in different colors, with the same color code being selected for a specific anion throughout all figures and tables in the main manuscript and the supporting information.

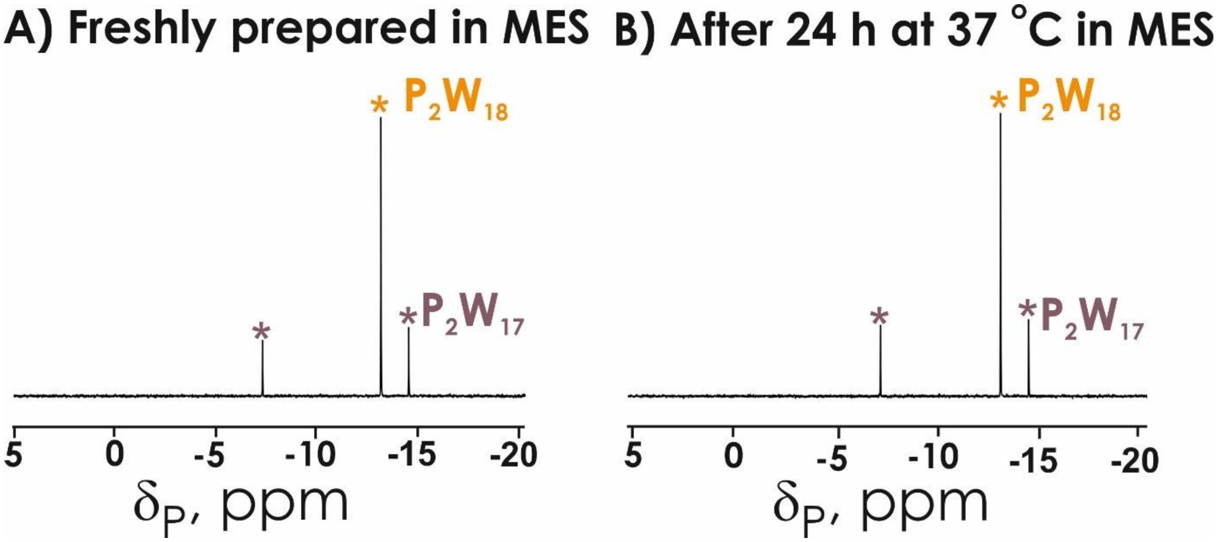

Fig. S38. ^{31}P NMR spectra of α-P_2W_{18} in MES buffer.

^{31}P NMR spectra for 10 mM solutions of $\mathbf{K_6[\alpha\text{-}P^{V}_2W^{VI}_{18}O_{62}]}$ in 0.1 M MES buffer pH 5.5 that were recorded approximately one hour after preparation (**A**) and after incubation for 24 h at 37 °C (**B**). The structures of all POMs are shown in **Figure S31**. The chemical shifts and percentages of parent-formed species are given in **Tables S10** and **S11**. To identify the individual anions, they are shown in different colors, with the same color code being selected for a specific anion throughout all figures and tables in the main manuscript and the supporting information.

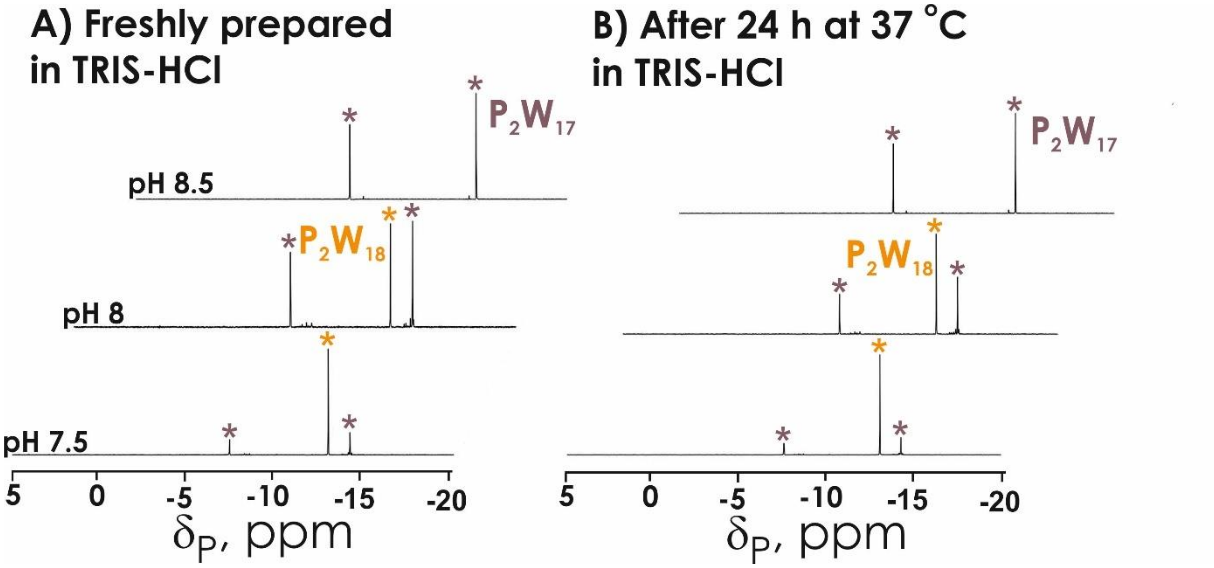

Fig. S39. ^{31}P NMR spectra of α-P_2W_{18} in tris-HCl buffer.

^{31}P NMR spectra for 10 mM solutions of **$K_6[\alpha\text{-}P^{V}_2W^{VI}_{18}O_{62}]$** in 0.1 M tris-HCl buffer (pH 7.5 – 8.5) that were recorded approximately one hour after preparation (**A**) and after incubation for 24 h at 37 °C (**B**). The structures of all POMs are shown in **Figure S31**. The chemical shifts and percentages of parent and formed species are given in **Tables S10** and **S11**. To identify the individual anions, they are shown in different colors, with the same color code being selected for a specific anion throughout all figures and tables in the main manuscript and the supporting information.

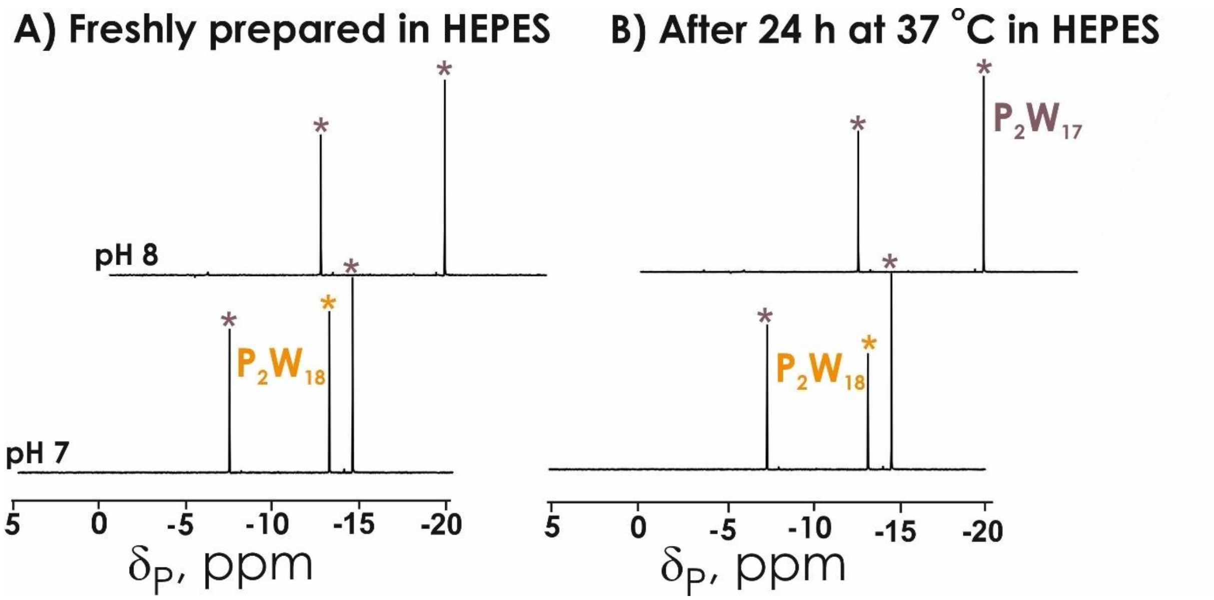

Fig. S40. ^{31}P NMR spectra of α-P_2W_{18} in HEPES buffer

^{31}P NMR spectra for 10 mM solutions of $\mathbf{K_6[\alpha\text{-}P^{V}_2W^{VI}_{18}O_{62}]}$ in HEPES buffer (pH 7 and 8) that were recorded approximately one hour after preparation (**A**) and after incubation for 24 h at 37 °C (**B**). The structures of all POMs are shown in **Figure S31**. The chemical shifts and percentages of parent and formed species are given in **Tables S10** and **S11**. To identify the individual anions, they are shown in different colors, with the same color code being selected for a specific anion throughout all figures and tables in the main manuscript and the supporting information.

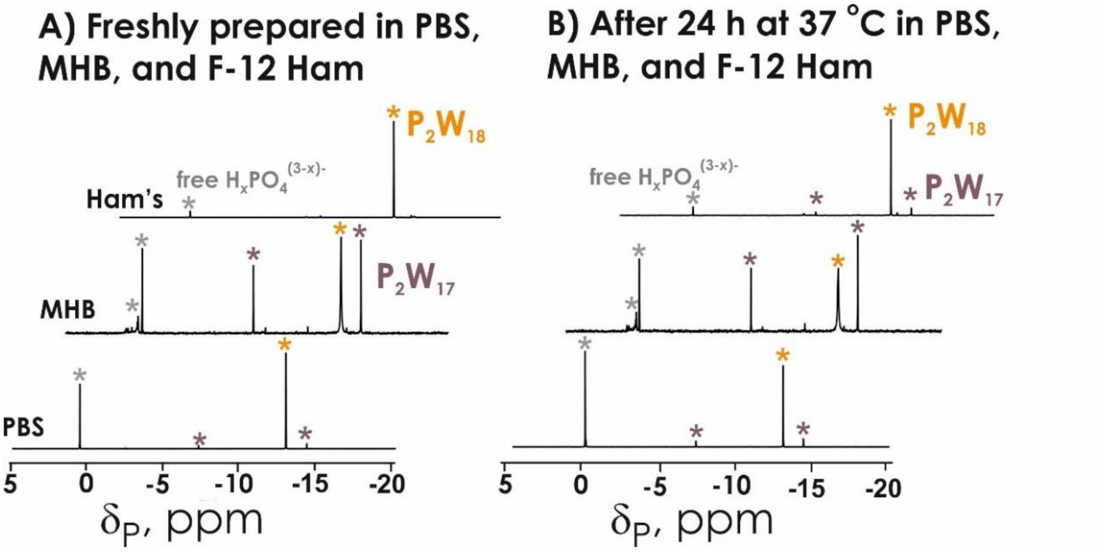

Fig. S41. ³¹P NMR spectra of α-P_2W_{18} in solutions with pH 7.4.

^{31}P NMR spectra for 10 mM solutions of **$K_6[\alpha\text{-}P^{V}_2W^{VI}_{18}O_{62}]$** in PBS, MHB and nutrient mixture F-12 Ham (pH 7.4) that were recorded approximately one hour after preparation (**A**) and after incubation for 24 h at 37 °C (**B**). The structures of all POMs are shown in **Figure S31**. The chemical shifts and percentages of parent and formed species are given in **Tables S10** and **S11**. To identify the individual anions, they are shown in different colors, with the same color code being selected for a specific anion throughout all figures and tables in the main manuscript and the supporting information.

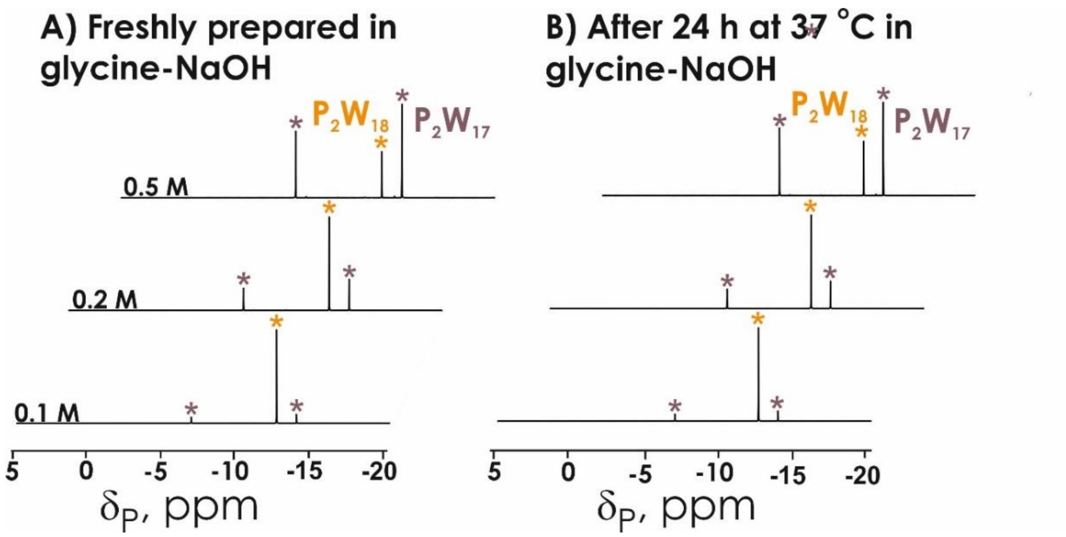

Fig. S42. ^{31}P NMR spectra of *α*-P_2W_{18} in glycine-NaOH buffer.

^{31}P NMR spectra for 10 mM **$K_6[\alpha\text{-}P^{V}_2W^{VI}_{18}O_{62}]$** solutions in glycine-NaOH (pH 8.6) with concentration 0.1. 0.2 and 0.5 M, respectively that were recorded approximately one hour after preparation (**A**) and after incubation for 24 h at 37 °C (**B**). The structures of all POMs are shown in **Figure S31**. The chemical shifts and percentages of parent and formed species are given in **Tables S10** and **S11**. To identify the individual anions, they are shown in different colors, with the same color code being selected for a specific anion throughout all figures and tables in the main manuscript and the supporting information.

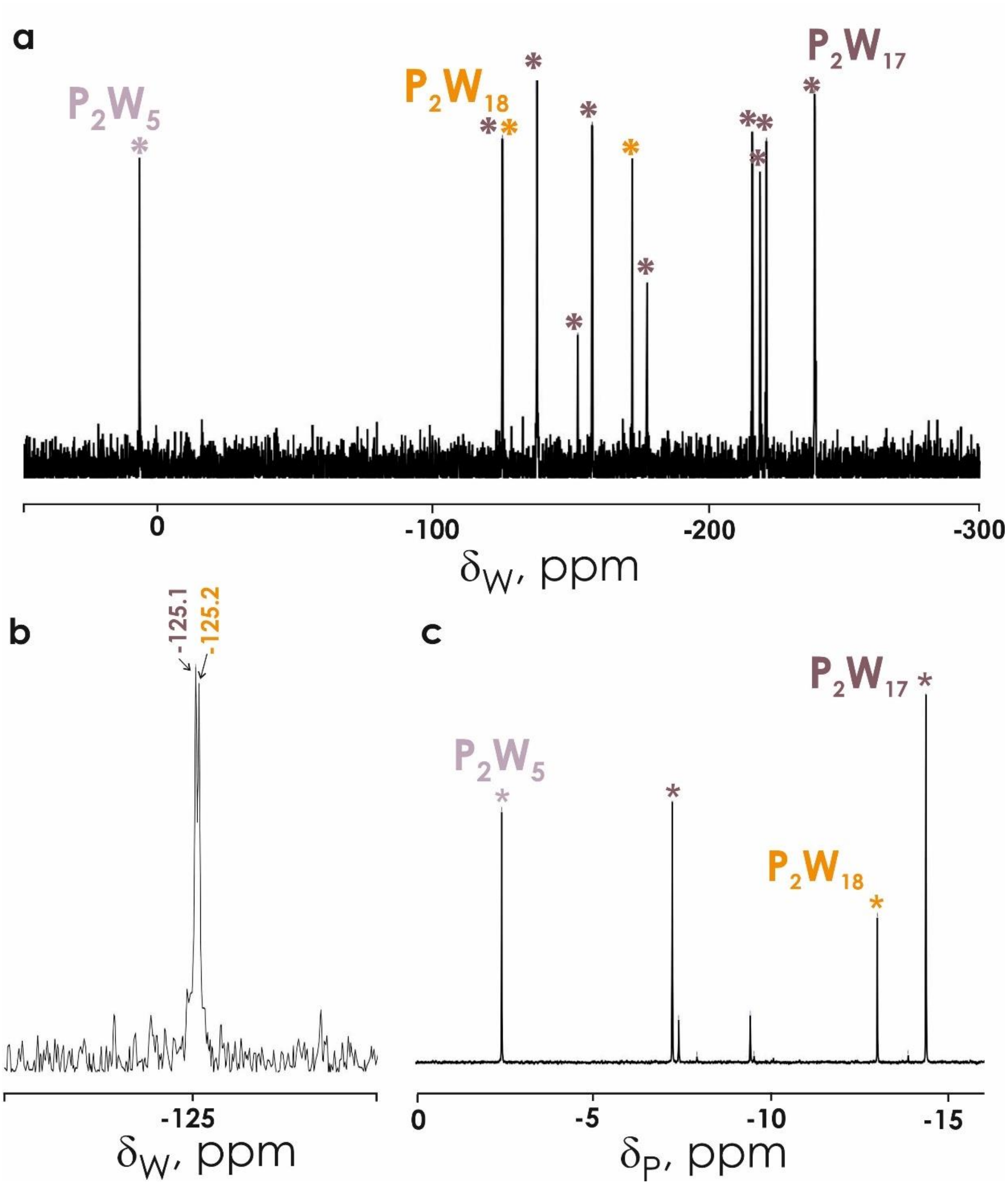

Fig. S43. ^{183}W NMR spectra of α-P_2W_{18}.

^{183}W (**a**, **b**) and ^{31}P (**c**) NMR spectra for 20 mM solutions of **$K_6[\alpha\text{-}P^V{}_2W^{VI}{}_{18}O_{62}]$** in 0.2 M sodium phosphate buffer at pH 8. The ^{183}W NMR spectrum shows 12 signals, two at –125.2 and –172.6 that correspond to 2 types of W ion in $[\alpha\text{-}P_2W_{18}O_{62}]^{6-}$; nine signals at –125.1, –137.8, –152.7, –157.9, –178.1, –216.5, –219.3, –221.7 and –239.3 ppm that correspond to $[\alpha_2\text{-}P^V{}_2W^{VI}{}_{17}O_{61}]^{10-}$ (Table S4); and one signal at +7.5 ppm that presumably arises from $[P^V{}_2W^{VI}{}_5O_{23}]^{6-}$. The structures of all POMs are shown in **Figure S11** and **S31**. To identify the individual anions, they are shown in different colors, with the same color code being selected for a specific anion throughout all figures and tables in the main manuscript and the supporting information.

Table S10. Analysis of NMR spectroscopic data recorded in α-P_2W_{18} solutions at room temperature.

Chemical shifts in ^{31}P NMR spectra measured in triplicate of **$K_6[\alpha\text{-}P^V_2W^{VI}_{18}O_{62}]$** (10 mM) dissolved in D_2O and different buffers (acetic acid – sodium acetate pH 4 – 5.5; sodium phosphate pH 3 – 8 (while phosphate does not buffer at pH range from 3.5 – 5.5, experiments were conducted at this pH to provide comparisons to previously published studies (*29*)); citric acid – sodium citrate pH 3 – 6.5; MES pH 5.5; PBS pH 7.4; tris-HCl pH 7.5 – 8.5; HEPES pH 7 – 8; MHB pH 7.4; Nutrient mixture F-12 Ham pH 7.4 and glycine-NaOH pH 8.6) with concentration 0.1 M. Glycine-NaOH buffer was used in two additional concentrations 0.2 and 0.5 M (**Figures S34** – **S42**). The species content was calculated based on the integration of ^{31}P signals considering only signals associated with POTs. Signals were assigned based on the literature data summarized in **Table S4**.

**pH**	**$K_6[\alpha\text{-}P_2W_{18}O_{62}]$** (10 mM) in Solvent / Buffer / Medium	Chemical shifts δ ^{31}P [ppm]	% of α-P_2W_{18} at RT in **$K_6[\alpha\text{-}P^V_2W^{VI}_{18}O_{62}]$** (10 mM) solution				% of P_2W_{17} at RT in **$K_6[\alpha\text{-}P^V_2W^{VI}_{18}O_{62}]$** (10 mM) solution				% of P_2W_5 at RT in **$K_6[\alpha\text{-}P^V_2W^{VI}_{18}O_{62}]$** (10 mM) solution				Average % of other POTs
			Sample			Mean of 1 to 3 ± SD[a]	Sample			Mean of 1 to 3 ± SD[a]	Sample			Mean of 1 to 3 ± SD[a]	
			#1	#2	#3		#1	#2	#3		#1	#2	#3		
-	D_2O	**–13.0**	100	100	100	100 ± 0	0	0	0	0	0	0	0	0	0
Strongly acidic environment 3 ≤ pH ≤ 4															
3	0.1 M Sodium phosphate ($H_2PO_4^-/H_3PO_4$) pH 3	0; **–13.0**	100	100	100	100 ± 0	0	0	0	0	0	0	0	0	0
	0.1 M Citric acid – sodium citrate (H_3Cit/H_2Cit^-) pH 3	**–13.0**	99	100	100	100 ± 1	0	0	0	0	0	0	0	0	0
4	0.1 M Sodium phosphate ($H_2PO_4^-/H_3PO_4$) pH 4	0; **–13.0**	100	100	100	100 ± 0	0	0	0	0	0	0	0	0	0

	0.1 M Citric acid – sodium citrate (H_2Cit^- /$HCit^{2-}$) pH 4	–11.3; –11.5; **–13.0**	99	100	100	100 ± 1	0	0	0	0	0	0	0	0	0
	0.1 M Acetic acid – sodium acetate (OAc^- /HOAc) pH 4	**–13.0**	100	100	100	100 ± 0	0	0	0	0	0	0	0	0	0
Moderately acidic environment 5 ≤ pH ≤ 6														
5	0.1 M Sodium phosphate ($H_2PO_4^-$/H_3PO_4) pH 5	0; –2.4; **–7.3**; –10.1; –11.5; –12.3; **–13.0**; **–14.4**	99	99	99	99 ± 0	0	0	1	0 ± 1	0	0	0	0	1
5	0.1 M Citric acid – sodium citrate (H_2Cit^- /$HCit^{2-}$) pH 5	0.1; **–7.3**; –10.1; –11.5; –12.3; **–13.0**; **–14.4**	98	96	99	98 ± 2	1	4	1	2 ± 2	0	0	0	0	0
5	0.1 M Acetic acid – sodium acetate (OAc^- /HOAc) pH 5	**–7.3**; –10.1; –11.5; –12.3; **–13.0**; **–14.4**	97	98	98	98 ± 1	2	1	2	2 ± 1	0	0	0	0	0
5.5	0.1 M Acetic acid – sodium acetate (OAc^- /HOAc) pH 5.5	**–7.3**; –10.1; –11.5; –12.3; **–13.0**; **–14.4**	94	94	95	94 ± 1	5	5	5	5 ± 0	0	0	0	0	1
5.5	0.1 M MES[b] pH 5.5	**–7.2**; **–13.0**; **–14.4**	73	70	69	71 ± 2	27	30	31	29 ± 2	0	0	0	0	0
6	0.1 M Sodium phosphate (HPO_4^{2-}/$H_2PO_4^-$) pH 6	0; **–2.4**; **–7.3**; **–13.0**; **–14.4**	81	76	76	78 ± 3	15	19	19	18 ± 2	4	5	5	5 ± 1	0

	0.1 M Citric acid – sodium citrate ($HCit^{2-}$/Cit^{3-}) pH 6	**–7.3**; –8.3; –10.1; –11.5; –12.3; **–13.0**; **–14.4**	92	86	95	91 ± 5	3	13	5	7 ± 5	0	0	0	0	2
Neutral environment 6.5 ≤ pH ≤ 7.5															
6.5	0.1 M Citric acid – sodium citrate ($HCit^{2-}$/Cit^{3-}) pH 6.5	**–7.3**; –8.3; –10.1; –11.5; –12.3; **–13.0**; **–14.4**	88	90	93	90 ± 3	6	9	7	7 ± 2	0	0	0	0	3
7	0.1 M Sodium phosphate (HPO_4^{2-}/$H_2PO_4^{-}$) pH 7	3.9; 3.2; 1.1; **–2.4**; **–7.2**; –7.4; –8.0; –9.5; **–13.0**; –13.9; **–14.4**	16	11	15	14 ± 3	54	57	58	56 ± 2	22	24	18	21 ± 3	0
	0.1 M HEPES[c] pH 7	**–7.3**; –8.0; **–13.0**; –14.0; **–14.4**	39	34	32	35 ± 4	61	66	68	65 ± 4	0	0	0	0	0
7.4	PBS[d] pH 7.4	0.7; **–7.3**; –8.0; –10.1; **–13.0**; –14.0; **–14.4**	90	91	91	91 ± 1	9	9	8	9 ± 1	0	0	0	0	0
	MHB[e] pH 7.4	0.6; –2.4; **–7.2**; –10.1; –12.3; **–13.1**; **–14.4**	72	70	73	72 ± 2	26	29	26	27 ± 2	0	0	0	0	1
	Nutrient mixture F-12 Ham[f]	0.5; **–7.2**; **–8.1**; –8.2; –13.0; **–13.4**; **–14.2**; **–14.4**	91	91	92	91 ± 1	3	3	3	3 ± 0	0	0	0	0	6
7.5	0.1 M tris-HCl[g] pH 7.5	**–7.3**; **–13.0**; **–14.4**	75	70	66	70 ± 5	22	26	30	26 ± 4	0	0	0	0	4
Moderately alkaline environment 8 ≤ pH ≤ 8.6															

8	0.1 M Sodium phosphate ($HPO_4^{2-}/H_2PO_4^-$) pH 8	4.1; 3.5; 1.5; **–2.4**; **–7.3**; –7.4; –9.4; –9.6; **–13.0**; –13.9; **–14.4**	12	6	10	9 ± 3	63	61	60	61 ± 2	25	23	25	24± 1	0
	0.1 M HEPES pH 8	**–7.3**; –8.0; –10.1; **–13.1**; –14.0; **–14.4**	0	0	0	0	99	100	99	99 ±	0	0	0	0	1
	0.1 M tris-HCl pH 8	**–7.4**; **–13.0**; **–14.3**	58	40	48	48 ± 9	38	55	49	47 ± 9	0	0	0	0	5
8.5	0.1 M tris-HCl pH 8.5	**–7.3**; **–13.0**; **–14.3**	9	0	0	3 ± 5	91	100	100	97 ± 5	0	0	0	0	0
8.6	0.1 M glycine-NaOH pH 8.6	**–7.3**; –10.1; –11.5; –12.3; **–13.0**; **–14.4**	89	84	85	86 ± 3	11	16	15	14 ± 3	0	0	0	0	0
	0.2 M glycine-NaOH pH 8.6	**–7.3**; **–13.0**; **–14.4**	61	59	61	60 ± 1	39	41	39	40 ± 4	0	0	0	0	0
	0.5 M glycine-NaOH pH 8.6	**–7.3**; –8.0; –10.1; **–13.0**; –13.9; **–14.4**	25	21	21	22 ± 2	75	79	79	78 ± 2	0	0	0	0	0

[a]SD – standard deviation; [b]MES – 2-(N-morpholino)ethanesulfonic acid, $C_6H_{13}NO_4S$ (Figure S1); [c]HEPES – 4-(2-hydroxyethyl)-1-piperazineethanesulfonic acid, $C_8H_{18}N_2O_4S$ (Figure S1); [d]PBS – phosphate buffer saline; [e]MHB – Mueller-Hinton broth, for more detailed information about composition see https://labmal.com/2019/11/20/mueller-hinton-agar-and-mueller-hinton-broth/; [f]Nutrient mixture F-12 Ham contains sodium pyruvate (0.11 g/L), phenol red, L-glutamine, and does not contain $NaHCO_3$ and HEPES, for more details please see https://www.sigmaaldrich.com/AT/en/technical-documents/technical-article/cell-culture-and-cell-culture-analysis/mammalian-cell-culture/f-12-ham; [g]tris – tris(hydroxymethyl)aminomethane, $C_4H_{11}NO_3$ (Figure S1).

Table S11. Analysis of NMR spectroscopic data recorded in *α*-P_2W_{18} solutions investigated after 24 h incubation at 37 °C. Chemical shifts in ^{31}P NMR spectra measured in triplicate of **K_6[*α*-$P^V{}_2W^{VI}{}_{18}O_{62}$]** (10 mM) dissolved in D_2O and different buffers (acetic acid – sodium acetate pH 4 – 5.5; sodium phosphate pH 3 – 8 (while phosphate does not buffer at pH range from 3.5 – 5.5, experiments were conducted at this pH to provide comparisons to previously published studies (*29*)); citric acid – sodium citrate pH 3 – 6.5; MES pH 5.5; PBS pH 7.4; tris-HCl pH 7.5 – 8.5; HEPES pH 7 – 8; MHB pH 7.4; Nutrient mixture F-12 Ham pH 7.4 and glycine-NaOH pH 8.6) with concentration 0.1 M and investigated after 24 h incubation at 37 °C. The glycine-NaOH buffer was used in two additional concentrations 0.2 and 0.5 M (**Figures S34** – **S42**). The species content was calculated based on the integration of ^{31}P signals considering only signals associated with POTs. Signals were assigned based on the literature data summarized in **Table S4**.

**pH**	**K_6[*α*-$P_2W_{18}O_{62}$]** (10 mM) in Solvent / Buffer / Medium	Chemical shifts δ ^{31}P [ppm]	% of *α*-P_2W_{18} in **K_6[*α*-$P^V{}_2W^{VI}{}_{18}O_{62}$]** (10 mM) solution after 24 h incubation at 37 °C				% of P_2W_{17} in **K_6[*α*-$P^V{}_2W^{VI}{}_{18}O_{62}$]** (10 mM) solution after 24 h incubation at 37 °C				% of P_2W_5 in **K_6[*α*-$P^V{}_2W^{VI}{}_{18}O_{62}$]** (10 mM) solution after 24 h incubation at 37 °C				Average % of other POT
			Sample #1	Sample #2	Sample #3	Mean of 1 to 3 ± SD[a]	Sample #1	Sample #2	Sample #3	Mean of 1 to 3 ± SD[a]	Sample #1	Sample #2	Sample #3	Mean of 1 to 3 ± SD[a]	
-	D_2O	–10.1; –11.5; –12.3; **–13.0**	100	100	100	100 ± 0	0	0	0	0	0	0	0	0	0
Strongly acidic environment 3 ≤ pH ≤ 4															
3	0.1 M Sodium phosphate ($H_2PO_4^-$ /H_3PO_4) pH 3	0; **–13.0**	100	100	100	100 ± 0	0	0	0	0	0	0	0	0	0
	0.1 M Citric acid – sodium citrate (H_3Cit/H_2Cit^-) pH 3	–10.1; –12.6; –12.3; **–13.0**	100	100	100	100 ± 0	0	0	0	0	0	0	0	0	0
4	0.1 M Sodium phosphate	0; **–13.0**	100	100	100	100 ± 0	0	0	0	0	0	0	0	0	0

	($H_2PO_4^-$ /H_3PO_4) pH 4														
	0.1 M Citric acid – sodium citrate (H_2Cit^- /$HCit^{2-}$) pH 4	0; –8.0; –10.1; –12.3; –13.0; –13.9	87	97	96	93 ± 6	2	3	3	3 ± 1	0	0	0	0	4
	0.1 M Acetic acid – sodium acetate (OAc^- /HOAc) pH 4	–10.1; –11.5; –12.3; –13.0	100	100	100	100 ± 0	0	0	0	0	0	0	0	0	0
Moderately acidic environment 5 ≤ pH ≤ 6															
5	0.1 M Sodium phosphate ($H_2PO_4^-$ /H_3PO_4) pH 5	0; –7.4; –10.1; –11.5; –12.3; –13.0; –14.3	99	99	99	99 ± 0	1	1	1	1 ± 0	0	0	0	0	0
	0.1 M Citric acid – sodium citrate (H_2Cit^- /$HCit^{2-}$) pH 5	2.2; 0; –7.3; –8.0; –8.1; –9.7; –13.0; –14.4	66	59	61	62 ± 4	30	39	38	36 ± 5	0	0	0	0	2
	0.1 M Acetic acid – sodium acetate (OAc^- /HOAc) pH 5	–7.3; –10.1; –13.0; –14.4	94	94	97	95 ± 2	3	3	3	3 ± 0	0	0	0	0	2
5.5	0.1 M Acetic acid – sodium acetate (OAc^- /HOAc) pH 5.5	–7.3; –13.0; –14.4	82	77	77	78 ± 3	18	23	23	21 ± 3	0	0	0	0	1
	0.1 M MES[b] pH 5.5	–7.3; –13.0; –14.4	66	68	66	67 ± 1	34	32	34	33 ± 1	0	0	0	0	0
6	0.1 M Sodium phosphate	–2.4; –7.3; –10.9; –13.0; –14.4	68	65	62	65 ± 3	31	33	36	33 ± 3	1	2	2	2 ± 1	2

	(HPO_4^{2-}/$H_2PO_4^-$) pH 6													
	0.1 M Citric acid – sodium citrate ($HCit^{2-}$/Cit^{3-}) pH 6	2.5; 0; **–7.3**; –7.6; –8.2; –9.6; **–13.0**; –13.9; **–14.4**	31	19	22	24 ± 6	57	72	70	66 ± 8	0	0	0	0	10
Neutral environment 6.5 ≤ pH ≤ 7.5														
6.5	0.1 M Citric acid – sodium citrate ($HCit^{2-}$/Cit^{3-}) pH 6.5	2.6; 0.2; –7.2; **–7.4**; –7.6; –7.9; –8.2; –9.0; –9.5; **–13.0**; –13.9; **–14.4**	20	11	14	15 ± 5	59	76	75	70 ± 9	0	0	0	0	15
7	0.1 M Sodium phosphate (HPO_4^{2-}/$H_2PO_4^-$) pH 7	3.8; 3.4; ;1.0; **–2.4**; **–7.2**; –7.4; –7.9; –9.5; –9.6; –10.9; –13.9; **–14.4**	0	0	0	0	71	70	66	69 ± 3	22	22	24	23 ± 1	8
	0.1 M HEPES[c] pH 7	**–7.3**; –8.0; –10.1; **–13.1**; –14.0; **–14.4**	30	29	28	29 ± 1	69	71	72	71 ± 1	0	0	0	0	0
7.4	PBS[d] pH 7.4	0; **–7.3**; –10.1; –10.9; –11.5; –12.3; **–13.0**; **–14.4**	83	74	84	80 ± 6	17	26	16	20 ± 6	0	0	0	0	0

	MHB[e] pH 7.4	0.9; 0.3; 0; **–7.3**; –8.1; –10.1; –10.9; **–13.1**; –13.5; –13.9; **–14.4**	72	67	68	69 ± 3	27	31	31	30 ± 2	0	0	0	0	1
	Nutrient mixture F-12 Ham[f]	0; **–7.3**; –8.1; –10.1; –10.8; –12.3; **–13.0**; –13.4; **–14.4**	81	79	81	80 ± 1	14	16	14	15 ± 1	0	0	0	0	5
7.5	0.1 M tris-HCl[g] pH 7.5	**–7.5**; –8.3; –8.6; –10.1; **–13.0**; –13.8; –14.1; –14.2; –14.3; **–14.4**	62	72	72	69 ± 6	26	26	26	26 ± 0	0	0	0	0	5
Moderately alkaline environment 8 ≤ pH ≤ 8.6														
8	0.1 M Sodium phosphate (HPO_4^{2-}/$H_2PO_4^{-}$) pH 8	4.0; 3.4; 1.3; **–2.4**; **–7.2**; –7.5; –8.0; –9.5; –9.6; –10.1; –10.8; –13.9; **–14.4**	0	0	0	0	60	64	65	63 ± 3	24	23	22	23 ± 1	14
	0.1 M HEPES pH 8	0.8; **–7.3**; –8.0; –10.1; –14.0; **–14.4**	0	0	0	0	99	99	100	99 ± 1	0	0	0	0	1
	0.1 M tris-HCl pH 8	**–7.4**; –8.1; –8.6; –10.1; **–13.0**; –	40	41	44	42 ± 2	48	52	50	50 ± 2	0	0	0	0	8

		13.8; –13.9; –14.1; –14.3; **–14.4**													
8.5	0.1 M tris-HCl pH 8.5	**–7.2**; –8.0; –14.0; **–14.4**	0	0	0	0	100	100	100	100 ± 0	0	0	0	0	0
8.6	0.1 M glycine-NaOH pH 8.6	**–7.3**; **–13.0**; **–14.4**	85	84	83	84 ± 1	15	16	17	16 ± 1	0	0	0	0	0
	0.2 M glycine-NaOH pH 8.6	**–7.3**; –8.0; –10.1; **–13.0**; –13.9; **–14.4**	62	60	64	62 ± 2	38	40	36	38 ± 2	0	0	0	0	0
	0.5 M glycine-NaOH pH 8.6	**–7.3**; –8.0; –10.1; **–13.0**; –13.9; **–14.4**	26	31	23	27 ± 4	73	69	77	73 ± 4	0	0	0	0	0

[a]SD – standard deviation; [b]MES – 2-(N-morpholino)ethanesulfonic acid, $C_6H_{13}NO_4S$ (Figure S1); [c]HEPES – 4-(2-hydroxyethyl)-1-piperazineethanesulfonic acid, $C_8H_{18}N_2O_4S$ (Figure S1); [d]PBS – phosphate buffer saline; [e]MHB – Mueller-Hinton broth, for more detailed information about composition see https://labmal.com/2019/11/20/mueller-hinton-agar-and-mueller-hinton-broth/; [f]Nutrient mixture F-12 Ham contains sodium pyruvate (0.11 g/L), phenol red, L-glutamine, and does not contain $NaHCO_3$ and HEPES, for more details please see https://www.sigmaaldrich.com/AT/en/technical-documents/technical-article/cell-culture-and-cell-culture-analysis/mammalian-cell-culture/f-12-ham; [g]tris – tris(hydroxymethyl)aminomethane, $C_4H_{11}NO_3$ (Figure S1).

7.3. ^{31}P NMR spectroscopic studies of Wells-Dawson POT $(NH_4)_6[\alpha/\beta\text{-}P^{V}_2W^{VI}_{18}O_{62}]$ solutions

All ^{31}P and ^{183}W peaks with the highest intensity were unambiguously assigned based on the literature data from **Table S4**. In some ^{31}P spectra, chemical shifts for peaks of lower intensity have not yet been described in the literature and therefore can not be assigned in this work.

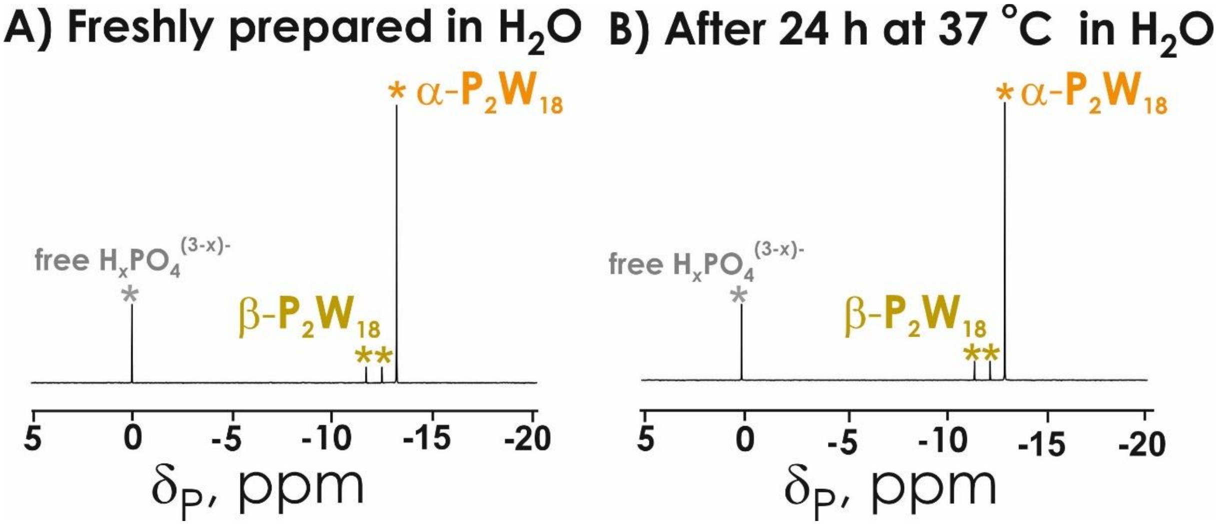

Fig. S44. ^{31}P NMR spectra of *α/β* -P_2W_{18} in H_2O.

^{31}P NMR spectra for 10 mM solutions of **$(NH_4)_6[\alpha/\beta\text{-}P^{V}_2W^{VI}_{18}O_{62}]$** in H_2O that were recorded approximately one hour after preparation (**A**) and after incubation for 24 h at 37 °C (**B**). The structures of all POMs are shown in **Figure S31**. The chemical shifts and percentages of formed species are given in **Tables S12** and **S13**. To identify the individual anions, they are shown in different colors, with the same color code being selected for a specific anion throughout all figures and tables in the main manuscript and the supporting information.

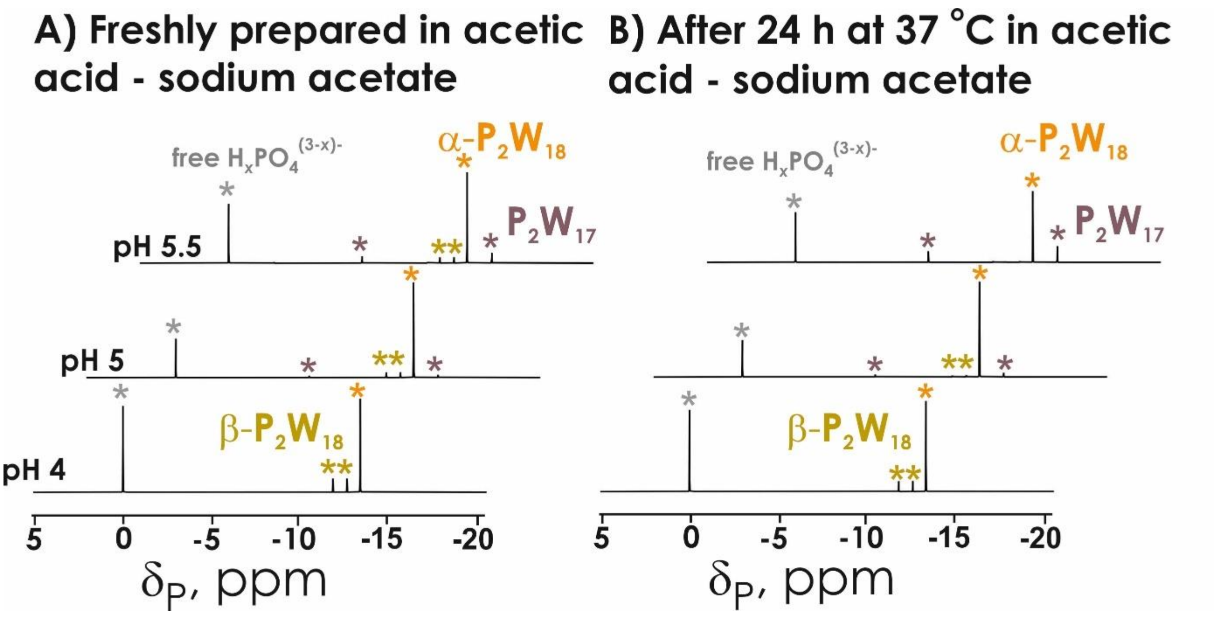

Fig. S45. ^{31}P NMR spectra of *α/β* -P_2W_{18} in NaOAc/HOAc buffer.

^{31}P NMR spectra for 10 mM solutions of **$(NH_4)_6$[*α/β*-$P^V{}_2W^{VI}{}_{18}O_{62}$]** in 0.1 M NaOAc/HOAc buffer (pH 4 – 5.5) that were recorded approximately one hour after preparation (**A**) and after incubation for 24 h at 37 °C (**B**). The structures of all POMs are shown in **Figure S31**. The chemical shifts and percentages of parent and formed species are given in **Tables S12** and **S13**. To identify the individual anions, they are shown in different colors, with the same color code being selected for a specific anion throughout all figures and tables in the main manuscript and the supporting information.

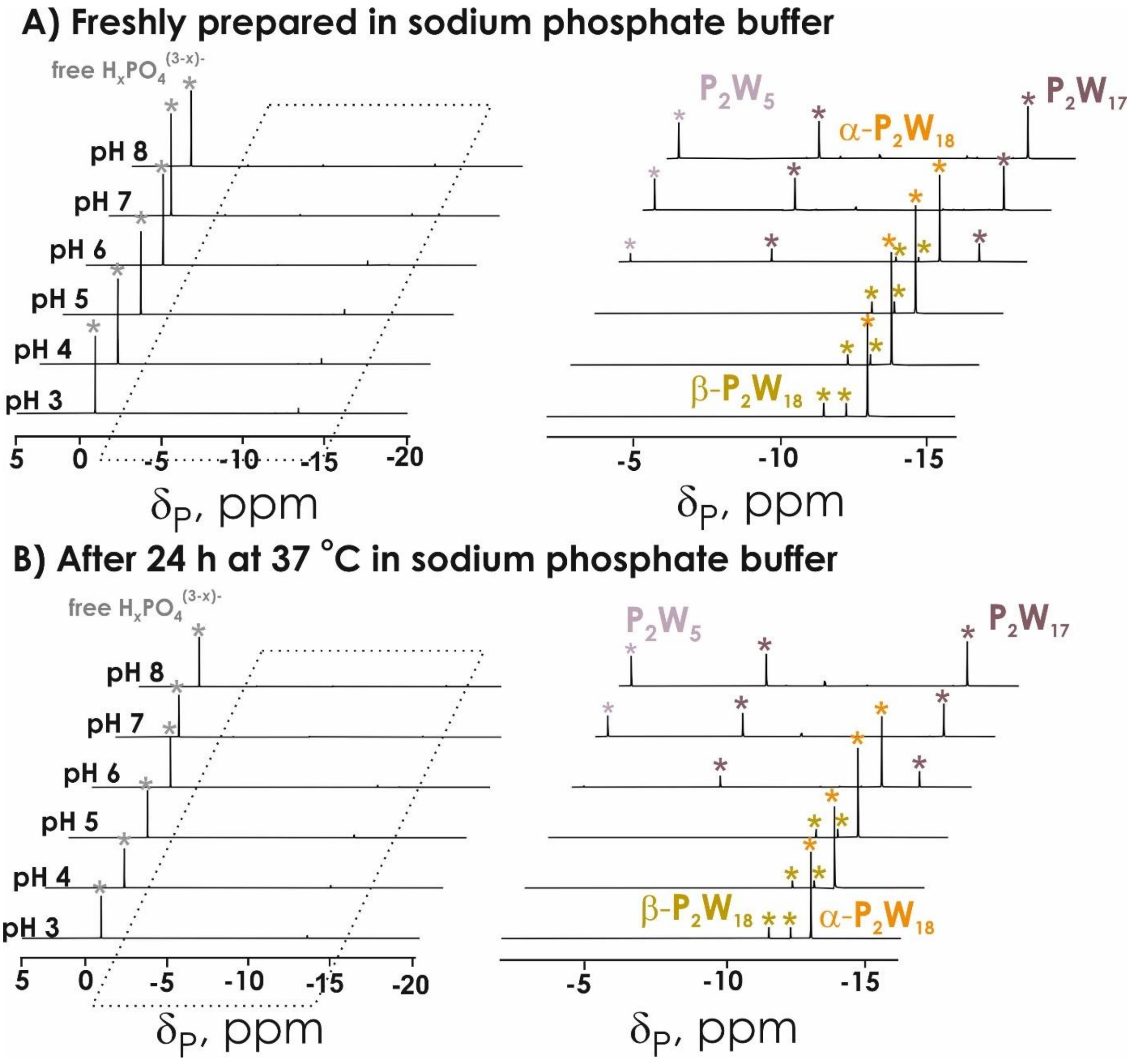

Fig. S46. ^{31}P NMR spectra of *α/β* -P_2W_{18} in sodium phosphate buffer.

^{31}P NMR spectra for 10 mM solutions of **$(NH_4)_6$[*α/β*-$P^V_2W^{VI}_{18}O_{62}$]** in 0.1 M sodium phosphate buffer (pH 3 – 8) that were recorded approximately one hour after preparation (**A**) and after incubation for 24 h at 37 °C (**B**). The structures of all POMs are shown in **Figure S31**. The chemical shifts and percentages of parent and formed species are given in **Tables S12** and **S13**. To identify the individual anions, they are shown in different colors, with the same color code being selected for a specific anion throughout all figures and tables in the main manuscript and the supporting information.

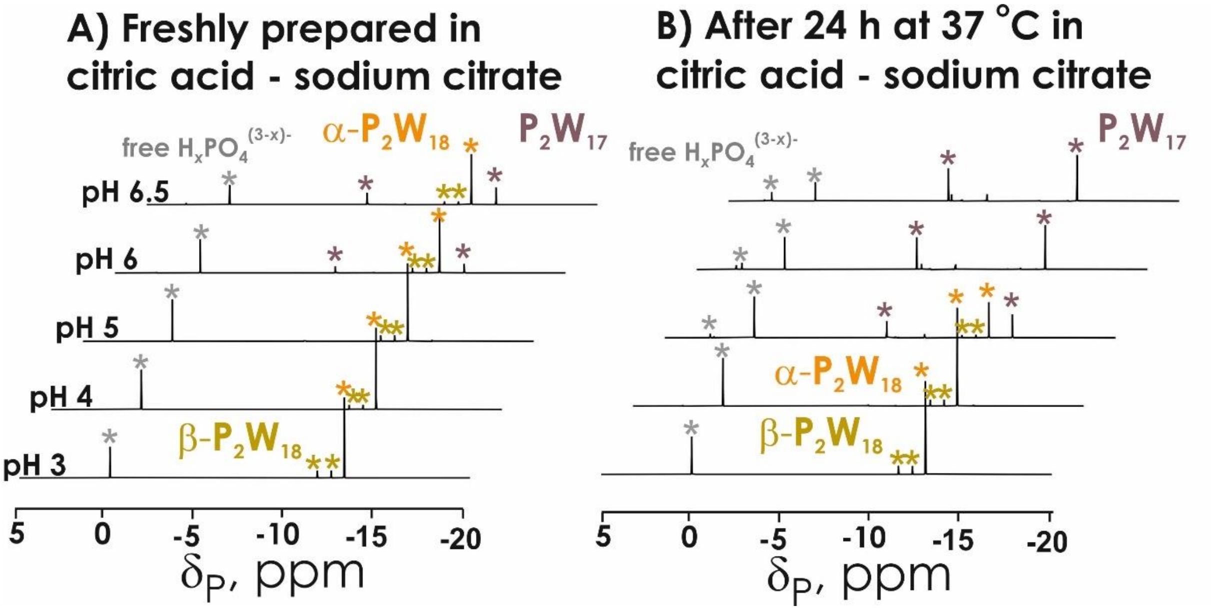

Fig. S47. ^{31}P NMR spectra of *α/β* -P_2W_{18} in citric acid – sodium citrate buffer.

^{31}P NMR spectra for 10 mM solutions of **$(NH_4)_6$[*α/β*-$P^V{}_2W^{VI}{}_{18}O_{62}$]** in 0.1 M citric acid – sodium citrate buffer (pH 3 – 6.5) that were recorded approximately one hour after preparation (**A**) and after incubation for 24 h at 37 °C (**B**). The structures of all POMs are shown in **Figure S31**. The chemical shifts and percentages of parent and formed species are given in **Tables S12** and **S13**. To identify the individual anions, they are shown in different colors, with the same color code being selected for a specific anion throughout all figures and tables in the main manuscript and the supporting information.

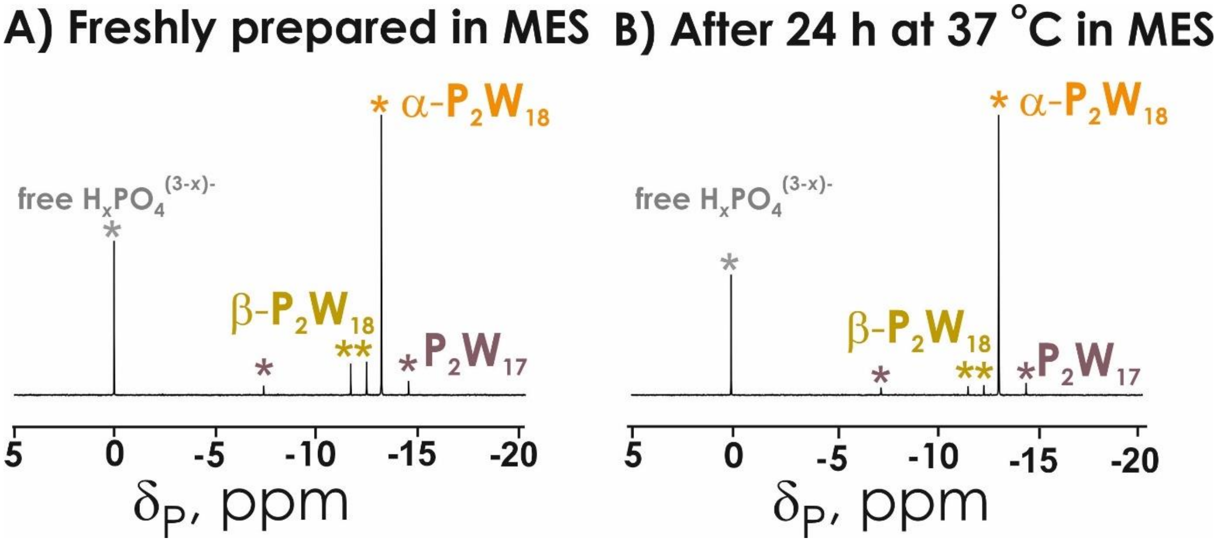

Fig. S48. ^{31}P NMR spectra of *α/β* -P_2W_{18} in MES buffer.

^{31}P NMR spectra for 10 mM solutions of **$(NH_4)_6[\alpha/\beta\text{-}P^V{}_2W^{VI}{}_{18}O_{62}]$** in 0.1 M MES buffer pH 5.5 that were recorded approximately one hour after preparation (**A**) and after incubation for 24 h at 37 °C (**B**). The structures of all POMs are shown in **Figure S31**. The chemical shifts and percentages of parent and formed species are given in **Tables S12** and **S13**. To identify the individual anions, they are shown in different colors, with the same color code being selected for a specific anion throughout all figures and tables in the main manuscript and the supporting information.

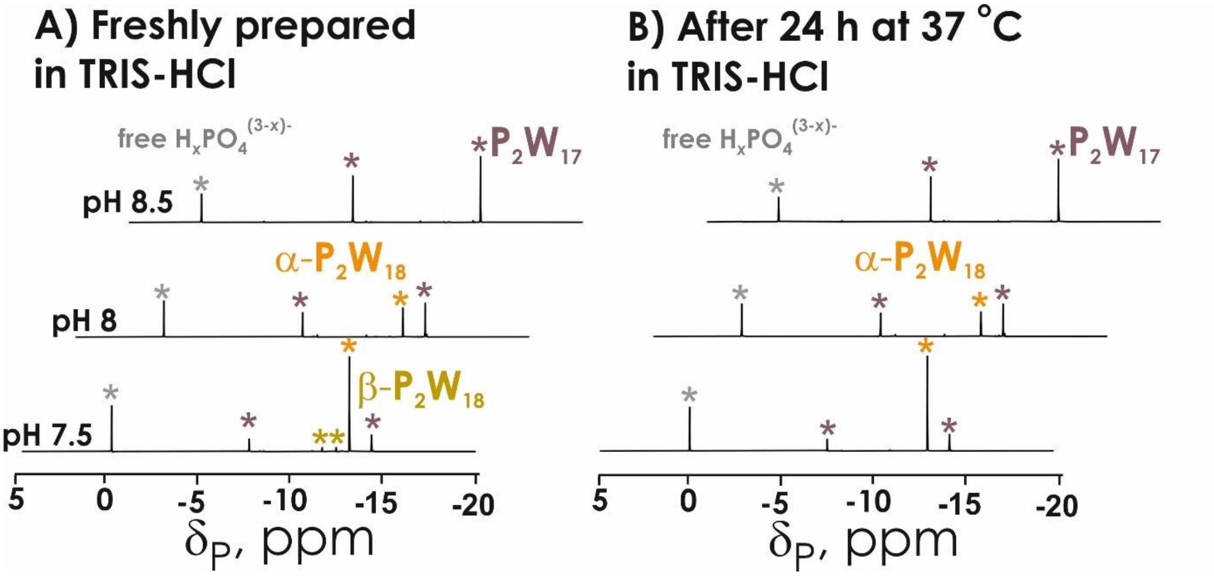

Fig. S49. ^{31}P NMR spectra of *α/β* -P_2W_{18} in tris-HCl buffer.

^{31}P NMR spectra for 10 mM solutions of **$(NH_4)_6[\alpha/\beta\text{-}P^{V}_2W^{VI}_{18}O_{62}]$** in 0.1 M tris-HCl buffer (pH 7.5 – 8.5) that were recorded approximately one hour after preparation (**A**) and after incubation for 24 h at 37 °C (**B**). The structures of all POMs are shown in **Figure S31**. The chemical shifts and percentages of parent and formed species are given in **Tables S12** and **S13**. To identify the individual anions, they are shown in different colors, with the same color code being selected for a specific anion throughout all figures and tables in the main manuscript and the supporting information.

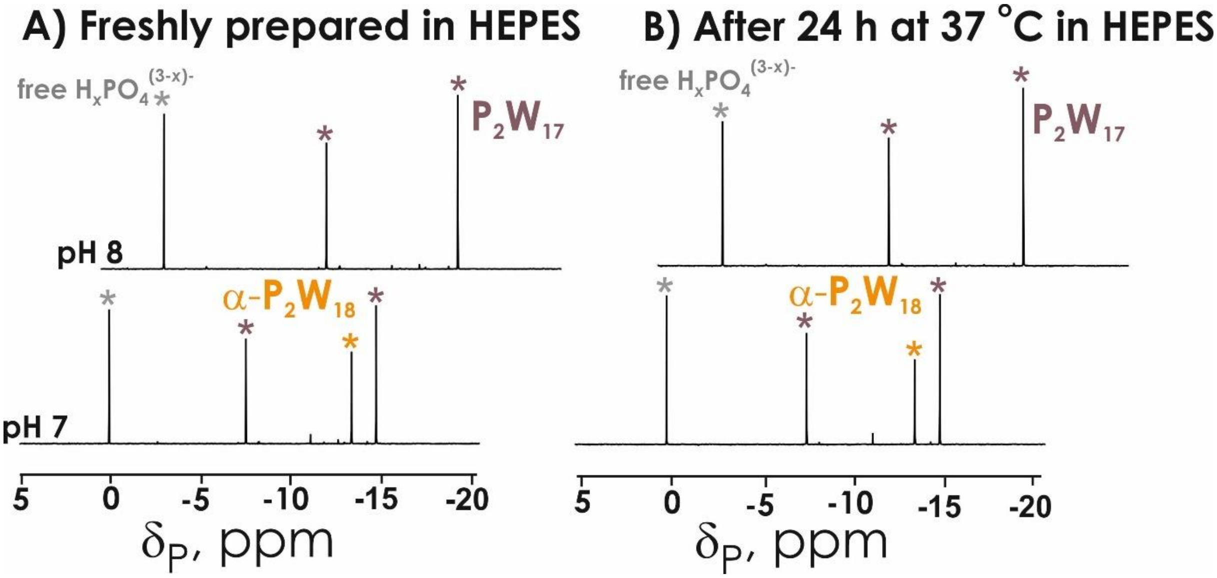

Fig. S50. ^{31}P NMR spectra of *α/β* -P_2W_{18} in HEPS buffer.

^{31}P NMR spectra for 10 mM solutions of **$(NH_4)_6$[*α/β*-$P^V{}_2W^{VI}{}_{18}O_{62}$]** in 0.1 M HEPES buffer (pH 7 and 8) that were recorded approximately one hour after preparation (**A**) and after incubation for 24 h at 37 °C (**B**). The structures of all POMs are shown in **Figure S31**. The chemical shifts and percentages of parent and formed species are given in **Tables S12** and **S13**. To identify the individual anions, they are shown in different colors, with the same color code being selected for a specific anion throughout all figures and tables in the main manuscript and the supporting information.

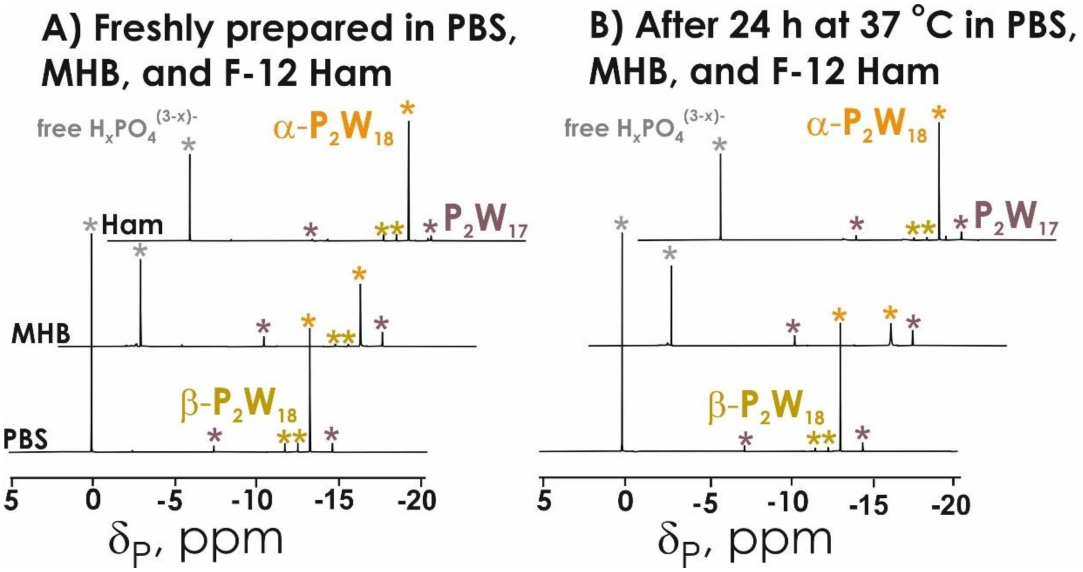

Fig. S51. ^{31}P NMR spectra of *α/β* -P_2W_{18} solutions with pH 7.4.

^{31}P NMR spectra for 10 mM solutions of **$(NH_4)_6$[*α/β*-$P^V{}_2W^{VI}{}_{18}O_{62}$]** in PBS, MHB, and nutrient mixture F-12 Ham (pH 7.4) that were recorded approximately one hour after preparation (**A**) and after incubation for 24 h at 37 °C (**B**). The structures of all POMs are shown in **Figure S31**. The chemical shifts and percentages of parent and formed species are given in **Tables S12** and **S13**. To identify the individual anions, they are shown in different colors, with the same color code being selected for a specific anion throughout all figures and tables in the main manuscript and the supporting information.

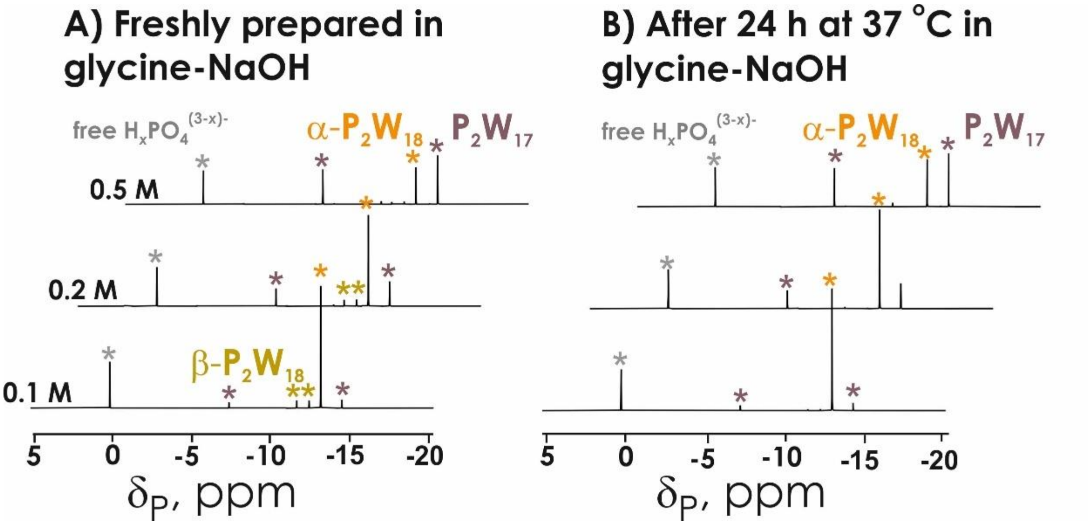

Fig. S52. ^{31}P NMR spectra of *α/β* -P_2W_{18} in glycine-NaOH buffer.

^{31}P NMR spectra for 10 mM solutions of **$(NH_4)_6$[*α/β*-$P^V{}_2W^{VI}{}_{18}O_{62}$]** in glycine-NaOH (pH 8.6) with concentration 0.1. 0.2 and 0.5 M. that were recorded approximately one hour after preparation (**A**) and after incubation for 24 h at 37 °C (**B**). The structures of all POMs are shown in **Figure S31**. The chemical shifts and percentages of parent and formed species are given in **Tables S12** and **S13**. To identify the individual anions, they are shown in different colors, with the same color code being selected for a specific anion throughout all figures and tables in the main manuscript and the supporting information.

Table S12. Analysis of NMR spectroscopic data recorded in *α/β* -P_2W_{18} solutions at room temperature.

Chemical shifts in ^{31}P NMR spectra measured in triplicate of **$(NH_4)_6$[*α/β*-$P^{V}_2W^{VI}_{18}O_{62}$]** (10 mM) dissolved in D_2O and different buffers (acetic acid – sodium acetate pH 4 – 5.5; sodium phosphate pH 3 – 8 (while phosphate does not buffer at pH range from 3.5 – 5.5, experiments were conducted at this pH to provide comparisons to previously published studies (*29*)); citric acid – sodium citrate pH 3 – 6.5; MES pH 5.5; PBS pH 7.4; tris-HCl pH 7.5 – 8.5; HEPES pH 7 – 8; MHB pH 7.4; Nutrient mixture F-12 Ham pH 7.4 and glycine-NaOH pH 8.6) with concentration 0.1 M (**Figures S44 – S52**). The glycine-NaOH buffer was used in two additional concentrations 0.2 and 0.5 M. The species content was calculated based on the integration of 31P signals considering only signals associated with POTs. Signals were assigned based on the literature data summarized in **Table S4**.

pH	**$(NH_4)_6$[*α/β*-$P_2W_{18}O_{62}$]** (10 mM) in Solvent / Buffer / Medium	Chemical shifts δ ^{31}P [ppm]	% of *α*-P_2W_{18} in **$(NH_4)_6$[*α/β*-$P^{V}_2W^{VI}_{18}O_{62}$]** (10 mM) solution at RT				% of *β*-P_2W_{18} in **$(NH_4)_6$[*α/β*-$P^{V}_2W^{VI}_{18}O_{62}$]** (10 mM) solution at RT				% of **sum** of two **P_2W_{18}** isomers in **$(NH_4)_6$[*α/β*-$P^{V}_2W^{VI}_{18}O_{62}$]** (10 mM) solution at RT				% of P_2W_{17} in **$(NH_4)_6$[*α/β*-$P^{V}_2W^{VI}_{18}O_{62}$]** (10 mM) solution at RT				% of P_2W_5 in **$(NH_4)_6$[*α/β*-$P^{V}_2W^{VI}_{18}O_{62}$]** (10 mM) solution after 24 h incubation at 37 °C				Average % of other POTs
			Sample #1	Sample #2	Sample #3	Mean of 1 to 3 ± SD[a]	Sample #1	Sample #2	Sample #3	Mean of 1 to 3 ± SD[a]	Sample #1	Sample #2	Sample #3	Mean of 1 to 3 ± SD[a]	Sample #1	Sample #2	Sample #3	Mean of 1 to 3 ± SD[a]	Sample #1	Sample #2	Sample #3	Mean of 1 to 3 ± SD[a]	
-	D_2O	0; –11.5; –12.3; –13.0	90	88	84	87 ± 3	10	12	16	13 ± 3	100	100	100	100 ± 0	0	0	0	0	0	0	0	0	0
Strongly acidic environment 3 ≤ pH ≤ 4																							
3	0.1 M Sodium phosphate ($H_2PO_4^-$ /H_3PO_4) pH 3	0; –11.5; –12.3; –13.0	80	82	88	83 ± 4	20	18	12	16 ± 4	100	100	100	100 ± 0	0	0	0	0	0	0	0	0	0
3	0.1 M Citric acid – sodium citrate (H_3Cit/H_2Cit^-) pH 3	0; –11.5; –12.3; –13.0	85	91	90	88 ± 3	15	8	10	11 ± 4	100	99	100	100 ± 1	0	0	0	0	0	0	0	0	0
4	0.1 M Sodium phosphate ($H_2PO_4^-$ /H_3PO_4) pH 4	0; –11.5; –12.3; –13.0	83	90	85	86 ± 4	17	10	15	14 ± 4	100	100	100	100 ± 0	0	0	0	0	0	0	0	0	0
4	0.1 M Citric acid – sodium citrate (H_2Cit^- /$HCit^{2-}$) pH 4	0; –11.5; –12.3; –13.1	90	86	87	88 ± 2	10	14	13	12 ± 2	100	100	100	100 ± 0	0	0	0	0	0	0	0	0	0
4	0.1 M Acetic acid – sodium	0; –11.5; –12.3; –13.0	88	92	85	88 ± 4	12	8	15	12 ± 4	100	100	100	100 ± 0	0	0	0	0	0	0	0	0	0

	acetate (OAc^-/HOAc) pH 4																						
Moderately acidic environment 5 ≤ pH ≤ 6																							
5	0.1 M Sodium phosphate ($H_2PO_4^-$/H_3PO_4) pH 5	0; –11.5; –12.3; –13.0	83	86	83	84 ± 2	17	14	17	16 ± 2	100	100	100	100 ± 0	0	0	0	0	0	0	0	0	0
	0.1 M Citric acid – sodium citrate (H_2Cit^-/$HCit^{2-}$) pH 5	0; –7.3; –11.5; –12.3; –13.0; –14.4	86	88	90	88 ± 2	12	10	8	10 ± 2	98	98	98	98 ± 0	2	2	2	2 ± 0	0	0	0	0	0
	0.1 M Acetic acid – sodium acetate (OAc^-/HOAc) pH 5	0; –7.3; –11.5; –12.3; –13.0; –14.4	87	85	80	84 ± 4	10	18	17	15 ± 4	97	98	97	97 ± 1	4	2	3	3 ± 1	0	0	0	0	0
5.5	0.1 M Acetic acid – sodium acetate (OAc^-/HOAc) pH 5.5	0; –2.4; –7.3; –10.9; –11.5; –12.3; –13.0; –14.4	76	73	78	76 ± 3	10	13	12	12 ± 2	86	86	90	87 ± 2	14	14	10	12 ± 2	0	0	0	0	0
	0.1 M MES[b] pH 5.5	0; –7.3; –11.5; –12.3; –13.0; –14.3	78	80	73	77 ± 4	16	10	15	14 ± 3	94	90	88	91 ± 3	6	10	12	9 ±3	0	0	0	0	0
6	0.1 M Sodium phosphate (HPO_4^{2-}/$H_2PO_4^-$) pH 6	0; –2.4; –7.3; –11.5; –12.3; –13.0; –14.4	64	73	67	68 ± 5	9	9	15	11 ± 3	73	82	82	79 ± 5	23	14	15	17 ± 5	3	4	5	4 ± 1	0
	0.1 M Citric acid – sodium citrate ($HCit^{2-}$/Cit^{3-}) pH 6	2.7; 0.3; –7.2; –7.4; –9.4; –11.5; –12.3; –12.7; –13.0; –14.4	71	73	61	68 ± 6	10	9	14	11 ± 3	81	82	75	79 ± 4	18	18	23	20 ± 3	0	0	0	0	1
Neutral environment 6.5 ≤ pH ≤ 7.5																							
6.5	0.1 M Citric acid – sodium citrate ($HCit^{2-}$/Cit^{3-}) pH 6.5	2.8; –0.4; –7.2; –8.0; –9.4; –11.5; –12.3; –12.7; –13.0; –13.9; –14.4	56	55	53	55 ± 2	6	9	9	8 ± 2	62	64	62	63 ± 1	36	34	36	35 ± 1	0	0	0	0	2

4	0.1 M Sodium phosphate (HPO_4^{2-} /$H_2PO_4^-$) pH 7	3.9; 3.2; 1.0; –2.4; –7.3; –8.0; –9.4; –11.5; –12.3; –12.7; –13.0; –13.9; –14.4	12	15	9	12 ± 3	0	3	2	2 ± 2	12	18	11	13 ± 3	61	58	63	61 ± 3	22	20	21	21 ± 1	5
	0.1 M HEPES[c] pH 7	0.1; –7.3; –10.9; –12.4; –13.1; –14.4	28	35	29	30 ± 4	4	5	8	6 ± 2	32	39	36	37 ± 4	64	59	60	61 ± 3	0	0	0	0	4
7.4	PBS[d] pH 7.4	0; –2.4; –7.3; –11.5; –12.3; –13.0; –14.4	78	82	70	76 ± 6	10	8	18	12 ± 5	88	90	88	89 ± 1	10	9	9	9 ± 1	0	0	0	0	2
	MHB[e] pH 7.4	0; –2.4; –7.3; –8.1; –10.9; –11.6; –12.6; –13.1; –14.4	72	67	69	69 ± 3	5	12	11	9 ± 4	77	79	80	79 ± 2	21	19	17	19 ± 2	0	0	0	0	2
	Nutrient mixture F-12 Ham[f]	0; –2.4; –7.3; –8.1; –8.2; –11.5; –12.3; –13.0; –14.2; –14.4	78	78	78	78 ± 0	8	9	7	8 ± 1	86	87	85	86 ± 1	10	9	11	10 ± 1	0	0	0	0	4
7.5	0.1 M tris-HCl[g] pH 7.5	0; –7.5; –8.1; –8.3; –11.0; –11.5; –12.3; –12.5; –13.0; –14.3	66	70	67	68 ± 2	5	10	8	8 ± 3	71	80	75	75 ± 5	25	19	23	22 ± 3	0	0	0	0	3
Moderately alkaline environment 8 ≤ pH ≤ 8.6																							
8	0.1 M Sodium phosphate (HPO_4^{2-} /$H_2PO_4^-$) pH 8	4; 3.3; 1.2; –2.4; –6.8; –7.3; –8.0; –9.3; –12.3; –12.7; –14.4	0	4	3	2 ± 2	0	2	0	1 ± 1	0	6	3	3 ± 3	67	64	70	67 ± 3	23	25	27	25 ± 2	5
	0.1 M HEPES pH 8	1.5; 0.8; –7.3; –8.0; –10.9; –12.4;	0	0	0	0	0	0	0	0	0	0	0	0	95	97	92	95 ± 3	0	0	0	0	5

		–12.7; –13.9; –14.4																					
	0.1 M tris-HCl pH 8	0.1; –7.5; –8.1; –8.3; –11.0; –11.5; –12.3; –12.5; –13.0; –13.9; –14.3; –14.4	33	43	38	38 ± 5	5	3	7	5 ± 2	38	46	45	43 ± 4	60	52	51	54 ± 5	0	0	0	0	2
8.5	0.1 M tris-HCl pH 8.5	1; –2.4; –7.3; –8.1; –11.0; –11.7; –12.4; –12.6; –14.0; –14.1; –14.4	0	1	2	1 ± 1	0	0	0	0	0	1	2	1 ± 1	95	94	94	94 ± 1	0	0	0	0	5
8.6	0.1 M glycine-NaOH pH 8.6	0; –7.3; –11.5; –12.3; –13.0; –14.4	80	80	79	80 ± 1	10	10	11	10 ± 1	90	90	90	90 ± 0	10	10	10	10 ± 0	0	0	0	0	0
	0.2 M glycine-NaOH pH 8.6	0; –7.3; –10.9; –11.5; –12.3; –13.0; –14.4	60	57	58	58 ± 2	8	5	10	8 ± 3	68	62	68	66 ± 3	30	37	30	32 ± 4	0	0	0	0	2
	0.5 M glycine-NaOH pH 8.6	0; –2.4; –7.3; –8.0; –10.9; –11.5; –12.3; –13.0; –14.4	27	19	22	23 ± 4	3	2	3	3 ± 1	30	21	25	25 ± 5	67	75	70	71 ± 4	0	0	0	0	4

[a]SD – standard deviation; [b]MES – 2-(N-morpholino)ethanesulfonic acid, $C_6H_{13}NO_4S$ (Figure S1); [c]HEPES – 4-(2-hydroxyethyl)-1-piperazineethanesulfonic acid, $C_8H_{18}N_2O_4S$ (Figure S1); [d]PBS – phosphate buffer saline; [e]MHB – Mueller-Hinton broth, for more detailed information about composition see https://labmal.com/2019/11/20/mueller-hinton-agar-and-mueller-hinton-broth/; [f]Nutrient mixture F-12 Ham contains sodium pyruvate (0.11 g/L), phenol red, L-glutamine, and does not contain $NaHCO_3$ and HEPES, for more details please see https://www.sigmaaldrich.com/AT/en/technical-documents/technical-article/cell-culture-and-cell-culture-analysis/mammalian-cell-culture/F-12-ham; [g]tris – tris(hydroxymethyl)aminomethane, $C_4H_{11}NO_3$ (Figure S1).

Table S13. Analysis of NMR spectroscopic data recorded in *α/β* -P_2W_{18} solutions investigated after 24 h incubation at 37 °C.

Chemical shifts in ^{31}P NMR spectra measured in triplicate of **$(NH_4)_6$[*α/β*-$P^V{}_2W^{VI}{}_{18}O_{62}$]** (10 mM) dissolved D_2O and buffers (acetic acid – sodium acetate pH 4 – 5.5; sodium phosphate pH 3 – 8 (while phosphate does not buffer at pH range from 3.5 – 5.5, experiments were conducted at this pH to provide comparisons to previously published studies (*29*)); citric acid – sodium citrate pH 3 – 6.5; MES pH 5.5; PBS pH 7.4; tris-HCl pH 7.5 – 8.5; HEPES pH 7 – 8; MHB pH 7.4; Nutrient mixture F-12 Ham pH 7.4 and glycine-NaOH pH 8.6) with concentration 0.1 M and investigated after 24 h incubation at 37 °C. The glycine-NaOH buffer was used in two additional concentrations 0.2 and 0.5 M (**Figures S44** – **S52**). The species content was calculated based on the integration of 31P signals considering only signals associated with POTs. Signals were assigned based on the literature data summarized in **Table S4**.

**pH**	**$(NH_4)_6$[*α/β*-$P_2W_{18}O_{62}$]** (10 mM) in Solvent / Buffer / Medium	Chemical shifts δ ^{31}P [ppm]	% of *α*-P_2W_{18} in **$(NH_4)_6$[*α/β*-$P^V{}_2W^{VI}{}_{18}O_{62}$]** (10 mM) solution after 24 h incubation at 37 °C				% of *β*-P_2W_{18} in **$(NH_4)_6$[*α/β*-$P^V{}_2W^{VI}{}_{18}O_{62}$]** (10 mM) solution after 24 h incubation at 37 °C				% of two **P_2W_{18}** isomers in **$(NH_4)_6$[*α/β*-$P^V{}_2W^{VI}{}_{18}O_{62}$]** (10 mM) solution after 24 h incubation at 37 °C				% of P_2W_{17} in **$(NH_4)_6$[*α/β*-$P^V{}_2W^{VI}{}_{18}O_{62}$]** (10 mM) solution after 24 h incubation at 37 °C				% of P_2W_5 in **$(NH_4)_6$[*α/β*-$P^V{}_2W^{VI}{}_{18}O_{62}$]** (10 mM) solution after 24 h incubation at 37 °C				Average % of other POTs
			Sample			Mean of 1 to 3 ± SD[a]	Sample			Mean of 1 to 3 ± SD[a]	Sample			Mean of 1 to 3 ± SD[a]	Sample			Mean of 1 to 3 ± SD[a]	Sample			Mean of 1 to 3 ± SD[a]	
			#1	#2	#3		#1	#2	#3		#1	#2	#3		#1	#2	#3		#1	#2	#3		
	D_2O	0; –11.5; –12.3; –13.0	89	94	84	89 ± 5	11	6	16	11 ± 5	100	100	100	100 ± 0	0	0	0	0	0	0	0	0	0
Strongly acidic environment 3 ≤ pH ≤ 4																							
3	0.1 M Sodium phosphate ($H_2PO_4^-$ /H_3PO_4) pH 3	0; –11.5; –12.3; –13.0	80	81	89	83 ± 5	20	19	11	17 ± 5	100	100	100	100 ± 0	0	0	0	0	0	0	0	0	0
	0.1 M Citric acid – sodium citrate (H_3Cit/H_2Cit^-) pH 3	0; –11.5; –12.3; –13.1	85	91	90	87 ± 3	15	8	10	11 ± 4	100	99	100	100 ± 1	0	0	0	0	0	0	0	0	0
4	0.1 M Sodium phosphate ($H_2PO_4^-$ /H_3PO_4) pH 4	0; –11.5; –12.3; –13.0	78	84	75	79 ± 5	22	16	25	21 ± 5	100	100	100	100 ± 0	0	0	0	0	0	0	0	0	0
	0.1 M Citric acid – sodium citrate (H_2Cit^- /$HCit^{2-}$) pH 4	2.2; 0; –8.1; –9.6; –11.5; –12.3; –	87	83	86	85 ± 2	11	14	11	12 ± 2	98	97	97	97 ± 1	2	0	0	1 ± 1	0	0	0	0	2

		13.0; –13.9																					
	0.1 M Acetic acid – sodium acetate (OAc^-/HOAc) pH 4	0; –11.5; –12.3; –13.0	80	88	88	85 ± 5	20	12	12	15 ± 5	100	100	100	100 ± 0	0	0	0	0	0	0	0	0	0
Moderately acidic environment 5 ≤ pH ≤ 6																							
	0.1 M Sodium phosphate ($H_2PO_4^-$/H_3PO_4) pH 5	0; –11.5; –12.3; –13.0	85	91	86	87 ± 3	15	9	14	13 ± 3	100	100	100	100 ± 0	0	0	0	0	0	0	0	0	0
5	0.1 M Citric acid – sodium citrate (H_2Cit^-/$HCit^{2-}$) pH 5	2.5; 2.2; 0; –7.4; –7.8; –8.0; –9.5; –11.5; –12.3; –13.0; –13.8; –14.3	38	40	46	41 ± 4	6	4	3	4 ± 2	44	44	49	46 ± 3	46	48	47	47 ± 1	0	0	0	0	7
	0.1 M Acetic acid – sodium acetate (OAc^-/HOAc) pH 5	0; –7.3; –11.5; –12.3; –13.0; –14.4	91	92	90	91 ± 1	3	2	4	3 ± 1	94	94	94	94 ± 0	7	6	6	6 ± 1	0	0	0	0	0
5.5	0.1 M Acetic acid – sodium acetate (OAc^-/HOAc) pH 5.5	0; –2.4; –7.3; –10.9; –11.5; –12.3; –13.0; –14.4	69	68	68	68 ± 1	2	2	1	2 ± 1	71	70	69	70 ± 1	28	29	30	29 ± 1	0	0	0	0	1
	0.1 M MES[b] pH 5.5	0; –7.3; –11.5; –12.3; –13.0; –14.3	72	74	73	73 ±1	4	0	0	1 ± 2	76	74	73	74 ± 2	23	25	27	25 ± 2	0	0	0	0	1
6	0.1 M Sodium phosphate (HPO_4^{2-}/$H_2PO_4^-$) pH 6	0; –2.4; –7.3; –10.9; –11.5; –12.3; –13.0; –14.4	67	68	70	68 ± 2	2	2	4	3 ± 1	69	70	74	71 ± 3	28	28	24	27 ± 2	0	0	0	0	2

	0.1 M Citric acid – sodium citrate ($HCit^{2-}$/Cit^{3-}) pH 6	2.8; 2.5; 0.1; –7.2; –7.5; –8.0; –9.4; –12.3; –12.7; –13.0; –13.9; –14.4	1	1	1	1 ± 0	0	0	0	0	1	1	1	1 ± 0	75	77	82	78 ± 4	0	0	0	0	21
Neutral environment 6.5 ≤ pH ≤ 7.5																							
6.5	0.1 M Citric acid – sodium citrate ($HCit^{2-}$/Cit^{3-}) pH 6.5	3; 2.9; 2.5; 0.2; –7.2; –7.4; –8.0; –9.4; –12.3; –13.4; –14.5	0	0	0	0	0	0	0	0	0	0	0	0	83	84	87	85 ± 2	0	0	0	0	15
7	0.1 M Sodium phosphate (HPO_4^{2-}/$H_2PO_4^{-}$) pH 7	3.9; 3.2; 1.0; –2.4; –7.3; –9.4; –14.4	0	0	0	0	0	0	0	0	0	0	0	0	67	73	75	72 ± 4	23	25	19	22 ± 3	
	0.1 M HEPES[c] pH 7	0.1; –7.3; –10.9; –13.1; –13.9; –14.4	28	27	31	29 ± 2	0	0	0	0	28	27	31	29 ± 2	70	70	66	69 ± 2	0	0	0	0	2
7.4	PBS[d] pH 7.4	0; –7.3; –11.5; –12.3; –13.0; –14.4	86	87	81	85 ± 3	4	3	8	5 ± 3	90	90	89	90 ± 1	10	9	9	9 ± 1	0	0	0	0	0
	MHB[e] pH 7.4	0; –7.3; –8.1; –10.9; –13.1; –14.4	72	74	72	73 ± 1	0	0	0	0	72	74	72	73 ± 1	25	24	26	25 ± 1	0	0	0	0	2
	Nutrient mixture F-12 Ham[f]	0; –7.3; –8.1; –11.5; –12.3; –13.0; –13.4; –14.4	80	81	80	80 ± 1	3	5	3	4 ± 1	83	86	83	84 ± 2	12	9	12	11 ± 2	0	0	0	0	3

7.5	0.1 M tris-HCl[g] pH 7.5	0; –7.5; –8.3; –11.0; –13.0; –13.8; –14.1; –14.3; –14.4	71	74	74	73 ± 2	0	0	0	0	71	74	74	73 ± 2	26	25	25	25 ± 1	0	0	0	0	2
Moderately alkaline environment 8 ≤ pH ≤ 8.6																							
8	0.1 M Sodium phosphate (HPO_4^{2-}/$H_2PO_4^-$) pH 8	4; 3.3; 1.2; –2.4; –7.3; –8.0; –9.3; –10.9; –12.3; –13.9; –14.4	0	0	0	0	0	0	0	0	0	0	0	0	69	62	68	66 ± 4	19	24	17	20 ± 4	4
	0.1 M HEPES pH 8	1.5; 0.8; –7.3; –8.0; –10.9; –12.4; –12.7; –13.9; –14.4	0	0	0	0	0	0	0	0	0	0	0	0	99	99	98	99 ± 1	0	0	0	0	1
	0.1 M tris-HCl pH 8	0.1; –7.5; –8.1; –8.3; –11.0; –13.1; –13.9; –14.3; –14.4	31	36	39	35 ±4	0	0	0	0	31	36	39	35 ±4	64	59	57	60 ±4	0	0	0	0	5
8.5	0.1 M tris-HCl pH 8.5	1; –2.4; –7.3; –8.1; –11.0; –14.0; –14.1; –14.4	0	0	0	0	0	0	0	0	0	0	0	0	95	95	95	95 ± 0	0	0	0	0	5
8.6	0.1 M glycine-NaOH pH 8.6	0; –7.3; –10.9; –11.5; –12.3; –13.0; –14.4	88	89	88	88 ± 1	2	2	2	2 ± 0	90	91	90	90 ± 1	9	9	9	9 ± 0	0	0	0	0	1
	0.2 M glycine-NaOH pH 8.6	0; –7.3; –10.9; –	67	61	68	65 ± 4	0	0	1	0	67	61	69	66 ± 4	31	38	32	34 ±	0	0	0	0	0

		**13.0**; **–14.4**																				
	0.5 M glycine-NaOH pH 8.6	0; **–7.3**; –8.0; –10.9; **–13.0**; **–14.4**	31	15	21	22 ± 8	0	0	0	0	31	15	21	22 ± 8	66	81	75	74 ± 8	0	0	0	0	4

[a]SD – standard deviation; [b]MES – 2-(N-morpholino)ethanesulfonic acid, $C_6H_{13}NO_4S$ (Figure S1); [c]HEPES – 4-(2-hydroxyethyl)-1-piperazineethanesulfonic acid, $C_8H_{18}N_2O_4S$ (Figure S1); [d]PBS – phosphate buffer saline; [e]MHB – Mueller-Hinton broth, for more detailed information about composition, see https://labmal.com/2019/11/20/mueller-hinton-agar-and-mueller-hinton-broth/; [f]Nutrient mixture F-12 Ham contains sodium pyruvate (0.11 g/L), phenol red, L-glutamine, and does not contain $NaHCO_3$ and HEPES, for more details please see https://www.sigmaaldrich.com/AT/en/technical-documents/technical-article/cell-culture-and-cell-culture-analysis/mammalian-cell-culture/f-12-ham; [g]tris – tris(hydroxymethyl)aminomethane, $C_4H_{11}NO_3$ (Figure S1).

7.4. Speciation in $K_6[\alpha\text{-}P^{V}_2W^{VI}_{18}O_{62}]$ Wells-Dawson POT solutions

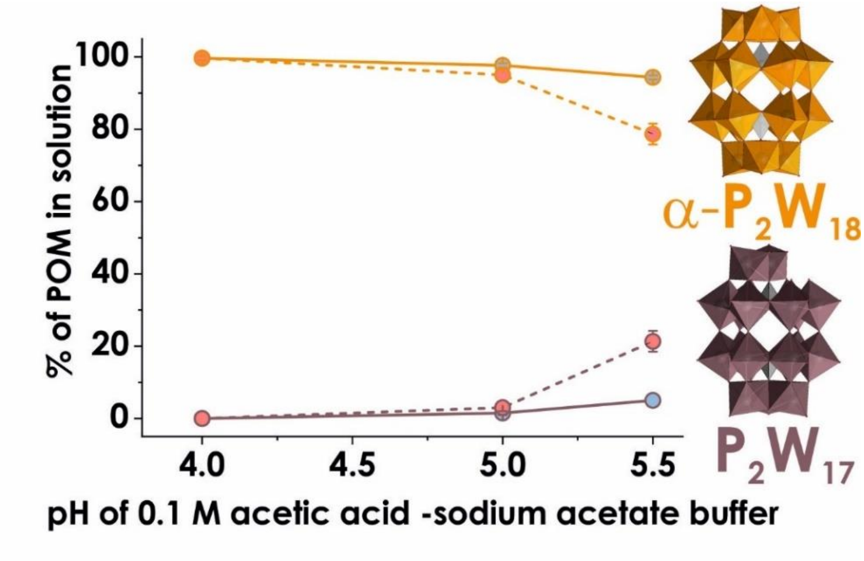

Fig. S53. Speciation of α-P_2W_{18} in acetic acid – sodium acetate buffer.

POM concentration curves in **$K_6[\alpha\text{-}P^{V}_2W^{VI}_{18}O_{62}]$** (10 mM) in 0.1 M acetic acid – sodium acetate buffer solutions before (solid line, blue dot in the middle) and after incubation (dash line, red dot in the middle) for 24 h at 37 °C. The exact percentage of all POM species present is given in **Supplementary Tables S10** and **S11**.

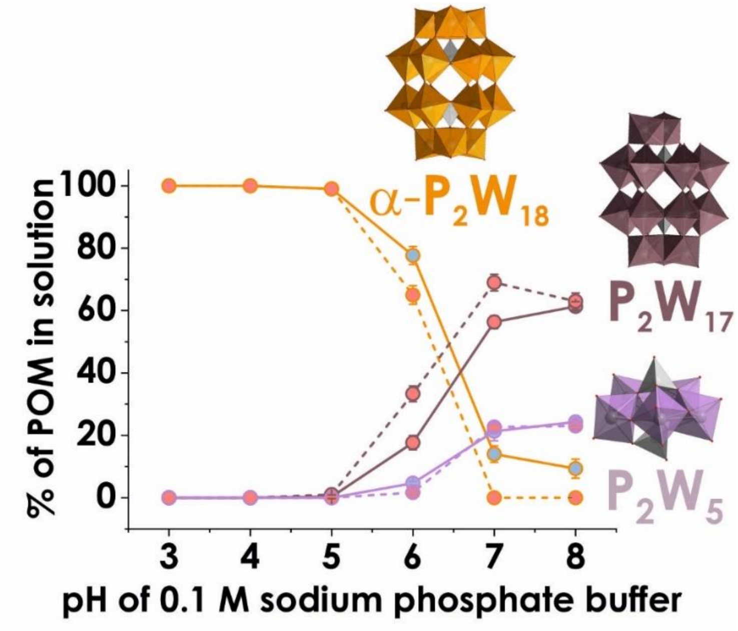

Fig. S54. Speciation of α-P_2W_{18} in sodium phosphate buffer.

POM concentration curves in **$K_6[\alpha\text{-}P^{V}_2W^{VI}_{18}O_{62}]$** (10 mM) in 0.1 M sodium phosphate buffer solutions before (solid line, blue dot in the middle) and after incubation (dash line, red dot in the middle) for 24 h at 37 °C. The exact percentage of all POM species present is given in **Supplementary Tables S10** and **S11**.

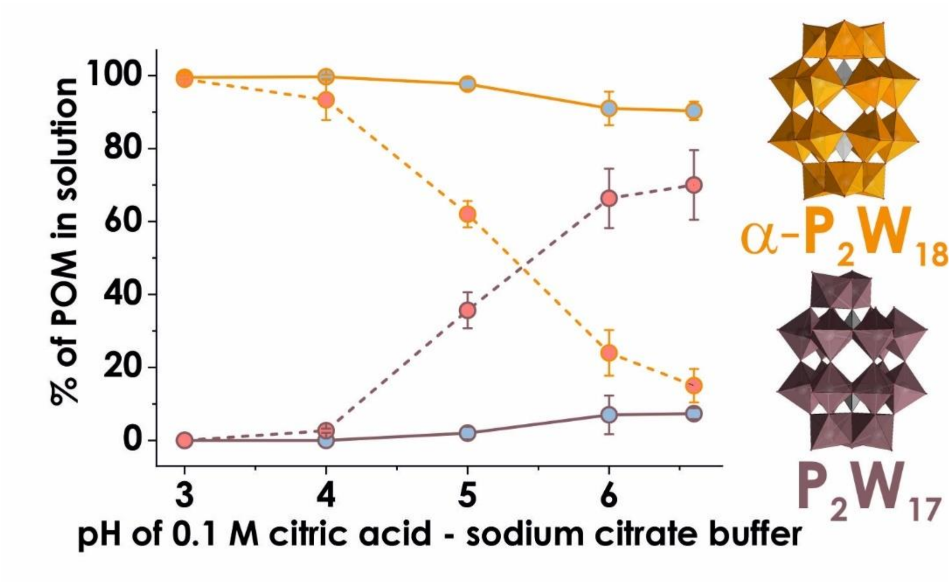

Fig. S55. Speciation of α-P_2W_{18} in citric acid – sodium citrate buffer.

POM concentration curves in **$K_6[\alpha\text{-}P^{V}_2W^{VI}_{18}O_{62}]$** (10 mM) in 0.1 M citric acid – sodium citrate buffer solutions before (solid line, blue dot in the middle) and after incubation (dash line, red dot in the middle) for 24 h at 37 °C. The exact percentage of all POM species present is given in **Supplementary Tables S10** and **S11**.

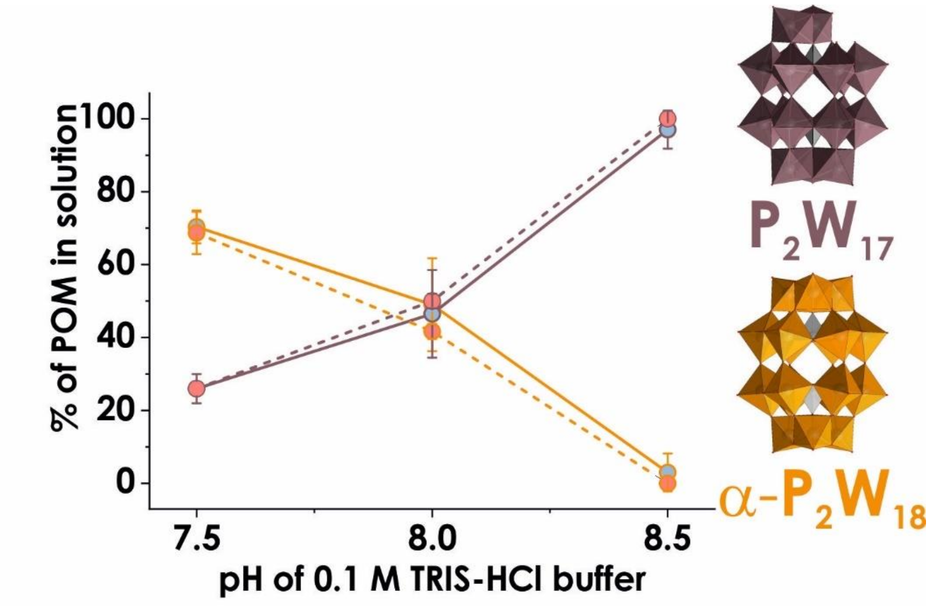

Fig. S56. Speciation of α-P_2W_{18} in TRIS-HCl buffer.

POM concentration curves in **$K_6[\alpha\text{-}P^V_2W^{VI}_{18}O_{62}]$** (10 mM) in 0.1 M tris-HCl buffer solutions before (solid line, blue dot in the middle) and after incubation (dash line, red dot in the middle) for 24 h at 37 °C. The exact percentage of all POM species present is given in **Supplementary Tables S10** and **S11**.

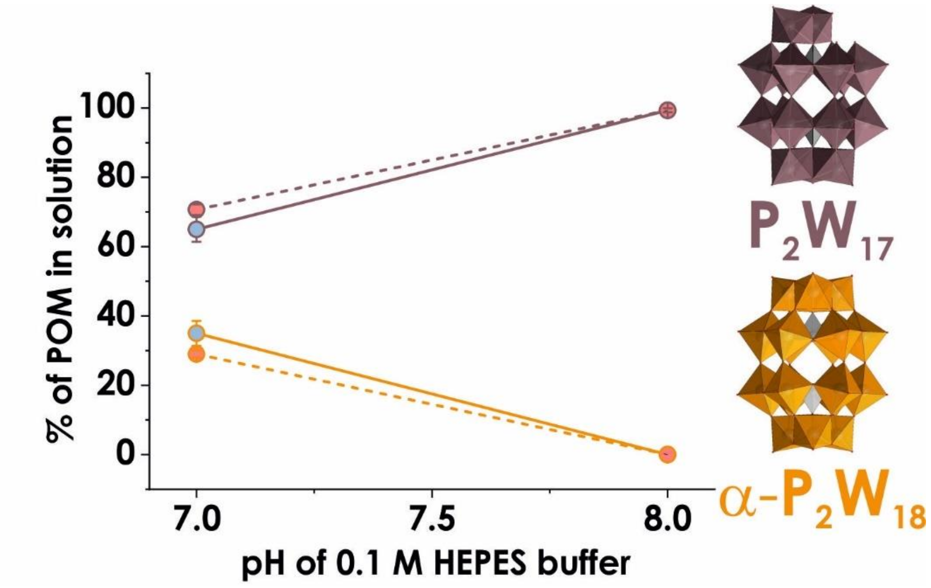

Fig. S57. Speciation of α-P_2W_{18} in HEPES buffer.

POM concentration curves in **$K_6[\alpha\text{-}P^{V}_2W^{VI}_{18}O_{62}]$** (10 mM) in 0.1 M HEPES buffer solutions before (solid line, blue dot in the middle) and after incubation (dash line, red dot in the middle) for 24 h at 37 °C. The exact percentage of all POM species present is given in **Supplementary Tables S10** and **S11**.

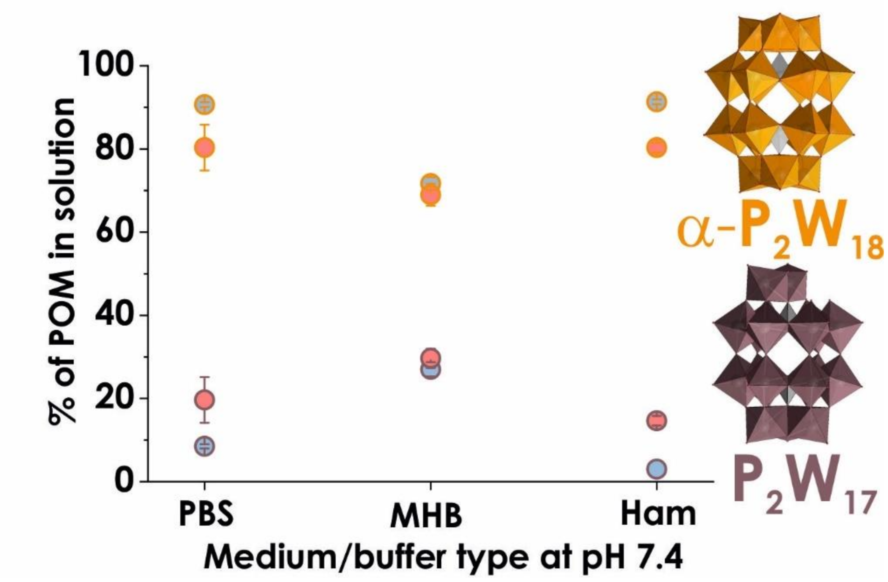

Fig. S58. Speciation of α-P_2W_{18} in solutions with pH 7.4.

POM concentration curves in **$K_6[\alpha\text{-}P^V{}_2W^{VI}{}_{18}O_{62}]$** (10 mM) in 0.1 M PBS, MHB and nutrient mixture F-12 Ham solutions before (blue dot in the middle) and after incubation (red dot in the middle) for 24 h at 37 °C. The exact percentage of all POM species present is given in **Supplementary Tables S10** and **S11**.

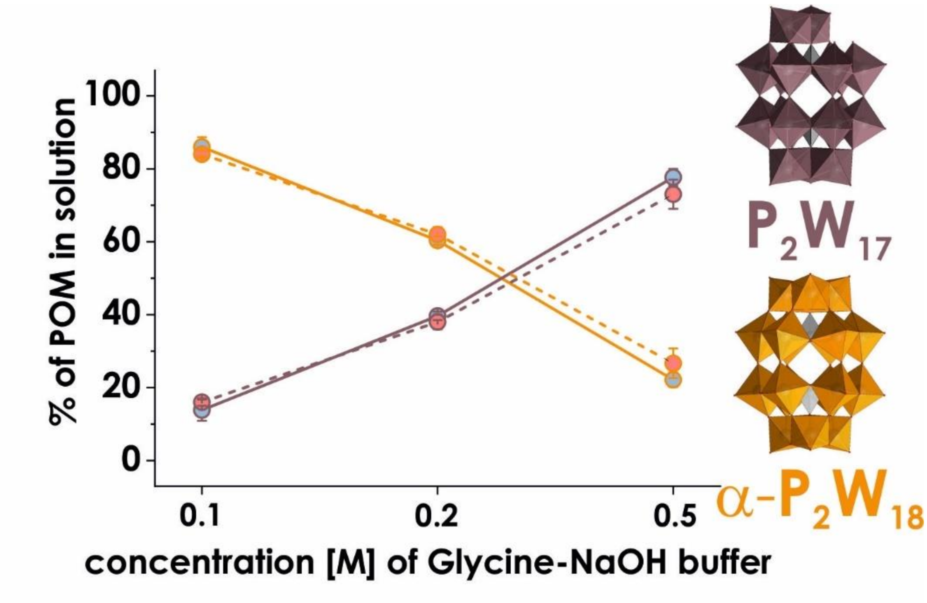

Fig. S59. Speciation of α-P_2W_{18} in glycine-NaOH buffer.

POM concentration curves in **$K_6[\alpha\text{-}P^V_2W^{VI}_{18}O_{62}]$** (10 mM) in glycine-NaOH (pH 8.6) with concentrations of 0.1. 0.2 and 0.5 M solutions before (solid line, blue dot in the middle) and after incubation (dash line, red dot in the middle) for 24 h at 37 °C. The exact percentage of all POM species present is given in **Supplementary Tables S10** and **S11**.

7.5. Speciation in Wells-Dawson $(NH_4)_6[\alpha/\beta\text{-}P^{V}_2W^{VI}_{18}O_{62}]$ POT solutions

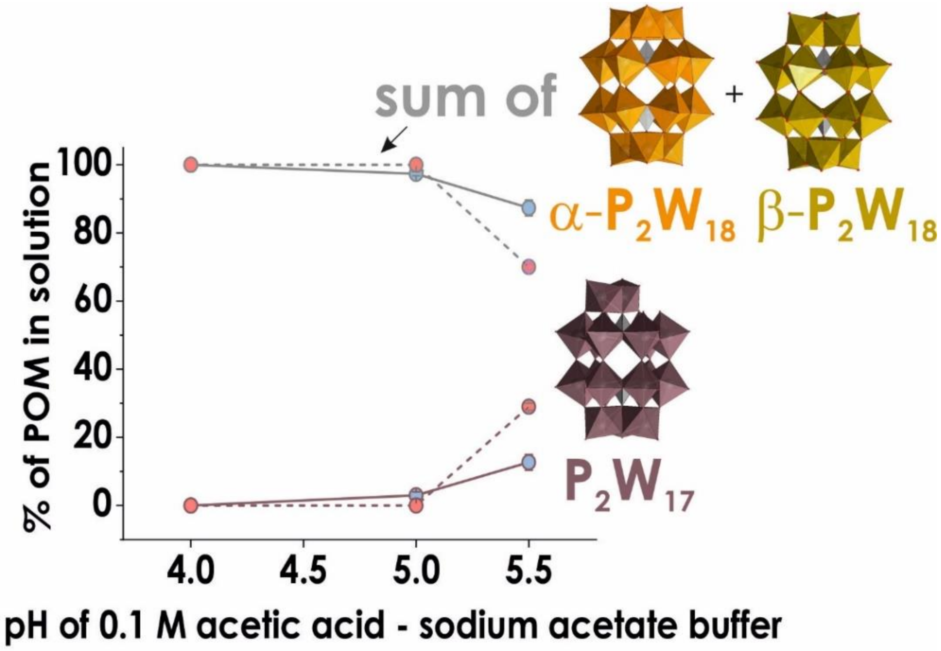

Fig. S60. Speciation of *α/β* -P_2W_{18} in acetic acid – sodium acetate buffer.

POM concentration curves in **$(NH_4)_6[\alpha/\beta\text{-}P^{V}_2W^{VI}_{18}O_{62}]$** (10 mM) in 0.1 M acetic acid – sodium acetate buffer solutions before (solid line, blue dot in the middle) and after incubation (dash line, red dot in the middle) for 24 h at 37 °C. The exact percentage of all POM species present is given in **Supplementary Tables S12** and **S13**.

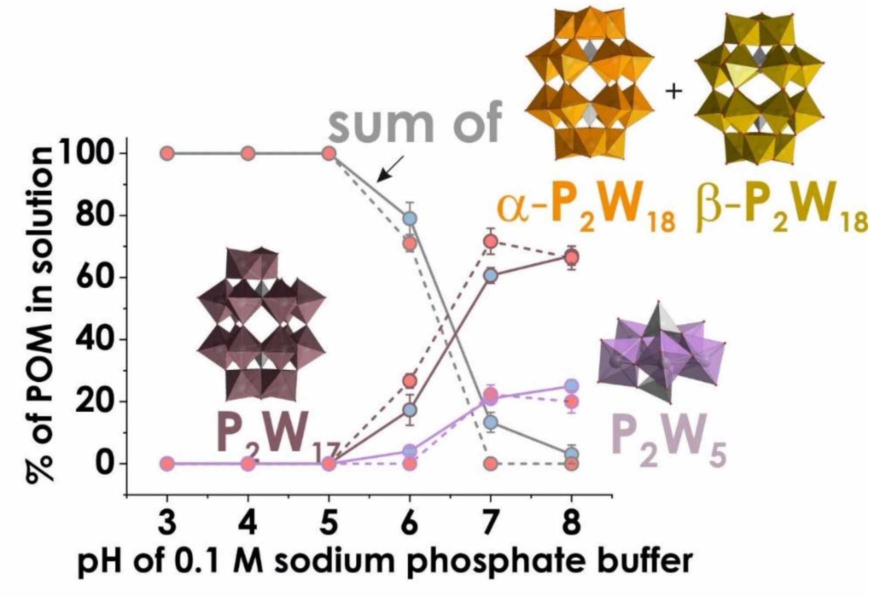

Fig. S61. Speciation of *α/β* -P_2W_{18} in sodium phosphate buffer.

POM concentration curves in **$(NH_4)_6$[*α/β*-$P^V{}_2W^{VI}{}_{18}O_{62}$]** (10 mM) in 0.1 M sodium phosphate buffer solutions before (solid line, blue dot in the middle) and after incubation (dash line, red dot in the middle) for 24 h at 37 °C. The exact percentage of all POM species present is given in **Supplementary Tables S12** and **S13**.

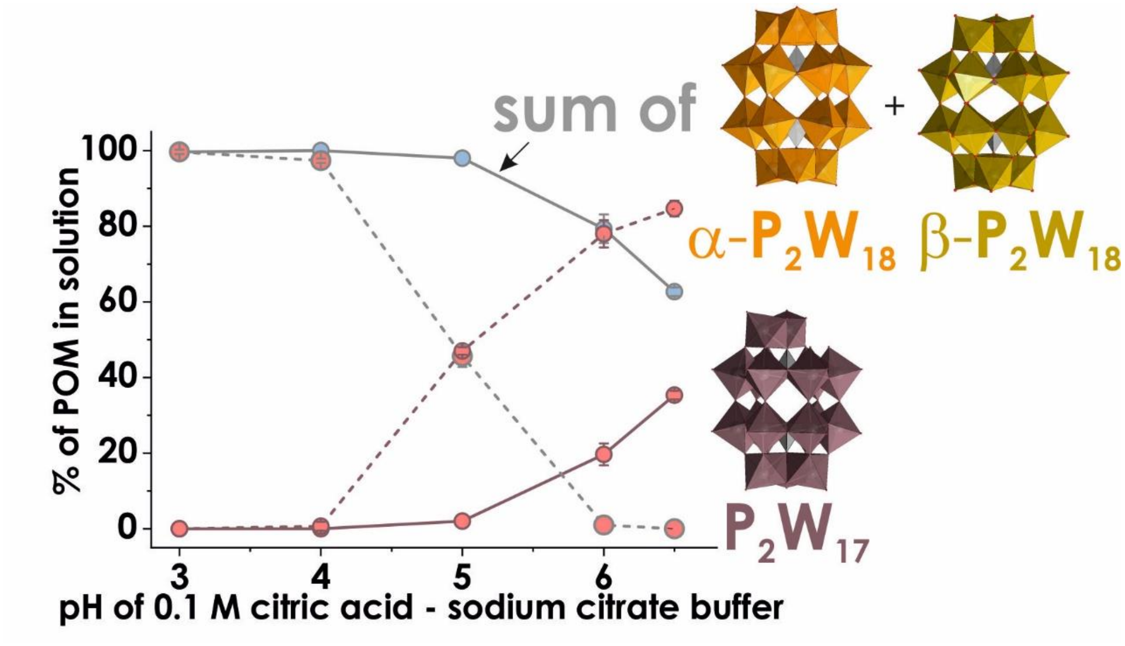

Fig. S62. Speciation of *α/β* -P_2W_{18} in citric acid – sodium citrate buffer.

POM concentration curves in **$(NH_4)_6$[*α/β*-$P^V{}_2W^{VI}{}_{18}O_{62}$]** (10 mM) in 0.1 M citric acid – sodium citrate buffer solutions before (solid line, blue dot in the middle) and after incubation (dash line, red dot in the middle) for 24 h at 37 °C. The exact percentage of all POM species present is given in **Supplementary Tables S12** and **S13**.

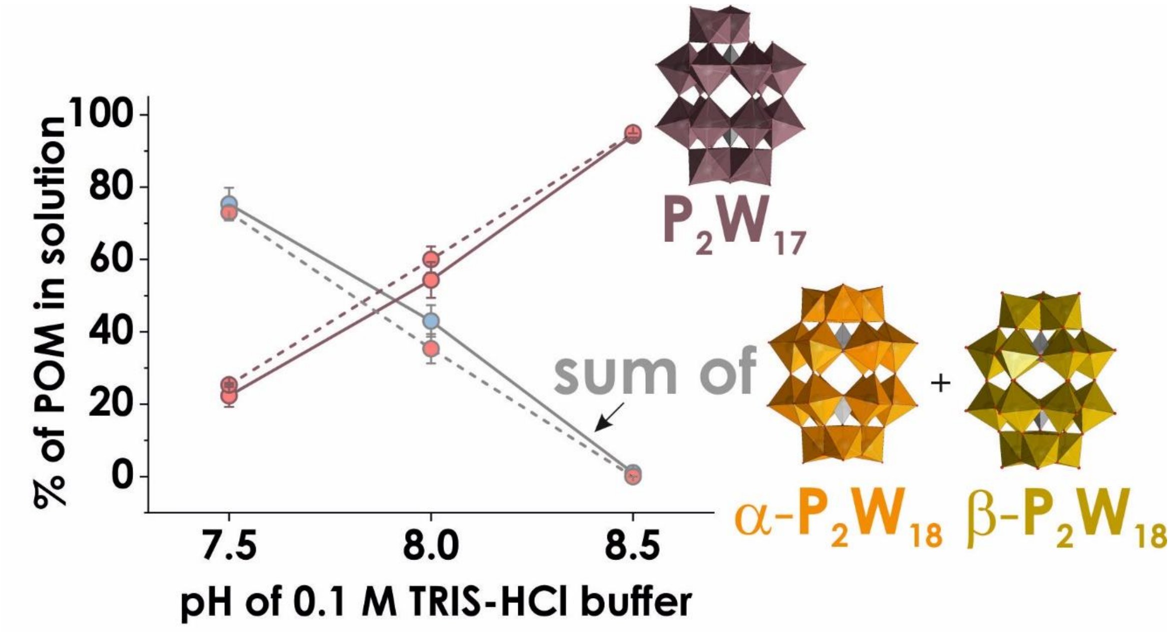

Fig. S63. Speciation of *α/β* -P_2W_{18} in tris-HCl buffer.

POM concentration curves in **$(NH_4)_6$[*α/β*-$P^V{}_2W^{VI}{}_{18}O_{62}$]** (10 mM) in 0.1 M tris-HCl buffer solutions before (solid line, blue dot in the middle) and after incubation (dash line, red dot in the middle) for 24 h at 37 °C. The exact percentage of all POM species present is given in **Supplementary Tables S12** and **S13**.

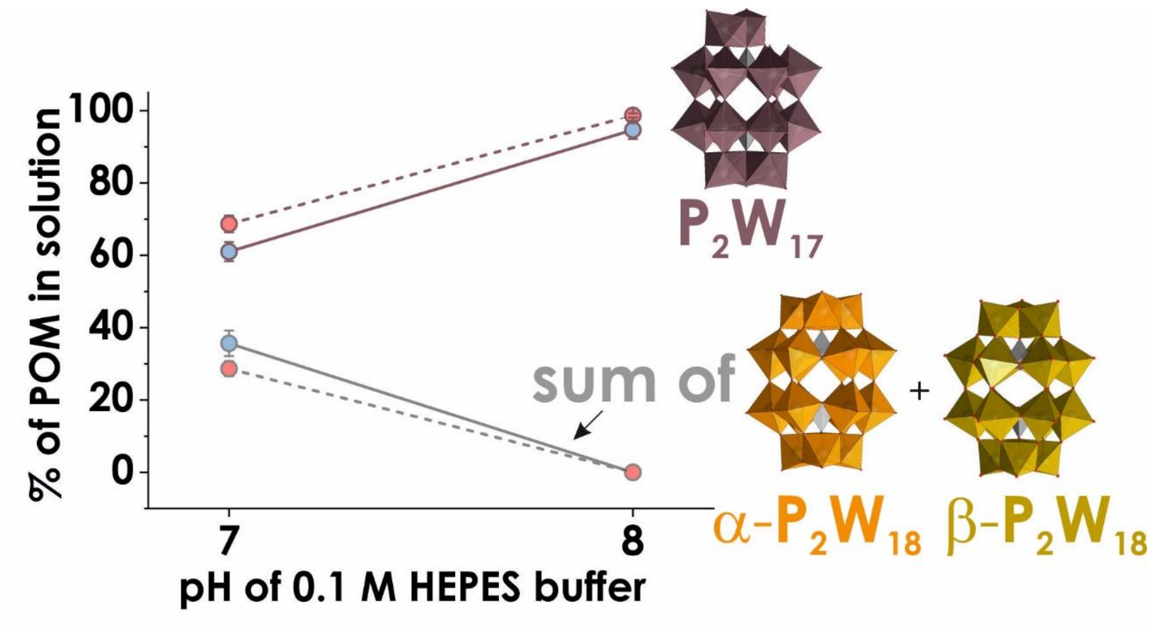

Fig. S64. Speciation of *α/β* -P_2W_{18} in HEPES buffer.

POM concentration curves in **$(NH_4)_6$[*α/β*-$P^V{}_2W^{VI}{}_{18}O_{62}$]** (10 mM) in 0.1 M HEPES buffer solutions before (solid line, blue dot in the middle) and after incubation (dash line, red dot in the middle) for 24 h at 37 °C. The exact percentage of all POM species present is given in **Supplementary Tables S12** and **S13**.

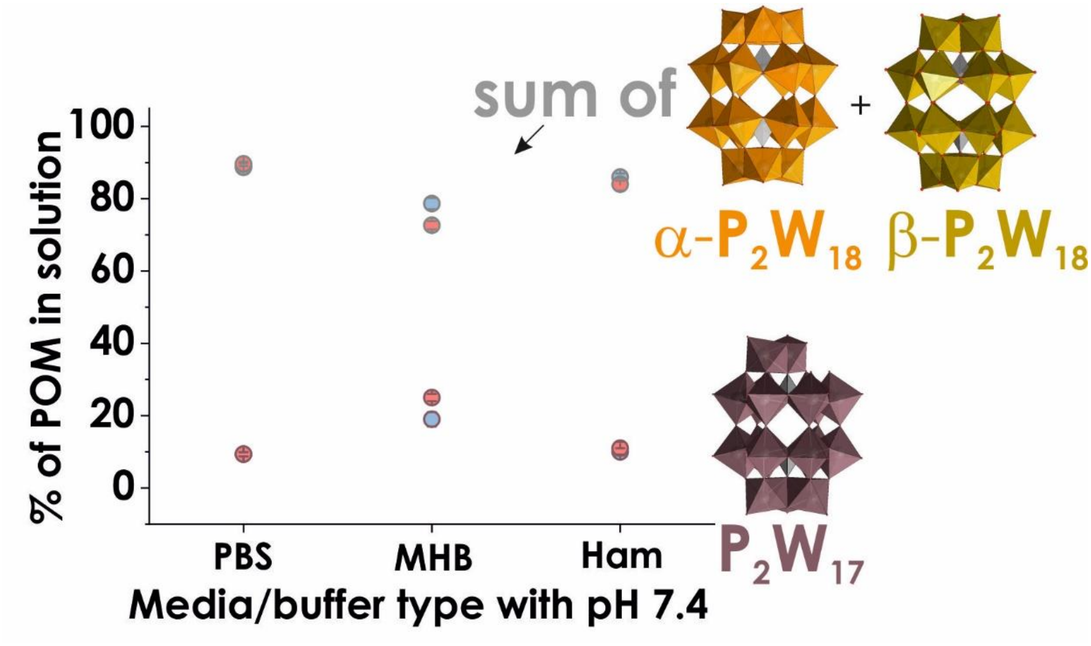

Fig. S65. Speciation of *α/β* -P_2W_{18} in solutions with pH 7.4.

POM concentration curves in **$(NH_4)_6[$*α/β*-$P^V_2W^{VI}_{18}O_{62}]$** (10 mM) in in 0.1 M PBS, MHB and nutrient mixture F-12 Ham solutions before (blue dot in the middle) and after incubation (red dot in the middle) for 24 h at 37 °C. The exact percentage of all POM species present is given in **Supplementary Tables S12** and **S13**.

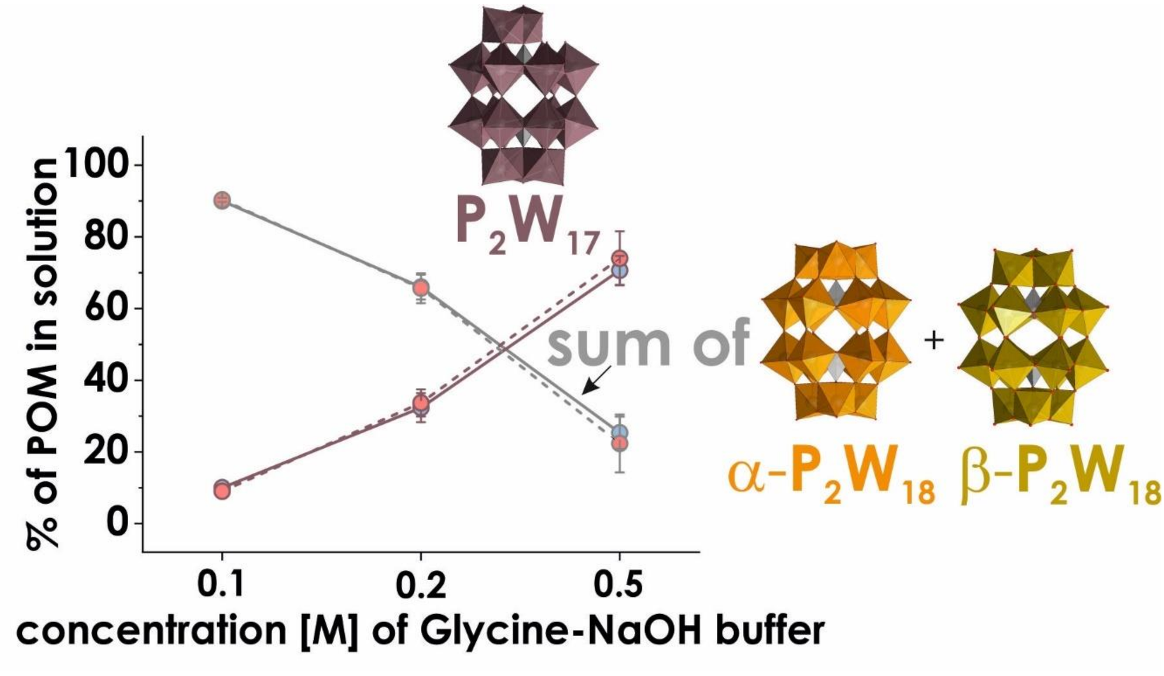

Fig. S66. Speciation of *α/β* -P_2W_{18} in glycine-NaOH buffer.

POM concentration curves in **$(NH_4)_6$[*α/β*-$P^V{}_2W^{VI}{}_{18}O_{62}$]** (10 mM) in glycine-NaOH (pH 8.6) with concentrations of 0.1. 0.2 and 0.5 M solutions before (solid line, blue dot in the middle) and after incubation (dash line, red dot in the middle) for 24 h at 37 °C. The exact percentage of all POM species present is given in **Supplementary Tables S12** and **S13**.

8. The P_5W_{30} Preyssler POT

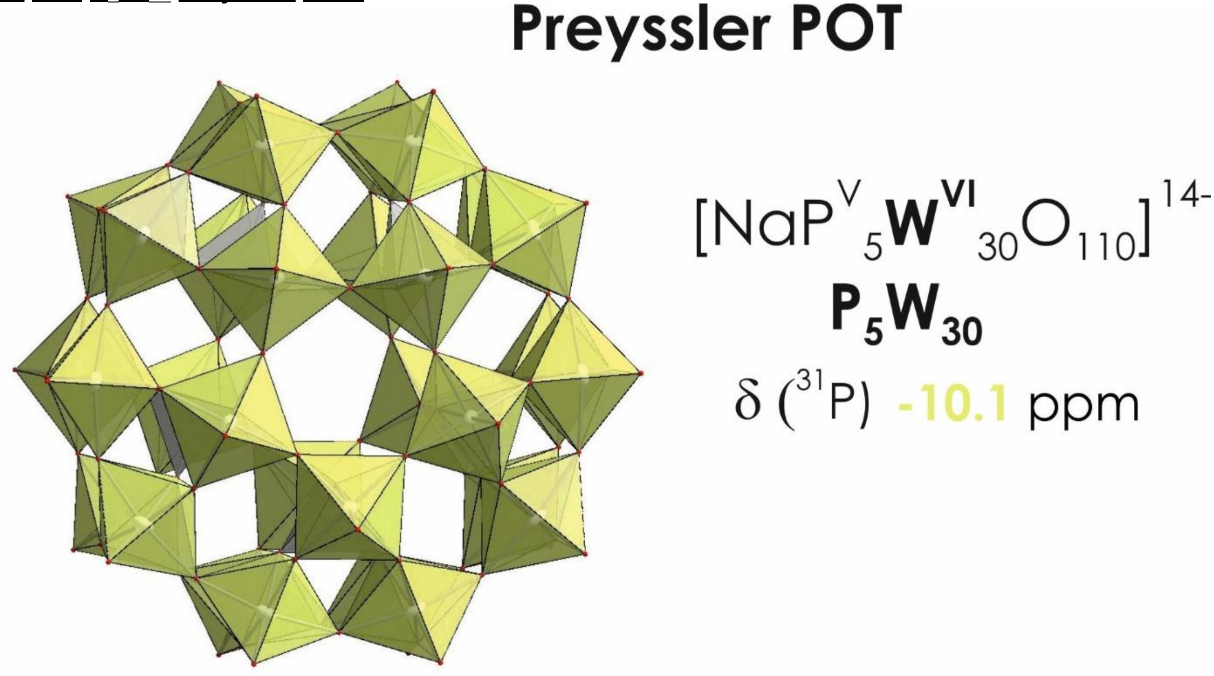

Fig. S67. Preyssler type POT.

Structure of the Preyssler POT $[NaP^{V}{}_5W^{VI}{}_{30}O_{110}]^{14-}$ (**P_5W_{30}**), indicating ^{31}P NMR chemical shift. Color code: $\{WO_6\}$, light green; $\{PO_4\}$, gray; O, red, Na omitted for clarity.

8.1. pH of Preyssler POT solutions

Table S14. pH in P_5W_{30} solutions.

pH values measured in triplicate of **$K_{12.5}Na_{1.5}[NaP^{V}_5W^{VI}_{30}O_{114}]$** (10 mM) dissolved D_2O and different buffers (acetic acid – sodium acetate pH 4 – 5.5; sodium phosphate pH 3 – 8 (while phosphate does not buffer at pH range from 3.5 – 5.5, experiments were conducted at this pH to provide comparisons to previously published studies (*29*)); citric acid – sodium citrate pH 3 – 6.5; MES pH 5.5; PBS pH 7.4; tris-HCl pH 7.5 – 8.5; HEPES pH 7 – 8; MHB pH 7.4; Nutrient mixture F-12 Ham pH 7.4 and glycine-NaOH pH 8.6) with concentration 0.1 M. The glycine-NaOH buffer was used in two additional concentrations 0.2 and 0.5 M.

pH	**$K_{14}[NaP_5W_{30}O_{110}]$** (10 mM) in Solvent / Buffer / Medium	pH after dissolving **$K_{12.5}Na_{1.5}[NaP^{V}_5W^{VI}_{30}O_{114}]$** (10 mM) at RT				pH after 24 h incubation of **$K_{12.5}Na_{1.5}[NaP^{V}_5W^{VI}_{30}O_{114}]$** (10 mM) solution at 37 °C			
		Sample			Mean of 1 to 3 ± SD[a]	Sample			Mean of 1 to 3 ± SD[a]
		#1	#2	#3		#1	#2	#3	
-	D_2O	2.71	2.83	3.44	2.99 ± 0.39	2.50	2.71	3.42	2.88 ± 0.48
Strongly acidic environment 3 ≤ pH ≤ 4									
3	0.1 M Sodium phosphate ($H_2PO_4^-$/H_3PO_4) pH 3	2.96	2.98	2.83	2.92 ± 0.08	2.92	2.97	2.81	2.90 ± 0.08
	0.1 M Citric acid – sodium citrate (H_3Cit/H_2Cit^-) pH 3	2.84	2.88	2.88	2.87 ± 0.02	2.66	2.83	2.64	2.71 ± 0.10
4	0.1 M Sodium phosphate ($H_2PO_4^-$/H_3PO_4) pH 4	3.84	3.92	3.97	3.91 ± 0.07	3.49	3.90	3.82	3.74 ± 0.22
	0.1 M Citric acid – sodium citrate (H_2Cit^-/$HCit^{2-}$) pH 4	3.83	3.89	3.72	3.81 ± 0.09	3.62	3.84	3.62	3.69 ± 0.13
	0.1 M Acetic acid – sodium acetate (OAc^-/HOAc) pH 4	3.91	3.91	3.82	3.88 ± 0.05	3.89	3.86	3.92	3.89 ± 0.03
Moderately acidic environment 5 ≤ pH ≤ 6									
5	0.1 M Sodium phosphate ($H_2PO_4^-$/H_3PO_4) pH 5	4.76	4.94	4.84	4.85 ± 0.09	4.62	4.89	4.82	4.78 ± 0.14
	0.1 M Citric acid – sodium citrate (H_2Cit^-/$HCit^{2-}$) pH 5	4.88	4.91	4.78	4.86 ± 0.07	4.68	4.85	4.68	4.74 ± 0.10
	0.1 M Acetic acid – sodium acetate (OAc^-/HOAc) pH 5	4.92	4.95	4.78	4.88 ± 0.09	4.91	4.91	4.85	4.89 ± 0.03

5.5	0.1 M Acetic acid – sodium acetate ($OAc^-/HOAc$) pH 5.5	5.43	5.46	5.36	5.42 ± 0.05	5.39	5.39	5.37	5.38 ± 0.01
	0.1 M MES[b] pH 5.5	5.78	5.43	5.54	5.58 ± 0.18	5.61	5.32	5.63	5.52 ± 0.17
6	0.1 M Sodium phosphate ($HPO_4^{2-}/H_2PO_4^-$) pH 6	5.87	5.99	5.85	5.90 ± 0.08	5.82	5.96	5.86	5.88 ± 0.07
	0.1 M Citric acid – sodium citrate ($HCit^{2-}/Cit^{3-}$) pH 6	5.87	5.97	5.85	5.90 ± 0.06	5.75	5.85	5.75	5.78 ± 0.06
Neutral environment 6.5 ≤ pH ≤ 7.5									
6.5	0.1 M Citric acid – sodium citrate ($HCit^{2-}/Cit^{3-}$) pH 6.5	6.26	6.49	6.39	6.38 ± 0.12	6.27	6.50	6.31	6.36 ± 0.12
7	0.1 M Sodium phosphate ($HPO_4^{2-}/H_2PO_4^-$) pH 7	7.13	7.08	6.98	7.06 ± 0.08	6.93	7.05	6.99	6.99 ± 0.06
	0.1 M HEPES[c] pH 7	7.19	7.13	7.14	7.15 ± 0.03	7.10	7.08	7.18	7.12 ± 0.05
7.4	PBS[d] pH 7.4	7.31	7.14	7.11	7.19 ± 0.11	6.92	6.97	6.71	6.87 ± 0.14
	MHB[e] pH 7.4	7.00	7.08	7.07	7.05 ± 0.04	6.67	7.08	6.63	6.79 ± 0.25
	Nutrient mixture F-12 Ham[f]	6.70	6.65	6.63	6.66 ± 0.04	5.01	4.95	4.93	4.96 ± 0.04
7.5	0.1 M tris-HCl[g] pH 7.5	7.29	7.30	7.44	7.34 ± 0.08	7.20	7.11	7.39	7.23 ± 0.14
Moderately alkaline environment 8 ≤ pH ≤ 8.6									
8	0.1 M Sodium phosphate ($HPO_4^{2-}/H_2PO_4^-$) pH 8	7.73	7.90	7.80	7.81 ± 0.09	7.40	7.80	7.75	7.65 ± 0.22
	0.1 M HEPES pH 8	8.12	8.00	8.08	8.07 ± 0.06	7.93	7.95	8.10	7.99 ± 0.09
	0.1 M tris-HCl pH 8	7.68	7.52	7.66	7.62 ± 0.09	7.58	7.34	7.63	7.52 ± 0.16
8.5	0.1 M tris-HCl pH 8.5	8.31	8.26	8.28	8.28 ± 0.03	8.16	8.06	8.28	8.17 ± 0.11
8.6	0.1 M glycine-NaOH pH 8.6	8.14	8.22	8.05	8.13 ± 0.08	7.73	7.84	7.75	7.77 ± 0.06
	0.2 M glycine-NaOH pH 8.6	8.34	8.35	8.17	8.29 ± 0.10	8.02	8.14	7.97	8.04 ± 0.09
	0.5 M glycine-NaOH pH 8.6	8.43	8.32	8.23	8.33 ± 0.10	8.00	8.19	8.05	8.08 ± 0.10

[a]SD – standard deviation; [b]MES – 2-(N-morpholino)ethanesulfonic acid, $C_6H_{13}NO_4S$ (Figure S1); [c]HEPES – 4-(2-hydroxyethyl)-1-piperazineethanesulfonic acid, $C_8H_{18}N_2O_4S$ (Figure S1); [d]PBS – phosphate buffer saline; [e]MHB – Mueller-Hinton broth, for more detailed information about composition, see https://labmal.com/2019/11/20/mueller-hinton-agar-and-mueller-hinton-broth/; [f]Nutrient mixture F-12 Ham contains sodium pyruvate (0.11 g/L), phenol red, L-glutamine, and does not contain $NaHCO_3$ and HEPES, for more details please see https://www.sigmaaldrich.com/AT/en/technical-documents/technical-article/cell-culture-and-cell-culture-analysis/mammalian-cell-culture/f-12-ham; [g]tris – tris(hydroxymethyl)aminomethane, $C_4H_{11}NO_3$ (Figure S1).

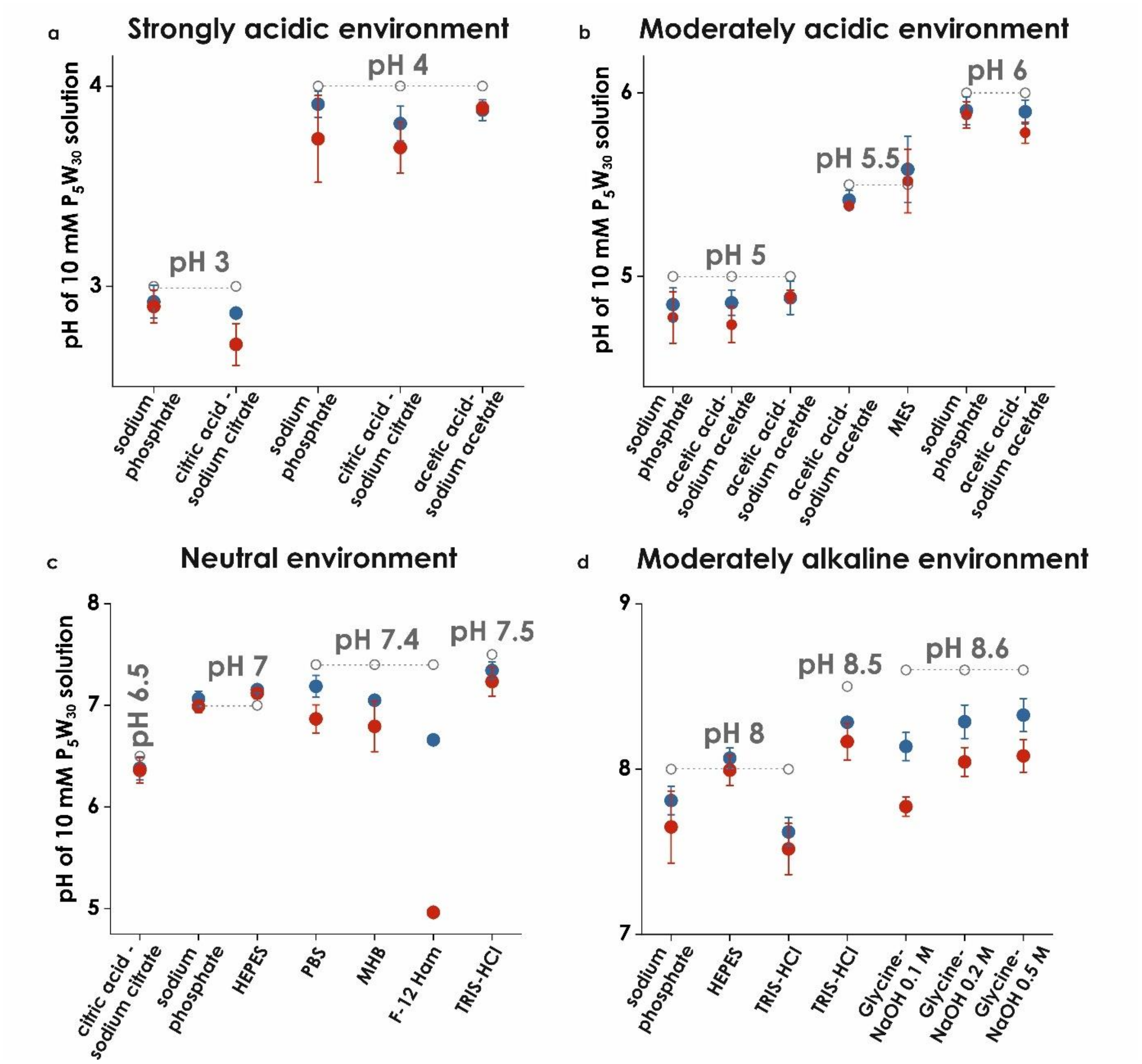

Fig. S68. pH in P_5W_{30} solutions.

The pH dependence curves for 10 mM solutions of **$K_{12.5}Na_{1.5}[NaP^{V}_5W^{VI}_{30}O_{114}]$** in different buffers in a) strongly acidic, b) moderately acidic, c) neutral, and d) moderately alkaline environments. The pH of the starting buffers is shown with a gray dashed line; the plots of the measured pH values immediately after the preparation of the solutions are shown in blue and after 24 h incubation at 37 °C in red. The error bar shows standard deviation (**Table S14**).

8.2. ^{31}P NMR spectroscopic studies of Preyssler POT solutions

All ^{31}P and ^{183}W peaks with the highest intensity were unambiguously assigned based on the literature data from **Table S4**. In some spectra, chemical shifts for peaks of lower intensity have not yet been described in the literature and therefore can not be assigned in this work.

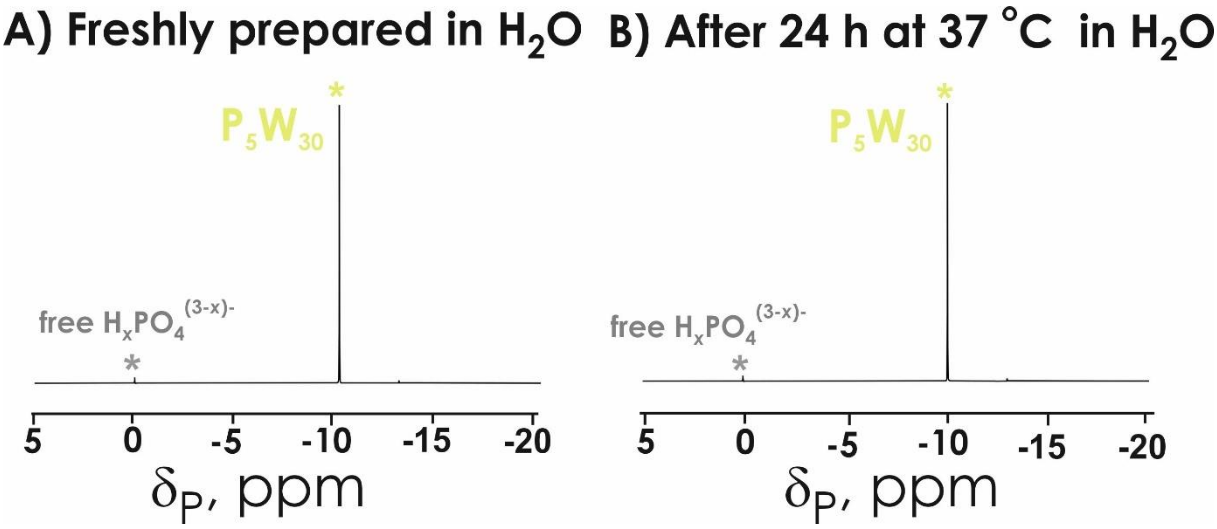

Fig. S69. ^{31}P NMR spectra of P_5W_{30} in H_2O.

^{31}P NMR spectra for 10 mM solutions of **$K_{12.5}Na_{1.5}[NaP^{V}_{5}W^{VI}_{30}O_{114}]$** in H_2O that were recorded approximately one hour after preparation (**A**) and after incubation for 24 h at 37 °C (**B**). The structures of all POMs are shown in **Figure S67**. The chemical shifts and percentages of parent and formed species are given in **Tables S15** and **S16**. To identify the individual anions, they are shown in different colors, with the same color code being selected for a specific anion throughout all figures and tables in the main manuscript and the supporting information.

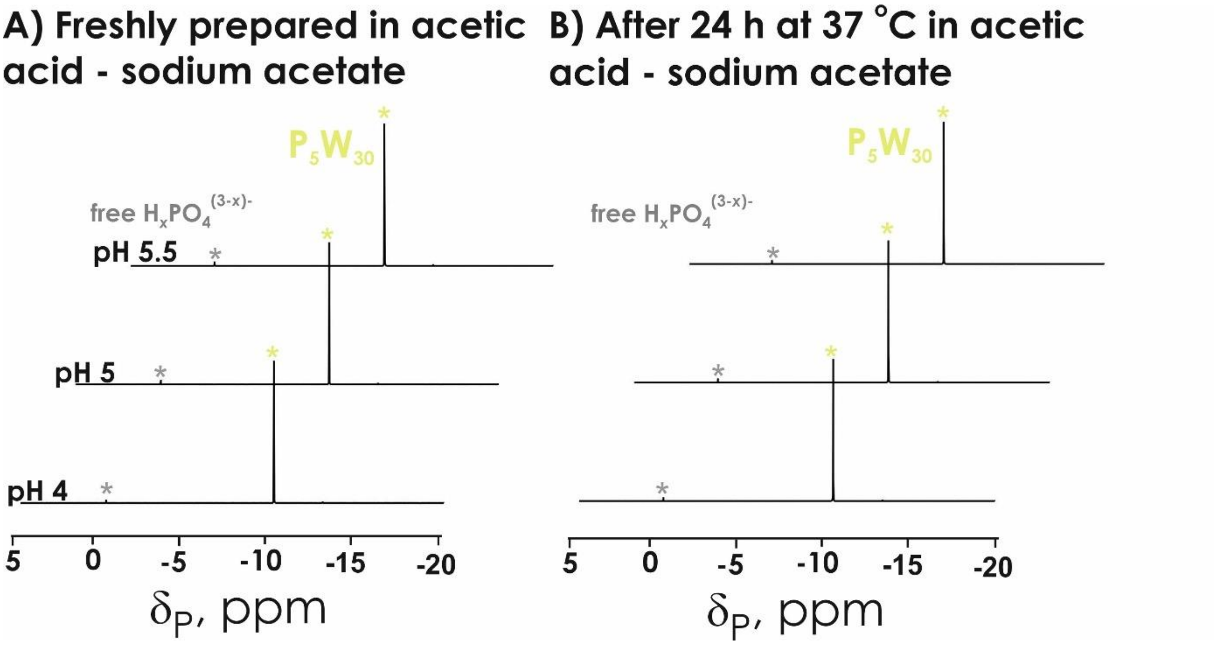

Fig. S70. ^{31}P NMR spectra of P_5W_{30} in acetic acid – sodium acetate buffer.

^{31}P NMR spectra for 10 mM solutions of **$K_{12.5}Na_{1.5}[NaP^{V}_{5}W^{VI}_{30}O_{114}]$** in 0.1 M acetic acid – sodium acetate buffer (pH 4 – 5.5) that were recorded approximately one hour after preparation (**A**) and after incubation for 24 h at 37 °C (**B**). The structures of all POMs are shown in **Figure S67**. The chemical shifts and percentages of parent and formed species are given in **Tables S15** and **S16**. To identify the individual anions, they are shown in different colors, with the same color code being selected for a specific anion throughout all figures and tables in the main manuscript and the supporting information.

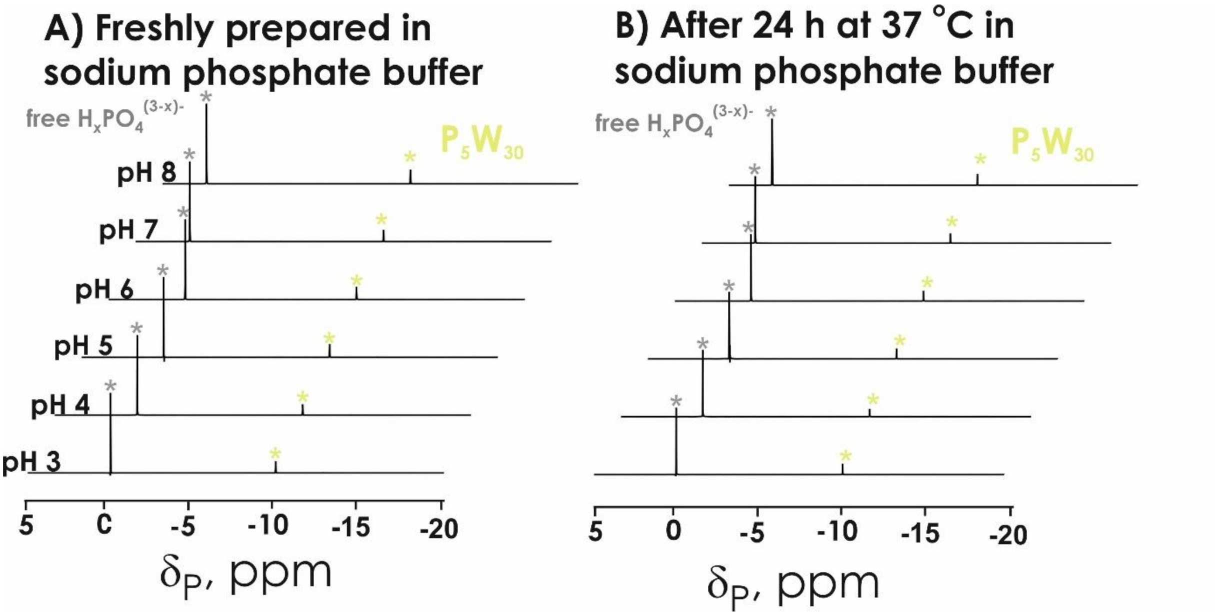

Fig. S71. ^{31}P NMR spectra of P_5W_{30} in sodium phosphate buffer.

^{31}P NMR spectra for 10 mM solutions of **$K_{12.5}Na_{1.5}[NaP^{V}_5W^{VI}_{30}O_{114}]$** in 0.1 M sodium phosphate buffer (pH 3 – 8) that were recorded approximately one hour after preparation (**A**) and after incubation for 24 h at 37 °C (**B**). The structures of all POMs are shown in **Figure S67**. The chemical shifts and percentages of parent and formed species are given in **Tables S15** and **S16**. To identify the individual anions, they are shown in different colors, with the same color code being selected for a specific anion throughout all figures and tables in the main manuscript and the supporting information.

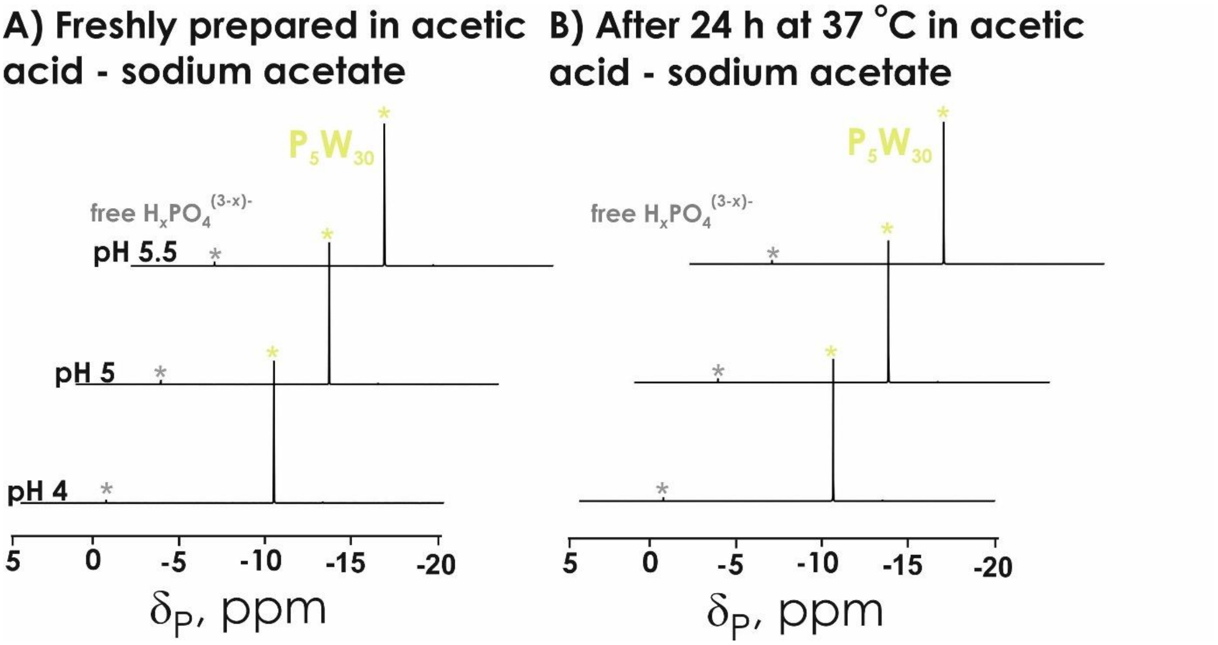

Fig. S72. ^{31}P NMR spectra of P_5W_{30} in citric acid – sodium citrate buffer.

^{31}P NMR spectra for 10 mM solutions of **$K_{12.5}Na_{1.5}[NaP^{V}{}_5W^{VI}{}_{30}O_{114}]$** in 0.1 M citric acid – sodium citrate buffer (pH 3 – 6.5) that were recorded approximately one hour after preparation (**A**) and after incubation for 24 h at 37 °C (**B**). The structures of all POMs are shown in **Figure S67**. The chemical shifts and percentages of parent and formed species are given in **Tables S15** and **S16**. To identify the individual anions, they are shown in different colors, with the same color code being selected for a specific anion throughout all figures and tables in the main manuscript and the supporting information.

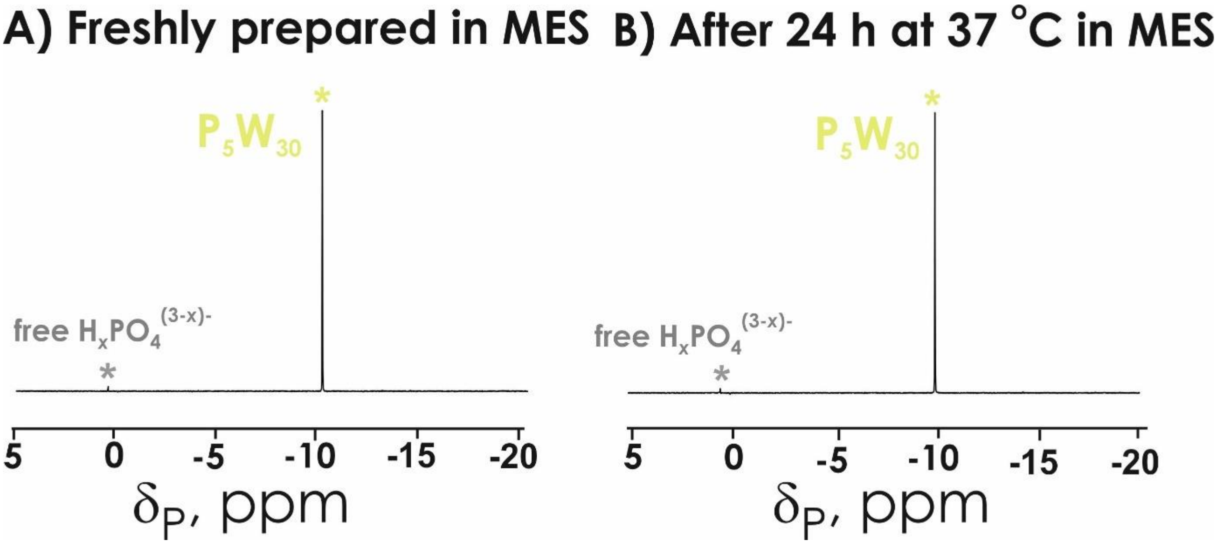

Fig. S73. ^{31}P NMR spectra of P_5W_{30} in MES buffer.

^{31}P NMR spectra for 10 mM solutions of **$K_{12.5}Na_{1.5}[NaP^{V}_{5}W^{VI}_{30}O_{114}]$** in 0.1 M MES buffer pH 5.5 that were recorded approximately one hour after preparation (**A**) and after incubation for 24 h at 37 °C (**B**). The structures of all POMs are shown in **Figure S67**. The chemical shifts and percentages of parent and formed species are given in **Tables S15** and **S16**. To identify the individual anions, they are shown in different colors, with the same color code being selected for a specific anion throughout all figures and tables in the main manuscript and the supporting information.

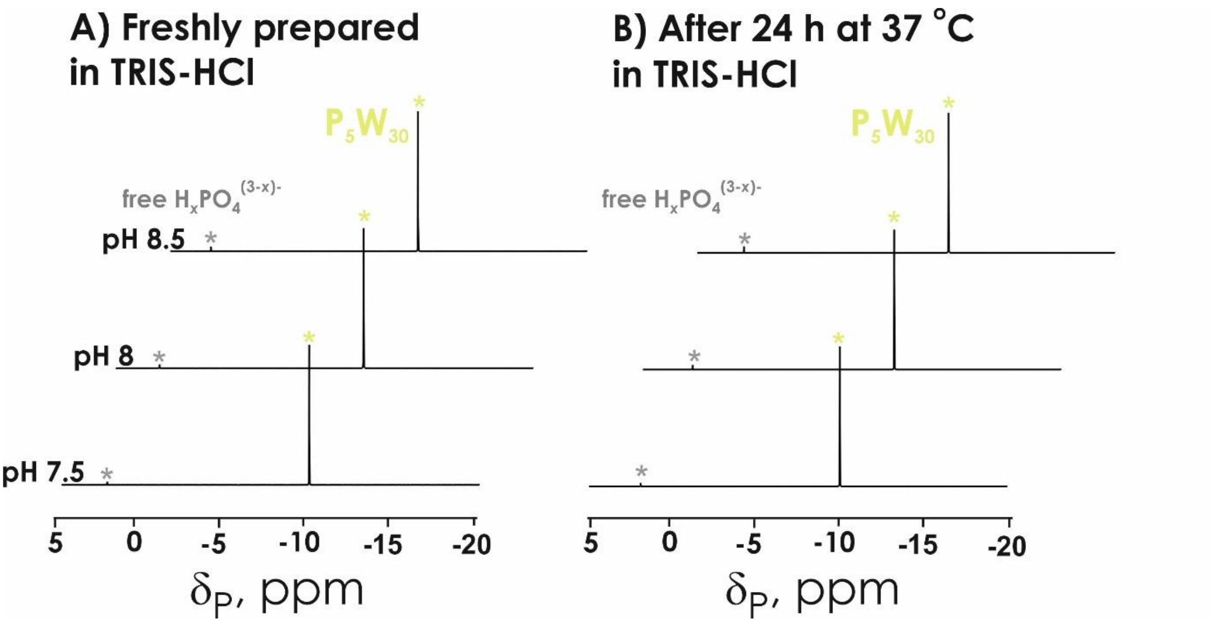

Fig. S74. ^{31}P NMR spectra of P_5W_{30} in tris-HCl buffer.

^{31}P NMR spectra for 10 mM solutions of **$K_{12.5}Na_{1.5}[NaP^{V}_5W^{VI}_{30}O_{114}]$** in 0.1 M tris-HCl buffer (pH 7.5 – 8.5) that were recorded approximately one hour after preparation (**A**) and after incubation for 24 h at 37 °C (**B**). The structures of all POMs are shown in **Figure S67**. The chemical shifts and percentages of parent and formed species are given in **Tables S15** and **S16**. To identify the individual anions, they are shown in different colors, with the same color code being selected for a specific anion throughout all figures and tables in the main manuscript and the supporting information.

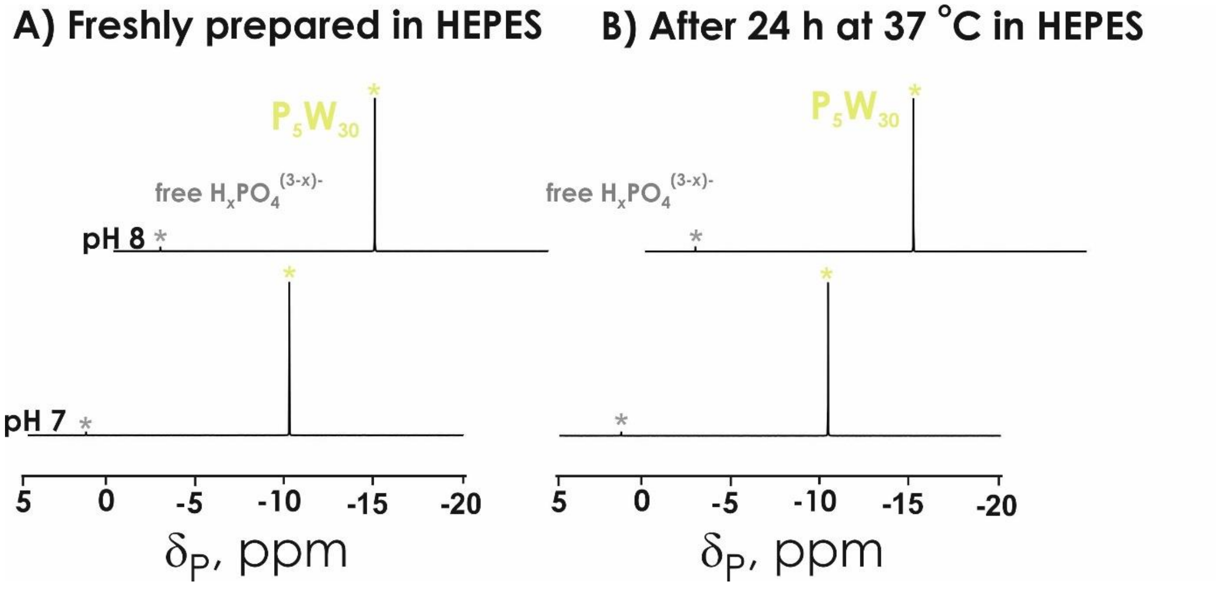

Fig. S75. ^{31}P NMR spectra of P_5W_{30} in HEPES buffer.

^{31}P NMR spectra for 10 mM solutions of **$K_{12.5}Na_{1.5}[NaP^{V}_5W^{VI}_{30}O_{114}]$** in 0.1 M HEPES buffer (pH 7 and 8) that were recorded approximately one hour after preparation (**A**) and after incubation for 24 h at 37 °C (**B**). The structures of all POMs are shown in **Figure S67**. The chemical shifts and percentages of parent and formed species are given in **Tables S15** and **S16**. To identify the individual anions, they are shown in different colors, with the same color code being selected for a specific anion throughout all figures and tables in the main manuscript and the supporting information.

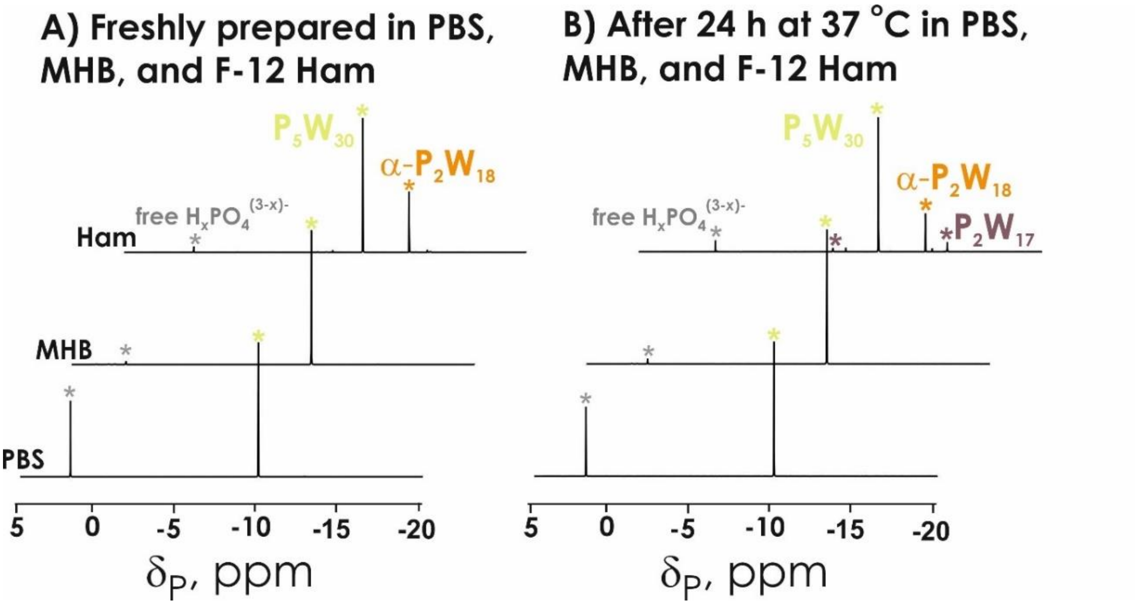

Fig. S76. ^{31}P NMR spectra of P_5W_{30} in solutions with pH 7.4.

^{31}P NMR spectra for 10 mM solutions of **$K_{12.5}Na_{1.5}[NaP^{V}_{5}W^{VI}_{30}O_{114}]$** in PBS, MHB and nutrient mixture F-12 Ham (pH 7.4) that were recorded approximately one hour after preparation (**A**) and after incubation for 24 h at 37 °C (**B**). The structures of all POMs are shown in **Figure S67**. The chemical shifts and percentages of parent and formed species are given in **Tables S15** and **S16**. To identify the individual anions, they are shown in different colors, with the same color code being selected for a specific anion throughout all figures and tables in the main manuscript and the supporting information.

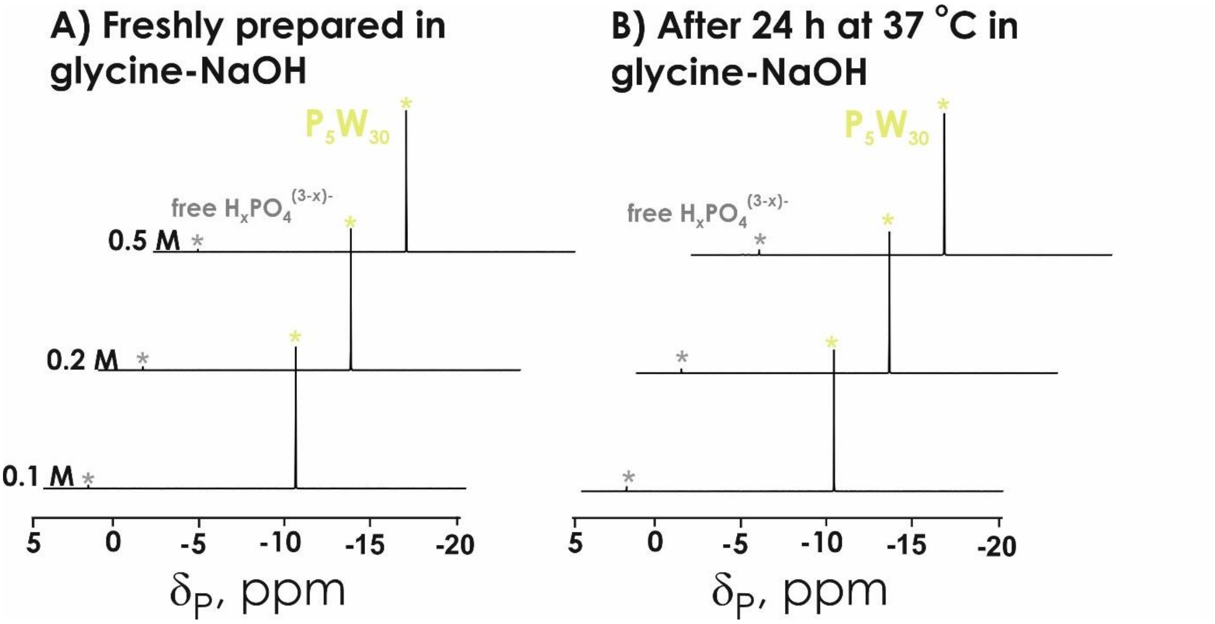

Fig. S77. ^{31}P NMR spectra of P_5W_{30} in glycine-NaOH buffer.

^{31}P NMR spectra for 10 mM solutions of **$K_{12.5}Na_{1.5}[NaP^{V}_{5}W^{VI}_{30}O_{114}]$** in glycine-NaOH (pH 8.6) with concentration 0.1. 0.2 and 0.5 M. that were recorded approximately one hour after preparation (**A**) and after incubation for 24 h at 37 °C (**B**). The structures of all POMs are shown in **Figure S67**. The chemical shifts and percentages of parent and formed species are given in **Tables S15** and **S16**. To identify the individual anions, they are shown in different colors, with the same color code being selected for a specific anion throughout all figures and tables in the main manuscript and the supporting information.

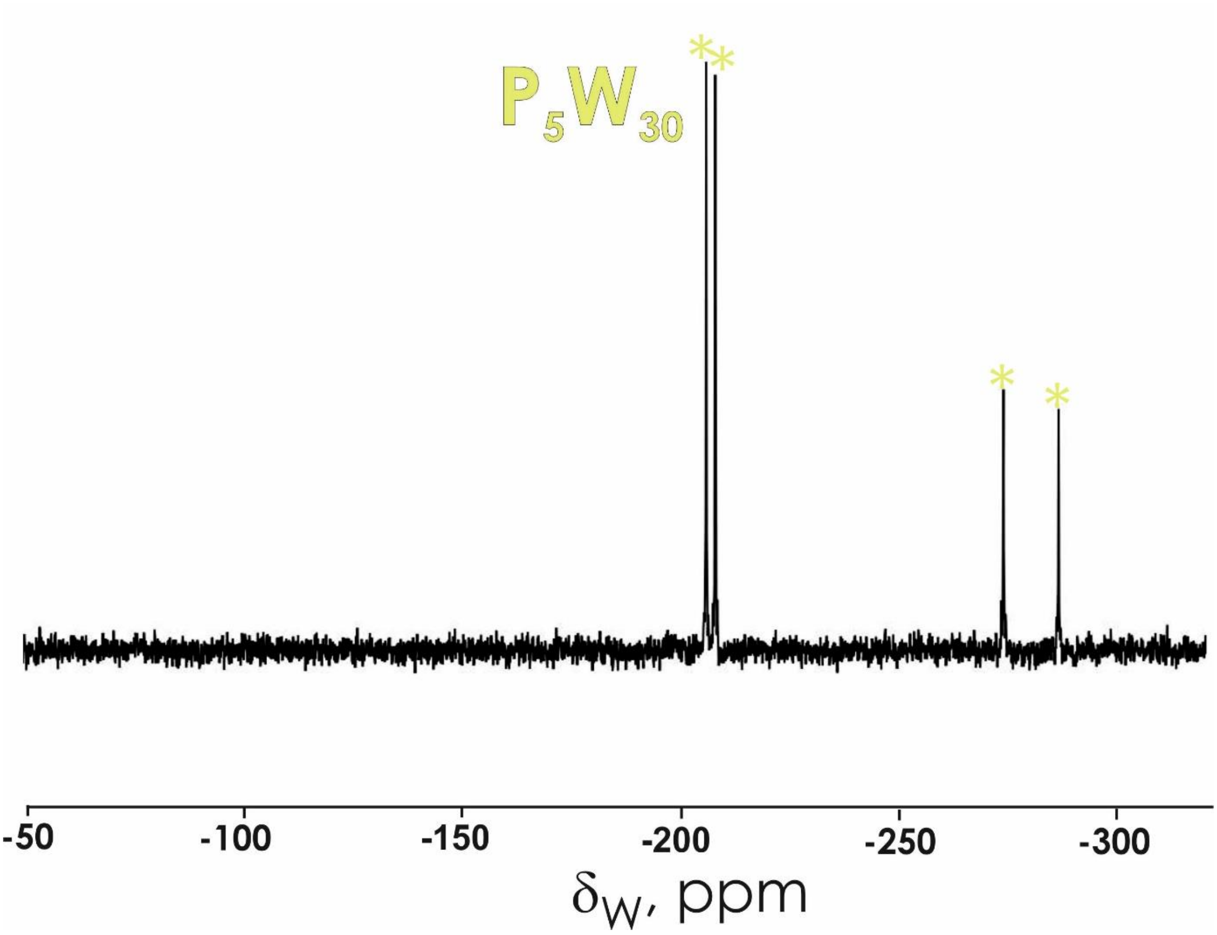

Fig. S78. ^{183}W NMR spectrum of P5W30 in tris-HCl buffer.

^{183}W NMR spectra for 20 mM solutions of **$K_{12.5}Na_{1.5}[NaP^{V}_{5}W^{VI}_{30}O_{114}]$** in 0.2 M tris-HCl pH 7.5 solution showing 4 signals at –205.8 (10W), – 207.9 (10W), –273.6 (5W), and –286.2 (5W) ppm that correspond to four types of W ions in $[NaP^{V}_{5}W^{VI}_{30}O_{114}]^{14-}$. The structures of all POMs are shown in **Figure S67**. To identify the individual anions, they are shown in different colors, with the same color code being selected for a specific anion throughout all figures and tables in the main manuscript and the supporting information.

Table S15. Analysis of NMR spectroscopic data recorded in P_5W_{30} solutions at room temperature.

Chemical shifts in ^{31}P NMR spectra measured in triplicate of **$K_{12.5}Na_{1.5}[NaP^{V}_5W^{VI}_{30}O_{114}]$** (10 mM) dissolved in D_2O and 0.1 M buffers (acetic acid – sodium acetate pH 4 – 5.5; sodium phosphate pH 3 – 8 (while phosphate does not buffer at pH range from 3.5 – 5.5, experiments were conducted at this pH to provide comparisons to previously published studies (*29*)); citric acid – sodium citrate pH 3 – 6.5; MES pH 5.5; PBS pH 7.4; tris-HCl pH 7.5 – 8.5; HEPES pH 7 – 8; MHB pH 7.4; Nutrient mixture F-12 Ham pH 7.4 and glycine-NaOH pH 8.6) (**Figures S69** – **S77**). The glycine-NaOH buffer was used in two additional concentrations 0.2 and 0.5 M. The species content was calculated based on the integration of ^{31}P signals considering only signals associated with POTs. Signals were assigned based on the literature data summarized in **Table S4**.

pH	**$K_{14}[NaP_5W_{30}O_{110}]$** (10 mM) in Solvent / Buffer / Medium	Chemical shifts δ ^{31}P [ppm]	% of P_5W_{30} at RT in **$K_{12.5}Na_{1.5}[NaP^{V}_5W^{VI}_{30}O_{114}]$** (10 mM) solution				Mean % of other POTs
			Sample			Mean of 1 to 3 ± SD[a]	
			#1	#2	#3		
-	D_2O	0; –10.1; –13.0	98	98	99	98 ± 1	2
Strongly acidic environment 3 ≤ pH ≤ 4							
3	0.1 M Sodium phosphate ($H_2PO_4^-/H_3PO_4$) pH 3	0; –10.1; –13.0	99	99	99	99 ± 0	1
	0.1 M Citric acid – sodium citrate (H_3Cit/H_2Cit^-) pH 3	0; –10.1; –13.1	97	99	99	98 ± 1	2
4	0.1 M Sodium phosphate ($H_2PO_4^-/H_3PO_4$) pH 4	0; –10.1; –13.0	98	99	99	99 ± 1	1
	0.1 M Citric acid – sodium citrate ($H_2Cit^-/HCit^{2-}$) pH 4	0; –10.1; –13.1	98	99	99	99 ± 1	1
	0.1 M Acetic acid – sodium acetate ($OAc^-/HOAc$) pH 4	0; –10.1; –13.0	99	98	99	99 ± 1	1
Moderately acidic environment 5 ≤ pH ≤ 6							
5	0.1 M Sodium phosphate ($H_2PO_4^-/H_3PO_4$) pH 5	0; –10.1; –13.0	98	99	99	99 ± 1	1
	0.1 M Citric acid – sodium citrate ($H_2Cit^-/HCit^{2-}$) pH 5	0; –10.1; –13.0	98	99	99	99 ± 1	1
	0.1 M Acetic acid – sodium acetate ($OAc^-/HOAc$) pH 5	0; –10.1; –13.0	97	98	100	98 ± 2	2
5.5	0.1 M Acetic acid – sodium acetate ($OAc^-/HOAc$) pH 5.5	0; –10.1; –13.0	99	98	99	99 ± 1	1
	0.1 M MES[b] pH 5.5	0; –10.1	100	100	100	100 ± 0	0
6	0.1 M Sodium phosphate ($HPO_4^{2-}/H_2PO_4^-$) pH 6	0; –10.1; –13.0	98	100	100	99 ± 1	1

	0.1 M Citric acid – sodium citrate ($HCit^{2-}/Cit^{3-}$) pH 6	0.4; –10.1; –13.1	98	99	99	99 ± 1	1
Neutral environment 6.5 ≤ pH ≤ 7.5							
6.5	0.1 M Citric acid – sodium citrate ($HCit^{2-}/Cit^{3-}$) pH 6.5	0.9; –10.1; –13.0	98	99	100	99 ± 1	1
7	0.1 M Sodium phosphate ($HPO_4^{2-}/H_2PO_4^-$) pH 7	1.7; –10.1	100	100	100	100 ± 0	0
	0.1 M HEPES[c] pH 7	1.7; –7.3; –10.1; –14.4	99	99	100	99 ± 1	1
7.4	PBS[d] pH 7.4	1.8; –10.1	100	99	100	100 ± 1	0
	MHB[e] pH 7.4	3.9; 3.5; 2.7; 2.4; –10.1	100	100	100	100 ± 0	0
	Nutrient mixture F-12 Ham[f]	2.4; –10.1	99	98	99	99 ± 1	1
7.5	0.1 M tris-HCl[g] pH 7.5	0; –10.1	100	99	100	99 ± 1	1
Moderately alkaline environment 8 ≤ pH ≤ 8.6							
8	0.1 M Sodium phosphate ($HPO_4^{2-}/H_2PO_4^-$) pH 8	2.4; –10.1	100	100	100	100 ± 0	0
	0.1 M HEPES pH 8	2.4; –10.1	100	99	100	100 ± 1	0
	0.1 M tris-HCl pH 8	0; –10.1	99	99	100	99 ± 1	1
8.5	0.1 M tris-HCl pH 8.5	0; –10.1	100	99	100	100 ± 1	1
8.6	0.1 M glycine-NaOH pH 8.6	2.4; –7.2; –10.1; –14.4	99	99	100	99 ± 1	0
	0.2 M glycine-NaOH pH 8.6	2.4; –7.2; –10.1; –14.4	100	100	100	100 ± 0	0
	0.5 M glycine-NaOH pH 8.6	2.4; –10.1; –14.4	100	100	100	100 ± 0	

[a]SD – standard deviation; [b]MES – 2-(N-morpholino)ethanesulfonic acid, $C_6H_{13}NO_4S$ (Figure S1); [c]HEPES – 4-(2-hydroxyethyl)-1-piperazineethanesulfonic acid, $C_8H_{18}N_2O_4S$ (Figure S1); [d]PBS – phosphate buffer saline; [e]MHB – Mueller-Hinton broth, for more detailed information about composition, see https://labmal.com/2019/11/20/mueller-hinton-agar-and-mueller-hinton-broth/; [f]Nutrient mixture F-12 Ham contains sodium pyruvate (0.11 g/L), phenol red, L-glutamine, and does not contain $NaHCO_3$ and HEPES, for more details please see https://www.sigmaaldrich.com/AT/en/technical-documents/technical-article/cell-culture-and-cell-culture-analysis/mammalian-cell-culture/f-12-ham; [g]tris – tris(hydroxymethyl)aminomethane, $C_4H_{11}NO_3$ (Figure S1).

Table S16. Analysis of NMR spectroscopic data recorded in P_5W_{30} solutions investigated after 24 h incubation at 37 °C.

Chemical shifts in ^{31}P NMR spectra measured in triplicate of **$K_{12.5}Na_{1.5}[NaP^{V}_5W^{VI}_{30}O_{114}]$** (10 mM) dissolved in D_2O and 0.1 M buffers (acetic acid – sodium acetate pH 4 – 5.5; sodium phosphate pH 3 – 8 (while phosphate does not buffer at pH range from 3.5 – 5.5, experiments were conducted at this pH to provide comparisons to previously published studies (*29*)); citric acid – sodium citrate pH 3 – 6.5; MES pH 5.5; PBS pH 7.4; tris-HCl pH 7.5 – 8.5; HEPES pH 7 – 8; MHB pH 7.4; Nutrient mixture F-12 Ham pH 7.4 and glycine-NaOH pH 8.6) and investigated after 24 h incubation at 37 °C (**Figures S69** – **S77**). The glycine-NaOH buffer was used in two additional concentrations 0.2 and 0.5 M. The species content was calculated based on the integration of ^{31}P signals considering only signals associated with POTs. Signals were assigned based on the literature data summarized in **Table S4**.

pH	**$K_{14}[NaP_5W_{30}O_{110}]$** (10 mM) in Solvent / Buffer / Medium	Chemical shifts δ ^{31}P [ppm]	% of P_5W_{30} at RT in **$K_{14}[NaP_5W_{30}O_{110}]$** (10 mM) solution after 24 h incubation at 37 °C				Mean % of other POTs
			Sample			Mean of 1 to 3 ± SD[a]	
			#1	#2	#3		
-	D_2O	0; –10.1; –13.0	98	98	99	98 ± 1	2
Strongly acidic environment 3 ≤ pH ≤ 4							
3	0.1 M Sodium phosphate ($H_2PO_4^-$ /H_3PO_4) pH 3	0; –10.1; –13.0	99	99	99	99 ± 0	1
	0.1 M Citric acid – sodium citrate (H_3Cit/H_2Cit^-) pH 3	0; –10.1; –13.1	99	99	99	99 ± 0	1
4	0.1 M Sodium phosphate ($H_2PO_4^-$ /H_3PO_4) pH 4	0; –10.1; –13.0	98	99	99	99 ± 1	1
	0.1 M Citric acid – sodium citrate ($H_2Cit^-/HCit^{2-}$) pH 4	0; –10.1; –13.1	99	99	99	99 ± 0	1
	0.1 M Acetic acid – sodium acetate (OAc^-/HOAc) pH 4	0; –10.1; –13.0	99	98	99	99 ± 1	1
Moderately acidic environment 5 ≤ pH ≤ 6							
5	0.1 M Sodium phosphate ($H_2PO_4^-$ /H_3PO_4) pH 5	0; –10.1; –13.0	98	100	99	99 ± 1	1
	0.1 M Citric acid – sodium citrate ($H_2Cit^-/HCit^{2-}$) pH 5	0; –10.1; –13.0	99	99	100	99 ± 1	1
	0.1 M Acetic acid – sodium acetate (OAc^-/HOAc) pH 5	0; –10.1; –13.0	99	98	100	99 ± 1	1

5.5	0.1 M Acetic acid – sodium acetate (OAc^{-}/HOAc) pH 5.5	0; –10.1	100	99	100	99 ± 1	0
	0.1 M MES[b] pH 5.5	0; –10.1	100	99	100	99 ± 1	0
6	0.1 M Sodium phosphate (HPO_4^{2-}/$H_2PO_4^{-}$) pH 6	0.4; –2.4; –7.2; –10.1; –14.4	99	99	99	99 ± 0	1
	0.1 M Citric acid – sodium citrate ($HCit^{2-}$/Cit^{3-}) pH 6	0.4; –10.1; –14.4	100	99	100	100 ± 1	0
Neutral environment 6.5 ≤ pH ≤ 7.5							
6.5	0.1 M Citric acid – sodium citrate ($HCit^{2-}$/Cit^{3-}) pH 6.5	0.9; –10.1	100	100	100	100 ± 0	0
7	0.1 M Sodium phosphate (HPO_4^{2-}/$H_2PO_4^{-}$) pH 7	1.7; –10.1	99	100	100	100 ± 1	0
	0.1 M HEPES[c] pH 7	1.7; –7.3; –10.1; –14.4	99	99	100	99 ± 1	1
7.4	PBS[d] pH 7.4	1.6; –10.1	100	100	100	100 ± 0	0
	MHB[e] pH 7.4	3.9; 3.5; 2.7; 2.4; –1.6; –7.8; –10.1; –14.4	99	99	100	99 ± 1	1
	Nutrient mixture F-12 Ham[f]	1.6; –10.1	99	99	99	99 ± 1	1
7.5	0.1 M tris-HCl[g] pH 7.5	0; –10.1	100	100	100	100 ± 0	0
Moderately alkaline environment 8 ≤ pH ≤ 8.6							
8	0.1 M Sodium phosphate (HPO_4^{2-}/$H_2PO_4^{-}$) pH 8	2.3; –10.1	99	100	100	100 ± 1	0
	0.1 M HEPES pH 8	2.4; –10.1	99	100	100	99 ± 1	0
	0.1 M tris-HCl pH 8	0; –10.1	100	99	100	99 ± 1	0
8.5	0.1 M tris-HCl pH 8.5	0; –10.1	100	100	100	100 ± 0	0
8.6	0.1 M glycine-NaOH pH 8.6	2.3; –10.1	100	100	100	100 ± 0	0
	0.2 M glycine-NaOH pH 8.6	2.3; –10.1	100	100	100	100 ± 0	0
	0.5 M glycine-NaOH pH 8.6	2.3; –10.1	100	100	100	100 ± 0	0

[a]SD – standard deviation; [b]MES – 2-(N-morpholino)ethanesulfonic acid, $C_6H_{13}NO_4S$ (Figure S1); [c]HEPES – 4-(2-hydroxyethyl)-1-piperazineethanesulfonic acid, $C_8H_{18}N_2O_4S$ (Figure S1); [d]PBS – phosphate buffer saline; [e]MHB – Mueller-Hinton broth, for more detailed information about composition, see https://labmal.com/2019/11/20/mueller-hinton-agar-and-mueller-hinton-broth/; [f]Nutrient mixture F-12 Ham contains sodium pyruvate (0.11 g/L), phenol red, L-glutamine, and does not contain $NaHCO_3$ and HEPES, for more details please see https://www.sigmaaldrich.com/AT/en/technical-documents/technical-article/cell-culture-and-cell-culture-analysis/mammalian-cell-culture/f-12-ham; [g]tris – tris(hydroxymethyl)aminomethane, $C_4H_{11}NO_3$ (Figure S1).

8.3. Speciation in Preyssler POT solutions

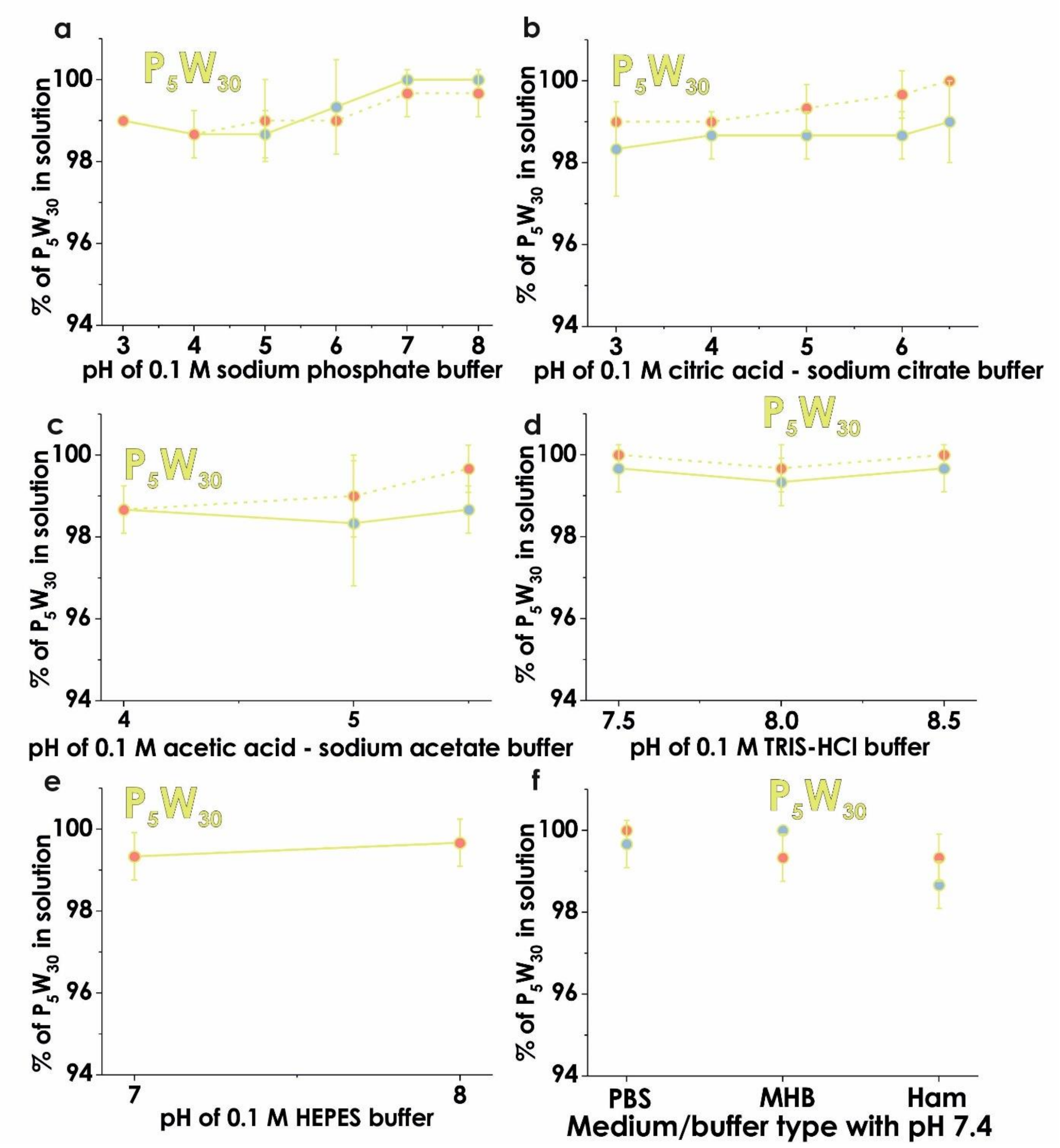

Fig. S79. Speciation in P_5W_{30} solutions.

Concentration curves of **P_5W_{30}** (light-green) before (solid line, blue dot in the middle) and after incubation (dash line, red dot in the middle) in **$K_{12.5}Na_{1.5}[NaP^{V}_5W^{VI}_{30}O_{114}]$** (10 mM) solutions. Color code: {WO_6}, orange; P, grey; O, red, Na omitted for clarity. The exact percentage of all POM species present is given in Supplementary **Tables S15** and **S16**.

9. The PMo_{12} Keggin POMo

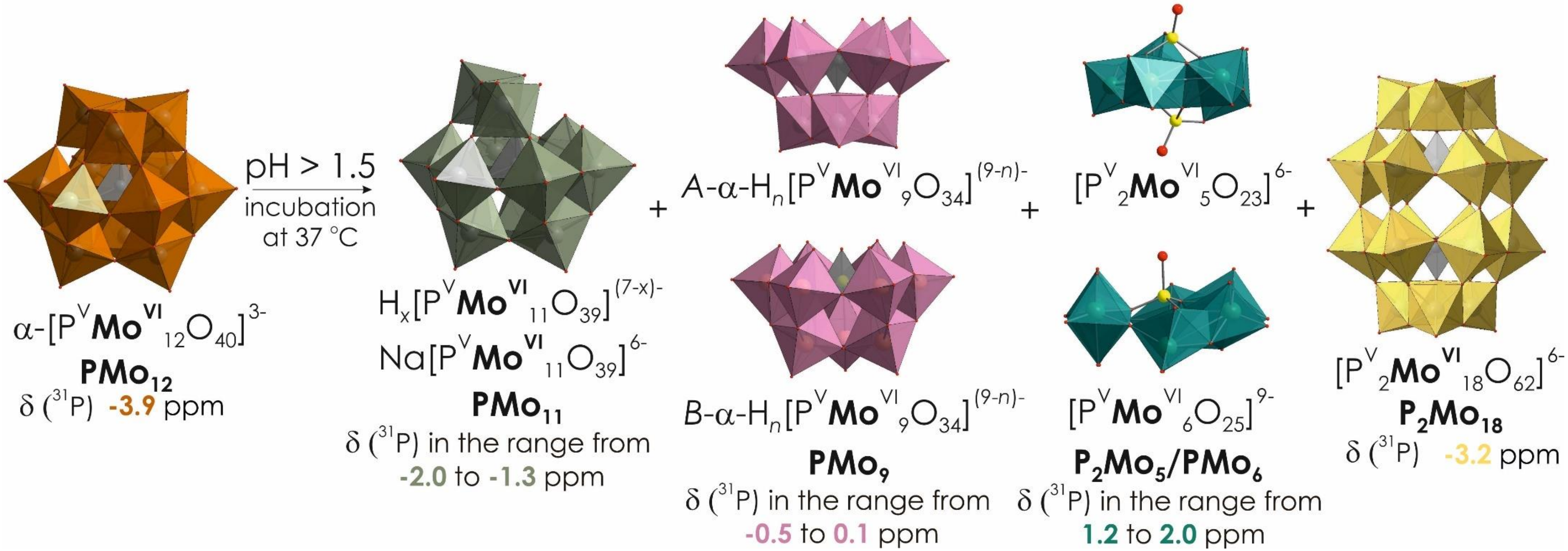

Fig. S80. Hydrolysis of PMo_{12}.

Structure of the Keggin POMo [α-$P^{V}Mo^{VI}{}_{12}O_{40}]^{3-}$ (**PMo_{12}**) and its hydrolysis scheme based on the current investigation. The polyanions that can be formed during hydrolysis in the pH range from 1.5 to 8.6 are shown with corresponding ^{31}P NMR chemical shifts. Color code: {MoO_6}, light-brown, gray-green, pink, turquoise, or yellow; {PO_4}, grey or yellow; O, red. To identify the individual anions, they are shown in different colors, with the same color code being selected for a specific anion throughout all figures and tables in the main manuscript and the supporting information.

9.1. pH of PMo_{12} Keggin POMo solutions

Table S17. pH in PMo_{12} solutions.

pH values measured in triplicate of **$H_3[\alpha\text{-}P^{V}Mo^{VI}_{12}O_{40}]$** (10 mM) dissolved in D_2O and different buffers (acetic acid – sodium acetate pH 4 – 5.5; sodium phosphate pH 3 – 8 (while phosphate does not buffer at pH range from 3.5 – 5.5, experiments were conducted at this pH to provide comparisons to previously published studies (*29*)); citric acid – sodium citrate pH 3 – 6.5; MES pH 5.5; PBS pH 7.4; tris-HCl pH 7.5 – 8.5; HEPES pH 7 – 8; MHB pH 7.4; Nutrient mixture F-12 Ham pH 7.4 and glycine-NaOH pH 8.6) with concentration 0.1 M. The glycine-NaOH buffer was used in two additional concentrations 0.2 and 0.5 M.

pH	**$Na_3[PMo_{12}O_{40}]$** (10 mM) in Solvent / Buffer / Medium	pH after dissolving **$H_3[\alpha\text{-}P^{V}Mo^{VI}_{12}O_{40}]$** (10 mM) at RT				pH after 24 h incubation of **$H_3[\alpha\text{-}P^{V}Mo^{VI}_{12}O_{40}]$** (10 mM) solution at 37 °C			
		Sample			Mean of 1 to 3 ± SD[a]	Sample			Mean of 1 to 3 ± SD[a]
		#1	#2	#3		#1	#2	#3	
-	D_2O	1.36	1.25	1.29	1.30 ± 0.06	1.16	1.18	1.25	1.20 ± 0.05
Strongly acidic environment 3 ≤ pH ≤ 4									
3	0.1 M Sodium phosphate ($H_2PO_4^-$/H_3PO_4) pH 3	1.98	2.00	1.92	1.97 ± 0.04	1.90	1.94	1.82	1.89 ± 0.06
	0.1 M Citric acid – sodium citrate (H_3Cit/H_2Cit^-) pH 3	1.41	1.37	1.37	1.38 ± 0.02	1.38	1.28	1.30	1.32 ± 0.05
4	0.1 M Sodium phosphate ($H_2PO_4^-$/H_3PO_4) pH 4	2.09	2.16	2.04	2.10 ± 0.06	2.03	2.11	1.94	2.03 ± 0.09
	0.1 M Citric acid – sodium citrate (H_2Cit^-/$HCit^{2-}$) pH 4	2.13	2.23	2.33	2.23 ± 0.10	2.08	2.15	2.27	2.17 ± 0.10
	0.1 M Acetic acid – sodium acetate (OAc^-/HOAc) pH 4	1.87	1.83	1.82	1.84 ± 0.03	1.72	1.76	1.79	1.76 ± 0.04
Moderately acidic environment 5 ≤ pH ≤ 6									
5	0.1 M Sodium phosphate ($H_2PO_4^-$/H_3PO_4) pH 5	2.15	2.06	2.06	2.09 ± 0.05	2.10	2.05	1.98	2.04 ± 0.06
	0.1 M Citric acid – sodium citrate (H_2Cit^-/$HCit^{2-}$) pH 5	4.02	3.52	3.62	3.72 ± 0.26	3.99	3.46	3.59	3.68 ± 0.28
	0.1 M Acetic acid – sodium acetate (OAc^-/HOAc) pH 5	3.93	4.00	3.95	3.96 ± 0.04	3.80	3.94	3.93	3.89 ± 0.08

5.5	0.1 M Acetic acid – sodium acetate ($OAc^-/HOAc$) pH 5.5	4.42	4.43	4.27	4.38 ± 0.09	4.10	4.40	4.25	4.25 ± 0.15
	0.1 M MES[b] pH 5.5	1.58	1.50	1.51	1.53 ± 0.04	1.47	1.45	1.49	1.47 ± 0.02
6	0.1 M Sodium phosphate ($HPO_4^{2-}/H_2PO_4^-$) pH 6	2.43	2.39	2.30	2.37 ± 0.07	2.38	2.35	2.40	2.38 ± 0.03
	0.1 M Citric acid – sodium citrate ($HCit^{2-}/Cit^{3-}$) pH 6	5.26	5.33	5.32	5.30 ± 0.04	5.25	5.31	5.31	5.29 ± 0.03
Neutral environment 6.5 ≤ pH ≤ 7.5									
6.5	0.1 M Citric acid – sodium citrate ($HCit^{2-}/Cit^{3-}$) pH 6.5	5.71	5.70	5.68	5.70 ± 0.02	5.65	5.67	5.66	5.66 ± 0.01
7	0.1 M Sodium phosphate ($HPO_4^{2-}/H_2PO_4^-$) pH 7	6.03	5.87	5.80	5.90 ± 0.12	5.72	5.80	5.71	5.74 ± 0.05
	0.1 M HEPES[c] pH 7	4.24	4.12	4.02	4.13 ± 0.11	4.15	4.07	3.82	4.01 ± 0.17
7.4	PBS[d] pH 7.4	1.64	1.64	1.61	1.63 ± 0.11	1.54	1.61	1.59	1.58 ± 0.04
	MHB[e] pH 7.4	3.60	3.68	3.54	3.61 ± 0.07	3.48	3.46	3.40	3.45 ± 0.04
	Nutrient mixture F-12 Ham[f]	1.65	1.68	1.69	1.67 ± 0.02	1.59	1.63	1.60	1.61 ± 0.02
7.5	0.1 M tris-HCl[g] pH 7.5	1.75	1.76	1.70	1.74 ± 0.03	1.65	1.73	1.68	1.69 ± 0.04
Moderately alkaline environment 8 ≤ pH ≤ 8.6									
8	0.1 M Sodium phosphate ($HPO_4^{2-}/H_2PO_4^-$) pH 8	5.88	5.89	5.94	5.90 ± 0.03	5.86	5.81	5.88	5.85 ± 0.04
	0.1 M HEPES pH 8	5.34	5.25	5.36	5.32 ± 0.06	5.29	5.26	5.22	5.26 ± 0.04
	0.1 M tris-HCl pH 8	2.13	2.03	1.99	2.05 ± 0.07	2.09	2.03	2.00	2.04 ± 0.05
8.5	0.1 M tris-HCl pH 8.5	3.95	4.62	4.32	4.30 ± 0.34	3.87	4.57	4.09	4.18 ± 0.36
8.6	0.1 M glycine-NaOH pH 8.6	2.77	2.80	2.71	2.76 ± 0.05	2.90	2.99	2.91	2.93 ± 0.05
	0.2 M glycine-NaOH pH 8.6	3.32	3.31	3.25	3.29 ± 0.04	3.47	3.60	3.57	3.55 ± 0.07
	0.5 M glycine-NaOH pH 8.6	4.08	3.97	3.97	4.01 ± 0.06	4.59	4.72	4.68	4.66 ± 0.06

[a]SD – standard deviation; [b]MES – 2-(N-morpholino)ethanesulfonic acid, $C_6H_{13}NO_4S$ (Figure S1); [c]HEPES – 4-(2-hydroxyethyl)-1-piperazineethanesulfonic acid, $C_8H_{18}N_2O_4S$ (Figure S1); [d]PBS – phosphate buffer saline; [e]MHB – Mueller-Hinton broth, for more detailed information about composition, see https://labmal.com/2019/11/20/mueller-hinton-agar-and-mueller-hinton-broth/; [f]Nutrient mixture F-12 Ham contains sodium pyruvate (0.11 g/L), phenol red, L-glutamine, and does not contain $NaHCO_3$ and HEPES, for more details please see https://www.sigmaaldrich.com/AT/en/technical-documents/technical-article/cell-culture-and-cell-culture-analysis/mammalian-cell-culture/f-12-ham; [g]tris – tris(hydroxymethyl)aminomethane, $C_4H_{11}NO_3$ (Figure S1).

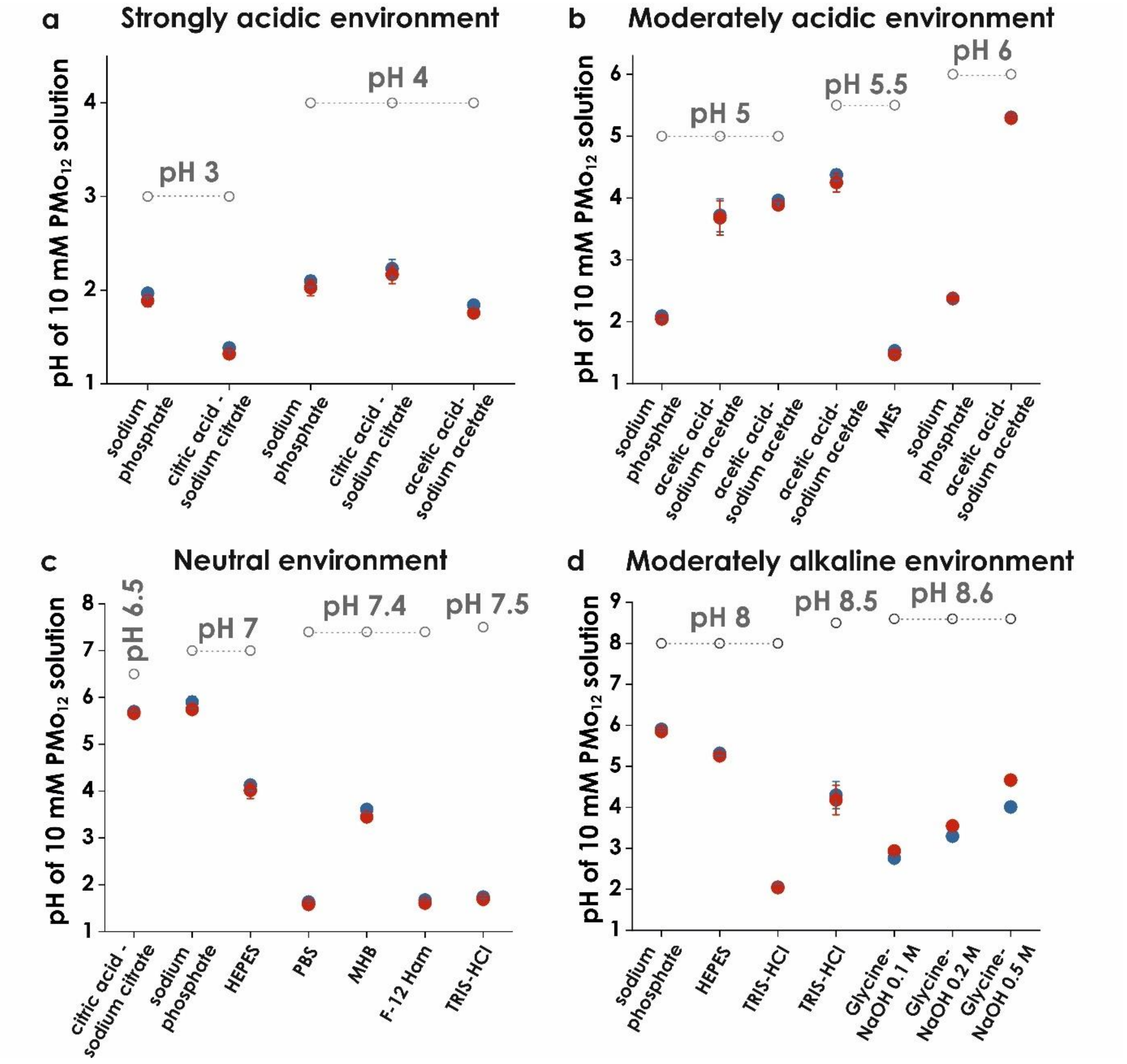

Fig. S81. pH in PMo12 solutions.

The pH dependence curves for 10 mM solutions of **$H_3[\alpha\text{-}P^VMo^{VI}_{12}O_{40}]$** in different buffers in a) strongly acidic, b) moderately acidic, c) neutra, and d) moderately alkaline environment. The pH of the starting buffers is shown with a gray dashed line; the plots of the measured pH values immediately after preparation of the solutions are shown in blue and after 24 h incubation at 37 °C in red. The error bar shows standard deviations (**Table S17**).

9.2. ^{31}P and ^{13}C NMR spectroscopic studies of PMo_{12} Keggin POMo solutions

All peaks with the highest intensity were unambiguously assigned based on the literature data from **Table S4**. In some spectra, chemical shifts for peaks of lower intensity have not yet been described in the literature and therefore can not be assigned in this work.

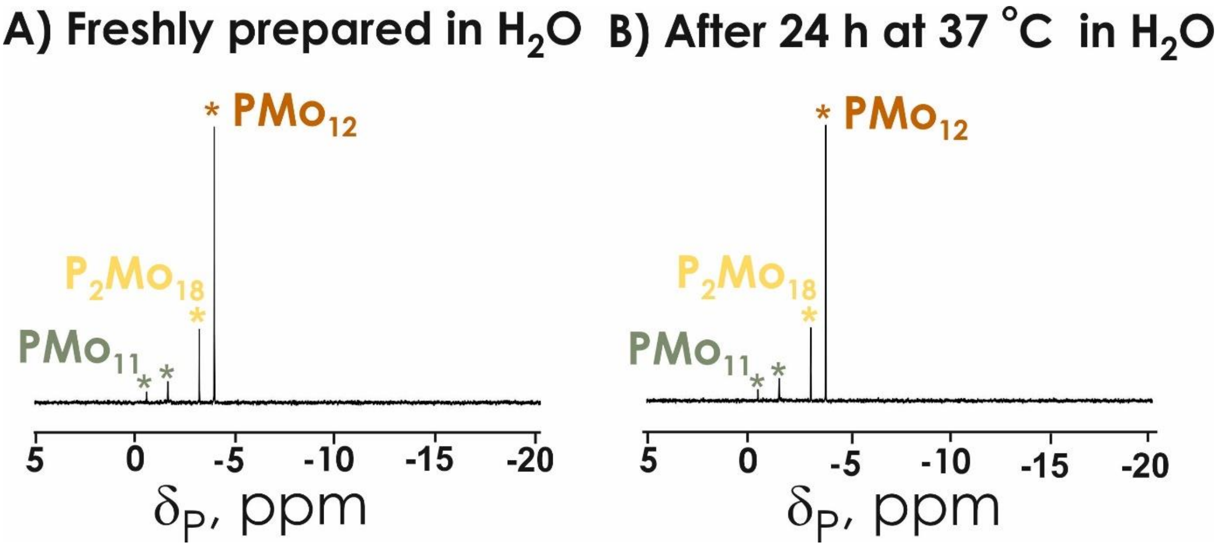

Fig. S82. ^{31}P NMR spectra of PMo_{12} in H_2O.

^{31}P NMR spectra for 10 mM solutions of **$H_3[\alpha\text{-}P^VMo^{VI}_{12}O_{40}]$** in D_2O that were recorded approximately one hour after preparation (**A**) and after incubation for 24 h at 37 °C (**B**). The chemical shifts and percentages of parent and formed species are given in **Tables S18** and **S19**. The structures of all POMs are shown in **Figure S80**. To identify the individual anions, they are shown in different colors, with the same color code being selected for a specific anion throughout all figures and tables in the main manuscript and the supporting information.

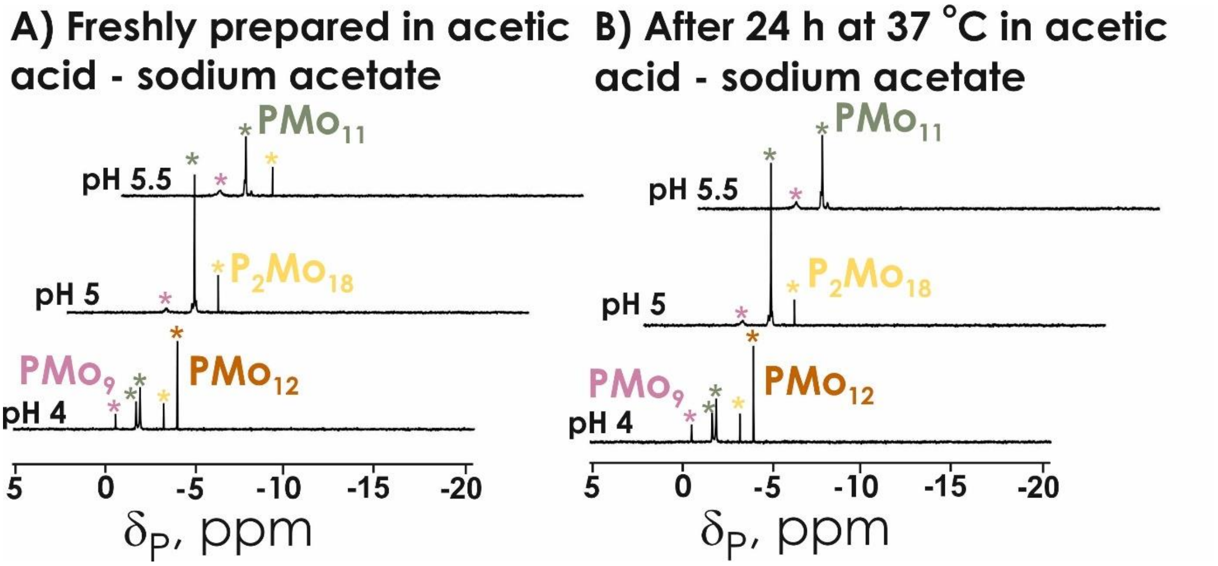

Fig. S83. ^{31}P NMR spectra of PMo$_{12}$ in NaOAc/HOAc buffer.

^{31}P NMR spectra for 10 mM solutions of **$H_3[\alpha$-$P^VMo^{VI}_{12}O_{40}]$** in 0.1 M NaOAc/HOAc buffer (pH 4 – 5.5) that were recorded approximately one hour after preparation (**A**) and after incubation for 24 h at 37 °C (**B**). The chemical shifts and percentages of parent and formed species are given in **Tables S18** and **S19**. The structures of all POMs are shown in **Figure S80**. To identify the individual anions, they are shown in different colors, with the same color code being selected for a specific anion throughout all figures and tables in the main manuscript and the supporting information.

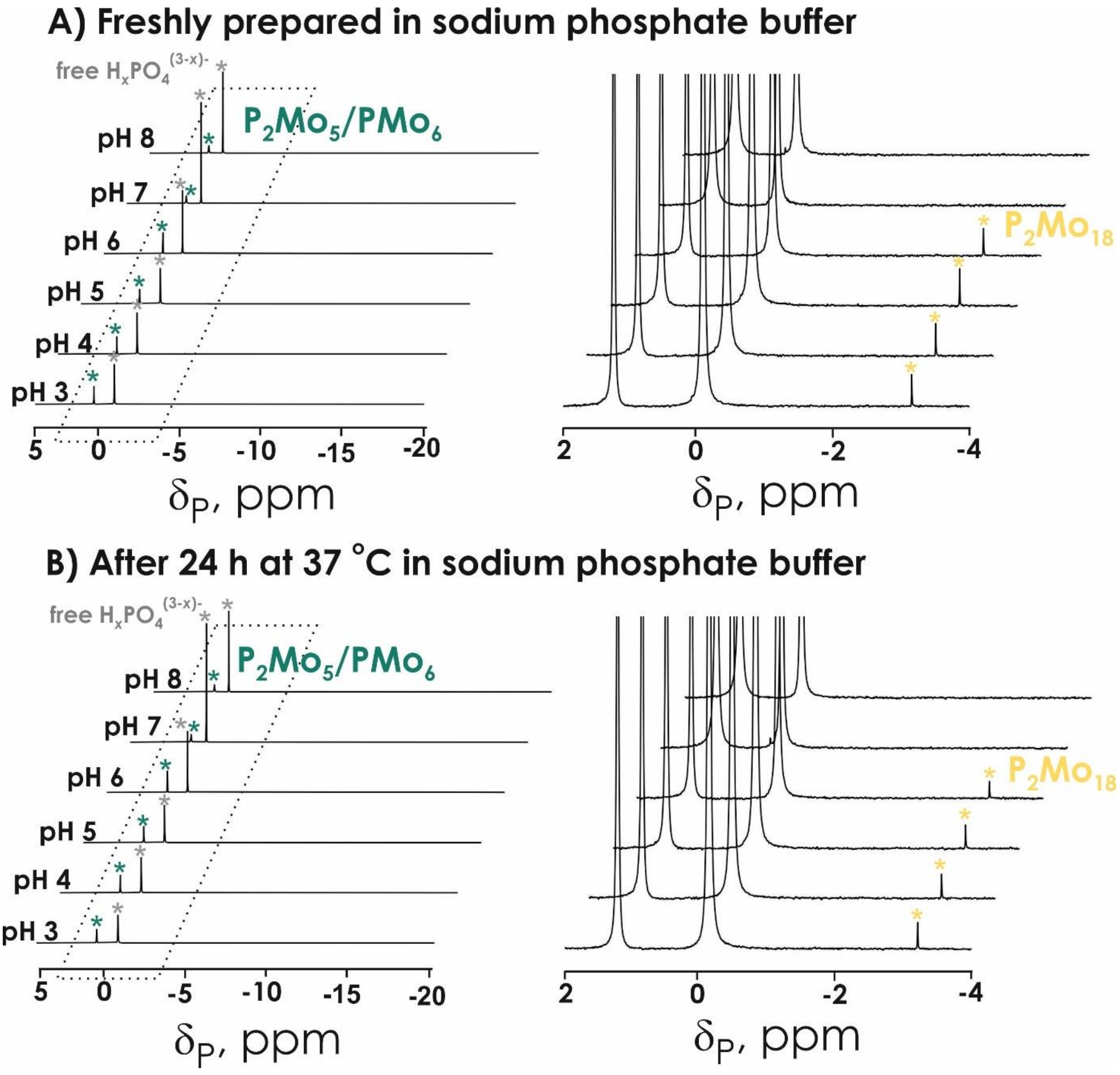

Fig. S84. ^{31}P NMR spectra of PMo_{12} in sodium phosphate buffer.

^{31}P NMR spectra for 10 mM solutions of **$H_3[\alpha\text{-}P^{V}Mo^{VI}_{12}O_{40}]$** in 0.1 M sodium phosphate buffer (pH 3 – 8) that were recorded approximately one hour after preparation (**A**) and after incubation for 24 h at 37 °C (**B**). The chemical shifts and percentages of parent and formed species are given in **Tables S18** and **S19**. The structures of all POMs are shown in **Figure S80**. To identify the individual anions, they are shown in different colors, with the same color code being selected for a specific anion throughout all figures and tables in the main manuscript and the supporting information.

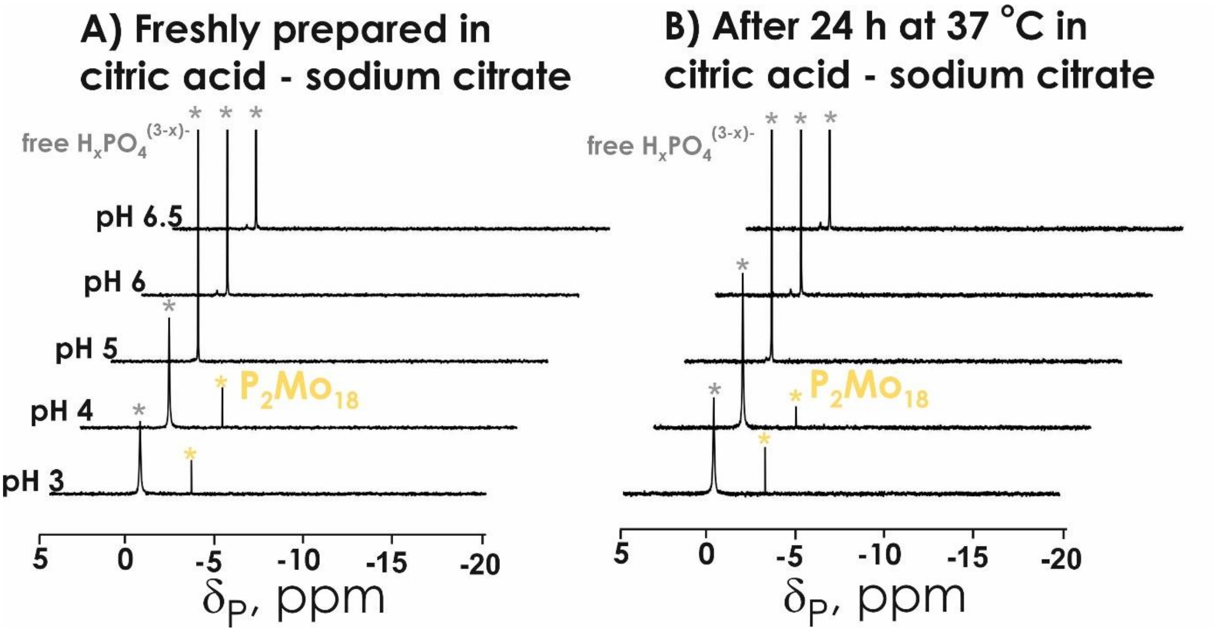

Fig. S85. ^{31}P NMR spectra of PMo12 in citric acid – sodium citrate buffer.

^{31}P NMR spectra for 10 mM solutions of **$H_3[\alpha\text{-}P^{V}Mo^{VI}_{12}O_{40}]$** in 0.1 M citric acid – sodium citrate buffer (pH 3 – 6.5) that were recorded approximately one hour after preparation (**A**) and after incubation for 24 h at 37 °C (**B**). The chemical shifts and percentages of parent and formed species are given in **Tables S18** and **S19**. The structures of all POMs are shown in **Figure S80**. To identify the individual anions, they are shown in different colors, with the same color code being selected for a specific anion throughout all figures and tables in the main manuscript and the supporting information.

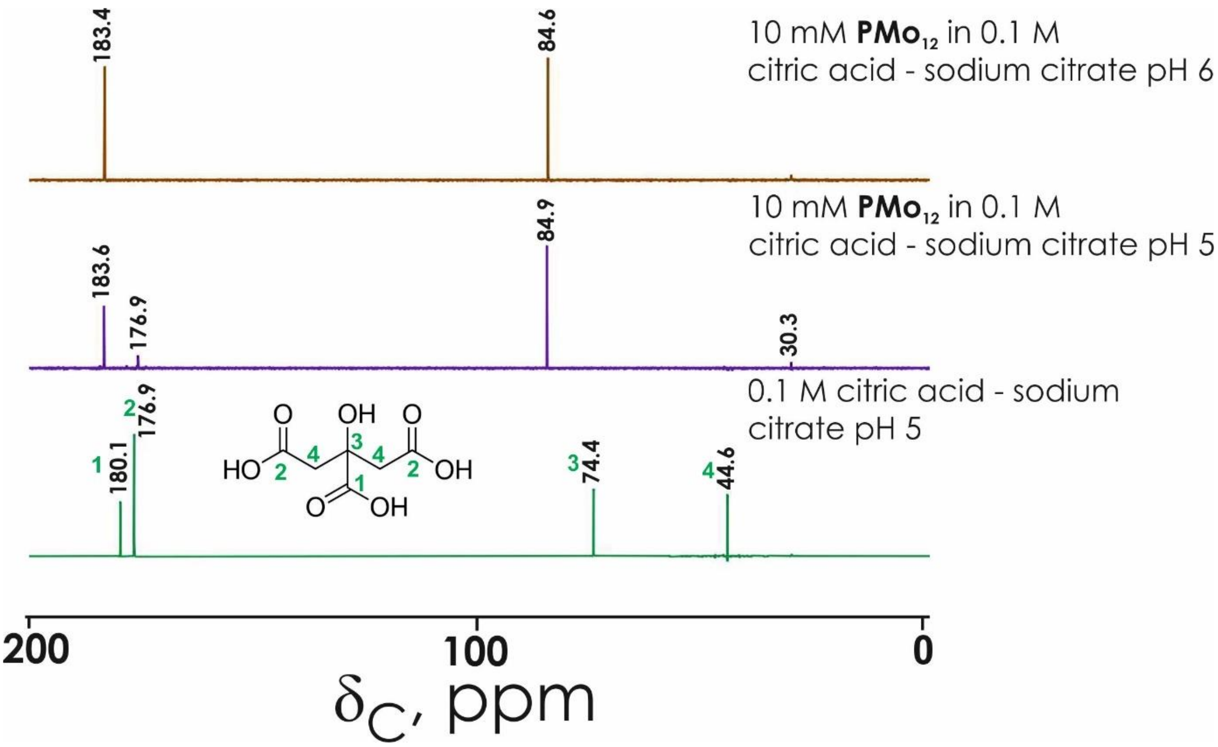

Fig. S86. ^{13}C NMR spectra of PMo12 in citric acid – sodium citrate buffer.

^{13}C NMR spectra for citric acid – sodium citrate buffer at pH 5 and for 10 mM solutions of **$H_3[\alpha\text{-}P^{V}Mo^{VI}_{12}O_{40}]$** in 0.1 M citric acid – sodium citrate buffer at pH 5 and 6 that were recorded approximately one hour after preparation. The difference in the spectra of blank buffer and POM solutions indicates that Cit^{3-} is likely forming Mo-Cit complexes, consistent with the ^{31}P NMR spectra for POM solutions, which do not show any POM-associated signals at pH > 4.

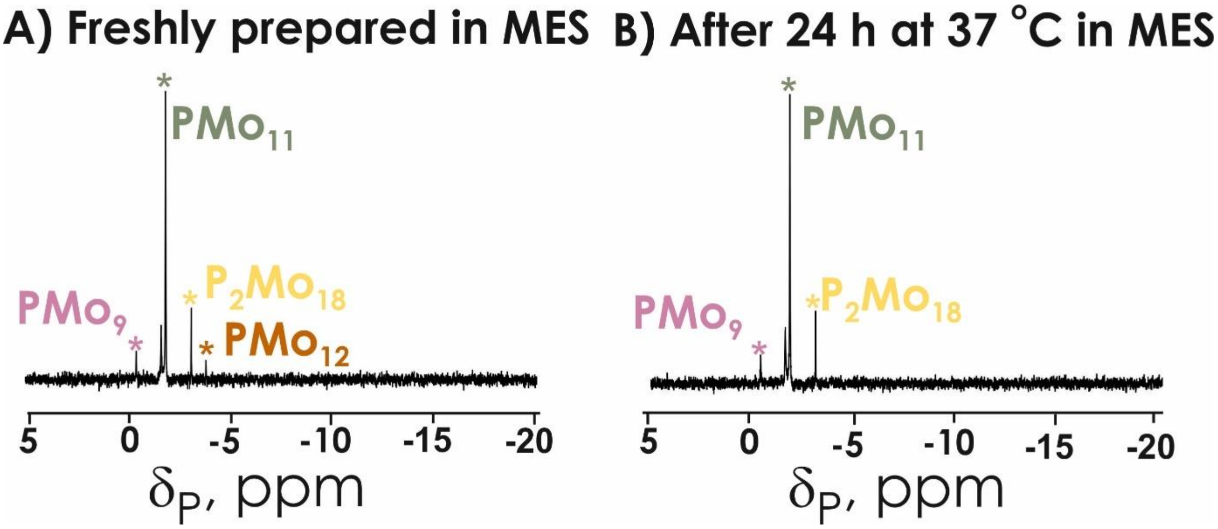

Fig. S87. ^{31}P NMR spectra of PMo_{12} in MES buffer.

^{31}P NMR spectra for 10 mM solutions of **$H_3[\alpha\text{-}P^VMo^{VI}_{12}O_{40}]$** in 0.1 M MES buffer pH 5.5 that were recorded approximately one hour after preparation (**A**) and after incubation for 24 h at 37 °C (**B**). The chemical shifts and percentages of parent and formed species are given in **Tables S18** and **S19**. The structures of all POMs are shown in **Figure S80**. To identify the individual anions, they are shown in different colors, with the same color code being selected for a specific anion throughout all figures and tables in the main manuscript and the supporting information.

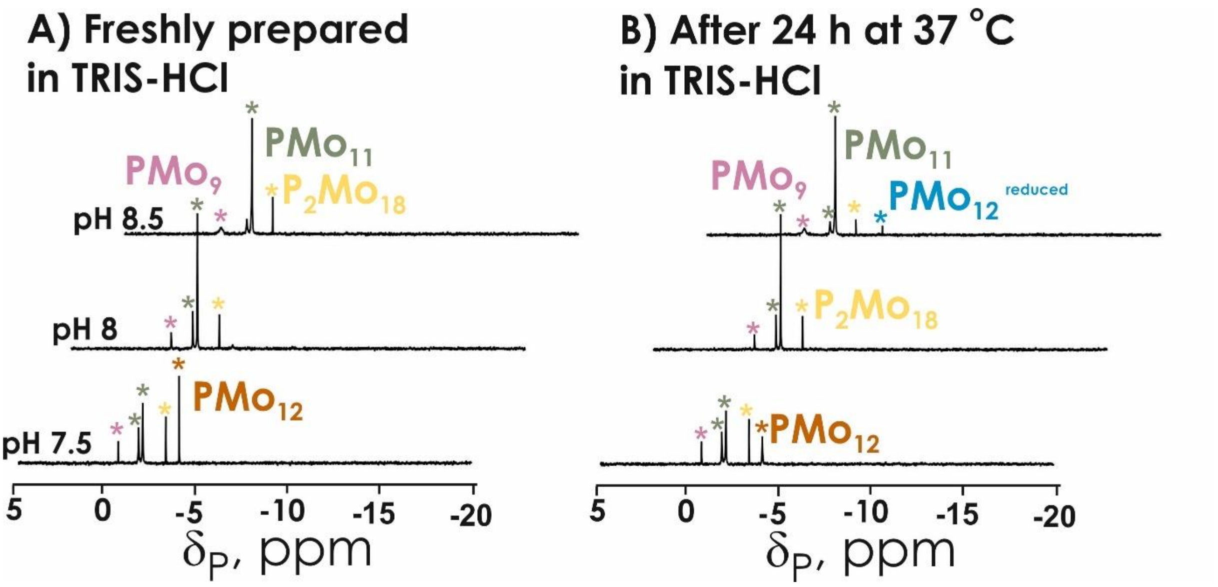

Fig. S88. ^{31}P NMR spectra of PMo$_{12}$ in tris-HCl buffer.

^{31}P NMR spectra for 10 mM solutions of **$H_3[\alpha\text{-}P^VMo^{VI}_{12}O_{40}]$** in 0.1 M tris-HCl buffer (pH 7.5 – 8.5) that were recorded approximately one hour after preparation (**A**) and after incubation for 24 h at 37 °C (**B**). The chemical shifts and percentages of parent and formed species are given in **Tables S18** and **S19**. The structures of all POMs are shown in **Figure S80**. To identify the individual anions, they are shown in different colors, with the same color code being selected for a specific anion throughout all figures and tables in the main manuscript and the supporting information.

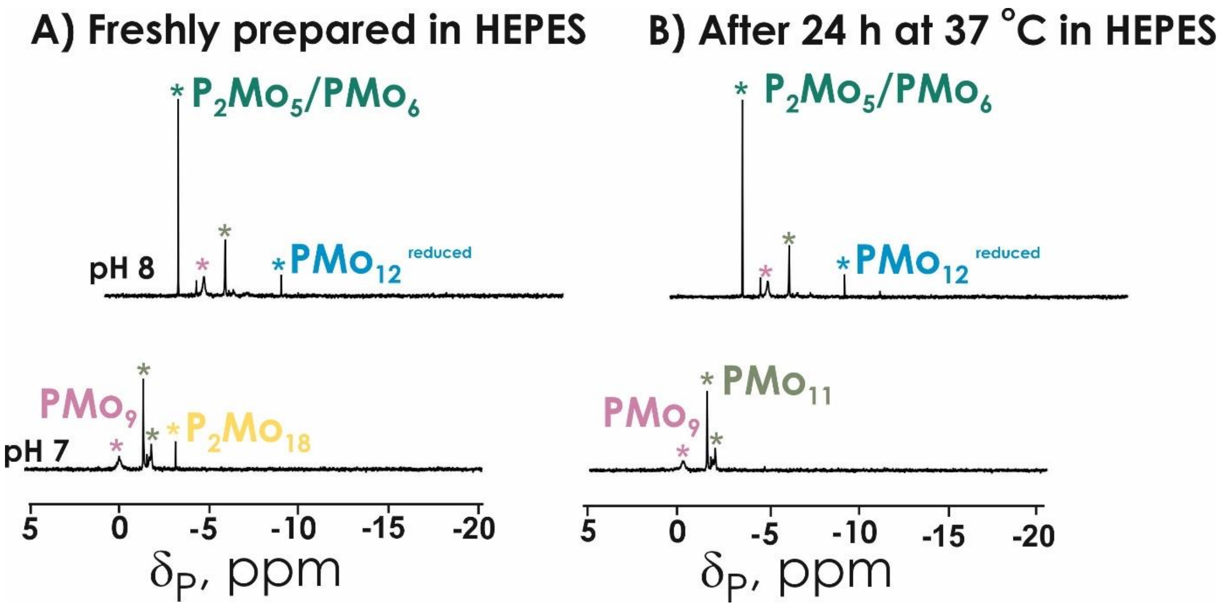

Fig. S89. ^{31}P NMR spectra of PMo_{12} in HEPES buffer.

^{31}P NMR spectra for 10 mM solutions of **$H_3[\alpha\text{-}P^{V}Mo^{VI}_{12}O_{40}]$** in 0.1 M HEPES buffer (pH 7 and 8) that were recorded approximately one hour after preparation (**A**) and after incubation for 24 h at 37 °C (**B**). The chemical shifts and percentages of parent and formed species are given in **Tables S18** and **S19**. The structures of all POMs are shown in **Figure S80**. To identify the individual anions, they are shown in different colors, with the same color code being selected for a specific anion throughout all figures and tables in the main manuscript and the supporting information.

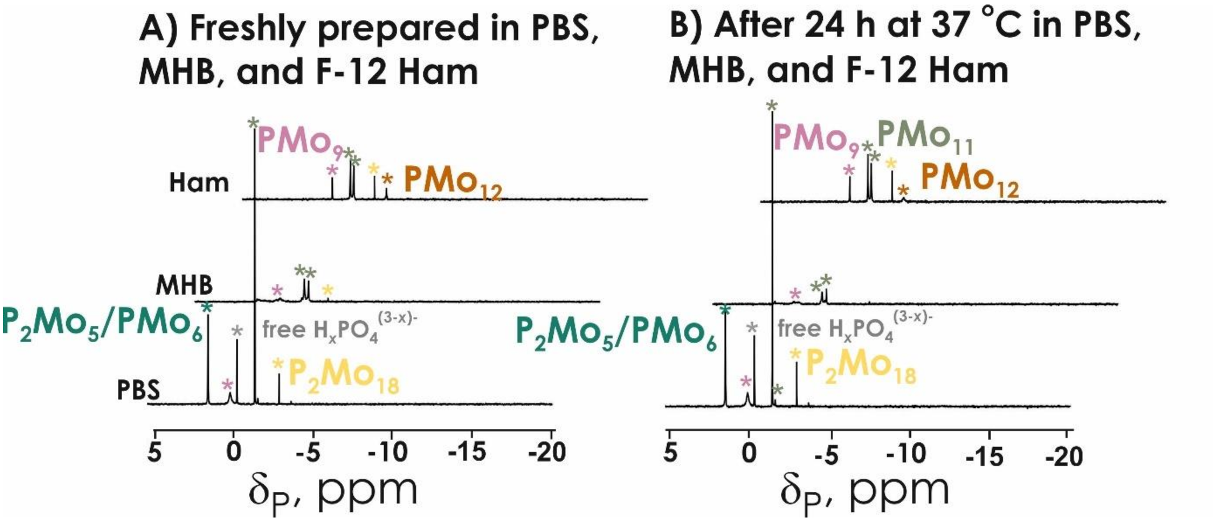

Fig. S90. ^{31}P NMR spectra of PMo$_{12}$ in solutions with pH 7.4.

^{31}P NMR spectra for 10 mM solutions of **$H_3[\alpha\text{-}P^VMo^{VI}{}_{12}O_{40}]$** in 0.1 M PBS, MHB and nutrient mixture F-12 Ham (pH 7.4) that were recorded approximately one hour after preparation (**A**) and after incubation for 24 h at 37 °C (**B**). The chemical shifts and percentages of parent and formed species are given in **Tables S18** and **S19**. The structures of all POMs are shown in **Figure S80**. To identify the individual anions, they are shown in different colors, with the same color code being selected for a specific anion throughout all figures and tables in the main manuscript and the supporting information.

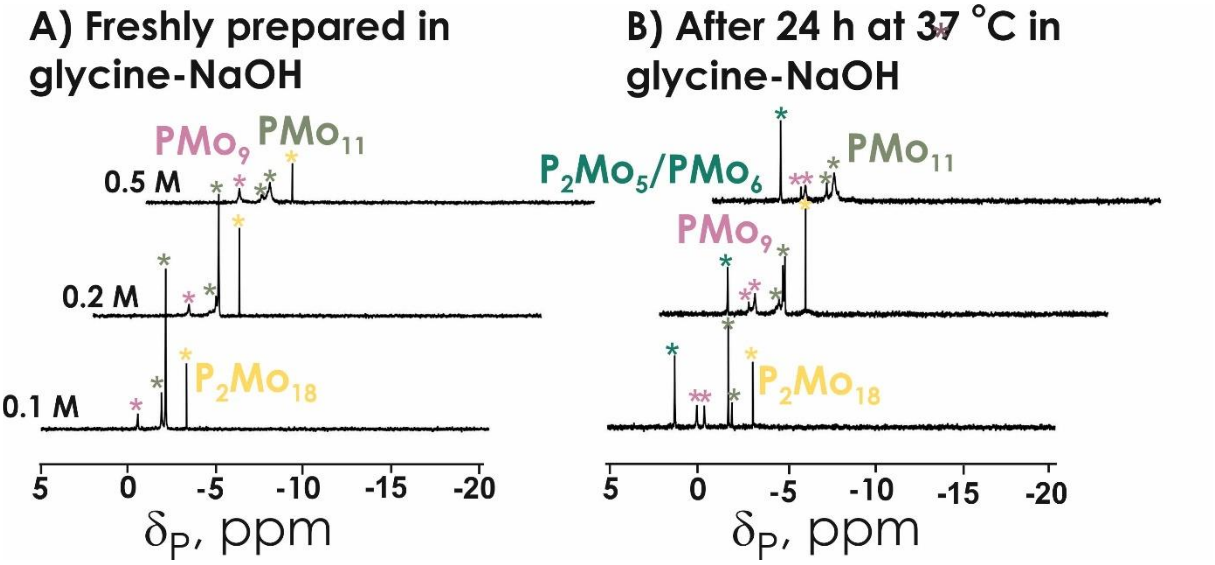

Fig. S91. ^{31}P NMR spectra of PMo_{12} in glycine-NaOH buffer.

^{31}P NMR spectra for 10 mM solutions of **$H_3[\alpha\text{-}P^{V}Mo^{VI}_{12}O_{40}]$** in glycine-NaOH (pH 8.6) with concentrations of 0.1, 0.2 and 0.5 M that were recorded approximately one hour after preparation (**A**) and after incubation for 24 h at 37 °C (**B**). The chemical shifts and percentages of parent and formed species are given in **Tables S18** and **S19**. The structures of all POMs are shown in **Figure S80**. To identify the individual anions, they are shown in different colors, with the same color code being selected for a specific anion throughout all figures and tables in the main manuscript and the supporting information.

Table S18. Analysis of NMR spectroscopic data recorded in PMo_{12} solutions at room temperature.

Chemical shifts in ^{31}P NMR spectra measured in triplicate of **$H_3[\alpha\text{-}P^VMo^{VI}{}_{12}O_{40}]$** (10 mM) dissolved in D_2O and 0.1 M buffers (acetic acid – sodium acetate pH 4 – 5.5; sodium phosphate pH 3 – 8 (while phosphate does not buffer at pH range from 3.5 – 5.5, experiments were conducted at this pH to provide comparisons to previously published studies (*29*)); citric acid – sodium citrate pH 3 – 6.5; MES pH 5.5; PBS pH 7.4; tris-HCl pH 7.5 – 8.5; HEPES pH 7 – 8; MHB pH 7.4; Nutrient mixture F-12 Ham pH 7.4 and glycine-NaOH pH 8.6) (**Figures S82** – **S91**). The glycine-NaOH buffer was used in two additional concentrations 0.2 and 0.5 M. The species content was calculated based on the integration of ^{31}P signals considering only signals associated with POTs. Signals were assigned based on the literature data summarized in **Table S4**.

pH	**$Na_3[PMo_{12}O_{40}]$** (10 mM) in Solvent / Buffer / Medium	Chemical shifts δ ^{31}P [ppm]	% of **PMo_{12}** in **$H_3[\alpha\text{-}P^VMo^{VI}{}_{12}O_{40}]$** (10 mM) at RT				% of **PMo_{11}** in **$H_3[\alpha\text{-}P^VMo^{VI}{}_{12}O_{40}]$** (10 mM) at RT				% of **PMo_9** in **$H_3[\alpha\text{-}P^VMo^{VI}{}_{12}O_{40}]$** (10 mM) at RT				% of **P_2Mo_{18}** in **$H_3[\alpha\text{-}P^VMo^{VI}{}_{12}O_{40}]$** (10 mM) at RT				% of **P_2Mo_5/PMo_6** in **$H_3[\alpha\text{-}P^VMo^{VI}{}_{12}O_{40}]$** (10 mM) at RT			
			Sample			Mean of 1 to 3 ± SD[a]	Sample			Mean of 1 to 3 ± SD[a]	Sample			Mean of 1 to 3 ± SD[a]	Sample			Mean of 1 to 3 ± SD[a]	Sample			Mean of 1 to 3 ± SD[a]
			#1	#2	#3		#1	#2	#3		#1	#2	#3		#1	#2	#3		#1	#2	#3	
-	D_2O	–0.5; –1.6; –3.1; –3.9	73	77	77	76 ± 2	9	9	10	9 ± 1	1	2	2	2 ± 1	17	12	11	13 ± 3	0	0	0	0
Strongly acidic environment 3 ≤ pH ≤ 4																						
3	0.1 M Sodium phosphate ($H_2PO_4^-/H_3PO_4$) pH 3	1.2; 0; –3.1	0	0	0	0	0	0	0	0	0	0	0	0	1	1	1	1 ± 0	99	99	99	99 ± 0
3	0.1 M Citric acid – sodium citrate (H_3Cit/H_2Cit^-) pH 3	0.2; –3.2	0	0	0	0	0	0	0	0	0	0	0	0	100	100	100	100 ± 0	0	0	0	0
4	0.1 M Sodium phosphate ($H_2PO_4^-/H_3PO_4$) pH 4	1.2; 0; –3.1	0	0	0	0	0	0	0	0	0	0	0	0	1	1	1	1 ± 0	99	99	99	99 ± 0
4	0.1 M Citric acid – sodium citrate ($H_2Cit^-/HCit^{2-}$) pH 4	0.1; –3.2	0	0	0	0	0	0	0	0	0	0	0	0	100	100	100	100 ± 0	0	0	0	0
4	0.1 M Acetic acid – sodium acetate ($OAc^-/HOAc$) pH 4	–0.5; –1.7; –1.9; –3.1; –3.9	23	22	24	23 ± 1	66	63	67	65 ± 2	6	6	5	6 ± 1	5	9	5	6 ± 2	0	0	0	0
Moderately acidic environment 5 ≤ pH ≤ 6																						

5	0.1 M Sodium phosphate ($H_2PO_4^-/H_3PO_4$) pH 5	1.2; 0; –3.2	0	0	0	0	0	0	0	0	0	0	0	0	1	1	1	1 ± 0	99	99	99	99 ± 0
	0.1 M Citric acid – sodium citrate ($H_2Cit^-/HCit^{2-}$) pH 5	0	0	0	0	0	0	0	0	0	0	0	0	0	0	0	0	0	0	0	0	0
	0.1 M Acetic acid – sodium acetate ($OAc^-/HOAc$) pH 5	–0.4; –1.9; –3.2	0	0	0	0	94	92	96	94 ± 2	0	0	0	0	6	8	4	6 ± 2	0	0	0	0
5.5	0.1 M Acetic acid – sodium acetate ($OAc^-/HOAc$) pH 5.5	–0.3; –1.6; –1.7; –2; –3.2	0	0	0	0	80	79	84	81 ± 3	14	13	10	12 ± 2	6	8	6	7 ± 1	0	0	0	0
	0.1 M MES[b] pH 5.5	–0.4; –1.6; –1.8; –3.1; –3.8	0	0	0	0	92	87	92	90 ± 3	3	4	3	3 ± 1	5	8	5	6 ± 2	0	0	0	0
6	0.1 M Sodium phosphate ($HPO_4^{2-}/H_2PO_4^-$) pH 6	1.2; 0; –3.1	0	0	0	0	0	0	0	0	0	0	0	0	1	1	1	1 ± 0	99	99	99	99 ± 0
	0.1 M Citric acid – sodium citrate ($HCit^{2-}/Cit^{3-}$) pH 6	0	0	0	0	0	0	0	0	0	0	0	0	0	0	0	0	0	0	0	0	0
Neutral environment 6.5 ≤ pH ≤ 7.5																						
6.5	0.1 M Citric acid – sodium citrate ($HCit^{2-}/Cit^{3-}$) pH 6.5	0	0	0	0	0	0	0	0	0	0	0	0	0	0	0	0	0	0	0	0	0
7	0.1 M Sodium phosphate ($HPO_4^{2-}/H_2PO_4^-$) pH 7	1.2; 0	0	0	0	0	0	0	0	0	0	0	0	0	0	0	0	0	100	100	100	100 ± 0
	0.1 M HEPES[c] pH 7	–0.1; –1.5; –1.7; –1.9; –3.2	0	0	0	0	78	78	78	78 ± 0	18	18	16	17 ± 1	4	4	6	5 ± 1	0	0	0	0
7.4	PBS[d] pH 7.4	1.3; –0.1; –0.5; –1.6; –1.8; –3.1	0	0	0	0	38	39	43	40 ± 3	30	31	31	31 ± 1	2	2	2	2 ± 0	29	28	24	27 ± 3

	MHB[e] pH 7.4	–0.2; –1.6; –1.7; –2.0; –3.2	0	0	0	0	100	98	98	99 ± 1	0	0	0	0	0	0	0	0	0	0	0	0
	Nutrient mixture F-12 Ham[f]	–0.5; –1.6; –1.9; –3.1; –3.9	12	11	10	11 ± 1	73	75	75	74 ± 1	9	9	9	9 ± 0	6	5	6	6 ± 1	0	0	0	0
7.5	0.1 M tris-HCl[g] pH 7.5	–0.5; –1.7; –1.9; –3.2; –3.9	22	20	24	22± 2	67	69	65	67 ± 2	5	5	5	5 ± 0	6	6	6	6 ± 0	0	0	0	0
Moderately alkaline environment 8 ≤ pH ≤ 8.6																						
8	0.1 M Sodium phosphate ($HPO_4^{2-}/H_2PO_4^{-}$) pH 8	1.2; 0.3	0	0	0	0	0	0	0	0	0	0	0	0	0	0	0	0	100	100	100	100 ± 0
	0.1 M HEPES pH 8	1.2; 0.1; –0.3; –1.5; –4.5	2	1	2	2 ± 1	31	37	32	33 ± 3	36	38	37	37 ± 1	0	0	0	0	31	24	29	28 ± 4
	0.1 M tris-HCl pH 8	–0.5; –1.7; –1.9; –3.2; –3.9	0	0	0	0	90	86	86	87 ± 3	5	6	7	6 ±1	5	8	7	7 ± 1	0	0	0	0
8.5	0.1 M tris-HCl pH 8.5	–0.3; –1.7; –2.0; –3.2	0	0	0	0	75	71	76	74 ± 3	21	24	18	21 ± 3	4	3	6	4 ± 2	0	0	0	0
8.6	0.1 M glycine-NaOH pH 8.6	–0.4; –1.8; –2.0; –3.2	0	0	0	0	87	87	86	87 ± 1	7	7	8	7 ± 1	6	6	6	6 ± 0	0	0	0	0
	0.2 M glycine-NaOH pH 8.6	–0.3; –1.9; –2.0; –3.2	0	0	0	0	83	83	86	84 ± 2	9	10	8	9 ± 1	8	6	6	7 ± 1	0	0	0	0
	0.5 M glycine-NaOH pH 8.6	–0.2; –1.5; –1.9; –3.2	0	0	0	0	74	74	76	75 ± 1	20	18	18	19 ± 1	5	5	6	5 ± 1	0	0	0	0

[a]SD – standard deviation; [b]MES – 2-(N-morpholino)ethanesulfonic acid, $C_6H_{13}NO_4S$ (Figure S1); [c]HEPES – 4-(2-hydroxyethyl)-1-piperazineethanesulfonic acid, $C_8H_{18}N_2O_4S$ (Figure S1); [d]PBS – phosphate buffer saline; [e]MHB – Mueller-Hinton broth, for more detailed information about composition, see https://labmal.com/2019/11/20/mueller-hinton-agar-and-mueller-hinton-broth/; [f]Nutrient mixture F-12 Ham contains sodium pyruvate (0.11 g/L), phenol red, L-glutamine, and does not contain $NaHCO_3$ and HEPES, for more details please see https://www.sigmaaldrich.com/AT/en/technical-documents/technical-article/cell-culture-and-cell-culture-analysis/mammalian-cell-culture/f-12-ham; [g]tris – tris(hydroxymethyl)aminomethane, $C_4H_{11}NO_3$ (Figure S1).

Table S19. Analysis of NMR spectroscopic data of PMo_{12} solutions investigated after 24 h incubation at 37 °C

Chemical shifts in ^{31}P NMR spectra measured in triplicate of **$H_3[\alpha\text{-}P^VMo^{VI}_{12}O_{40}]$** (10 mM) dissolved in D_2O and 0.1 M buffers (acetic acid – sodium acetate pH 4 – 5.5; sodium phosphate pH 3 – 8 (while phosphate does not buffer at pH range from 3.5 – 5.5, experiments were conducted at this pH to provide comparisons to previously published studies (*29*)); citric acid – sodium citrate pH 3 – 6.5; MES pH 5.5; PBS pH 7.4; tris-HCl pH 7.5 – 8.5; HEPES pH 7 – 8; MHB pH 7.4; Nutrient mixture F-12 Ham pH 7.4 and glycine-NaOH pH 8.6) and investigated after 24 h incubation at 37 °C (**Figures S82** – **S91**). The glycine-NaOH buffer was used in two additional concentrations 0.2 and 0.5 M. The species content was calculated based on the integration of ^{31}P signals considering only signals associated with POTs. Signals were assigned based on the literature data summarized in **Table S4**.

pH	**$Na_3[PMo_{12}O_{40}]$** (10 mM) in Solvent / Buffer / Medium	Chemical shifts δ ^{31}P [ppm]	% of **PMo_{12}** in **$H_3[\alpha\text{-}P^VMo^{VI}_{12}O_{40}]$** (10 mM) after 24 h incubation at 37 °C				% of **PMo_{11}** in **$H_3[\alpha\text{-}P^VMo^{VI}_{12}O_{40}]$** (10 mM) after 24 h incubation at 37 °C				% of **PMo_9** in **$H_3[\alpha\text{-}P^VMo^{VI}_{12}O_{40}]$** (10 mM) after 24 h incubation at 37 °C				% of **P_2Mo_{18}** in **$H_3[\alpha\text{-}P^VMo^{VI}_{12}O_{40}]$** (10 mM) after 24 h incubation at 37 °C				% of **P_2Mo_5/PMo_6** in **$H_3[\alpha\text{-}P^VMo^{VI}_{12}O_{40}]$** (10 mM) after 24 h incubation at 37 °C			
			Sample #1	Sample #2	Sample #3	Mean of 1 to 3 ± SD[a]	Sample #1	Sample #2	Sample #3	Mean of 1 to 3 ± SD[a]	Sample #1	Sample #2	Sample #3	Mean of 1 to 3 ± SD[a]	Sample #1	Sample #2	Sample #3	Mean of 1 to 3 ± SD[a]	Sample #1	Sample #2	Sample #3	Mean of 1 to 3 ± SD[a]
-	D_2O	–0.5; –1.6; –3.1; **–3.9**	71	76	77	74 ± 3	11	9	12	11 ± 2	2	2	1	2 ± 1	16	13	10	13 ± 3	0	0	0	0
Strongly acidic environment 3 ≤ pH ≤ 4																						
3	0.1 M Sodium phosphate ($H_2PO_4^-$/H_3PO_4) pH 3	**1.2**; 0; –3.1	0	0	0	0	0	0	0	0	0	0	0	0	1	1	1	1 ± 0	99	99	99	99 ± 0
3	0.1 M Citric acid – sodium citrate (H_3Cit/H_2Cit^-) pH 3	–0.2; –3.2	0	0	0	0	0	0	0	0	0	0	0	0	100	100	100	100 ± 0	0	0	0	0
4	0.1 M Sodium phosphate ($H_2PO_4^-$/H_3PO_4) pH 4	**1.2**; 0; –3.1	0	0	0	0	0	0	0	0	0	0	0	0	1	1	1	1 ± 0	99	99	99	99 ± 0
4	0.1 M Citric acid – sodium citrate (H_2Cit^-/$HCit^{2-}$) pH 4	–0.1; –3.2	0	0	0	0	0	0	0	0	0	0	0	0	100	100	100	100 ± 0	0	0	0	0
4	0.1 M Acetic acid – sodium acetate (OAc^-/HOAc) pH 4	–0.5; –1.7; –1.9; –3.2; **–3.9**	25	22	23	23 ± 2	64	65	65	65 ± 1	6	5	6	6 ± 1	5	8	6	6 ± 2	0	0	0	0
Moderately acidic environment 5 ≤ pH ≤ 6																						
5	0.1 M Sodium phosphate ($H_2PO_4^-$/H_3PO_4) pH 5	**1.2**; 0; –3.1	0	0	0	0	0	0	0	0	0	0	0	0	1	1	1	1 ± 0	99	99	99	99 ± 0

	0.1 M Citric acid – sodium citrate (H_2Cit^-/$HCit^{2-}$) pH 5	0	0	0	0	0	0	0	0	0	0	0	0	0	0	0	0	0	0	0	0	0
	0.1 M Acetic acid – sodium acetate (OAc^-/HOAc) pH 5	–0.4; –1.9; –3.2	0	0	0	0	97	96	98	97 ± 1	0	0	0	0	3	4	2	3 ± 1	0	0	0	0
5.5	0.1 M Acetic acid – sodium acetate (OAc^-/HOAc) pH 5.5	–0.3; –1.6; –1.7; –2.0	0	0	0	0	88	86	89	88 ± 2	12	14	11	12 ± 2	0	0	0	0	0	0	0	0
	0.1 M MES[b] pH 5.5	–0.4; –1.6; –1.8; –3.1	0	0	0	0	92	88	93	91 ± 3	2	4	3	3 ± 1	6	8	4	6 ± 2	0	0	0	0
6	0.1 M Sodium phosphate (HPO_4^{2-}/$H_2PO_4^-$) pH 6	1.2; 0; –3.1	0	0	0	0	0	0	0	0	0	0	0	0	1	1	1	1 ± 0	99	99	99	99 ± 0
	0.1 M Citric acid – sodium citrate ($HCit^{2-}$/Cit^{3-}) pH 6	0	0	0	0	0	0	0	0	0	0	0	0	0	0	0	0	0	0	0	0	0
Neutral environment 6.5 ≤ pH ≤ 7.5																						
6.5	0.1 M Citric acid – sodium citrate ($HCit^{2-}$/Cit^{3-}) pH 6.5	0	0	0	0	0	0	0	0	0	0	0	0	0	0	0	0	0	0	0	0	0
7	0.1 M Sodium phosphate (HPO_4^{2-}/$H_2PO_4^-$) pH 7	1.2; 0	0	0	0	0	0	0	0	0	0	0	0	0	0	0	0	0	100	100	100	100 ± 0
	0.1 M HEPES[c] pH 7	–0.1; –1.5; –1.7; –1.8; –1.9	0	0	0	0	82	85	84	84 ± 2	18	15	16	16 ± 2	0	0	0	0	0	0	0	0
7.4	PBS[d] pH 7.4	1.3; –0.1; –0.5; –1.6; –1.8; –3.1	0	0	0	0	39	38	41	39 ± 2	30	29	31	30 ± 1	4	4	3	4 ± 1	27	29	25	27 ± 2
	MHB[e] pH 7.4	1.2; 0; –1.8; –2.0	0	0	0	0	100	95	92	95 ± 4	0	3	6	0	0	0	0	0	0	2	2	1 ± 1
	Nutrient mixture F-12 Ham[f]	–0.5; –1.6; –1.9; –3.1; –3.9	3	4	4	4 ± 1	81	79	79	80 ± 1	10	11	10	10 ± 1	6	6	7	6 ± 1	0	0	0	0
7.5	0.1 M tris-HCl[g] pH 7.5	–0.5; –1.7; –1.9; –3.2; –3.9	20	16	21	19 ± 3	68	69	66	68 ± 2	6	6	6	6 ± 0	6	8	7	7 ± 1	0	0	0	0
Moderately alkaline environment 8 ≤ pH ≤ 8.6																						

8	0.1 M Sodium phosphate (HPO_4^{2-}/$H_2PO_4^{-}$) pH 8	1.2; 0.3	0	0	0	0	0	0	0	0	0	0	0	0	0	0	0	0	100	100	100	100 ± 0
	0.1 M HEPES pH 8	1.1; 0.1; –0.3; –1.5; –2; –4.6	2	3	3	3 ± 1	26	32	27	28 ± 3	33	36	34	34 ± 2	0	0	0	0	38	30	36	35 ± 4
	0.1 M tris-HCl pH 8	–0.5; –1.7; –1.9; –3.2	0	0	0	0	88	86	86	87 ± 1	6	6	7	6 ±1	6	8	7	7 ± 1	0	0	0	0
8.5	0.1 M tris-HCl pH 8.5	–0.3; –1.7; –2.0; –3.2; –4.6	0	0	0	0	72	73	76	74 ± 2	25	25	23	24 ± 1	1	0	0	1 ± 0	0	2	0	1 ± 1
8.6	0.1 M glycine-NaOH pH 8.6	1.2; 0.1; –0.5; –1.8; –2.0; –3.2	0	0	0	0	39	33	38	37 ± 3	13	17	21	17 ± 4	7	7	6	6 ± 1	36	43	35	38 ± 4
	0.2 M glycine-NaOH pH 8.6	1.2; 0; –0.3; –1.7; –1.9; –2.0; –3.2	0	0	0	0	59	50	45	51 ± 7	18	17	19	18 ± 1	9	6	7	7 ± 1	13	27	29	23 ± 8
	0.5 M glycine-NaOH pH 8.6	1.2; 0; –0.2; –1.4; –1.8	0	0	0	0	59	62	60	60 ± 2	13	14	18	15 ± 3	0	0	0	0	25	24	24	24 ± 1

[a]SD – standard deviation; [b]MES – 2-(N-morpholino)ethanesulfonic acid, $C_6H_{13}NO_4S$ (Figure S1); [c]HEPES – 4-(2-hydroxyethyl)-1-piperazineethanesulfonic acid, $C_8H_{18}N_2O_4S$ (Figure S1); [d]PBS – phosphate buffer saline; [e]MHB – Mueller-Hinton broth, for more detailed information about composition, see https://labmal.com/2019/11/20/mueller-hinton-agar-and-mueller-hinton-broth/; [f]Nutrient mixture F-12 Ham contains sodium pyruvate (0.11 g/L), phenol red, L-glutamine, and does not contain $NaHCO_3$ and HEPES, for more details please see https://www.sigmaaldrich.com/AT/en/technical-documents/technical-article/cell-culture-and-cell-culture-analysis/mammalian-cell-culture/f-12-ham; [g]tris – tris(hydroxymethyl)aminomethane, $C_4H_{11}NO_3$ (Figure S1).

9.3. Speciation in PMo_{12} Keggin POMo solutions

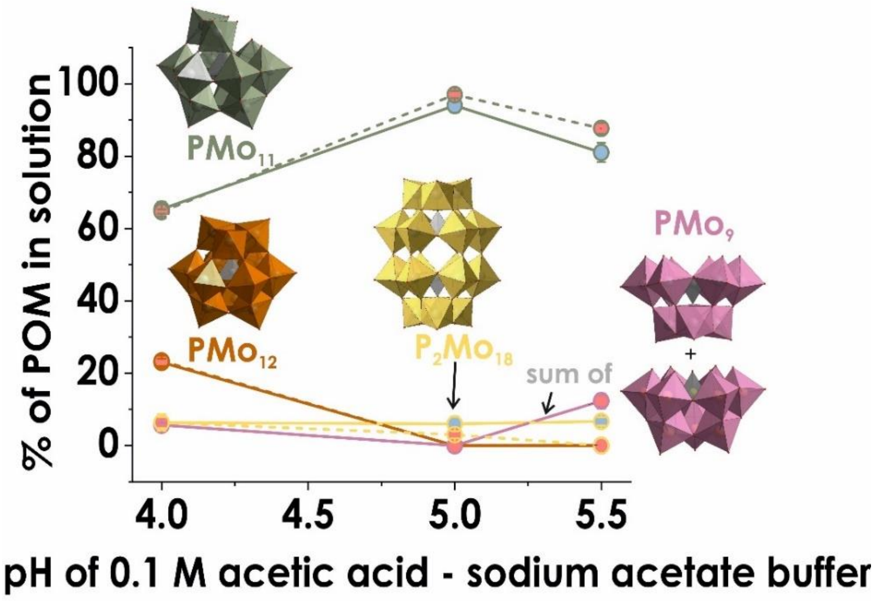

Fig. S92. Speciation of PMo_{12} in acetic acid – sodium acetate buffer.

POM concentration curves in **$H_3[\alpha\text{-}P^{V}Mo^{VI}_{12}O_{40}]$** (10 mM) in 0.1 M acetic acid – sodium acetate buffer solutions before (solid line, blue dot in the middle) and after incubation (dash line, red dot in the middle) for 24 h at 37 °C. The exact percentage of all POM species present is given in **Tables S18** and **S19**.

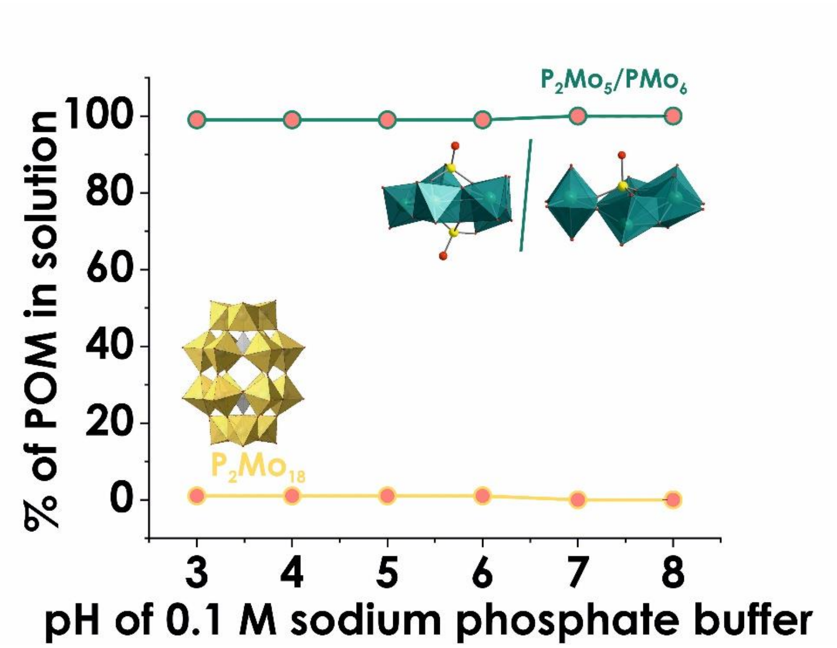

Fig. S93. Speciation of PMo_{12} in sodium phosphate buffer.

POM concentration curves in **$H_3[\alpha\text{-}P^VMo^{VI}_{12}O_{40}]$** (10 mM) in 0.1 M sodium phosphate buffer solutions before (solid line, blue dot in the middle) and after incubation (dash line, red dot in the middle) for 24 h at 37 °C. The exact percentage of all POM species present is given in **Tables S18** and **S19**.

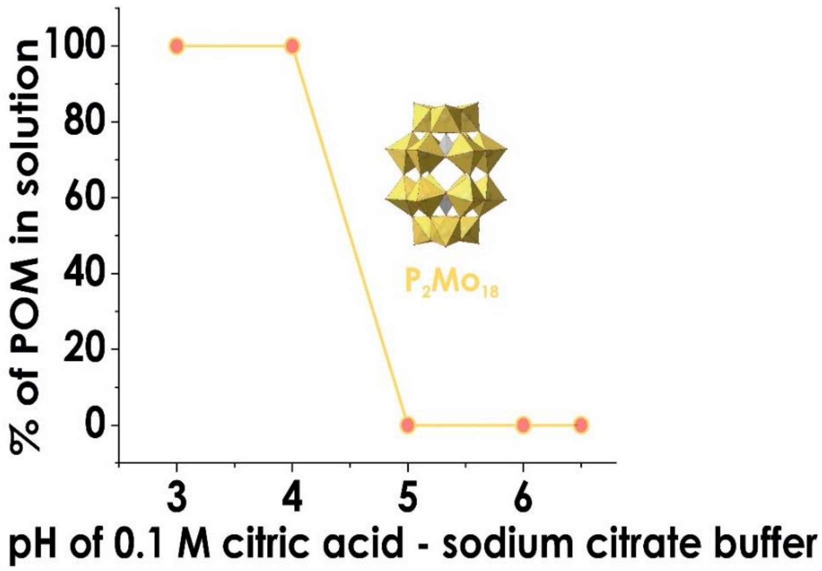

Fig. S94. Speciation of PMo_{12} in citric acid – sodium citrate buffer.

POM concentration curves in $\mathbf{H_3[\alpha\text{-}P^{V}Mo^{VI}_{12}O_{40}]}$ (10 mM) in 0.1 M citric acid – sodium citrate buffer solutions before (solid line, blue dot in the middle) and after incubation (dash line, red dot in the middle) for 24 h at 37 °C. The exact percentage of all POM species present is given in **Tables S18** and **S19**.

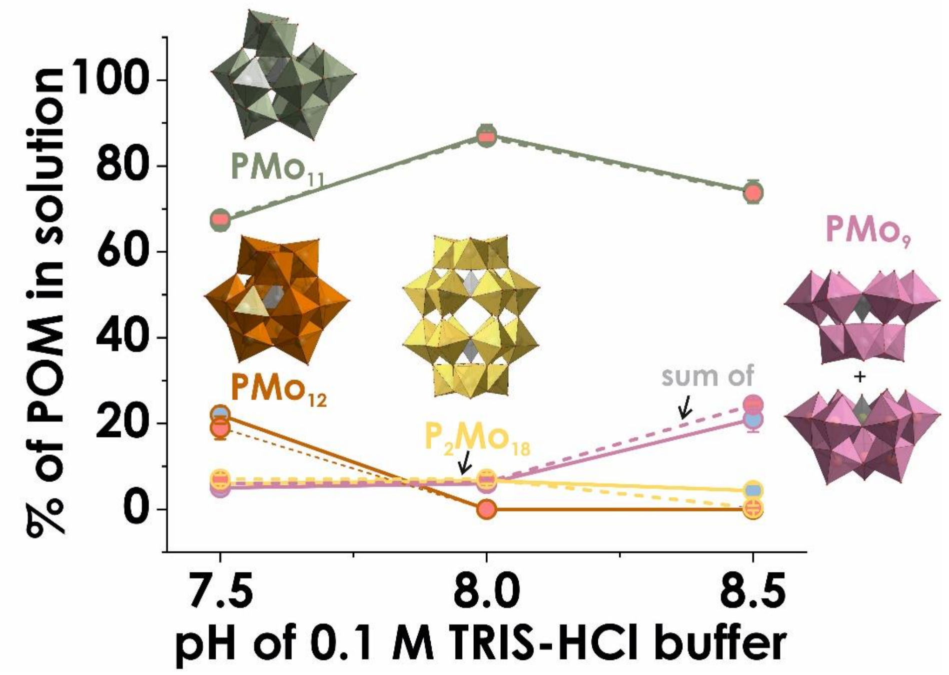

Fig. S95. Speciation of PMo12 in tris-HCl buffer.

POM concentration curves in **$H_3[\alpha\text{-}P^{V}Mo^{VI}_{12}O_{40}]$** (10 mM) in 0.1 M tris-HCl buffer solutions before (solid line, blue dot in the middle) and after incubation (dash line, red dot in the middle) for 24 h at 37 °C. The exact percentage of all POM species present is given in **Tables S18** and **S19**.

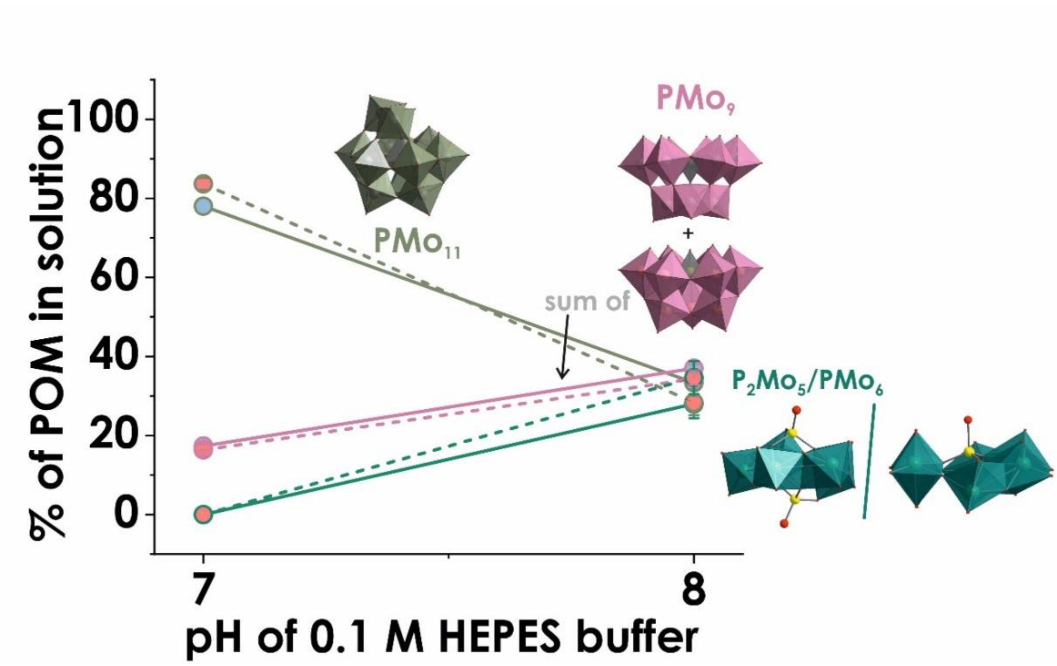

Fig. S96. Speciation of PMo12 in HEPES buffer.

POM concentration curves in **$H_3[\alpha\text{-}P^{V}Mo^{VI}_{12}O_{40}]$** (10 mM) in 0.1 M HEPES buffer solutions before (solid line, blue dot in the middle) and after incubation (dash line, red dot in the middle) for 24 h at 37 °C. The exact percentage of all POM species present is given in **Tables S18** and **S19**.

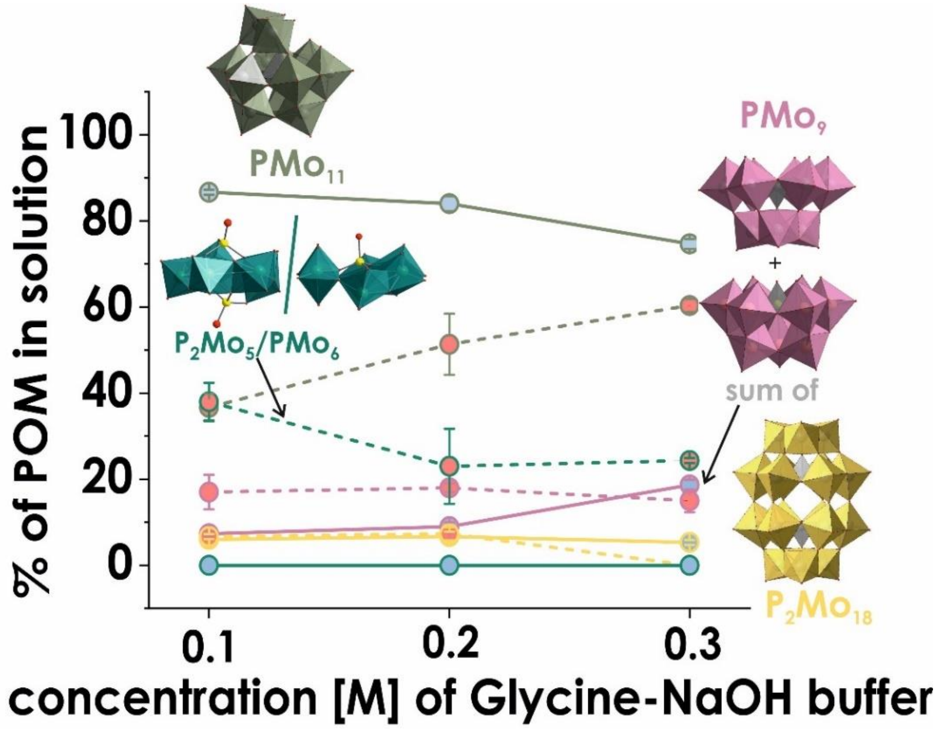

Fig. S97. Speciation of PMo12 in glycine-NaOH buffer.

POM concentration curves in **$H_3[\alpha\text{-}P^VMo^{VI}{}_{12}O_{40}]$** (10 mM) in glycine-NaOH (pH 8.6) with concentrations of 0.1. 0.2 and 0.5 M solutions before (solid line, blue dot in the middle) and after incubation (dash line, red dot in the middle) for 24 h at 37 °C. The exact percentage of all POM species present is given in **Tables S18** and **S19**.

10. The P_2Mo_{18} Wells-Dawson POMo

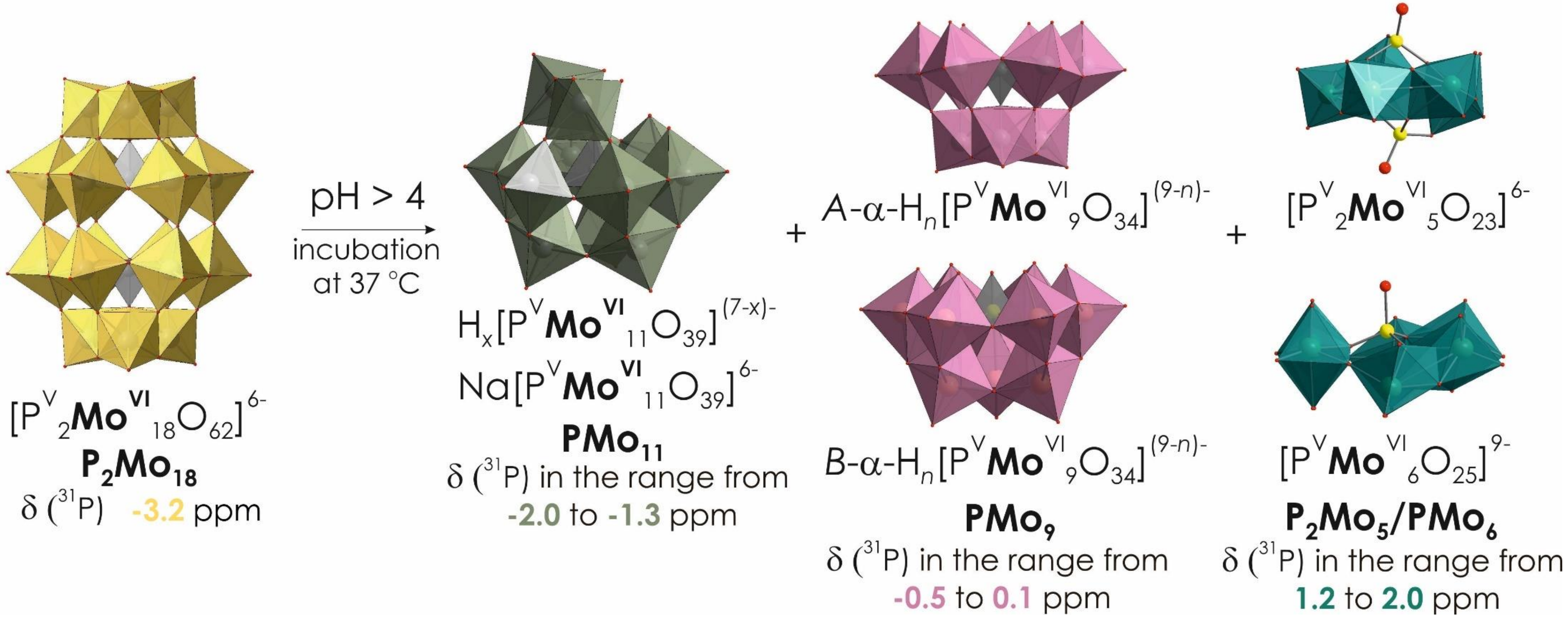

Fig. S98. Hydrolysis of P_2Mo_{18}.

Structure of the Wells-Dawson POMo $[P^V{}_2Mo^{VI}{}_{18}O_{62}]^{6-}$ (**P_2Mo_{18}**) and its hydrolysis scheme based on the current investigation. The polyanions that can be formed during hydrolysis in the pH range from 4 to 8.6 are shown with corresponding ^{31}P NMR chemical shifts. Color code: {MoO_6}, light-brown, gray-green, pink, turquoise, or yellow; {PO_4}, grey or yellow; O, red. To identify the individual anions, they are shown in different colors, with the same color code being selected for a specific anion throughout all figures and tables in the main manuscript and the supporting information.

10.1. pH of Wells-Dawson P_2Mo_{18} POMo solutions

Table S20. pH in P_2Mo_{18} solutions.

pH values measured in triplicate of **$(NH_4)_6[\alpha\text{-}P^V_2Mo^{VI}_{18}O_{62}]$** (10 mM) dissolved D_2O and different buffers (acetic acid – sodium acetate pH 4 – 5.5; sodium phosphate pH 3 – 8 (while phosphate does not buffer at pH range from 3.5 – 5.5, experiments were conducted at this pH to provide comparisons to previously published studies (*29*)); citric acid – sodium citrate pH 3 – 6.5; MES pH 5.5; PBS pH 7.4; tris-HCl pH 7.5 – 8.5; HEPES pH 7 – 8; MHB pH 7.4; Nutrient mixture F-12 Ham pH 7.4 and glycine-NaOH pH 8.6) with concentration 0.1 M. The glycine-NaOH buffer was used in two additional concentrations 0.2 and 0.5 M.

pH	**$(NH_4)_6[P_2Mo_{18}O_{62}]$** (10 mM) in Solvent / Buffer / Medium	pH after dissolving **$(NH_4)_6[\alpha\text{-}P^V_2Mo^{VI}_{18}O_{62}]$** (10 mM) at RT				pH after 24 h incubation of **$(NH_4)_6[\alpha\text{-}P^V_2Mo^{VI}_{18}O_{62}]$** (10 mM) solution at 37 °C			
		Sample			Mean of 1 to 3 ± SD[a]	Sample			Mean of 1 to 3 ± SD[a]
		#1	#2	#3		#1	#2	#3	
-	D_2O	3.89	3.81	3.74	3.81 ± 0.08	2.90	2.73	2.76	2.80 ± 0.09
Strongly acidic environment 3 ≤ pH ≤ 4									
3	0.1 M Sodium phosphate ($H_2PO_4^-$/H_3PO_4) pH 3	2.86	2.88	2.82	2.85 ± 0.03	2.15	2.24	2.12	2.17 ± 0.06
	0.1 M Citric acid – sodium citrate (H_3Cit/H_2Cit^-) pH 3	2.04	2.03	2.02	2.03 ± 0.01	1.53	1.58	1.32	1.48 ± 0.14
4	0.1 M Sodium phosphate ($H_2PO_4^-$/H_3PO_4) pH 4	3.31	3.34	3.39	3.35 ± 0.04	2.31	2.40	2.27	2.33 ± 0.07
	0.1 M Citric acid – sodium citrate (H_2Cit^-/$HCit^{2-}$) pH 4	2.75	2.76	3.32	2.94 ± 0.33	2.08	2.34	2.19	2.20 ± 0.13
	0.1 M Acetic acid – sodium acetate (OAc^-/HOAc) pH 4	3.93	3.93	3.90	3.92 ± 0.02	3.24	3.19	3.29	3.24 ± 0.05
Moderately acidic environment 5 ≤ pH ≤ 6									
5	0.1 M Sodium phosphate ($H_2PO_4^-$/H_3PO_4) pH 5	3.43	3.42	3.51	3.45 ± 0.05	2.40	2.42	2.29	2.37 ± 0.07
	0.1 M Citric acid – sodium citrate (H_2Cit^-/$HCit^{2-}$) pH 5	4.58	4.69	4.66	4.64 ± 0.06	4.54	4.40	4.31	4.42 ± 0.12
	0.1 M Acetic acid – sodium acetate (OAc^-/HOAc) pH 5	4.74	4.70	4.66	4.70 ± 0.04	4.24	4.15	4.22	4.20 ± 0.05

5.5	0.1 M Acetic acid – sodium acetate ($OAc^–$/HOAc) pH 5.5	4.99	4.89	4.87	4.92 ± 0.06	4.45	4.47	4.54	4.49 ± 0.05
	0.1 M MES[b] pH 5.5	4.02	3.91	3.87	3.93 ± 0.08	2.69	2.6	2.62	2.64 ± 0.05
6	0.1 M Sodium phosphate ($HPO_4^{2–}$/$H_2PO_4^–$) pH 6	3.90	3.80	4.30	4.00 ± 0.26	2.75	2.70	2.51	2.65 ± 0.13
	0.1 M Citric acid – sodium citrate ($HCit^{2–}$/$Cit^{3–}$) pH 6	5.55	5.81	5.8	5.72 ± 0.15	5.44	5.22	5.55	5.40 ± 0.17
Neutral environment 6.5 ≤ pH ≤ 7.5									
6.5	0.1 M Citric acid – sodium citrate ($HCit^{2–}$/$Cit^{3–}$) pH 6.5	5.66	6.29	6.23	6.06 ± 0.35	5.57	5.49	5.63	5.56 ± 0.07
7	0.1 M Sodium phosphate ($HPO_4^{2–}$/$H_2PO_4^–$) pH 7	5.99	5.84	6.32	6.05 ± 0.25	5.89	5.74	6.07	5.90 ± 0.17
	0.1 M HEPES[c] pH 7	5.19	5.18	5.02	5.13 ± 0.10	4.41	4.64	4.59	4.55 ± 0.12
7.4	PBS[d] pH 7.4	4.15	4.05	4.04	4.08 ± 0.06	2.53	2.63	2.54	2.58 ± 0.04
	MHB[e] pH 7.4	4.73	4.98	4.70	4.80 ± 0.15	3.95	3.79	3.78	3.84 ± 0.10
	Nutrient mixture F-12 Ham[f]	4.46	4.36	4.30	4.37 ± 0.08	2.79	2.92	3.13	2.95 ± 0.17
7.5	0.1 M tris-HCl[g] pH 7.5	4.59	4.52	4.52	4.54 ± 0.04	3.00	3.12	3.24	3.12 ± 0.12
Moderately alkaline environment 8 ≤ pH ≤ 8.6									
8	0.1 M Sodium phosphate ($HPO_4^{2–}$/$H_2PO_4^–$) pH 8	6.02	5.93	6.29	6.08 ± 0.19	5.97	5.85	6.04	5.95 ± 0.10
	0.1 M HEPES pH 8	5.62	5.57	5.81	5.67 ± 0.13	5.39	5.37	5.23	5.33 ± 0.09
	0.1 M tris-HCl pH 8	4.91	4.70	4.68	4.76 ± 0.13	3.41	3.45	3.62	3.49 ± 0.11
8.5	0.1 M tris-HCl pH 8.5	5.32	5.12	5.16	5.20 ± 0.11	4.47	4.52	4.78	4.59 ± 0.17
8.6	0.1 M glycine-NaOH pH 8.6	4.41	4.55	4.58	4.51 ± 0.09	3.48	3.31	3.45	3.41 ± 0.09
	0.2 M glycine-NaOH pH 8.6	4.60	4.88	5.11	4.86 ± 0.26	3.91	3.78	3.89	3.86 ± 0.07
	0.5 M glycine-NaOH pH 8.6	4.76	4.94	5.23	4.98 ± 0.24	4.50	4.33	4.46	4.43 ± 0.09

[a]SD – standard deviation; [b]MES – 2-(N-morpholino)ethanesulfonic acid, $C_6H_{13}NO_4S$ (Figure S1); [c]HEPES – 4-(2-hydroxyethyl)-1-piperazineethanesulfonic acid, $C_8H_{18}N_2O_4S$ (Figure S1); [d]PBS – phosphate buffer saline; [e]MHB – Mueller-Hinton broth, for more detailed information about composition, see https://labmal.com/2019/11/20/mueller-hinton-agar-and-mueller-hinton-broth/; [f]Nutrient mixture F-12 Ham contains sodium pyruvate (0.11 g/L), phenol red, L-glutamine, and does not contain $NaHCO_3$ and HEPES, for more details please see https://www.sigmaaldrich.com/AT/en/technical-documents/technical-article/cell-culture-and-cell-culture-analysis/mammalian-cell-culture/f-12-ham; [g]tris – tris(hydroxymethyl)aminomethane, $C_4H_{11}NO_3$ (Figure S1).

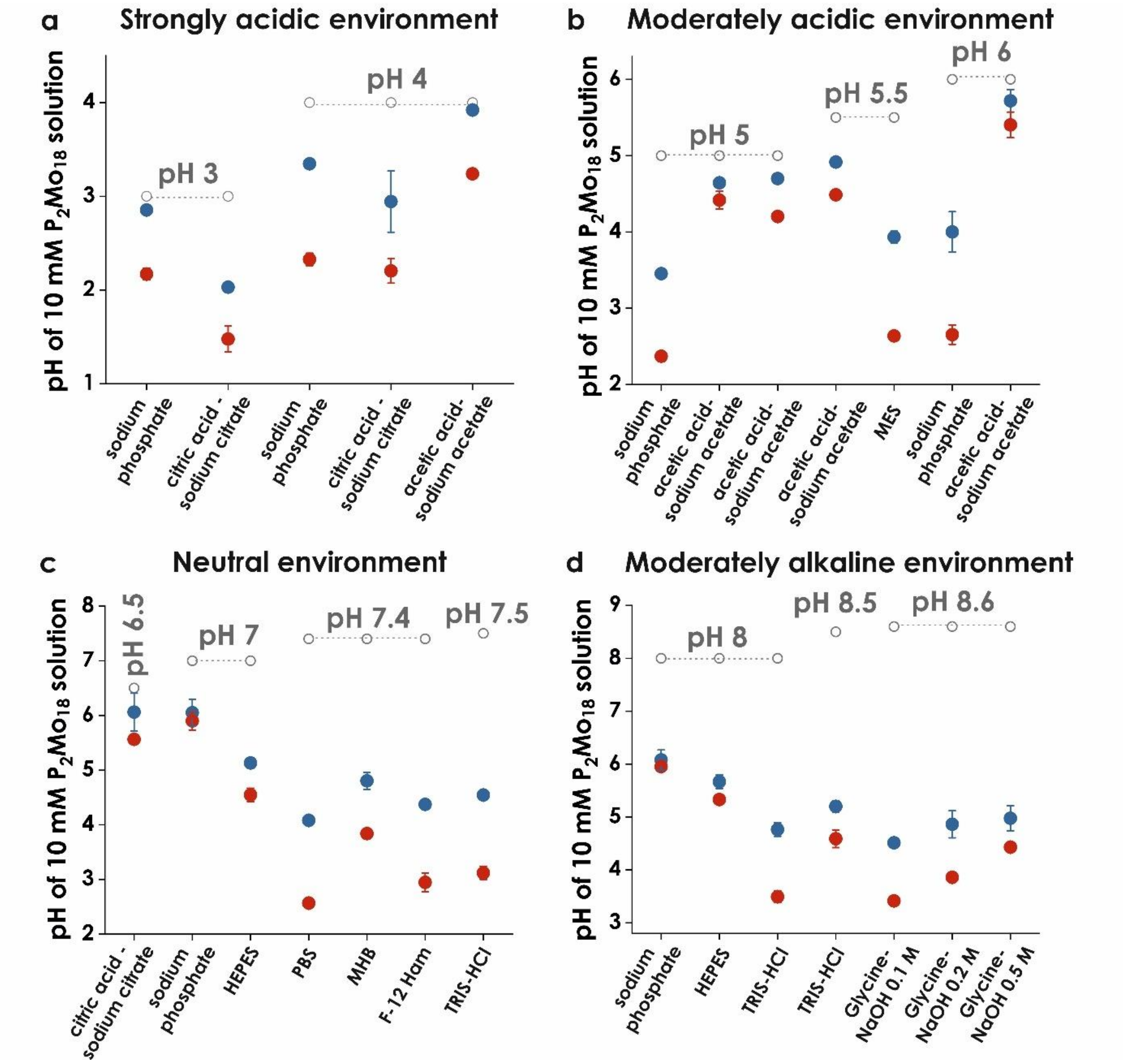

Fig. S99. pH in P_2Mo_{18} solutions.

The pH dependence curves for 10 mM solutions of **$(NH_4)_6[\alpha\text{-}P^{V}_2Mo^{VI}_{18}O_{62}]$** in different buffers in a) strongly acidic, b) moderately acidic, c) neutral, and d) moderately alkaline environments. The pH of the starting buffers is shown with a gray dashed line; the plots of the measured pH values immediately after the preparation of the solutions are shown in blue and after 24 h incubation at 37 °C in red. The error bar shows standard deviation (**Table S20**).

10.2. ^{31}P NMR spectroscopic studies of P_2Mo_{18} Wells-Dawson POMo solutions

All ^{31}P peaks with the highest intensity were unambiguously assigned based on the literature data from **Table S4**. In some spectra, chemical shifts for peaks of lower intensity have not yet been described in the literature and therefore can not be assigned in this work.

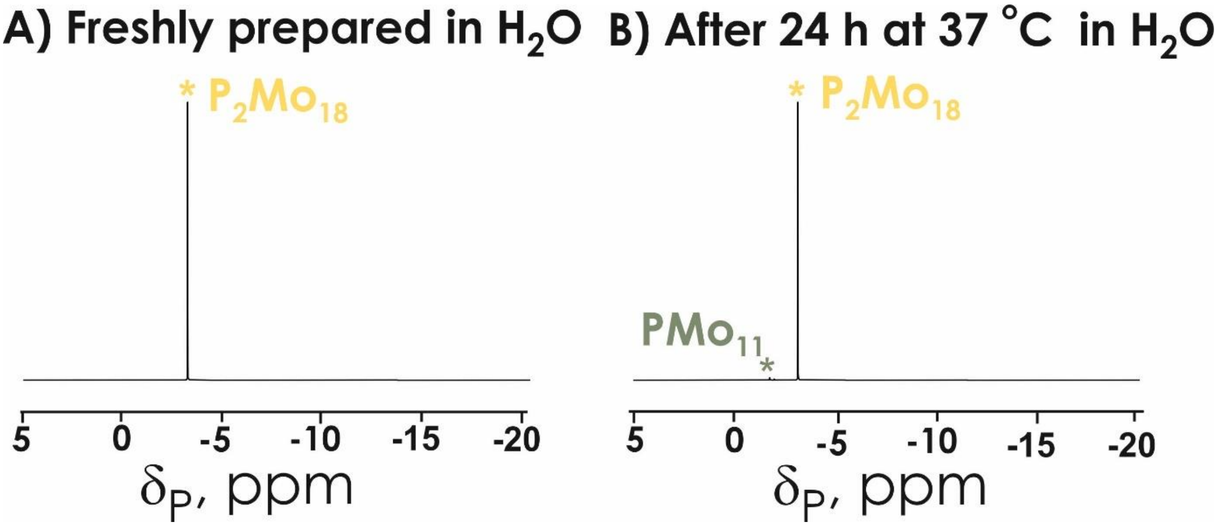

Fig. S100. ^{31}P NMR spectra of P_2Mo_{18} in H_2O.

^{31}P NMR spectra for 10 mM solutions of **$(NH_4)_6[\alpha\text{-}P^{V}{}_2Mo^{VI}{}_{18}O_{62}]$** in D_2O that were recorded approximately one hour after preparation (**A**) and after incubation for 24 h at 37 °C (**B**). The chemical shifts and percentages of parent and formed species are given in **Tables S21** and **S22**. The structures of all POMs are shown in **Figure S98**. To identify the individual anions, they are shown in different colors, with the same color code being selected for a specific anion throughout all figures and tables in the main manuscript and the supporting information.

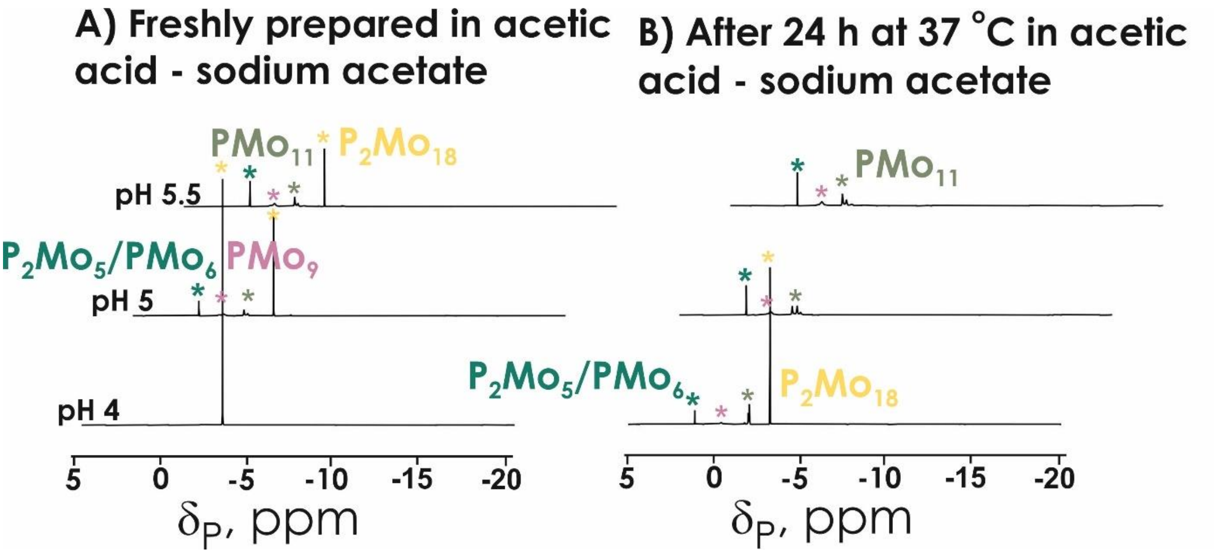

Fig. S101. ^{31}P NMR spectra of P_2Mo_{18} in NaOAc/HOAc buffer.

^{31}P NMR spectra for 10 mM solutions of **$(NH_4)_6[\alpha\text{-}P^V{}_2Mo^{VI}{}_{18}O_{62}]$** in 0.1 M NaOAc/HOAc buffer (pH 4 – 5.5) that were recorded approximately one hour after preparation (**A**) and after incubation for 24 h at 37 °C (**B**). The chemical shifts and percentages of parent and formed species are given in **Tables S21** and **S22**. The structures of all POMs are shown in **Figure S98**. To identify the individual anions, they are shown in different colors, with the same color code being selected for a specific anion throughout all figures and tables in the main manuscript and the supporting information.

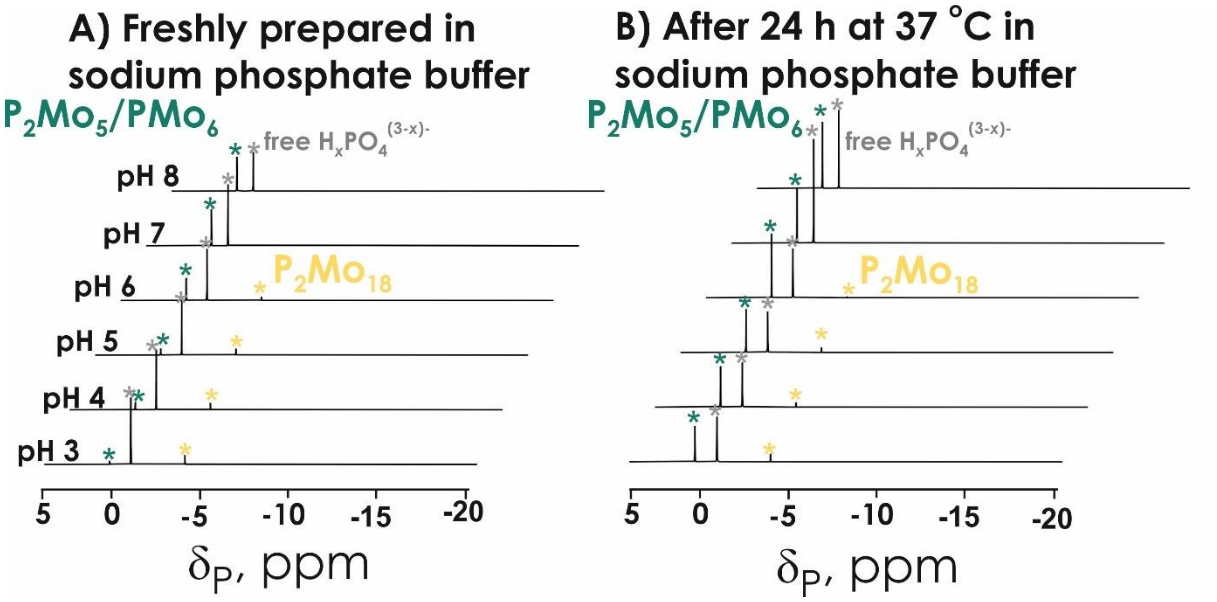

Fig. S102. ^{31}P NMR spectra of P_2Mo_{18} in sodium phosphate buffer.

^{31}P NMR spectra for 10 mM solutions of **$(NH_4)_6[\alpha\text{-}P^{V}_2Mo^{VI}_{18}O_{62}]$** in 0.1 M sodium phosphate buffer (pH 3 – 8) that were recorded approximately one hour after preparation (**A**) and after incubation for 24 h at 37 °C (**B**). The chemical shifts and percentages of parent and formed species are given in **Tables S21** and **S22**. The structures of all POMs are shown in **Figure S98**. To identify the individual anions, they are shown in different colors, with the same color code being selected for a specific anion throughout all figures and tables in the main manuscript and the supporting information.

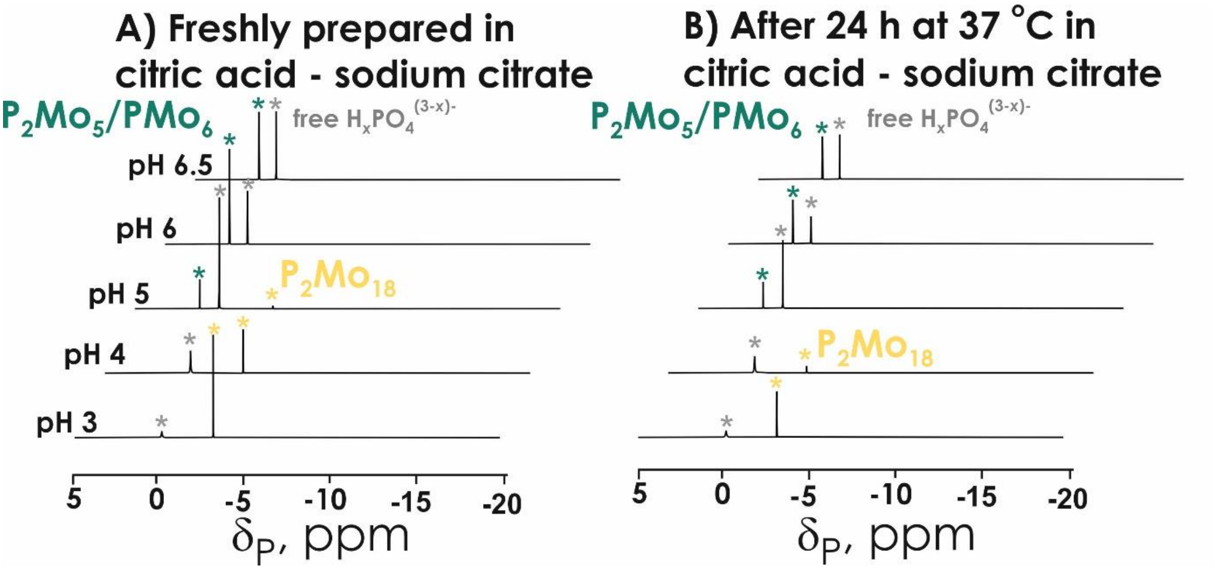

Fig. S103. ^{31}P NMR spectra of P_2Mo_{18} in citric acid – sodium citrate buffer.

^{31}P NMR spectra for 10 mM solutions of **$(NH_4)_6[\alpha\text{-}P^{V}_2Mo^{VI}_{18}O_{62}]$** in 0.1 M citric acid – sodium citrate buffer (pH 3 – 6.5) that were recorded approximately one hour after preparation (**A**) and after incubation for 24 h at 37 °C (**B**). The chemical shifts and percentages of parent and formed species are given in **Tables S21** and **S22**. The structures of all POMs are shown in **Figure S98**. To identify the individual anions, they are shown in different colors, with the same color code being selected for a specific anion throughout all figures and tables in the main manuscript and the supporting information.

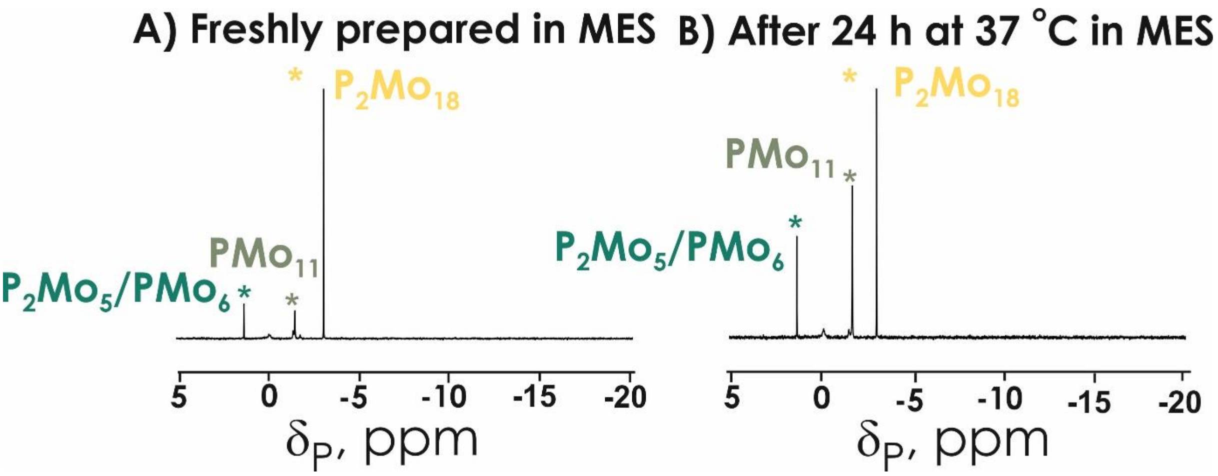

Fig. S104. ^{31}P NMR spectra of P_2Mo_{18} in MES buffer.

^{31}P NMR spectra for 10 mM solutions of **$(NH_4)_6[\alpha\text{-}P^{V}_2Mo^{VI}_{18}O_{62}]$** in 0.1 M MES buffer pH 5.5 that were recorded approximately one hour after preparation (**A**) and after incubation for 24 h at 37 °C (**B**). The chemical shifts and percentages of parent and formed species are given in **Tables S21** and **S22**. The structures of all POMs are shown in **Figure S98**. To identify the individual anions, they are shown in different colors, with the same color code being selected for a specific anion throughout all figures and tables in the main manuscript and the supporting information.

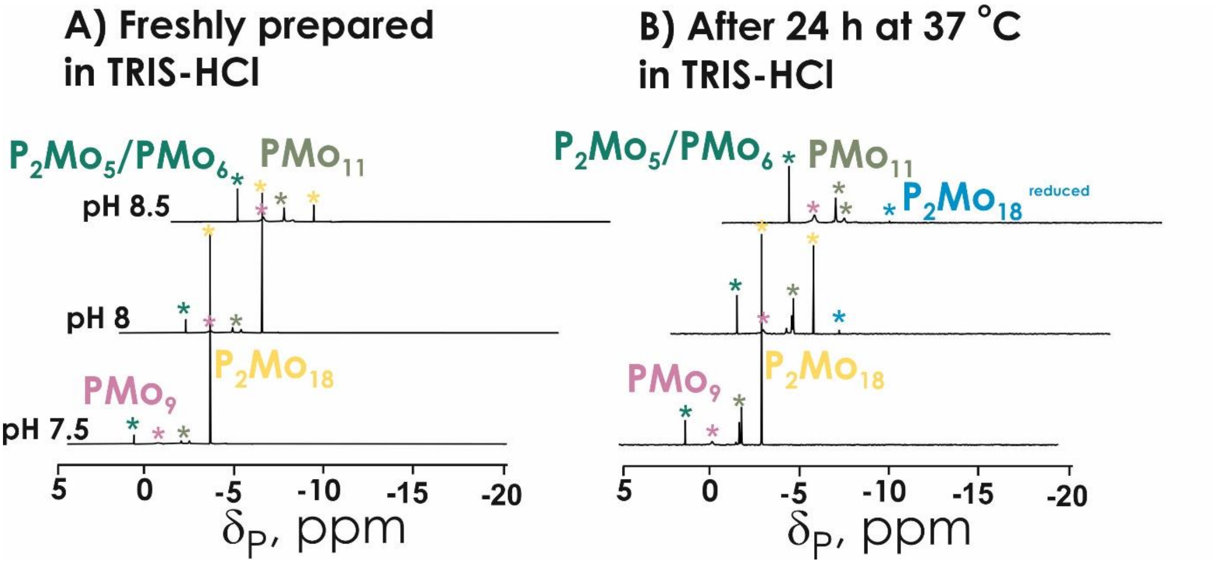

Fig. S105. ^{31}P NMR spectra of P_2Mo_{18} in tris-HCl buffer.

^{31}P NMR spectra for 10 mM solutions of **$(NH_4)_6[\alpha\text{-}P^V{}_2Mo^{VI}{}_{18}O_{62}]$** in 0.1 M tris-HCl buffer (pH 7.5 – 8.5) that were recorded approximately one hour after preparation (**A**) and after incubation for 24 h at 37 °C (**B**). The chemical shifts and percentages of parent and formed species are given in **Tables S21** and **S22**. The structures of all POMs are shown in **Figure S98**. To identify the individual anions, they are shown in different colors, with the same color code being selected for a specific anion throughout all figures and tables in the main manuscript and the supporting information.

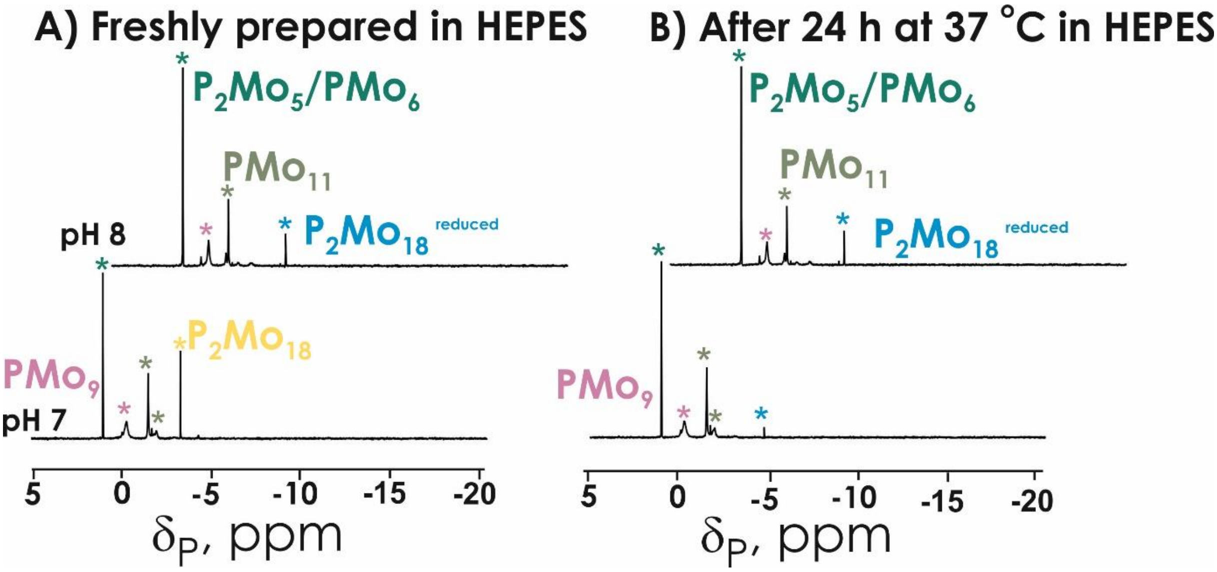

Fig. S106. ^{31}P NMR spectra of P_2Mo_{18} in HEPES buffer.

^{31}P NMR spectra for 10 mM solutions of **$(NH_4)_6[\alpha\text{-}P^V_2Mo^{VI}_{18}O_{62}]$** in 0.1 M HEPES buffer (pH 7 and 8) that were recorded approximately one hour after preparation (**A**) and after incubation for 24 h at 37 °C (**B**). The chemical shifts and percentages of parent and formed species are given in **Tables S21** and **S22**. The structures of all POMs are shown in **Figure S98**. To identify the individual anions, they are shown in different colors, with the same color code being selected for a specific anion throughout all figures and tables in the main manuscript and the supporting information.

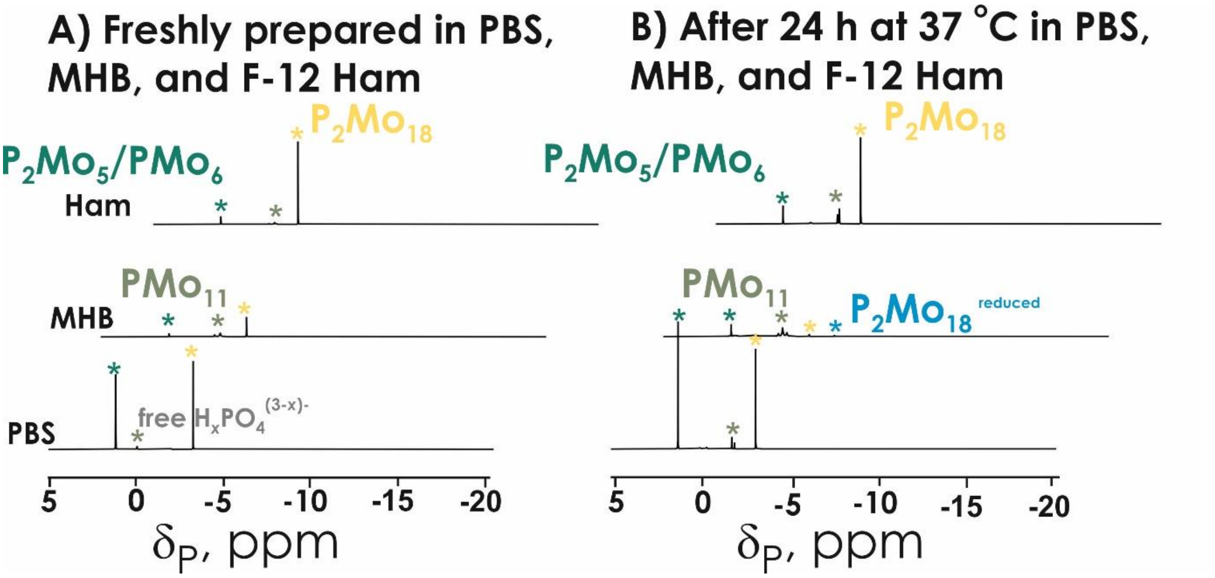

Fig. S107. ^{31}P NMR spectra of P_2Mo_{18} in solutions with pH 7.4.

^{31}P NMR spectra for 10 mM solutions of **$(NH_4)_6[\alpha\text{-}P^V_2Mo^{VI}_{18}O_{62}]$** in 0.1 M PBS, MHB and nutrient mixture F-12 Ham (pH 7.4) that were recorded approximately one hour after preparation (**A**) and after incubation for 24 h at 37 °C (**B**). The chemical shifts and percentages of parent and formed species are given in **Tables S21** and **S22**. The structures of all POMs are shown in **Figure S98**. To identify the individual anions, they are shown in different colors, with the same color code being selected for a specific anion throughout all figures and tables in the main manuscript and the supporting information.

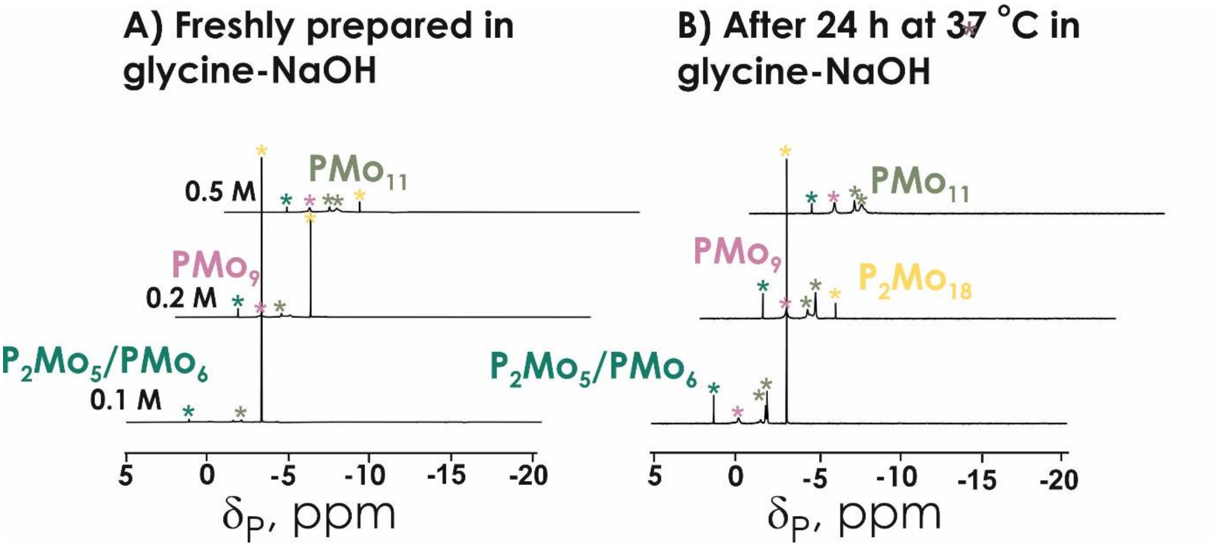

Fig. S108. ^{31}P NMR spectra of P_2Mo_{18} in glycine-NaOH buffer.

^{31}P NMR spectra for 10 mM solutions of **$(NH_4)_6[\alpha\text{-}P^{V}_2Mo^{VI}_{18}O_{62}]$** in glycine-NaOH (pH 8.6) with concentration 0.1. 0.2 and 0.5 M that were recorded approximately one hour after preparation (**A**) and after incubation for 24 h at 37 °C (**B**). The chemical shifts and percentages of parent and formed species are given in **Tables S21** and **S22**. The structures of all POMs are shown in **Figure S98**. To identify the individual anions, they are shown in different colors, with the same color code being selected for a specific anion throughout all figures and tables in the main manuscript and the supporting information.

Table S21. Analysis of NMR spectroscopic data recorded in P_2Mo_{18} solutions at room temperature.

Chemical shifts in ^{31}P NMR spectra measured in triplicate of **$(NH_4)_6[\alpha\text{-}P^{V}{}_2Mo^{VI}{}_{18}O_{62}]$** (10 mM) D_2O and 0.1 M buffers (acetic acid – sodium acetate pH 4 – 5.5; sodium phosphate pH 3 – 8 (while phosphate does not buffer at pH range from 3.5 – 5.5, experiments were conducted at this pH to provide comparisons to previously published studies (*29*)); citric acid – sodium citrate pH 3 – 6.5; MES pH 5.5; PBS pH 7.4; tris-HCl pH 7.5 – 8.5; HEPES pH 7 – 8; MHB pH 7.4; Nutrient mixture F-12 Ham pH 7.4 and glycine-NaOH pH 8.6) (**Figures S100** – **S108**). The glycine-NaOH buffer was used in two additional concentrations 0.2 and 0.5 M. The species content was calculated based on the integration of ^{31}P signals considering only signals associated with POTs. Signals were assigned based on the literature data summarized in **Table S4**.

pH	**$Na_6[P_2Mo_{18}O_{62}]$** (10 mM) in Solvent / Buffer / Medium	Chemical shifts δ ^{31}P [ppm]	% of P_2Mo_{18} in **$(NH_4)_6[\alpha\text{-}P^{V}{}_2Mo^{VI}{}_{18}O_{62}]$** (10 mM) at RT				% of PMo_{11} in **$(NH_4)_6[\alpha\text{-}P^{V}{}_2Mo^{VI}{}_{18}O_{62}]$** (10 mM) at RT				% of PMo_9 in **$(NH_4)_6[\alpha\text{-}P^{V}{}_2Mo^{VI}{}_{18}O_{62}]$** (10 mM) at RT				% of P_2Mo_5/PMo_6 in **$(NH_4)_6[\alpha\text{-}P^{V}{}_2Mo^{VI}{}_{18}O_{62}]$** (10 mM) at RT				Average % of other POMos present in solution
			Sample			Mean of 1 to 3 ± SD[a]	Sample			Mean of 1 to 3 ± SD[a]	Sample			Mean of 1 to 3 ± SD[a]	Sample			Mean of 1 to 3 ± SD[a]	
			#1	#2	#3		#1	#2	#3		#1	#2	#3		#1	#2	#3		
-	D_2O	–3.1	100	100	100	100 ± 0	0	0	0	0	0	0	0	0	0	0	0	0	0
Strongly acidic environment 3 ≤ pH ≤ 4																			
3	0.1 M Sodium phosphate ($H_2PO_4^-$ /H_3PO_4) pH 3	1.2; 0; –3.2	66	66	66	66 ± 0	0	0	0	0	0	0	0	0	34	34	34	34 ± 0	0
	0.1 M Citric acid – sodium citrate (H_3Cit/H_2Cit^-) pH 3	0; –3.2	100	100	100	100 ± 0	0	0	0	0	0	0	0	0	0	0	0	0	0
4	0.1 M Sodium phosphate ($H_2PO_4^-$ /H_3PO_4) pH 4	1.2; 0; –3.2	46	40	45	44 ± 3	0	0	0	0	0	0	0	0	54	60	55	56 ± 3	0
	0.1 M Citric acid – sodium citrate (H_2Cit^- /$HCit^{2-}$) pH 4	0; –3.2	100	100	100	100 ± 0	0	0	0	0	0	0	0	0	0	0	0	0	0
	0.1 M Acetic acid – sodium acetate (OAc^- /HOAc) pH 4	–3.1	100	100	100	100 ± 0	0	0	0	0	0	0	0	0	0	0	0	0	0
Moderately acidic environment 5 ≤ pH ≤ 6																			
6	0.1 M Sodium phosphate ($H_2PO_4^-$ /H_3PO_4) pH 5	1.2; 0; –3.2	35	35	36	35 ± 1	0	0	0	0	0	0	0	0	65	65	64	65 ± 1	0
	0.1 M Citric acid – sodium citrate (H_2Cit^- /$HCit^{2-}$) pH 5	1.2; 0; –3.2	0	0	0	0	0	0	0	0	0	0	0	0	100	100	100	100 ± 0	0

	0.1 M Acetic acid – sodium acetate ($OAc^{-}/HOAc$) pH 5	**1.2**; **–0.3**; **–1.4**; **–1.6**; **–2.0**; **–3.2**; –4.2	59	51	51	54 ± 5	19	25	21	22 ± 3	12	16	19	16 ± 3	7	7	8	7 ± 1	1
5.5	0.1 M Acetic acid – sodium acetate ($OAc^{-}/HOAc$) pH 5.5	**1.2**; **–0.3**; **–1.4**; **–1.6**; **–2.0**; **–3.2**; –4.2	31	31	25	29 ± 3	33	33	37	34 ± 2	24	24	23	24 ± 1	11	11	13	12 ± 1	1
	0.1 M MES[b] pH 5.5	**1.3**; **–0.1**; **–1.4**; **–1.5**; **–1.8**; **–3.1**	70	69	68	69 ± 1	23	24	24	24 ± 1	1	1	1	1 ± 0	6	6	7	6 ± 1	0
6.0	0.1 M Sodium phosphate ($HPO_4^{2-}/H_2PO_4^{-}$) pH 6	**1.2**; 0; **–3.2**	10	10	11	10 ± 1	0	0	0	0	0	0	0	0	90	90	89	90 ± 1	0
	0.1 M Citric acid – sodium citrate ($HCit^{2-}/Cit^{3-}$) pH 6	**1.2**; 0	0	0	0	0	0	0	0	0	0	0	0	0	100	100	100	100 ± 0	
Neutral environment 6.5 ≤ pH ≤ 7.5																			
6.5	0.1 M Citric acid – sodium citrate ($HCit^{2-}/Cit^{3-}$) pH 6.5	**1.2**; 0	0	0	0	0	0	0	0	0	0	0	0	0	100	100	100	100 ± 0	0
7	0.1 M Sodium phosphate ($HPO_4^{2-}/H_2PO_4^{-}$) pH 7	**1.2**; 0.4	0	0	0	0	0	0	0	0	0	0	0	0	100	100	100	100 ± 0	0
	0.1 M HEPES[c] pH 7	**1.1**; 0; **–0.2**; **–1.4**; **–1.6**; **–1.9**; **–3.2**	11	9	7	9 ± 2	44	41	45	43 ± 2	30	34	34	33 ± 2	14	16	14	15 ± 1	0
7.4	PBS[d] pH 7.4	**1.2**; 0; **–1.9**; **–3.1**	54	50	48	51 ± 3	0	2	2	1 ± 1	0	0	0	0	46	48	50	48 ± 2	0
	MHB[e] pH 7.4	**1.2**; 0; **–0.2**; **–1.4**; **–1.7**; **–3.2**	33	33	32	33 ± 1	51	48	55	51 ± 4	10	13	7	10 ± 3	6	6	6	6 ± 0	0
	Nutrient mixture F-12 Ham[f]	**1.2**; 0; **–0.2**; **–1.5**; **–1.8**; **–1.9**; **–3.1**	81	73	75	76 ± 4	10	13	14	12 ± 2	1	4	3	3 ± 1	8	10	8	9 ± 1	0
7.5	0.1 M tris-HCl[g] pH 7.5	**1.2**; **–0.3**; **–1.5**; **–2.0**; **–3.2**	73	77	71	74 ± 3	19	14	17	17 ± 3	4	6	7	6 ± 2	4	3	5	4 ± 1	0
Moderately alkaline environment 8 ≤ pH ≤ 8.6																			
8	0.1 M Sodium phosphate ($HPO_4^{2-}/H_2PO_4^{-}$) pH 8	**1.2**; 0.3	0	0	0	0	0	0	0	0	0	0	0	0	100	100	100	100 ± 0	0

	0.1 M HEPES pH 8	1.1; 0; –0.3; –1.3; –1.4; –4.6	0	0	0	0	25	25	24	25 ± 1	27	27	24	26 ± 2	46	46	50	47 ± 2	2
	0.1 M tris-HCl pH 8	1.2; –0.3; –1.5; –2.0; –3.2	52	57	50	53 ± 4	24	23	24	24 ± 1	17	15	18	17 ± 2	7	5	8	7 ± 2	0
8.5	0.1 M tris-HCl pH 8.5	1.2; –0.3; –1.3; –1.5; –2.0; –3.2; –4.1	6	6	5	6 ± 1	39	40	36	38 ± 2	39	38	40	39 ± 1	17	16	19	17 ± 2	0
	0.1 M glycine-NaOH pH 8.6	1.2; 0; –0.2; –1.5; –2.0; –3.2; –4.1	78	87	79	81 ± 5	15	9	15	13 ± 3	5	2	4	4 ± 2	2	2	2	2 ± 0	0
8.6	0.2 M glycine-NaOH pH 8.6	1.2; –0.2; –1.4; –1.9; –3.2; –4.1	35	42	42	40 ± 4	37	35	30	34 ± 4	22	20	21	21 ± 1	5	4	6	5 ± 1	0
	0.5 M glycine-NaOH pH 8.6	1.2; –0.2; –1.4; –1.8; –3.2	0	0	0	0	71	73	71	72 ± 1	24	23	24	24 ± 1	5	4	5	5 ± 1	0

[a]SD – standard deviation; [b]MES – 2-(N-morpholino)ethanesulfonic acid, $C_6H_{13}NO_4S$ (Figure S1); [c]HEPES – 4-(2-hydroxyethyl)-1-piperazineethanesulfonic acid, $C_8H_{18}N_2O_4S$ (Figure S1); [d]PBS – phosphate buffer saline; [e]MHB – Mueller-Hinton broth, for more detailed information about composition, see https://labmal.com/2019/11/20/mueller-hinton-agar-and-mueller-hinton-broth/; [f]Nutrient mixture F-12 Ham contains sodium pyruvate (0.11 g/L), phenol red, L-glutamine, and does not contain $NaHCO_3$ and HEPES, for more details please see https://www.sigmaaldrich.com/AT/en/technical-documents/technical-article/cell-culture-and-cell-culture-analysis/mammalian-cell-culture/f-12-ham; [g]tris – tris(hydroxymethyl)aminomethane, $C_4H_{11}NO_3$ (Figure S1).

Table S22. Analysis of NMR spectroscopic data of P_2Mo_{18} solutions investigated after 24 h incubation at 37 °C.

Chemical shifts in ^{31}P NMR spectra measured in triplicate of **$(NH_4)_6[\alpha\text{-}P^{V}{}_2Mo^{VI}{}_{18}O_{62}]$** (10 mM) dissolved in D_2O and 0.1 M buffers (acetic acid – sodium acetate pH 4 – 5.5; sodium phosphate pH 3 – 8 (while phosphate does not buffer at pH range from 3.5 – 5.5, experiments were conducted at this pH to provide comparisons to previously published studies (*29*)); citric acid – sodium citrate pH 3 – 6.5; MES pH 5.5; PBS pH 7.4; tris-HCl pH 7.5 – 8.5; HEPES pH 7 – 8; MHB pH 7.4; Nutrient mixture F-12 Ham pH 7.4 and glycine-NaOH pH 8.6) and investigated after 24 h incubation at 37 °C (**Figures S100** – **S108**). The glycine-NaOH buffer was used in two additional concentrations 0.2 and 0.5 M. The species content was calculated based on the integration of ^{31}P signals considering only signals associated with POTs. Signals were assigned based on the literature data summarized in **Table S4**.

**pH**	**$Na_6[P_2Mo_{18}O_{62}]$** (10 mM) in Solvent / Buffer / Medium	Chemical shifts δ ^{31}P [ppm]	% of P_2Mo_{18} in **$(NH_4)_6[\alpha\text{-}P^{V}{}_2Mo^{VI}{}_{18}O_{62}]$** (10 mM) after 24 h incubation at 37 °C				% of PMo_{11} in **$(NH_4)_6[\alpha\text{-}P^{V}{}_2Mo^{VI}{}_{18}O_{62}]$** (10 mM) after 24 h incubation at 37 °C				% of PMo_9 in **$(NH_4)_6[\alpha\text{-}P^{V}{}_2Mo^{VI}{}_{18}O_{62}]$** (10 mM) after 24 h incubation at 37 °C				% of P_2Mo_5/PMo_6 in **$(NH_4)_6[\alpha\text{-}P^{V}{}_2Mo^{VI}{}_{18}O_{62}]$** (10 mM) after 24 h incubation at 37 °C				Average % of other POMos present in solution
			Sample			Mean of 1 to 3 ± SD[a]	Sample			Mean of 1 to 3 ± SD[a]	Sample			Mean of 1 to 3 ± SD[a]	Sample			Mean of 1 to 3 ± SD[a]	
			#1	#2	#3		#1	#2	#3		#1	#2	#3		#1	#2	#3		
-	D_2O	–1.8; –2.0; –3.1	98	98	99	98 ± 1	2	2	1	2 ± 1	0	0	0	0	0	0	0	0	0
Strongly acidic environment 3 ≤ pH ≤ 4																			
3	0.1 M Sodium phosphate ($H_2PO_4^-$/H_3PO_4) pH 3	1.2; 0; –3.2	15	13	11	13 ± 2	0	0	0	0	0	0	0	0	85	87	89	87 ± 2	0
	0.1 M Citric acid – sodium citrate (H_3Cit/H_2Cit^-) pH 3	–0.2; –3.2	100	100	100	100 ± 0	0	0	0	0	0	0	0	0	0	0	0	0	0
4	0.1 M Sodium phosphate ($H_2PO_4^-$/H_3PO_4) pH 4	1.2; 0; –3.2	9	7	8	8 ± 1	0	0	0	0	0	0	0	0	91	93	92	92 ± 1	0
	0.1 M Citric acid – sodium citrate ($H_2Cit^-/HCit^{2-}$) pH 4	0; –3.2	99	100	100	100 ± 1	0	0	0	0	0	0	0	0	1	0	0	1 ± 1	0
	0.1 M Acetic acid – sodium acetate ($OAc^-/HOAc$) pH 4	1.2; –0.3; –1.7; –1.9; –2.0; –3.2	59	58	55	57 ± 2	31	32	33	32 ± 1	5	5	6	5 ± 1	5	5	6	5 ± 1	1
Moderately acidic environment 5 ≤ pH ≤ 6																			
5	0.1 M Sodium phosphate ($H_2PO_4^-$/H_3PO_4) pH 5	1.2; 0; –3.2	6	7	5	6 ± 1	0	0	0	0	0	0	0	0	94	93	95	94 ± 1	0
	0.1 M Citric acid – sodium citrate ($H_2Cit^-/HCit^{2-}$) pH 5	1.2; 0	0	0	0	0	0	0	0	0	0	0	0	0	100	100	100	100 ± 0	0

	0.1 M Acetic acid – sodium acetate ($OAc^{–}/HOAc$) pH 5	1.2; –0.2; –1.5; –1.8; –2.0	0	0	0	0	59	61	60	60 ± 1	28	27	28	28 ± 1	13	12	12	12 ± 1	0
5.5	0.1 M Acetic acid – sodium acetate ($OAc^{–}/HOAc$) pH 5.5	1.2; –0.2; –1.5; –1.7; –2.0	0	0	0	0	56	56	55	56 ± 1	31	31	32	31 ± 1	13	13	13	13 ± 0	0
	0.1 M MES[b] pH 5.5	1.3; –0.2; –1.6; –1.7; –3.1	39	37	40	39 ± 2	44	46	43	44 ± 2	7	5	6	6 ± 1	10	12	11	11 ± 1	0
6	0.1 M Sodium phosphate ($HPO_4^{2–}/H_2PO_4^{–}$) pH 6	1.2; 0; –3.2	1	2	2	2 ± 1	0	0	0	0	0	0	0	0	99	98	98	98 ± 1	0
	0.1 M Citric acid – sodium citrate ($HCit^{2–}/Cit^{3–}$) pH 6	1.2; 0.1	0	0	0	0	0	0	0	0	0	0	0	0	100	100	100	100 ± 0	0
Neutral environment 6.5 ≤ pH ≤ 7.5																			
6.5	0.1 M Citric acid – sodium citrate ($HCit^{2–}/Cit^{3–}$) pH 6.5	1.2; 0.2	0	0	0	0	0	0	0	0	0	0	0	0	100	100	100	100 ± 0	0
7	0.1 M Sodium phosphate ($HPO_4^{2–}/H_2PO_4^{–}$) pH 7	1.2; 0	0	0	0	0	0	0	0	0	0	0	0	0	100	100	100	100 ± 0	0
	0.1 M HEPES[c] pH 7	1.1; 0; –0.2; –1.4; –1.6; –1.9; –4.6	0	0	0	0	52	50	52	51 ± 1	33	34	33	33 ± 1	14	15	14	14 ± 1	2
7.4	PBS[d] pH 7.4	1.2; 0; –0.4; –1.8; –2.0; –3.2	32	36	34	34 ± 2	9	3	9	7 ± 3	3	3	2	3 ± 1	56	51	55	54 ± 3	2
	MHB[e] pH 7.4	1.2; 0; –0.2; –1.5; –1.7; –2.0; –3.2; –4.6	3	3	1	2 ± 1	66	67	72	68 ± 3	17	19	16	17 ± 2	13	10	10	11 ± 2	2
	Nutrient mixture F-12 Ham[f]	1.2; –0.3; –1.7; –1.9; –3.2	54	50	48	51 ± 3	30	35	36	34 ± 3	6	5	7	6 ± 1	10	10	9	10 ± 1	0
7.5	0.1 M tris-HCl[g] pH 7.5	1.2; –0.2; –1.7; –1.9; –2.0; –3.2	45	48	42	45 ± 3	39	37	41	39 ± 2	9	8	9	9 ± 1	7	7	8	7 ± 1	0
Moderately alkaline environment 8 ≤ pH ≤ 8.6																			

8	0.1 M Sodium phosphate (HPO_4^{2-}/$H_2PO_4^-$) pH 8	**1.2**; 0.3	0	0	0	0	0	0	0	0	0	0	0	0	100	100	100	100 ± 0	0
	0.1 M HEPES pH 8	**1.1**; 0; **–0.2**; **–1.3**; **–1.4**; –4.6	0	0	0	0	25	25	23	24 ± 1	25	26	25	25 ± 1	48	47	49	48 ± 1	3
	0.1 M tris-HCl pH 8	**1.2**; **–0.3**; **–1.6**; **–1.9**; **–2.0**; **–3.2**; –4.6	18	22	14	18 ± 4	53	52	55	53 ± 2	16	15	18	16 ± 2	12	10	12	11 ± 1	2
8.5	0.1 M tris-HCl pH 8.5	**1.2**; **–0.3**; **–1.3**; **–1.5**; **–2.0**; –4.6	0	0	0	0	44	47	40	44 ± 2	40	38	40	40 ± 1	16	15	20	16 ± 3	0
8.6	0.1 M glycine-NaOH pH 8.6	**1.2**; **–0.3**; **–1.4**; **–1.9**; **–2.0**; **–3.2**	40	42	35	39 ± 4	41	40	46	42 ± 3	13	12	13	13 ± 1	6	6	6	6 ± 0	0
	0.2 M glycine-NaOH pH 8.6	**1.2–0.2**; **–1.5**; **–2.0**; **–3.2**	2	2	0	1 ± 1	68	68	68	68 ± 0	25	25	27	26 ± 1	5	5	5	5 ± 0	0
	0.5 M glycine-NaOH pH 8.6	**1.2**; **–0.2**; **–1.4**; **–1.8**	0	0	0	0	70	73	70	71 ± 2	25	24	24	24 ± 1	5	3	6	5 ± 2	0

[a]SD – standard deviation; [b]MES – 2-(N-morpholino)ethanesulfonic acid, $C_6H_{13}NO_4S$ (Figure S1); [c]HEPES – 4-(2-hydroxyethyl)-1-piperazineethanesulfonic acid, $C_8H_{18}N_2O_4S$ (Figure S1); [d]PBS – phosphate buffer saline; [e]MHB – Mueller-Hinton broth, for more detailed information about composition, see https://labmal.com/2019/11/20/mueller-hinton-agar-and-mueller-hinton-broth/; [f]Nutrient mixture F-12 Ham contains sodium pyruvate (0.11 g/L), phenol red, L-glutamine, and does not contain $NaHCO_3$ and HEPES, for more details please see https://www.sigmaaldrich.com/AT/en/technical-documents/technical-article/cell-culture-and-cell-culture-analysis/mammalian-cell-culture/f-12-ham; [g]tris – tris(hydroxymethyl)aminomethane, $C_4H_{11}NO_3$ (Figure S1).

10.3. Speciation in P_2Mo_{18} Wells-Dawson POMo solutions

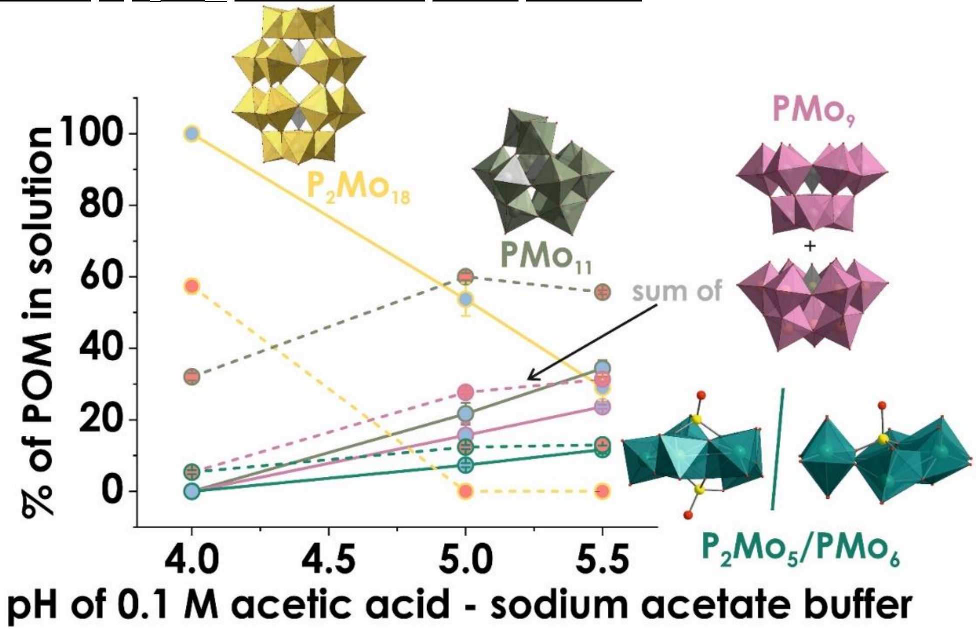

Fig. S109. Speciation of P_2Mo_{18} in acetic acid – sodium acetate buffer.

POM concentration curves in **$(NH_4)_6[\alpha\text{-}P^V{}_2Mo^{VI}{}_{18}O_{62}]$** (10 mM) in 0.1 M acetic acid – sodium acetate buffer solutions before (solid line, blue dot in the middle) and after incubation (dash line, red dot in the middle) for 24 h at 37 °C. The exact percentage of all POM species present is given in **Tables S21** and **S22**.

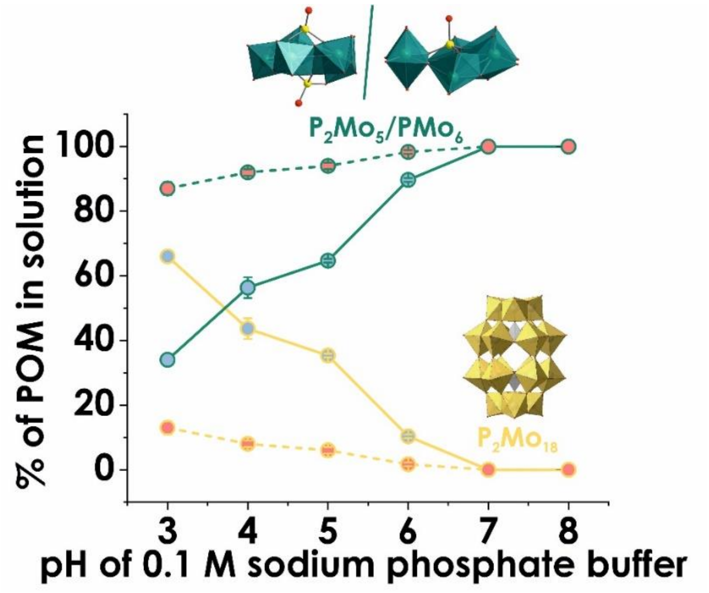

Fig. S110. Speciation of P_2Mo_{18} in sodium phosphate buffer.

POM concentration curves in **$(NH_4)_6[\alpha\text{-}P^V{}_2Mo^{VI}{}_{18}O_{62}]$** (10 mM) in 0.1 M sodium phosphate buffer solutions before (solid line, blue dot in the middle) and after incubation (dash line, red dot in the middle) for 24 h at 37 °C. The exact percentage of all POM species present is given in **Tables S21** and **S22**.

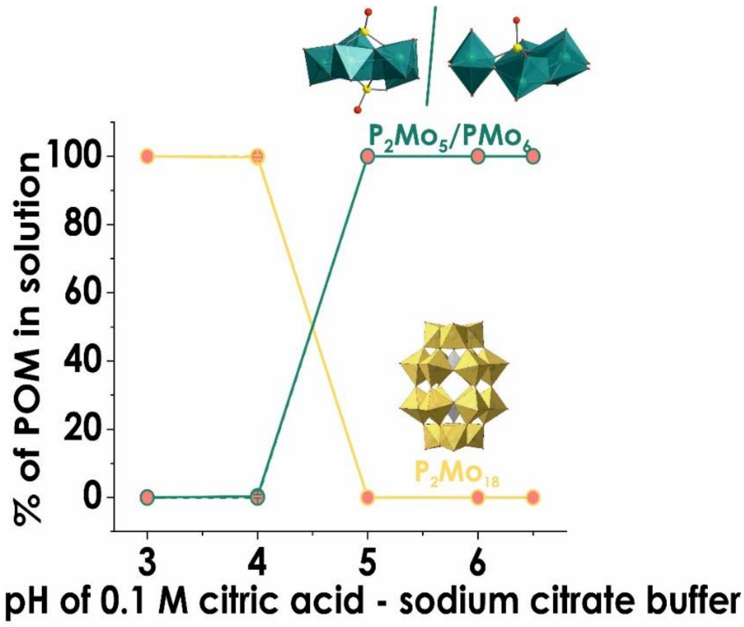

Fig. S111. Speciation of P_2Mo_{18} in citric acid – sodium citrate buffer.

POM concentration curves of **$(NH_4)_6[\alpha\text{-}P^V{}_2Mo^{VI}{}_{18}O_{62}]$** (10 mM) in 0.1 M citric acid – sodium citrate buffer solutions before (solid line, blue dot in the middle) and after incubation (dash line, red dot in the middle) for 24 h at 37 °C. The exact percentage of all POM species present is given in **Tables S21** and **S22**.

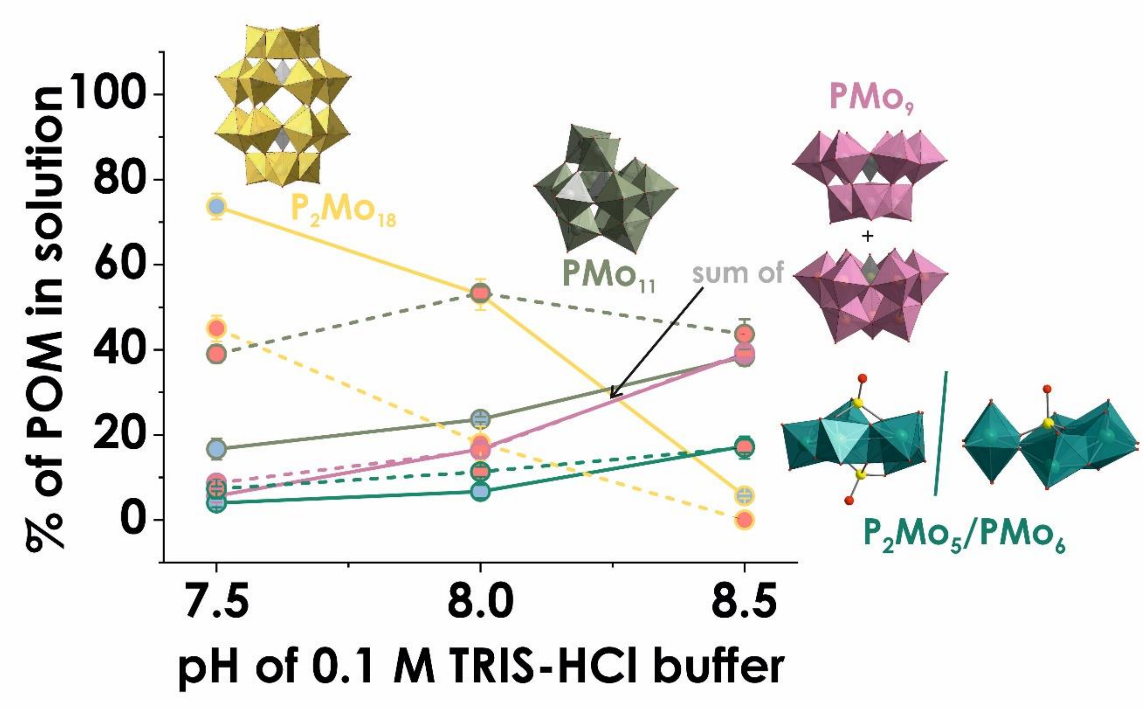

Fig. S112. Speciation of P_2Mo_{18} in tris-HCl buffer.

POM concentration curves of **$(NH_4)_6[\alpha\text{-}P^V{}_2Mo^{VI}{}_{18}O_{62}]$** (10 mM) in 0.1 M tris-HCl buffer solutions before (solid line, blue dot in the middle) and after incubation (dash line, red dot in the middle) for 24 h at 37 °C. The exact percentage of all POM species present is given in **Tables S21** and **S22**.

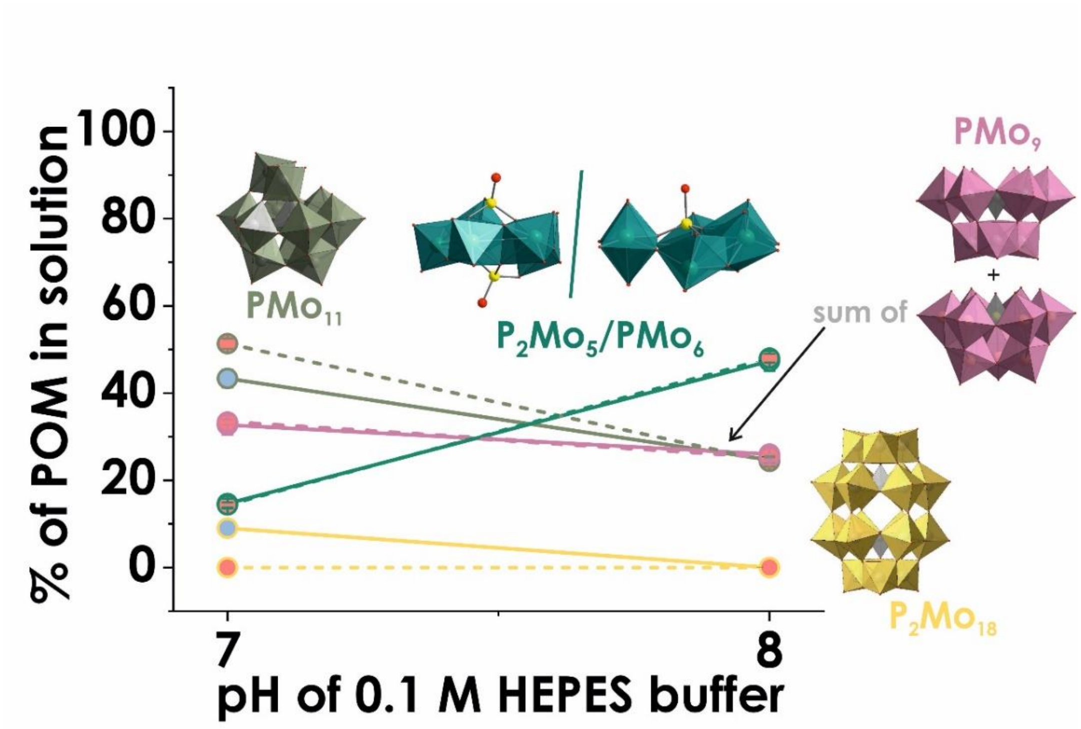

Fig. S113. Speciation of P_2Mo_{18} in HEPES buffer.

POM concentration curves of **$(NH_4)_6[\alpha\text{-}P^{V}_2Mo^{VI}_{18}O_{62}]$** (10 mM) in 0.1 M HEPES buffer solutions before (solid line, blue dot in the middle) and after incubation (dash line, red dot in the middle) for 24 h at 37 °C. The exact percentage of all POM species present is given in **Tables S21** and **S22**.

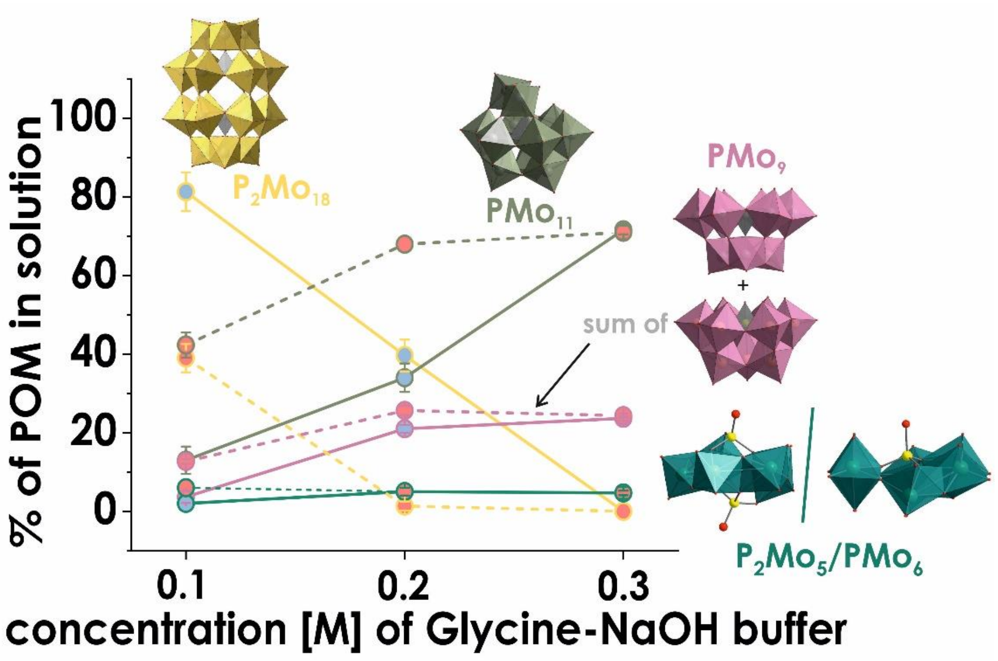

Fig. S114. Speciation of P_2Mo_{18} in glycine-NaOH buffer.

POM concentration curves of **$(NH_4)_6[\alpha\text{-}P^{V}_2Mo^{VI}_{18}O_{62}]$** (10 mM) in glycine-NaOH (pH 8.6) with concentrations of 0.1. 0.2 and 0.5 M solutions before (solid line, blue dot in the middle) and after incubation (dash line, red dot in the middle) for 24 h at 37 °C. The exact percentage of all POM species present is given in **Tables S21** and **S22**.

11. The $(ZrPW_{11})_2$ sandwich Keggin POT

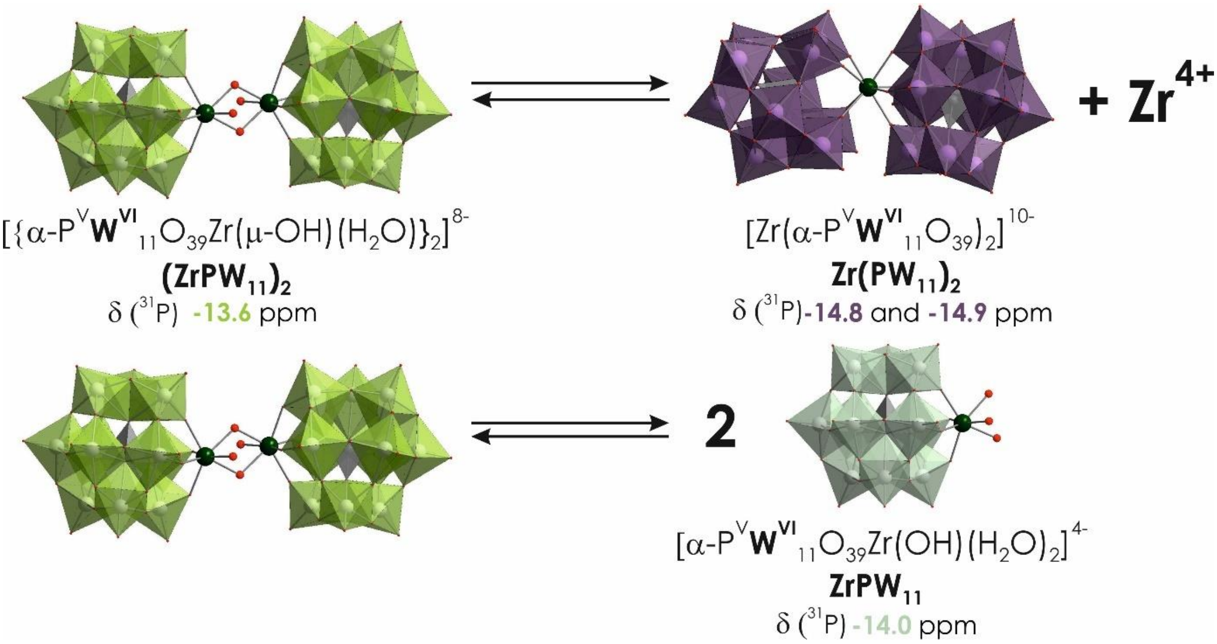

Fig. S115. Hydrolysis of $(ZrPW_{11})_2$.

Equilibrium reactions between a 2:2 Zr-sandwich $[\{\alpha\text{-}P^{V}W^{VI}_{11}O_{39}Zr^{IV}(\mu\text{-}OH)(H_2O)\}_2]^{8-}$ (**$(ZrPW_{11})_2$**) and a 1:2 Zr-sandwich $[Zr^{IV}(\alpha\text{-}P^{V}W^{VI}_{11}O_{39})_2]^{10-}$ (**$(ZrPW_{11})_2$**) or a monomer $[\alpha\text{-}P^{V}W^{VI}_{11}O_{39}Zr^{IV}(OH)(H_2O)_2]^{4-}$ (**$ZrPW_{11}$**). Color code: $\{WO_6\}$, grassy green, purple or sage green; $\{PO_4\}$, gray; Zr, blue; O, red. To identify the individual anions, they are shown in different colors, with the same color code being selected for a specific anion throughout all figures and tables in the main manuscript and the supporting information.

11.1. pH of $(ZrPW_{11})_2$ POT solutions

Table S23. pH in $(ZrPW_{11})_2$ solutions.

pH values measured in triplicate of $(Et_2NH_2)_8[\{\alpha\text{-}P^{V}W^{VI}_{11}O_{39}Zr^{IV}(\mu\text{-}OH)(H_2O)\}_2]\cdot 7H_2O$ (in this section both neutral salt and the anion have the abbreviation **$(ZrPW_{11})_2$**) (10 mM) dissolved in D_2O and 0.1 M buffers (acetic acid – sodium acetate pH 4 – 5.5; sodium phosphate pH 3 – 8 (while phosphate does not buffer at pH range from 3.5 – 5.5, experiments were conducted at this pH to provide comparisons to previously published studies (*29*)); citric acid – sodium citrate pH 3 – 6.5; MES pH 5.5; PBS pH 7.4; tris-HCl pH 7.5 – 8.5; HEPES pH 7 – 8; MHB pH 7.4; Nutrient mixture F-12 Ham pH 7.4 and glycine-NaOH pH 8.6) with concentration 0.1 M. The glycine-NaOH buffer was used in two additional concentrations 0.2 and 0.5 M.

**pH**	**(ZrPW11)₂** (10 mM) in Solvent / Buffer / Medium	pH after dissolving **$(ZrPW_{11})_2$** (10 mM) at RT				pH after 24 h incubation of **$(ZrPW_{11})_2$** (10 mM) solution at 37 °C			
		Sample			Mean of 1 to 3 ± SD[a]	Sample			Mean of 1 to 3 ± SD[a]
		#1	#2	#3		#1	#2	#3	
-	D_2O	4.69	4.71	4.79	4.73 ± 0.05	4.52	4.62	4.62	4.59 ± 0.06
Strongly acidic environment 3 ≤ pH ≤ 4									
3	0.1 M Sodium phosphate ($H_2PO_4^-$/H_3PO_4) pH 3	3.33	3.36	3.24	3.31 ± 0.06	3.31	3.40	3.23	3.31 ± 0.09
	0.1 M Citric acid – sodium citrate (H_3Cit/H_2Cit^-) pH 3	2.60	2.57	2.59	2.59 ± 0.02	2.59	2.54	2.64	2.59 ± 0.05
4	0.1 M Sodium phosphate ($H_2PO_4^-$/H_3PO_4) pH 4	4.29	4.34	4.34	4.32 ± 0.03	4.18	4.27	4.29	4.25 ± 0.06
	0.1 M Citric acid – sodium citrate (H_2Cit^-/$HCit^{2-}$) pH 4	3.73	3.73	3.76	3.74 ± 0.02	3.72	3.72	3.77	3.74 ± 0.03
	0.1 M Acetic acid – sodium acetate (OAc^-/HOAc) pH 4	3.95	3.94	3.94	3.94 ± 0.01	3.91	3.94	3.91	3.92 ± 0.02
Moderately acidic environment 5 ≤ pH ≤ 6									
5	0.1 M Sodium phosphate ($H_2PO_4^-$/H_3PO_4) pH 5	4.69	4.7	4.72	4.70 ± 0.02	4.54	4.55	4.62	4.57 ± 0.04
	0.1 M Citric acid – sodium citrate (H_2Cit^-/$HCit^{2-}$) pH 5	4.88	4.91	4.90	4.90 ± 0.02	4.89	4.91	4.90	4.90 ± 0.01
	0.1 M Acetic acid – sodium acetate (OAc^-/HOAc) pH 5	4.94	4.93	4.94	4.94 ± 0.01	4.87	4.89	4.88	4.88 ± 0.01

5.5	0.1 M Acetic acid – sodium acetate ($OAc^{–}/HOAc$) pH 5.5	5.44	5.45	5.47	5.45 ± 0.02	5.28	5.32	5.33	5.31 ± 0.03
	0.1 M MES[b] pH 5.5	5.27	5.24	5.25	5.25 ± 0.02	4.88	4.95	4.92	4.92 ± 0.04
6	0.1 M Sodium phosphate ($HPO_4^{2–}/H_2PO_4^{–}$) pH 6	5.86	5.85	5.89	5.87 ± 0.02	5.76	5.71	5.76	5.74 ± 0.02
	0.1 M Citric acid – sodium citrate ($HCit^{2–}/Cit^{3–}$) pH 6	6.00	6.02	5.99	6.00 ± 0.02	6.08	6.11	6.05	6.08 ± 0.03
Neutral environment 6.5 ≤ pH ≤ 7.5									
6.5	0.1 M Citric acid – sodium citrate ($HCit^{2–}/Cit^{3–}$) pH 6.5	6.59	6.66	6.62	5.62 ± 0.04	6.96	6.99	6.94	6.96 ± 0.03
7	0.1 M Sodium phosphate ($HPO_4^{2–}/H_2PO_4^{–}$) pH 7	6.93	6.9	7.03	6.95 ± 0.07	6.87	6.88	6.89	6.88 ± 0.01
	0.1 M HEPES[c] pH 7	7.07	7.04	7.10	7.07 ± 0.03	6.76	6.78	6.83	6.79 ± 0.04
7.4	PBS[d] pH 7.4	6.11	6.28	6.41	6.27 ± 0.07	5.76	6.05	6.13	5.98 ± 0.19
	MHB[e] pH 7.4	7.00	6.94	7.07	7.00 ± 0.07	6.58	6.58	6.65	6.60 ± 0.04
	Nutrient mixture F-12 Ham[f]	6.14	6.2	6.53	6.29 ± 0.21	5.87	5.97	6.42	6.09 ± 0.29
7.5	0.1 M tris-HCl[g] pH 7.5	6.33	6.25	6.35	6.31 ± 0.05	6.05	6.07	6.11	6.08 ± 0.03
Moderately alkaline environment 8 ≤ pH ≤ 8.6									
8	0.1 M Sodium phosphate ($HPO_4^{2–}/H_2PO_4^{–}$) pH 8	7.22	7.19	7.25	7.22 ± 0.03	7.18	7.24	7.18	7.20 ± 0.03
	0.1 M HEPES pH 8	7.59	7.55	7.65	7.60 ± 0.05	7.17	7.21	7.19	7.19 ± 0.03
	0.1 M tris-HCl pH 8	6.74	6.60	6.80	6.71 ± 0.10	6.58	6.54	6.60	6.57 ± 0.03
8.5	0.1 M tris-HCl pH 8.5	7.35	7.33	7.39	7.35 ± 0.03	7.07	7.12	7.07	7.09 ± 0.03
8.6	0.1 M glycine-NaOH pH 8.6	6.41	6.49	6.58	6.49 ± 0.09	5.80	5.98	6.14	5.97 ± 0.17
	0.2 M glycine-NaOH pH 8.6	6.81	6.90	7.06	6.92 ± 0.13	6.68	6.77	7.02	6.82 ± 0.18
	0.5 M glycine-NaOH pH 8.6	7.23	7.25	7.39	7.29 ± 0.09	6.94	6.96	7.03	6.98 ± 0.05

[a]SD – standard deviation; [b]MES – 2-(N-morpholino)ethanesulfonic acid, $C_6H_{13}NO_4S$ (Figure S1); [c]HEPES – 4-(2-hydroxyethyl)-1-piperazineethanesulfonic acid, $C_8H_{18}N_2O_4S$ (Figure S1); [d]PBS – phosphate buffer saline; [e]MHB – Mueller-Hinton broth, for more detailed information about composition, see https://labmal.com/2019/11/20/mueller-hinton-agar-and-mueller-hinton-broth/; [f]Nutrient mixture F-12 Ham contains sodium pyruvate (0.11 g/L), phenol red, L-glutamine, and does not contain $NaHCO_3$ and HEPES, for more details please see https://www.sigmaaldrich.com/AT/en/technical-documents/technical-article/cell-culture-and-cell-culture-analysis/mammalian-cell-culture/f-12-ham; [g]tris – tris(hydroxymethyl)aminomethane, $C_4H_{11}NO_3$ (Figure S1).

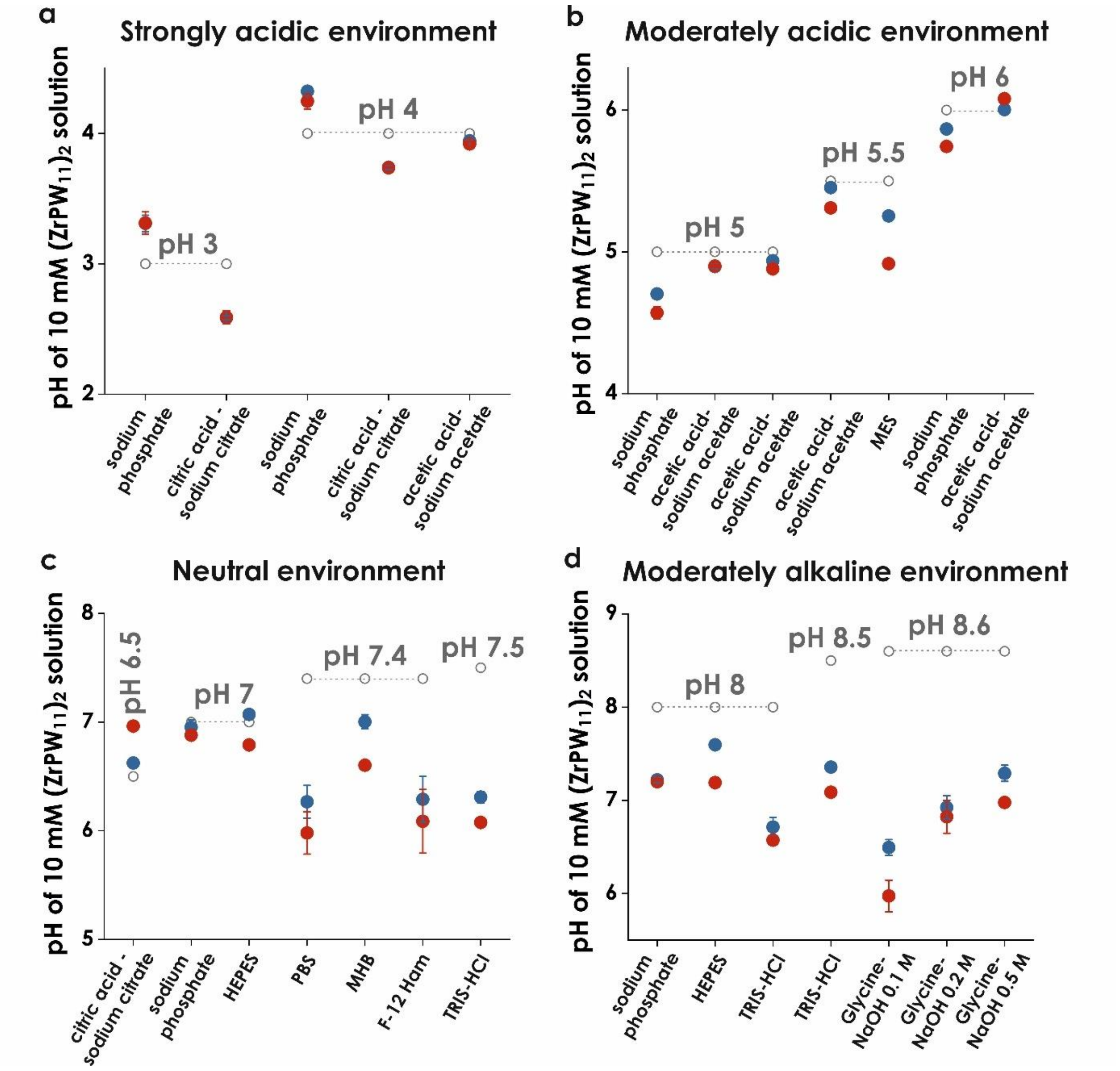

Fig. S116. pH in $(ZrPW_{11})_2$ solutions.

The pH dependence curves for 10 mM solutions of **$(ZrPW_{11})_2$** in different buffers in a) strongly acidic, b) moderately acidic, c) neutral, and d) moderately alkaline environments. The pH of the starting buffers is shown with a gray dashed line; the plots of the measured pH values immediately after the preparation of the solutions are shown in blue and after 24 h incubation at 37 °C in red. The error bar shows standard deviation (**Table S23**).

11.2. ^{31}P NMR spectroscopic studies of $(ZrPW_{11})_2$ POT solutions

All peaks with the highest intensity were unambiguously assigned based on the literature data from **Table S4**. In some spectra, chemical shifts for peaks of lower intensity have not yet been described in the literature and therefore can not be assigned in this work.

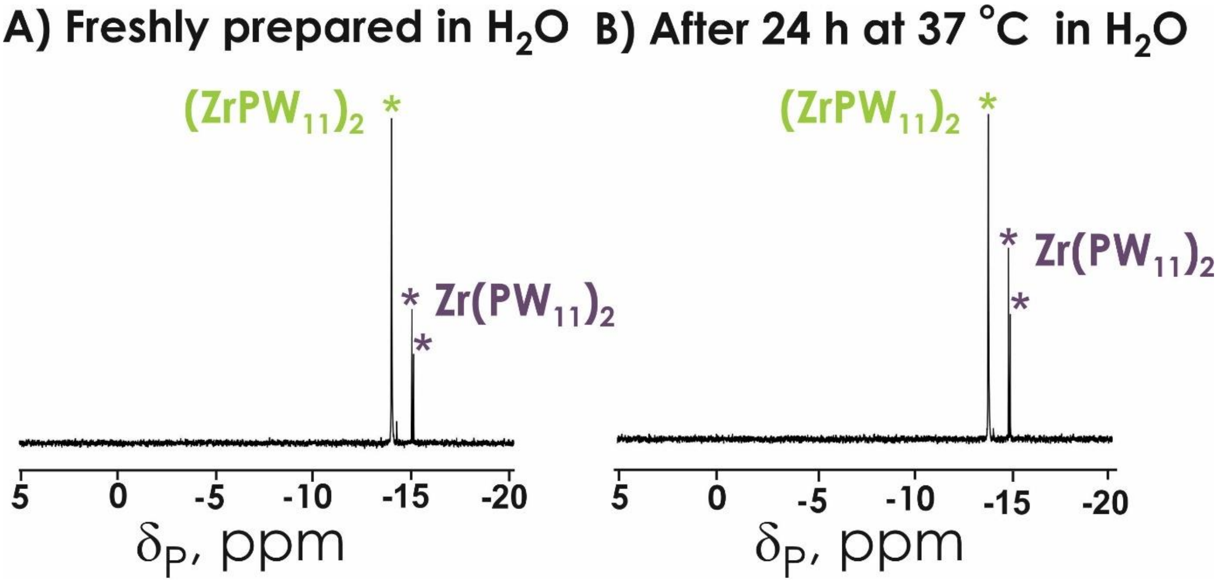

Fig. S117. ^{31}P NMR spectra of $(ZrPW_{11})_2$ in H_2O.

^{31}P NMR spectra for 10 mM solutions of $\mathbf{(ZrPW_{11})_2}$ in D_2O that were recorded approximately one hour after preparation (**A**) and after incubation for 24 h at 37 °C (**B**). The chemical shifts and percentages of parent and formed species are given in **Tables S24** and **S25**. The structures of all POMs are shown in **Figure S115**. To identify the individual anions, they are shown in different colors, with the same color code being selected for a specific anion throughout all figures and tables in the main manuscript and the supporting information.

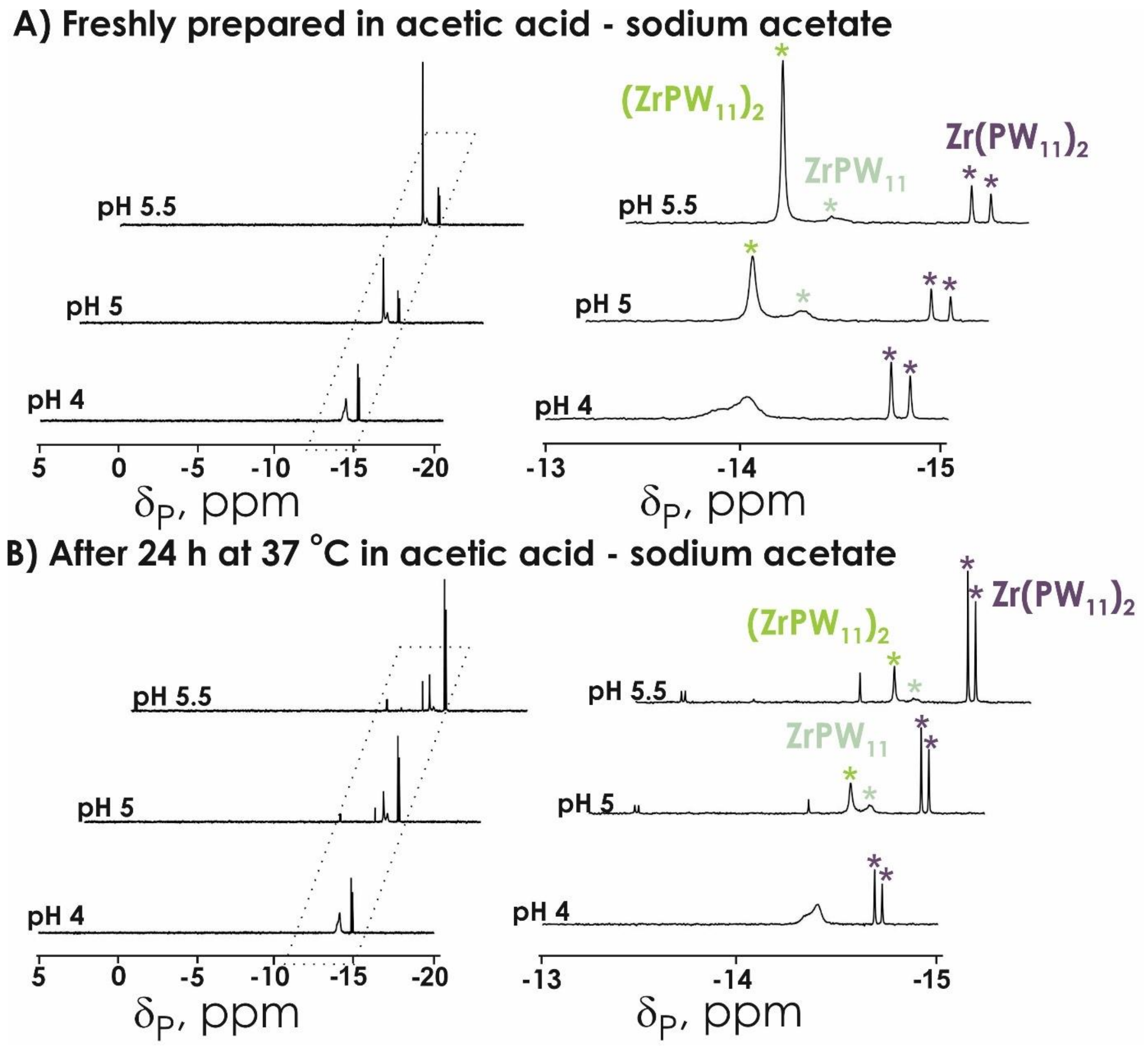

Fig. S118. ^{31}P NMR spectra of (ZrPW$_{11}$)$_2$ in NaOAc/HOAc buffer.

^{31}P NMR spectra for 10 mM solutions of **(ZrPW$_{11}$)$_2$** in 0.1 M NaOAc/HOAc buffer (pH 4 – 5.5) that were recorded approximately one hour after preparation (**A**) and after incubation for 24 h at 37 °C (**B**). The chemical shifts and percentages of parent and formed species are given in **Tables S24** and **S25**. The structures of all POMs are shown in **Figure S115**. To identify the individual anions, they are shown in different colors, with the same color code being selected for a specific anion throughout all figures and tables in the main manuscript and the supporting information.

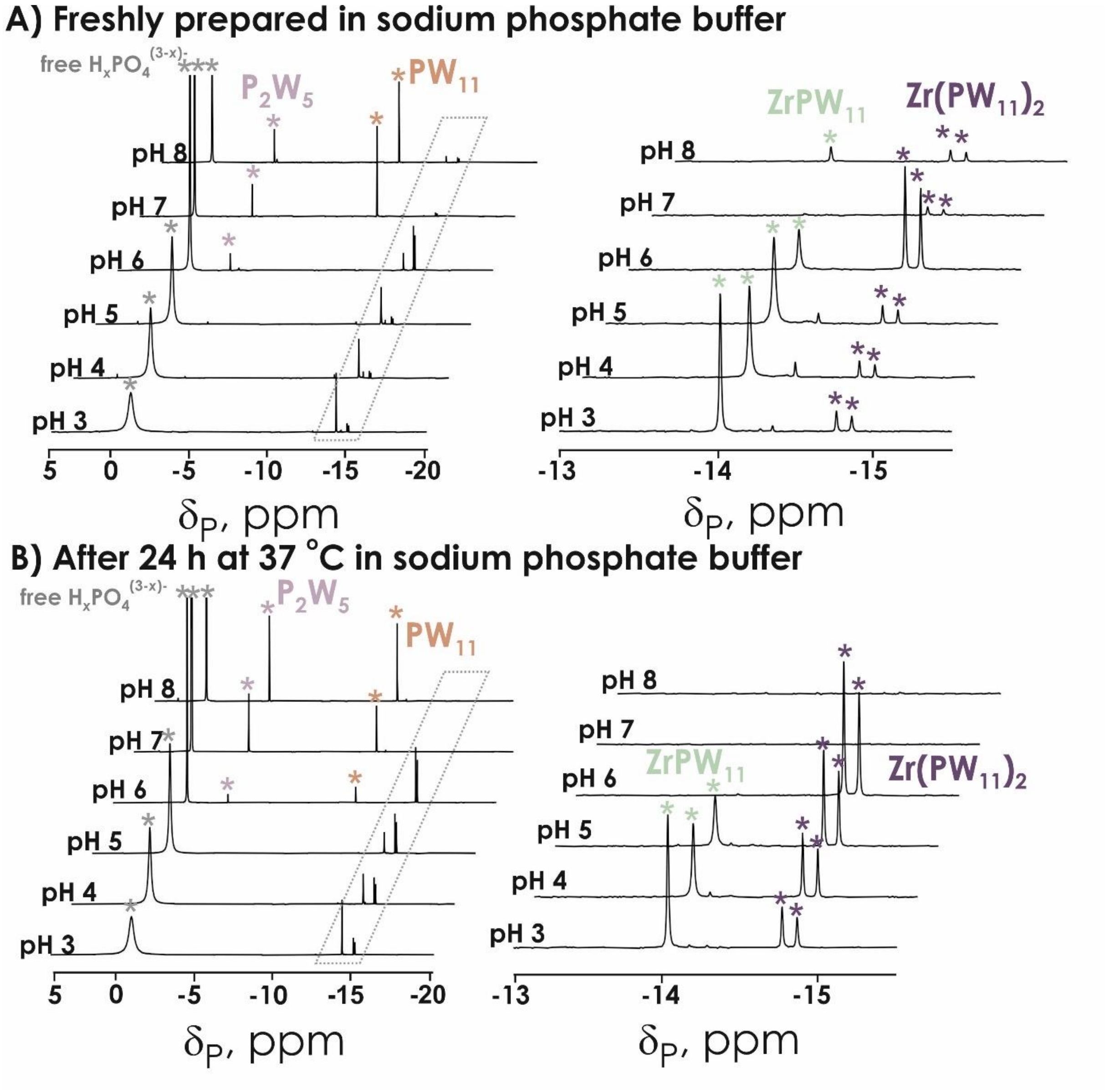

Fig. S119. ^{31}P NMR spectra of (ZrPW$_{11}$)$_2$ in sodium phosphate buffer.

^{31}P NMR spectra for 10 mM solutions of **(ZrPW$_{11}$)$_2$** in 0.1 M sodium phosphate buffer (pH 3 – 8) that were recorded approximately one hour after preparation (**A**) and after incubation for 24 h at 37 °C (**B**). The chemical shifts and percentages of parent and formed species are given in **Tables S24** and **S25**. The structures of all POMs are shown in **Figure S115**. To identify the individual anions, they are shown in different colors, with the same color code being selected for a specific anion throughout all figures and tables in the main manuscript and the supporting information.

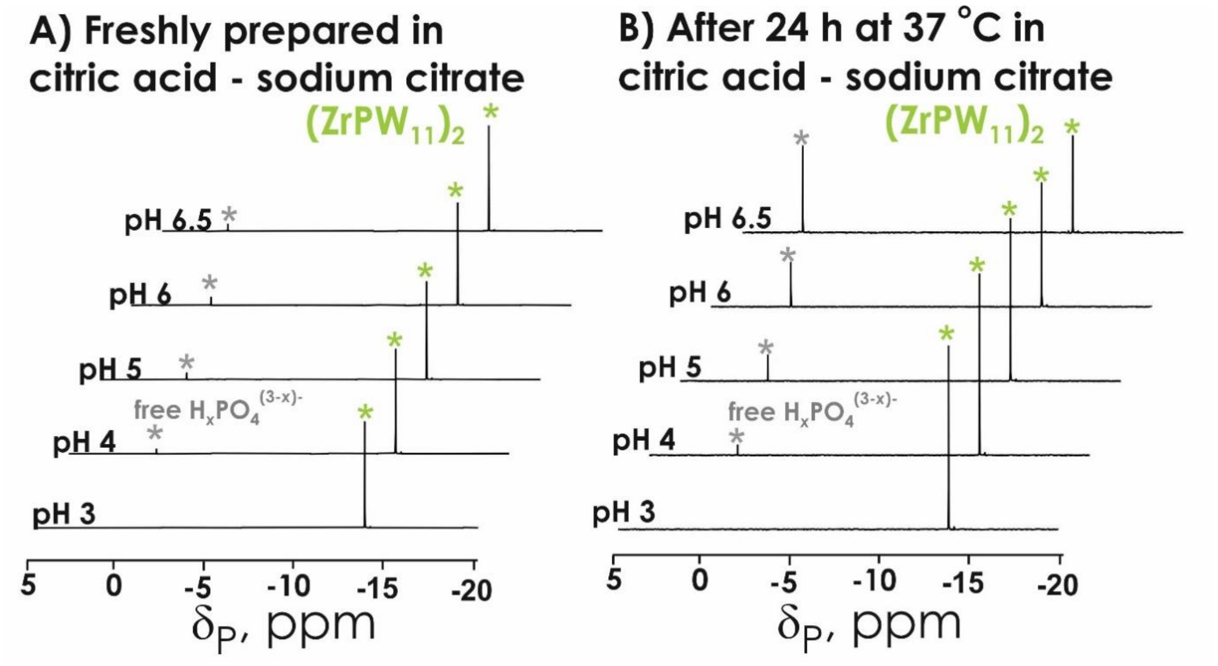

Fig. S120. ^{31}P NMR spectra of $(ZrPW_{11})_2$ in citric acid – sodium citrate buffer.

^{31}P NMR spectra for 10 mM solutions of **$(ZrPW_{11})_2$** in 0.1 M citric acid – sodium citrate buffer (pH 3 – 6.5) that were recorded approximately one hour after preparation (**A**) and after incubation for 24 h at 37 °C (**B**). The chemical shifts and percentages of parent and formed species are given in **Tables S24** and **S25**. The structures of all POMs are shown in **Figure S115**. To identify the individual anions, they are shown in different colors, with the same color code being selected for a specific anion throughout all figures and tables in the main manuscript and the supporting information.

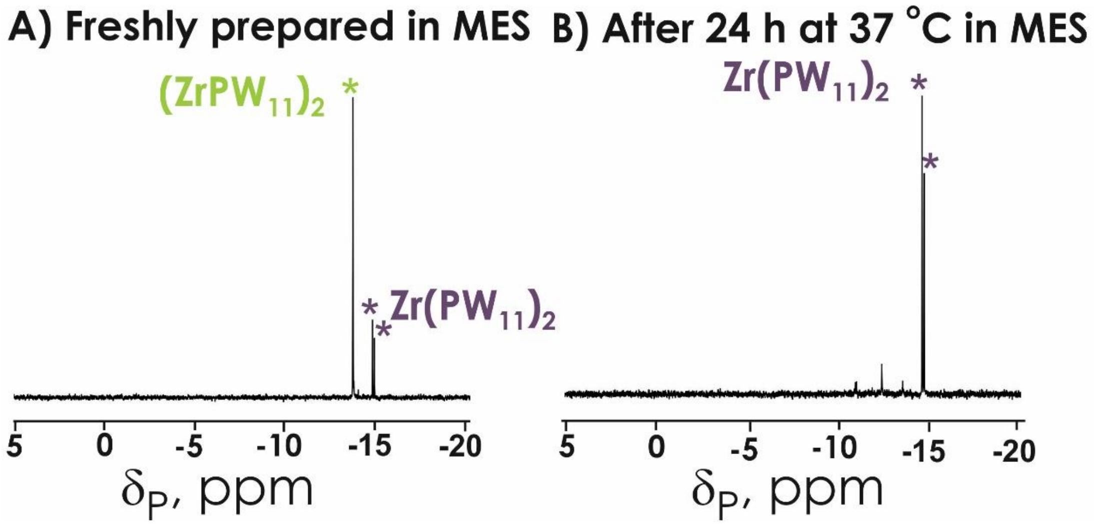

Fig. S121. ^{31}P NMR spectra of (ZrPW$_{11}$)$_2$ in MES buffer.

^{31}P NMR spectra for 10 mM solutions of **(ZrPW$_{11}$)$_2$** in 0.1 M MES buffer pH 5.5 that were recorded approximately one hour after preparation (**A**) and after incubation for 24 h at 37 °C (**B**). The chemical shifts and percentages of parent and formed species are given in **Tables S24** and **S25**. The structures of all POMs are shown in **Figure S115**. To identify the individual anions, they are shown in different colors, with the same color code being selected for a specific anion throughout all figures and tables in the main manuscript and the supporting information.

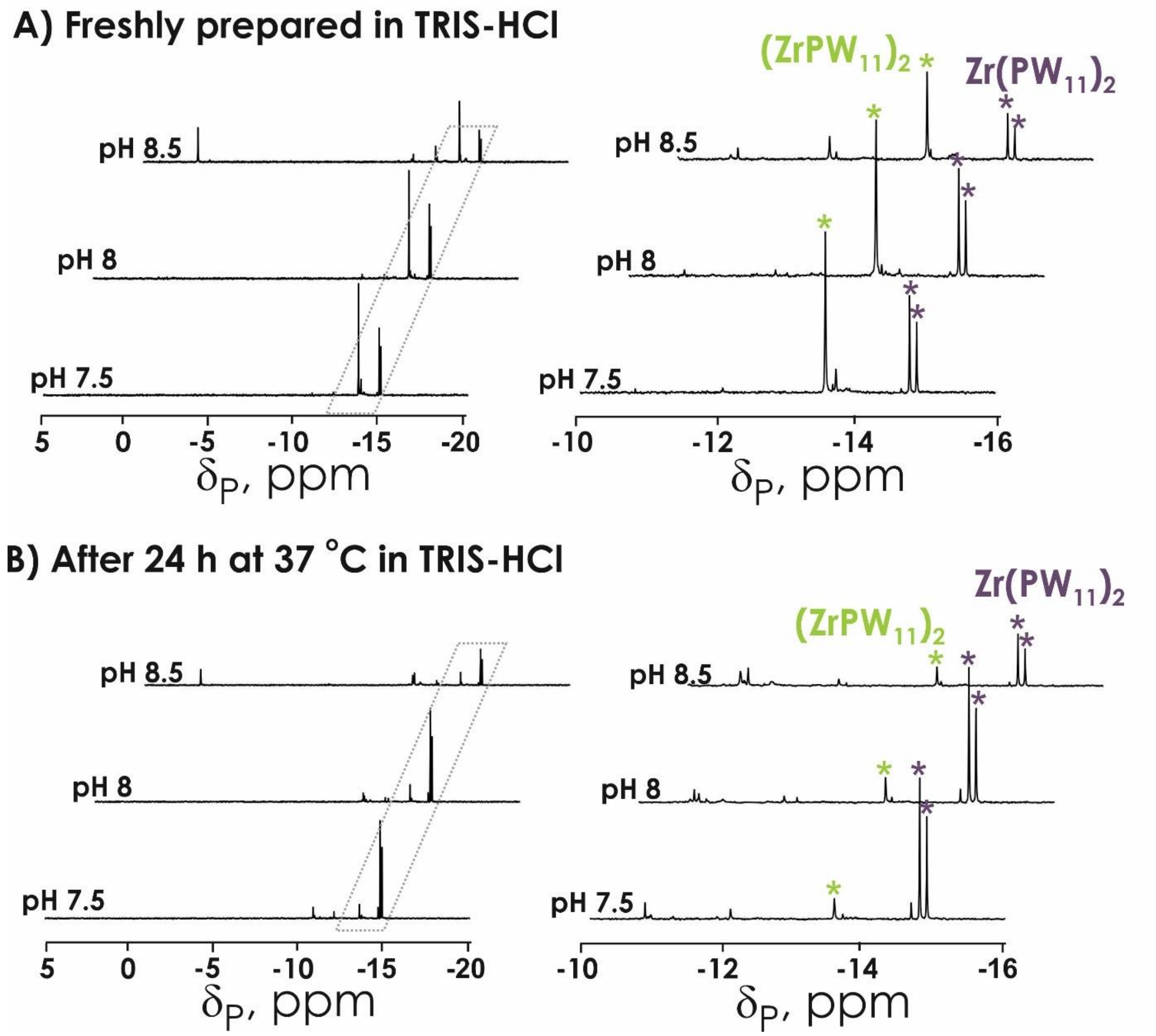

Fig. S122. ^{31}P NMR spectra of $(ZrPW_{11})_2$ in tris-HCl buffer.

^{31}P NMR spectra for 10 mM solutions of $\mathbf{(ZrPW_{11})_2}$ in 0.1 M tris-HCl buffer (pH 7.5 – 8.5) that were recorded approximately one hour after preparation (**A**) and after incubation for 24 h at 37 °C (**B**). The chemical shifts and percentages of parent and formed species are given in **Tables S24** and **S25**. The structures of all POMs are shown in **Figure S115**. To identify the individual anions, they are shown in different colors, with the same color code being selected for a specific anion throughout all figures and tables in the main manuscript and the supporting information.

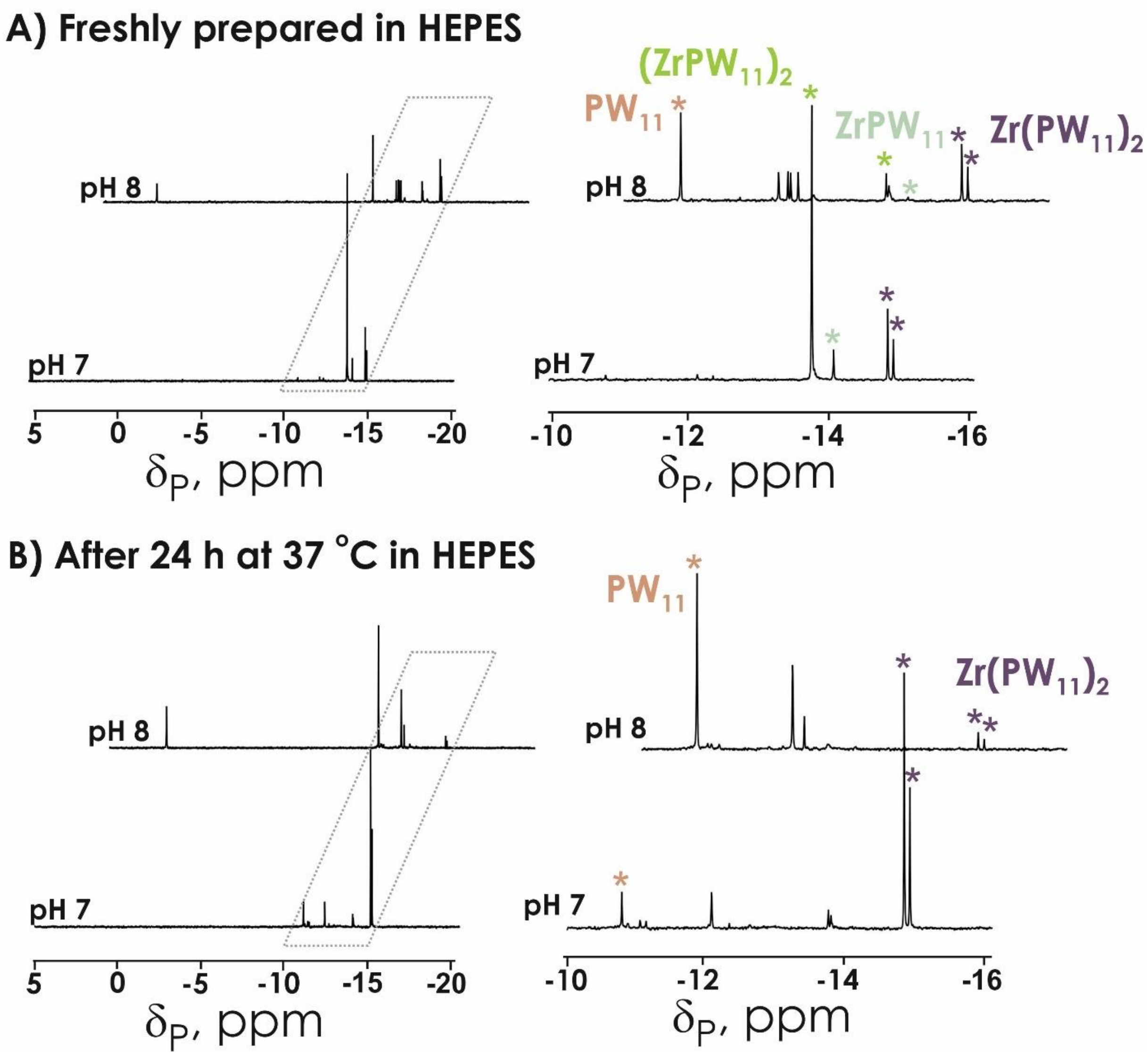

Fig. S123. ^{31}P NMR spectra of $(ZrPW_{11})_2$ in HEPES buffer.

^{31}P NMR spectra for 10 mM solutions of **$(ZrPW_{11})_2$** in 0.1 M HEPES buffer (pH 7 and 8) that were recorded approximately one hour after preparation (**A**) and after incubation for 24 h at 37 °C (**B**). The chemical shifts and percentages of parent and formed species are given in **Tables S24** and **S25**. The structures of all POMs are shown in **Figure S115**. To identify the individual anions, they are shown in different colors, with the same color code being selected for a specific anion throughout all figures and tables in the main manuscript and the supporting information.

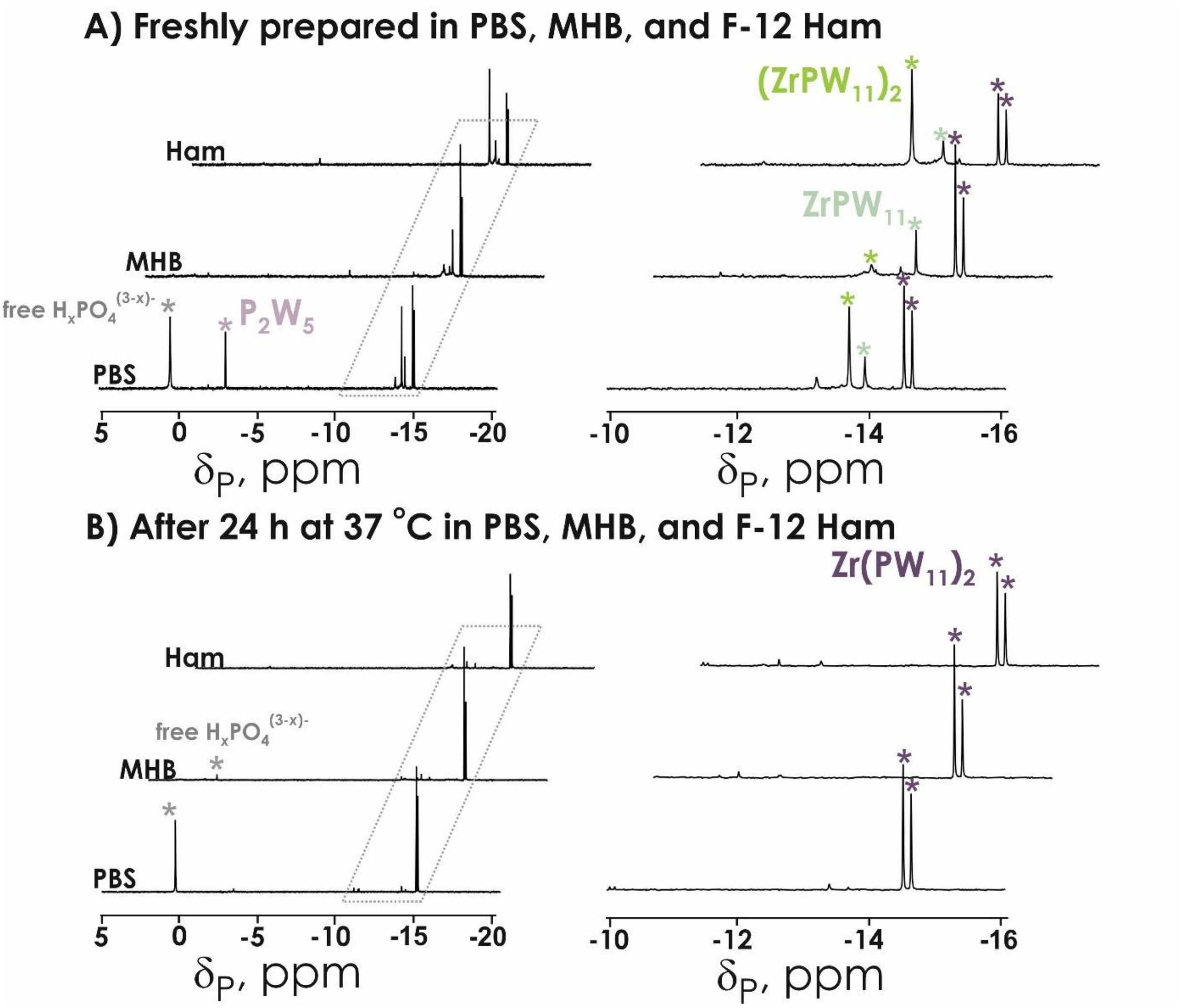

Fig. S124. ^{31}P NMR spectra of $(ZrPW_{11})_2$ in solutions with pH 7.4.

^{31}P NMR spectra for 10 mM solutions of **$(ZrPW_{11})_2$** in 0.1 M PBS, MHB and Ham's (pH 7.4) that were recorded approximately one hour after preparation (**A**) and after incubation for 24 h at 37 °C (**B**). The chemical shifts and percentages of parent and formed species are given in **Tables S24** and **S25**. The structures of all POMs are shown in **Figure S115**. To identify the individual anions, they are shown in different colors, with the same color code being selected for a specific anion throughout all figures and tables in the main manuscript and the supporting information.

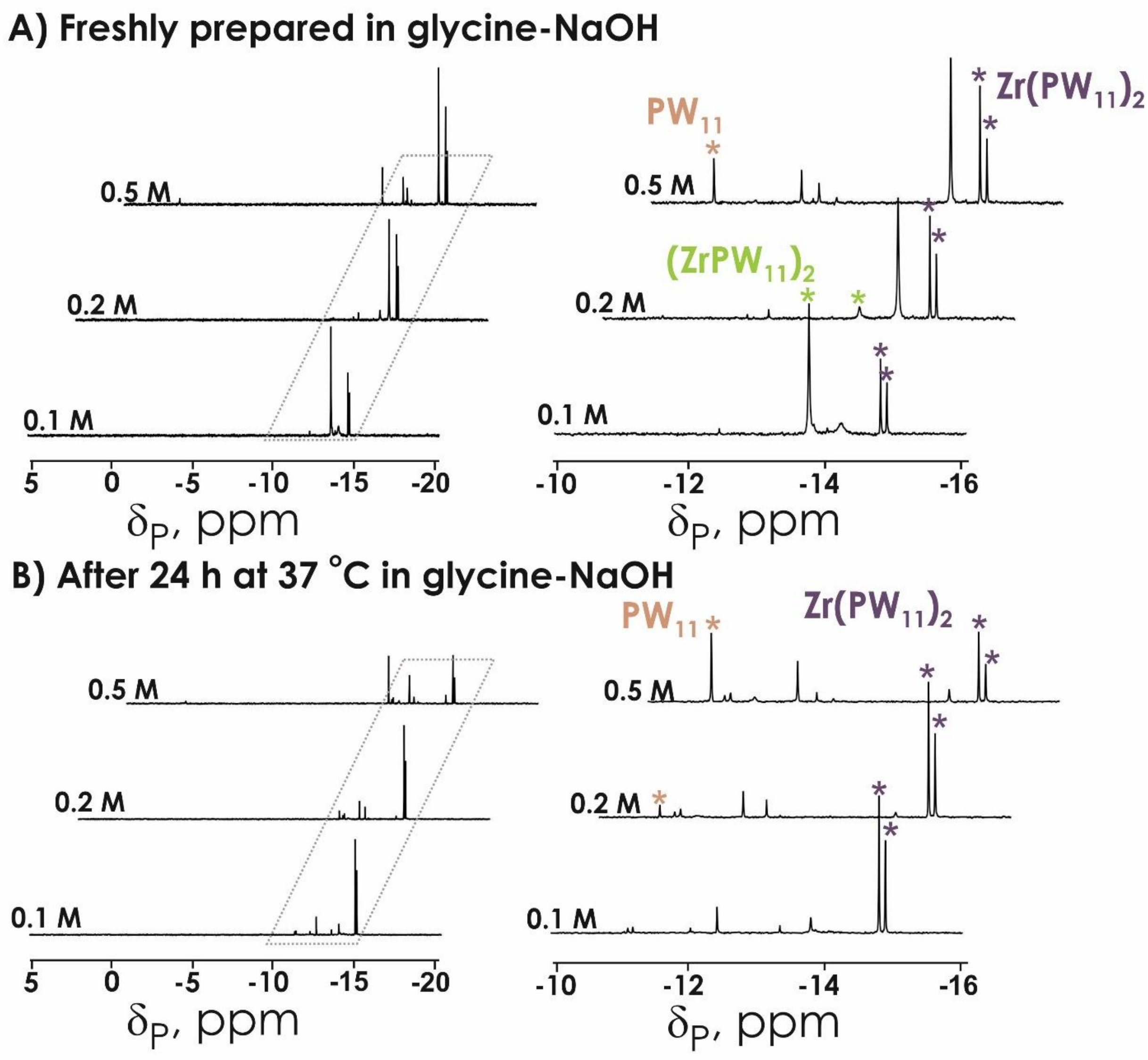

Fig. S125. ^{31}P NMR spectra of $(ZrPW_{11})_2$ in glycine-NaOH buffer.

^{31}P NMR spectra for 10 mM solutions of **$(ZrPW_{11})_2$** in glycine-NaOH (pH 8.6) with concentration 0.1, 0.2 and 0.5 M. that were recorded approximately one hour after preparation (**A**) and after incubation for 24 h at 37 °C (**B**). The chemical shifts and percentages of parent and formed species are given in **Tables S24** and **S25**. The structures of all POMs are shown in **Figure S11** and **S115**. To identify the individual anions, they are shown in different colors, with the same color code being selected for a specific anion throughout all figures and tables in the main manuscript and the supporting information.

Table S24. Analysis of NMR spectroscopic data recorded in $(ZrPW_{11})_2$ solutions at room temperature.

Chemical shifts in ^{31}P NMR spectra measured in triplicate of **$(ZrPW_{11})_2$** (10 mM) dissolved D_2O and 0.1 M buffers (acetic acid – sodium acetate pH 4 – 5.5; sodium phosphate pH 3 – 8 (while phosphate does not buffer at pH range from 3.5 – 5.5, experiments were conducted at this pH to provide comparisons to previously published studies (*29*)); citric acid – sodium citrate pH 3 – 6.5; MES pH 5.5; PBS pH 7.4; tris-HCl pH 7.5 – 8.5; HEPES pH 7 – 8; MHB pH 7.4; Nutrient mixture F-12 Ham pH 7.4 and glycine-NaOH pH 8.6) (**Figures S117** – **S125**). The glycine-NaOH buffer was used in two additional concentrations 0.2 and 0.5 M. The species content was calculated based on the integration of ^{31}P signals considering only signals associated with POTs. Signals were assigned based on the literature data summarized in **Table S4**.

**pH**	**$(ZrPW_{11})_2$** (10 mM) in Solvent / Buffer / Medium	Chemical shifts δ ^{31}P [ppm]	% of $(ZrPW_{11})_2$ in 10 mM **$(ZrPW_{11})_2$** at RT				% of $ZrPW_{11}$ in 10 mM **$(ZrPW_{11})_2$** at RT				% of $Zr(PW_{11})_2$ in 10 mM **$(ZrPW_{11})_2$** at RT				% of PW_{11} in 10 mM **$(ZrPW_{11})_2$** at RT				% of P_2W_5 in 10 mM **$(ZrPW_{11})_2$** at RT				Average % of other POTs
			Sample			Mean of 1 to 3 ± SD[a]	Sample			Mean of 1 to 3 ± SD[a]	Sample			Mean of 1 to 3 ± SD[a]	Sample			Mean of 1 to 3 ± SD[a]	Sample			Mean of 1 to 3 ± SD[a]	
			#1	#2	#3		#1	#2	#3		#1	#2	#3		#1	#2	#3		#1	#2	#3		
-	D_2O	**–13.7**; –14.0; **–14.8**; **–14.9**	84	79	79	81 ± 3	2	1	1	1 ± 1	14	20	20	18 ± 3	0	0	0	0	0	0	0	0	0
Strongly acidic environment 3 ≤ pH ≤ 4																							
3	0.1 M Sodium phosphate ($H_2PO_4^-$ /H_3PO_4) pH 3	0.3; –12.4; –14.0; –14.3; –14.4; **–14.8**; **–14.9**	0	0	0	0	95	95	95	95 ± 0	3	3	4	3 ± 1	0	0	0	0	0	0	0	0	2
	0.1 M Citric acid – sodium citrate (H_3Cit/H_2Cit^-) pH 3	**–13.8**; –14.1	99	100	99	99 ± 1	1	0	1	1 ± 0	0	0	0	0	0	0	0	0	0	0	0	0	0
4	0.1 M Sodium phosphate ($H_2PO_4^-$ /H_3PO_4) pH 4	2.1; –0.1; –12.4; –14.1; –14.4; **–14.8**; **–14.9**	0	0	0	0	95	95	95	95 ± 0	3	3	3	3 ± 0	0	0	0	0	0	0	0	0	2
	0.1 M Citric acid – sodium citrate (H_2Cit^- /$HCit^{2-}$) pH 4	0; **–13.8**; –14.1	99	99	99	99 ± 0	1	1	1	1 ± 0	0	0	0	0	0	0	0	0	0	0	0	0	0

	0.1 M Acetic acid – sodium acetate (OAc^{-}/HOAc) pH 4	–14.0; –14.8; –14.9	0	0	0	0	78	72	75	75 ± 3	22	28	25	25 ± 3	0	0	0	0	0	0	0	0	0
Moderately acidic environment 5 ≤ pH ≤ 6																							
5	0.1 M Sodium phosphate ($H_2PO_4^{-}$/H_3PO_4) pH 5	2.2; 0.1; –2.4; –12.4; –14.1; –14.4; –14.8; –14.9	0	0	0	0	95	95	92	94 ± 2	2	2	5	3 ± 2	0	0	0	0	1	1	1	1 ± 0	3
	0.1 M Citric acid – sodium citrate (H_2Cit^{-}/$HCit^{2-}$) pH 5	0; –13.8; –14.1	99	99	99	99 ± 0	1	1	1	1 ± 0	0	0	0	0	0	0	0	0	0	0	0	0	0
	0.1 M Acetic acid – sodium acetate (OAc^{-}/HOAc) pH 5	–13.9; –14.1; –14.8; –14.9	59	64	66	63 ± 4	20	21	21	21 ± 1	21	15	13	16 ± 4	0	0	0	0	0	0	0	0	0
5.5	0.1 M Acetic acid – sodium acetate (OAc^{-}/HOAc) pH 5.5	–13.8; –14.0; –14.8; –14.9	76	78	72	75 ± 3	6	7	8	7 ± 1	18	15	20	18 ± 3	0	0	0	0	0	0	0	0	0
	0.1 M MES[b] pH 5.5	–13.6; –13.9; –14.7; –14.8	70	73	70	71 ± 2	0	0	0	0	30	27	30	29 ± 2	0	0	0	0	0	0	0	0	0
6	0.1 M Sodium phosphate (HPO_4^{2-}/$H_2PO_4^{-}$) pH 6	0.2; –2.4; –3.0; –10.9; –14.0; –14.8; –14.9	0	0	0	0	17	23	17	19 ± 3	66	63	66	65 ± 2	0	0	0	0	17	14	17	16 ± 2	0
	0.1 M Citric acid – sodium citrate ($HCit^{2-}$/Cit^{3-}) pH 6	0.4; –11.6; –13.8; –14.1	99	99	99	99 ± 0	1	1	1	1 ± 0	0	0	0	0	0	0	0	0	0	0	0	0	0
Neutral environment 6.5 ≤ pH ≤ 7.5																							
6.5	0.1 M Citric acid – sodium citrate ($HCit^{2-}$/Cit^{3-}) pH 6.5	1.1; –13.8; –14.1	99	99	99	99 ± 0	1	1	1	1 ± 0	0	0	0	0	0	0	0	0	0	0	0	0	0
7	0.1 M Sodium phosphate (HPO_4^{2-}/$H_2PO_4^{-}$) pH 7	1.4; –2.4; –10.8; –14.8; –14.9	0	0	0	0	0	0	0	0	1	1	0	1 ± 1	67	70	60	66 ± 5	32	29	40	34 ± 6	0

	0.1 M HEPES[c] pH 7	–13.8; –14.1; –14.8; –14.9	74	70	72	72 ± 2	4	7	3	5 ± 2	22	23	24	23 ± 1	0	0	1	0	0	0	0	0	0
7.4	PBS[d] pH 7.4	0.3; –3.0; –13.7; –14.1; –14.3; –14.7; –14.8	8	4	4	5 ± 2	35	30	24	30 ± 6	30	36	43	36 ± 7	0	0	0	0	16	19	20	18 ± 2	29
7.4	MHB[e] pH 7.4	1.1; –7.8; –11.6; –13.9; –14.1; –14.3; –14.8; –14.9	13	20	12	15 ± 4	4	3	2	3 ± 1	63	59	68	63 ± 5	0	0	0	0	0	0	0	0	19
7.4	Nutrient mixture F-12 Ham[f]	–3.0; –11.8; –13.7; –13.9; –14.1; –14.3; –14.8; –14.9	60	61	59	60 ± 1	8	10	9	9 ± 1	24	26	25	25 ± 1	0	0	0	0	0	0	0	0	6
7.5	0.1 M tris-HCl[g] pH 7.5	–10.8; –12.1; –13.6; –13.7; –13.8; –13.0; –14.7; –14.8; –14.9	65	60	61	62 ± 3	0	0	0	0	34	39	38	37 ± 3	0	0	0	0	0	0	0	0	1
Moderately alkaline environment 8 ≤ pH ≤ 8.6																							
8	0.1 M Sodium phosphate (HPO_4^{2-}/$H_2PO_4^-$) pH 8	3.5; 1.8; –2.4; –10.8; –14.1	0	0	0	0	1	1	1	1 ± 0	0	1	0	0	67	69	57	64 ± 6	32	29	42	34 ± 7	0
8	0.1 M HEPES pH 8	1.9; –10.8; 12.1; 12.2; –12.3; –12.4; –	18	17	13	16 ± 3	0	0	0	0	26	22	17	22 ± 5	24	25	32	27 ± 4	0	0	0	0	35

		12.5; –12.7; –13.8; –14.8; –14.9																					
	0.1 M tris-HCl pH 8	–10.9; –12.1; –12.3; –13.6; –13.7; –13.8; –13.9; –14.7; –14.8; –14.9	58	58	46	54 ± 7	0	0	0	0	41	39	52	44 ± 7	0	0	0	0	0	0	0	0	0
8.5	0.1 M tris-HCl pH 8.5	1.8; 1.1; –10.8; –10.9; –12.2; –12.3; –13.6; –13.7; –14.0; –14.8; –14.9	41	52	43	45 ± 6	7	2	7	5 ± 3	31	29	30	30 ± 1	7	3	7	6 ± 2	0	0	0	0	14
	0.1 M glycine-NaOH pH 8.6	–12.4; –13.7; –14.0; –14.2; –14.8; –14.9	64	62	52	59 ± 6	1	1	0	1 ± 0	24	24	35	28 ± 6	0	0	0	0	0	0	0	0	12
8.6	0.2 M glycine-NaOH pH 8.6	–12.4; –13.8; –14.3; –14.8; –14.9	13	6	9	9 ± 3	0	0	0	0	34	33	35	34 ± 1	0	0	0	0	0	0	0	0	57
	0.5 M glycine-NaOH pH 8.6	1.6; –10.9; –12.2; –12.4; –12.5; –12.7; –14.4; –	0	0	0	0	0	0	0	0	35	34	30	33 ± 3	7	9	8	8 ± 1	0	0	0	0	59

		**14.8**; **– 14.9**																					

[a]SD – standard deviation; [b]MES – 2-(N-morpholino)ethanesulfonic acid, $C_6H_{13}NO_4S$ (Figure S1); [c]HEPES – 4-(2-hydroxyethyl)-1-piperazineethanesulfonic acid, $C_8H_{18}N_2O_4S$ (Figure S1); [d]PBS – phosphate buffer saline; [e]MHB – Mueller-Hinton broth, for more detailed information about composition, see https://labmal.com/2019/11/20/mueller-hinton-agar-and-mueller-hinton-broth/; [f]Nutrient mixture F-12 Ham contains sodium pyruvate (0.11 g/L), phenol red, L-glutamine, and does not contain $NaHCO_3$ and HEPES, for more details please see https://www.sigmaaldrich.com/AT/en/technical-documents/technical-article/cell-culture-and-cell-culture-analysis/mammalian-cell-culture/f-12-ham; [g]tris – tris(hydroxymethyl)aminomethane, $C_4H_{11}NO_3$ (Figure S1).

Table S25. Analysis of NMR spectroscopic data of $(ZrPW_{11})_2$ solutions investigated after 24 h incubation at 37 °C.

Chemical shifts in ^{31}P NMR spectra measured in triplicate of **$(ZrPW_{11})_2$** (10 mM) dissolved D_2O and 0.1 M buffers (acetic acid – sodium acetate pH 4 – 5.5; sodium phosphate pH 3 – 8 (while phosphate does not buffer at pH range from 3.5 – 5.5, experiments were conducted at this pH to provide comparisons to previously published studies (*29*)); citric acid – sodium citrate pH 3 – 6.5; MES pH 5.5; PBS pH 7.4; tris-HCl pH 7.5 – 8.5; HEPES pH 7 – 8; MHB pH 7.4; Nutrient mixture F-12 Ham pH 7.4 and glycine-NaOH pH 8.6) and investigated after 24 h incubation at 37 °C (**Figures S117 – S125**). The glycine-NaOH buffer was used in two additional concentrations 0.2 and 0.5 M. The species content was calculated based on the integration of ^{31}P signals considering only signals associated with POTs. Signals were assigned based on the literature data summarized in **Table S4**.

pH	**$(ZrPW_{11})_2$** (10 mM) in Solvent / Buffer / Medium	Chemical shifts δ ^{31}P [ppm]	% of **$(ZrPW_{11})_2$** in 10 mM **$(ZrPW_{11})_2$** after 24 h incubation at 37 °C				% of **$ZrPW_{11}$** in 10 mM **$(ZrPW_{11})_2$** after 24 h incubation at 37 °C				% of **$Zr(PW_{11})_2$** in 10 mM **$(ZrPW_{11})_2$** after 24 h incubation at 37 °C				% of **PW_{11}** in 10 mM **$(ZrPW_{11})_2$** after 24 h incubation at 37 °C				% of **P_2W_5** in 10 mM **$(ZrPW_{11})_2$** after 24 h incubation at 37 °C				Average % of other POTs
			Sample			Mean of 1 to 3 ± SD[a]	Sample			Mean of 1 to 3 ± SD[a]	Sample			Mean of 1 to 3 ± SD[a]	Sample			Mean of 1 to 3 ± SD[a]	Sample			Mean of 1 to 3 ± SD[a]	
			#1	#2	#3		#1	#2	#3		#1	#2	#3		#1	#2	#3		#1	#2	#3		
-	D_2O	**–13.7**; –14.0; **–14.8**; **–14.9**	77	75	76	76 ± 1	1	1	1	1 ± 0	23	24	23	23 ± 1	0	0	0	0	0	0	0	0	0
Strongly acidic environment 3 ≤ pH ≤ 4																							
3	0.1 M Sodium phosphate ($H_2PO_4^–$ /H_3PO_4) pH 3	–0.3; –14.0; **–14.8**; **–14.9**	0	0	0	0	83	85	90	86 ± 4	17	15	10	14 ± 4	0	0	0	0	0	0	0	0	0
3	0.1 M Citric acid – sodium citrate ($H_3Cit/H_2Cit^–$) pH 3	**–13.8**; –14.1	99	99	99	99 ± 0	1	1	1	1 ± 0	0	0	0	0	0	0	0	0	0	0	0	0	0
4	0.1 M Sodium phosphate ($H_2PO_4^–$ /H_3PO_4) pH 4	0.1; –12.4; –14.1; –14.2; –14.3; **–14.8**; **–14.9**	0	0	0	0	57	50	50	52 ± 4	42	49	49	47 ± 4	0	0	0	0	0	0	0	0	1
4	0.1 M Citric acid – sodium citrate ($H_2Cit^–$ /$HCit^{2–}$) pH 4	0; **–13.8**; –14.1	99	99	99	99 ± 0	1	1	1	1 ± 0	0	0	0	0	0	0	0	0	0	0	0	0	0
4	0.1 M Acetic acid – sodium acetate ($OAc^–$ /HOAc) pH 4	–14.0; **–14.8**; **–14.9**	0	0	0	0	75	72	76	74 ± 2	24	28	24	25 ± 2	0	0	0	0	0	0	0	0	0
Moderately acidic environment 5 ≤ pH ≤ 6																							
5	0.1 M Sodium phosphate	0; –12.4; **–13.8**; –14.1;	0	0	0	0	39	33	33	35 ± 3	59	65	65	63 ± 3	0	0	0	0	0	0	0	0	2

	($H_2PO_4^-$/H_3PO_4) pH 5	–14.2; –14.3; –14.8; –14.9																					
	0.1 M Citric acid – sodium citrate (H_2Cit^-/$HCit^{2-}$) pH 5	0; –13.8; –14.1	99	99	99	99 ± 0	1	1	1	1 ± 0	0	0	0	0	0	0	0	0	0	0	0	0	0
	0.1 M Acetic acid – sodium acetate (OAc^-/HOAc) pH 5	–11.1; –11.1; –13.3; –13.9; –14.1; –14.8; –14.9	34	35	32	34 ± 2	15	16	17	16 ± 1	47	43	44	45 ± 2	0	0	0	0	0	0	0	0	5
5.5	0.1 M Acetic acid – sodium acetate (OAc^-/HOAc) pH 5.5	–11.1; –11.1; –13.4; –13.8; –14.1; –14.8; –14.9	25	22	19	22 ± 3	1	1	1	1 ± 0	62	64	69	65 ± 4	0	0	0	0	0	0	0	0	12
	0.1 M MES[b] pH 5.5	–11.0; –11.1; –12.5; –13.6; –14.7; –14.8	2	1	6	3 ± 2	0	0	0	0	93	92	87	91 ± 3	0	0	1	0 ± 1	0	0	0	0	6
6	0.1 M Sodium phosphate (HPO_4^{2-}/$H_2PO_4^-$) pH 6	0.2; –2.4; –10.9; –14.8; –14.9	0	0	0	0	0	0	0	0	71	75	73	73 ± 2	15	13	17	15 ± 2	14	12	10	12 ± 2	0
	0.1 M Citric acid – sodium citrate ($HCit^{2-}$/Cit^{3-}) pH 6	0.5; –12.1; –13.8; –14.1	98	98	98	98 ± 0	2	2	2	2 ± 0	0	0	0	0	0	0	0	0	0	0	0	0	0
Neutral environment 6.5 ≤ pH ≤ 7.5																							
6.5	0.1 M Citric acid – sodium citrate ($HCit^{2-}$/Cit^{3-}) pH 6.5	1.5; –11.5; –12.2; –13.5; –13.8; –14.1	99	98	98	98 ± 1	0	1	0	1 ± 1	0	0	0	0	0	0	0	0	0	0	0	0	1
7	0.1 M Sodium phosphate (HPO_4^{2-}/$H_2PO_4^-$) pH 7	3.5; 1.4; –2.4; –10.8; –11.4	0	0	0	0	0	0	0	0	0	0	0	0	31	38	22	30 ± 8	69	62	78	70 ± 8	0
	0.1 M HEPES[c] pH 7	–10.8; –10.9; –11.1; –11.2; –12.1; –13.8; –14.8; –14.9	7	6	4	6 ± 2	0	0	0	0	78	75	73	75 ± 1	8	7	14	10 ± 4	0	0	0	0	9
7.4	PBS[d] pH 7.4	0.2; –3.0; –11.0; –11.1;	2	3	4	3 ± 1	0	0	0	0	85	93	90	89 ± 4	0	1	3	1 ± 2	4	2	2	3 ± 1	4

		–13.7; –13.8; –14.1; –14.8; –14.9																					
	MHB[e] pH 7.4	0.8; –10.9; –11.0; –11.9; –12.1; –12.6; –14.8; –14.9	0	0	0	0	0	0	0	0	95	97	93	95 ± 2	2	1	2	2 ± 1	0	0	0	0	3
	Nutrient mixture F-12 Ham[f]	–11.0; –11.1; –12.0; –12.5; –13.7; –14.7; –14.8	10	5	5	7 ± 3	0	0	0	0	86	83	89	86 ± 3	0	0	0	0	0	0	0	0	7
7.5	0.1 M tris-HCl[g] pH 7.5	–10.8; –10.9; –12.1; –13.6; –13.7; –14.7; –14.8; –14.9	11	11	10	11 ± 1	0	0	0	0	74	76	73	74 ± 2	7	6	9	7 ± 2	0	0	0	0	8
Moderately alkaline environment 8 ≤ pH ≤ 8.6																							
	0.1 M Sodium phosphate (HPO_4^{2-}/$H_2PO_4^-$) pH 8	3.5; 1.7; –2.4; –10.8; –11.4	0	0	0	0	0	0	1	1 ± 1	0	0	0	0	32	35	30	32 ± 3	68	65	69	67 ± 2	0
8	0.1 M HEPES pH 8	1.7; –10.8; –12.2; –12.3; –12.4; –12.7; –14.8; –14.9	0	0	0	0	0	0	0	0	10	5	4	6 ± 3	54	58	56	56 ± 2	0	0	0	0	38
	0.1 M tris-HCl pH 8	1.0; –10.8; –10.9; –12.1; –12.3; –13.6; –13.7; –14.7; –14.8; –14.9	12	11	11	11 ± 1	0	0	0	0	74	76	75	75 ± 1	10	9	9	9 ± 1	0	0	0	0	5
8.5	0.1 M tris-HCl pH 8.5	1.7; –10.8; –10.9; –11.2; –12.2; –12.3; –13.6; –13.7; –14.7; –14.8; –14.9	10	14	13	12 ± 2	0	0	0	0	44	45	39	43 ± 3	34	31	35	33 ± 2	0	0	0	0	12
8.6	0.1 M glycine-NaOH pH 8.6	–11.0; –11.1; –12.0; –12.4; –13.3; –13.8; –14.1; –14.8; –14.9	10	9	6	8 ± 2	0	0	0	0	75	78	81	78 ± 3	0	0	0	0	0	0	0	0	14
	0.2 M glycine-NaOH pH 8.6	–10.8; –11.1; –11.2; –12.1;	0	0	2	1 ± 1	0	0	0	0	75	74	69	73 ± 3	4	3	5	4 ± 1	0	0	0	0	22

		–12.4; –12.6; –13.4; –14.3; **–14.8**; **–14.9**																					
	0.5 M glycine-NaOH pH 8.6	–10.9; –11.1; –11.2; –11.6; –12.2; –12.5; –12.7; –14.4; **–14.8**; **–14.9**	0	0	0	0	0	0	0	0	39	37	33	36 ± 3	25	27	30	27 ± 3	0	0	0	0	37

[a]SD – standard deviation; [b]MES – 2-(N-morpholino)ethanesulfonic acid, $C_6H_{13}NO_4S$ (Figure S1); [c]HEPES – 4-(2-hydroxyethyl)-1-piperazineethanesulfonic acid, $C_8H_{18}N_2O_4S$ (Figure S1); [d]PBS – phosphate buffer saline; [e]MHB – Mueller-Hinton broth, for more detailed information about composition, see https://labmal.com/2019/11/20/mueller-hinton-agar-and-mueller-hinton-broth/; [f]Nutrient mixture F-12 Ham contains sodium pyruvate (0.11 g/L), phenol red, L-glutamine, and does not contain $NaHCO_3$ and HEPES, for more details please see https://www.sigmaaldrich.com/AT/en/technical-documents/technical-article/cell-culture-and-cell-culture-analysis/mammalian-cell-culture/f-12-ham; [g]tris – tris(hydroxymethyl)aminomethane, $C_4H_{11}NO_3$ (Figure S1).

11.3 Speciation in $(ZrPW_{11})_2$ POT solutions

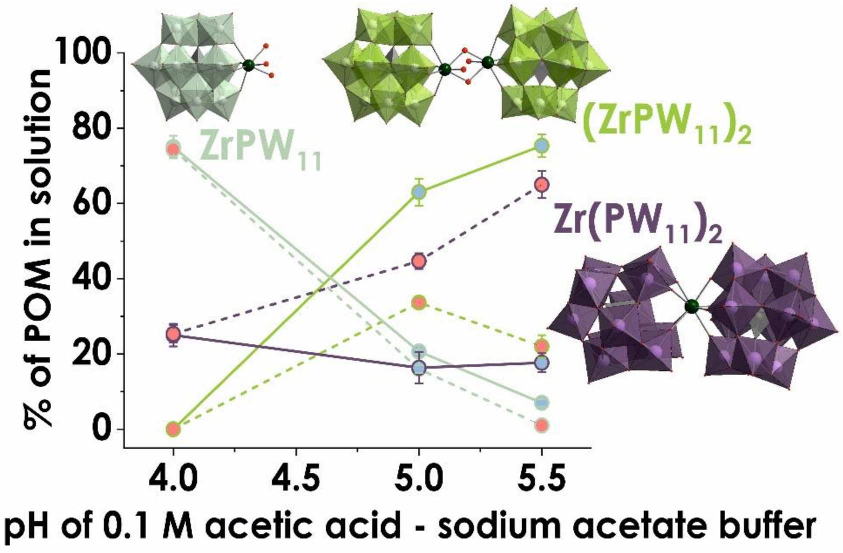

Fig. S126. Speciation of $(ZrPW_{11})_2$ in acetic acid – sodium acetate buffer.

POM concentration curves of **$(ZrPW_{11})_2$** (10 mM) in 0.1 M acetic acid – sodium acetate buffer solutions before (solid line, blue dot in the middle) and after incubation (dash line, red dot in the middle) for 24 h at 37 °C. The exact percentage of all POM species present is given in **Tables S24** and **S25**.

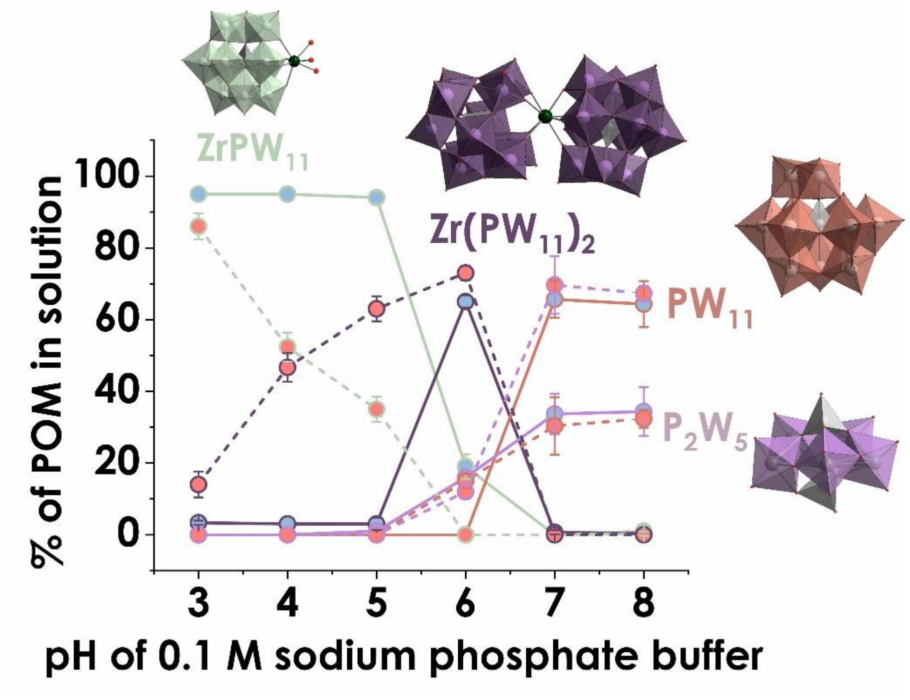

Fig. S127. Speciation of $(ZrPW_{11})_2$ in sodium phosphate buffer.

POM concentration curves of **$(ZrPW_{11})_2$** (10 mM) in 0.1 M sodium phosphate buffer solutions before (solid line, blue dot in the middle) and after incubation (dash line, red dot in the middle) for 24 h at 37 °C. The exact percentage of all POM species present is given in **Tables S24** and **S25**.

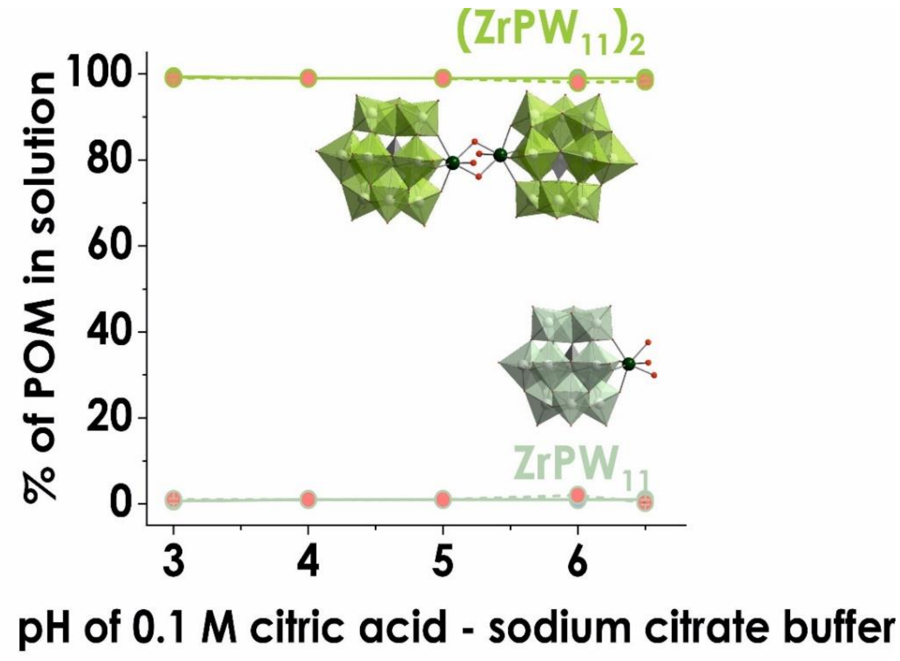

Fig. S128. Speciation of $(ZrPW_{11})_2$ in citric acid – sodium citrate buffer.

POM concentration curves of **$(ZrPW_{11})_2$** (10 mM) in 0.1 M citric acid – sodium citrate buffer solutions before (solid line, blue dot in the middle) and after incubation (dash line, red dot in the middle) for 24 h at 37 °C. The exact percentage of all POM species present is given in **Tables S24** and **S25**.

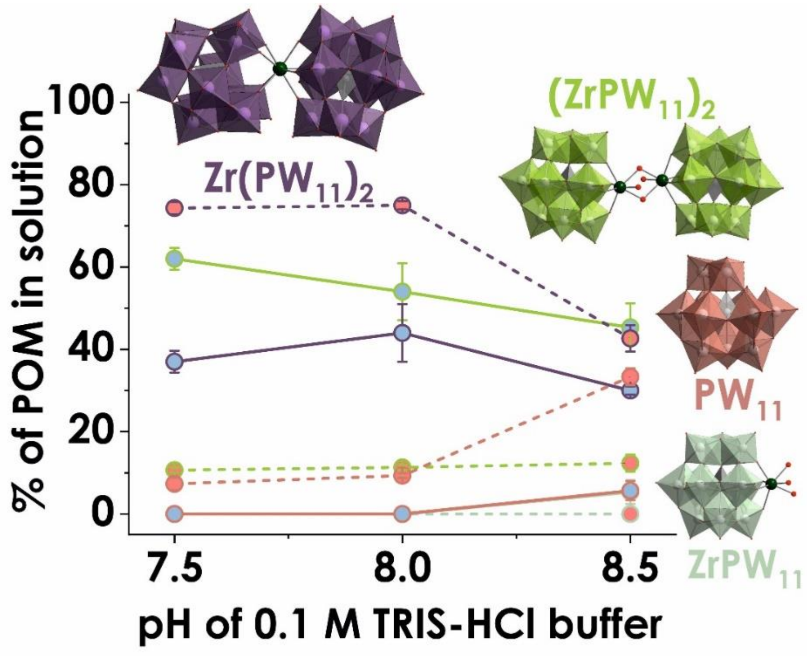

Fig. S129. Speciation of $(ZrPW_{11})_2$ in tris-HCl buffer.

POM concentration curves of $\mathbf{(ZrPW_{11})_2}$ (10 mM) in 0.1 M tris-HCl buffer solutions before (solid line, blue dot in the middle) and after incubation (dash line, red dot in the middle) for 24 h at 37 °C. The exact percentage of all POM species present is given in **Tables S24** and **S25**.

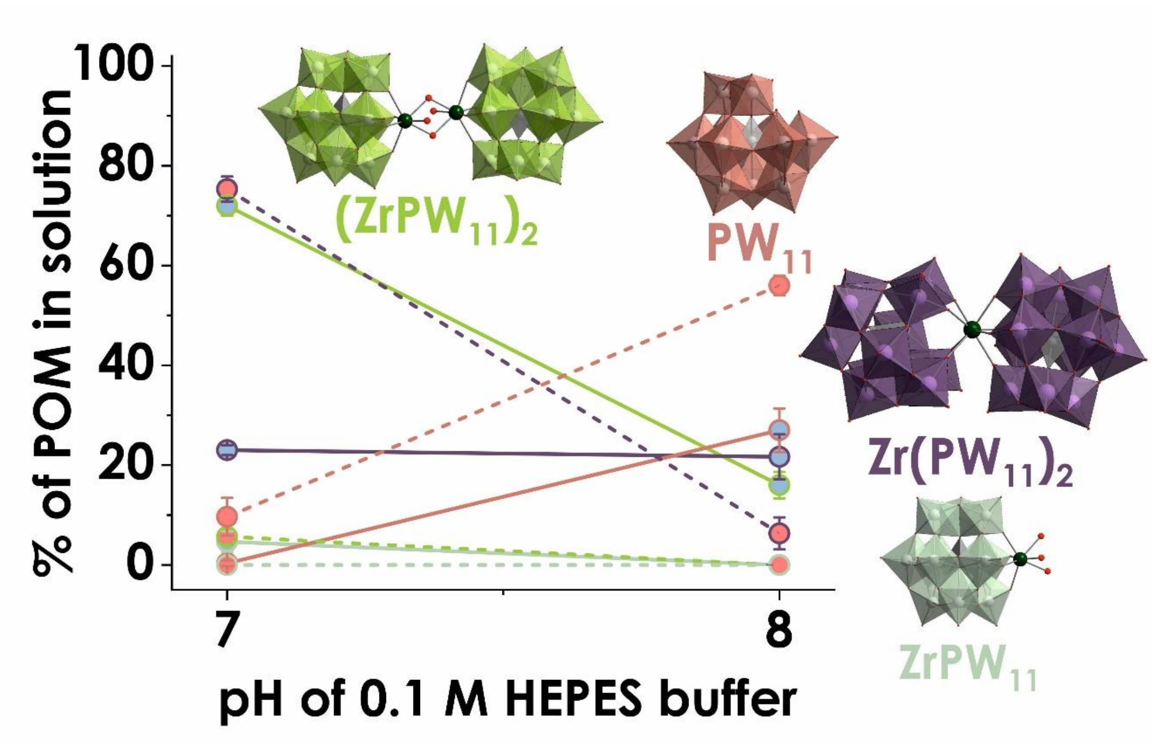

Fig. S130. Speciation of $(ZrPW_{11})_2$ in HEPES buffer.

POM concentration curves of $\mathbf{(ZrPW_{11})_2}$ (10 mM) in 0.1 M HEPES buffer solutions before (solid line, blue dot in the middle) and after incubation (dash line, red dot in the middle) for 24 h at 37 °C. The exact percentage of all POM species present is given in **Tables S24** and **S25**.

12. Decavanadate V_{10}

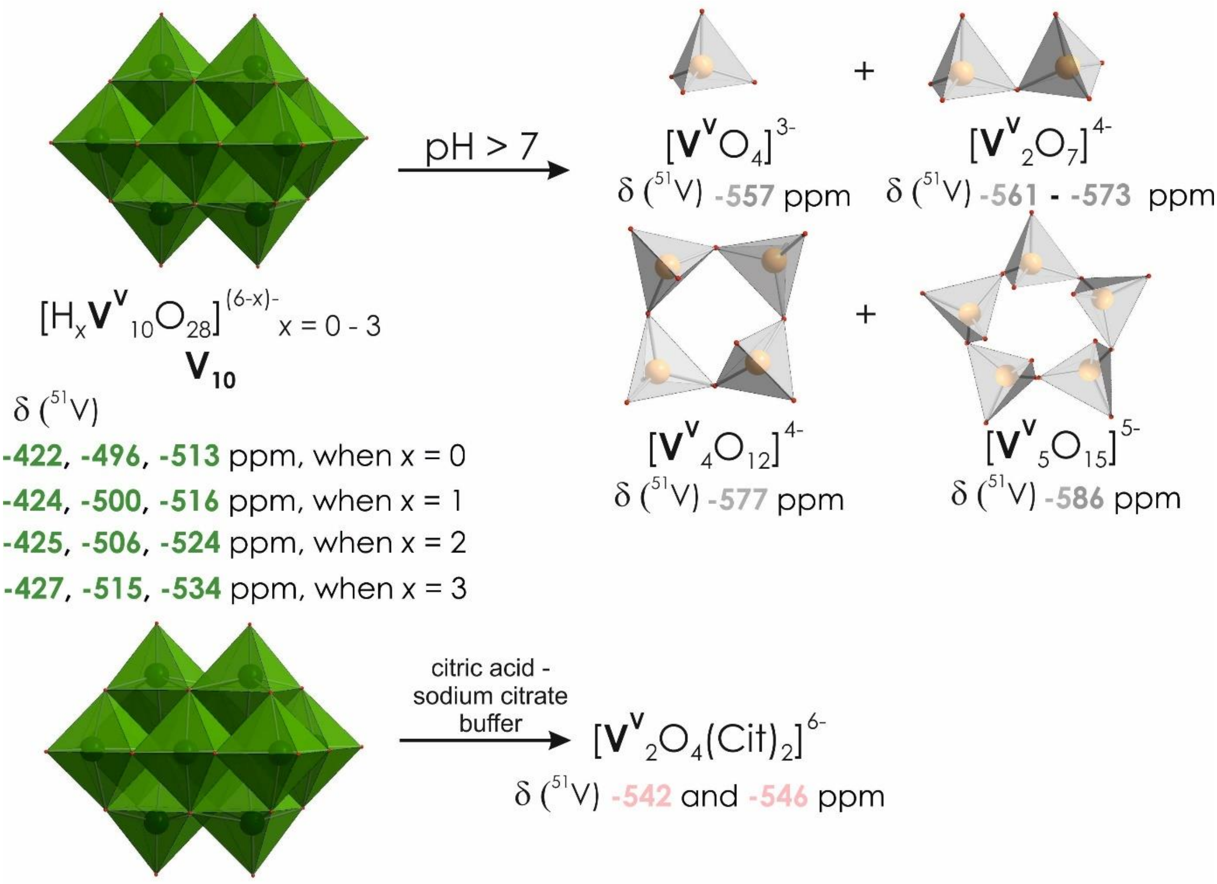

Fig. S131. Hydrolysis of V_{10}.

Structure of the decavanadate anion $[V^V{}_{10}O_{28}]^{6-}$ (**V_{10}**) and its hydrolysis scheme. The polyanions that can be formed during hydrolysis in the pH range from 4 to 8.6 are shown with corresponding ^{51}V NMR chemical shifts. Color code: {VO_6}, green; {VO_4}, grey; O, red. To identify the individual anions, they are shown in different colors, with the same color code being selected for a specific anion throughout all figures and tables in the main manuscript and the supporting information.

12.1. pH of decavanadate solutions

Table S26. pH in V_{10} solutions.

pH values measured in triplicate of **$Na_2K_4[V^V{}_{10}O_{28}]$** (10 mM) dissolved D_2O and 0.1 M buffers (acetic acid – sodium acetate pH 4 – 5.5; sodium phosphate pH 3 – 8 (while phosphate does not buffer at pH range from 3.5 – 5.5, experiments were conducted at this pH to provide comparisons to previously published studies (*29*)); citric acid – sodium citrate pH 3 – 6.5; MES pH 5.5; PBS pH 7.4; tris-HCl pH 7.5 – 8.5; HEPES pH 7 – 8; MHB pH 7.4; Nutrient mixture F-12 Ham pH 7.4 and glycine-NaOH pH 8.6). The glycine-NaOH buffer was used in two additional concentrations of 0.2 and 0.5 M.

pH	**$Na_2K_4[V^V{}_{10}O_{28}]$** (10 mM) in Solvent / Buffer / Medium	pH after dissolving **$Na_2K_4[V^V{}_{10}O_{28}]$** (10 mM) at RT				pH after 24 h incubation of **$Na_2K_4[V^V{}_{10}O_{28}]$** (10 mM) solution at 37 °C			
		Sample			Mean of 1 to 3 ± SD[a]	Sample			Mean of 1 to 3 ± SD[a]
		#1	#2	#3		#1	#2	#3	
-	D_2O	5.54	6.14	5.52	5.73 ± 0.35	5.43	5.74	5.45	5.54 ± 0.17
Strongly acidic environment 3 ≤ pH ≤ 4									
3	0.1 M Sodium phosphate ($H_2PO_4^-$/H_3PO_4) pH 3	4.16	4.34	4.07	4.19 ± 0.13	3.87	4.46	4.14	4.16 ± 0.30
	0.1 M Citric acid – sodium citrate (H_3Cit/H_2Cit^-) pH 3	2.75	2.87	2.51	2.71 ± 0.18	2.96	2.97	2.92	2.95 ± 0.03
4	0.1 M Sodium phosphate ($H_2PO_4^-$/H_3PO_4) pH 4	4.71	5.12	4.80	4.88 ± 0.22	4.75	5.19	4.84	4.93 ± 0.23
	0.1 M Citric acid – sodium citrate (H_2Cit^-/$HCit^{2-}$) pH 4	4.12	4.22	4.19	4.17 ± 0.05	4.44	4.44	4.33	4.40 ± 0.06
	0.1 M Acetic acid – sodium acetate (OAc^-/HOAc) pH 4	4.12	4.27	4.20	4.20 ± 0.08	4.11	4.28	4.16	4.18 ± 0.09
Moderately acidic environment 5 ≤ pH ≤ 6									
5	0.1 M Sodium phosphate ($H_2PO_4^-$/H_3PO_4) pH 5	5.01	5.32	5.02	5.12 ± 0.18	5.01	5.36	5.05	5.14 ± 0.19
	0.1 M Citric acid – sodium citrate (H_2Cit^-/$HCit^{2-}$) pH 5	4.97	5.06	5.00	5.01 ± 0.05	5.73	6.02	5.96	5.90 ± 0.15
	0.1 M Acetic acid – sodium acetate (OAc^-/HOAc) pH 5	4.98	5.09	5.02	5.03 ± 0.06	4.97	5.10	5.01	5.03 ± 0.07

5.5	0.1 M Acetic acid – sodium acetate ($OAc^-/HOAc$) pH 5.5	5.47	5.56	5.45	5.49 ± 0.06	5.44	5.55	5.45	5.48 ± 0.06
	0.1 M MES[b] pH 5.5	5.27	5.45	5.26	5.33 ± 0.11	5.23	5.40	5.25	5.29 ± 0.09
6	0.1 M Sodium phosphate ($HPO_4^{2-}/H_2PO_4^-$) pH 6	5.87	5.82	5.68	5.79 ± 0.10	5.70	5.78	5.70	5.73 ± 0.05
	0.1 M Citric acid – sodium citrate ($HCit^{2-}/Cit^{3-}$) pH 6	6.58	6.08	6.01	6.22 ± 0.31	7.27	6.93	6.95	7.05 ± 0.19
	Neutral environment 6.5 ≤ pH ≤ 7.5								
6.5	0.1 M Citric acid – sodium citrate ($HCit^{2-}/Cit^{3-}$) pH 6.5	6.37	6.74	6.60	6.57 ± 0.19	7.32	7.10	7.12	7.18 ± 0.12
7	0.1 M Sodium phosphate ($HPO_4^{2-}/H_2PO_4^-$) pH 7	6.87	6.78	6.68	6.78 ± 0.10	6.36	6.29	6.28	6.31 ± 0.04
	0.1 M HEPES[c] pH 7	6.71	6.88	6.39	6.66 ± 0.25	6.29	6.36	6.24	6.30 ± 0.06
7.4	PBS[d] pH 7.4	5.95	5.82	5.74	5.83 ± 0.11	5.87	5.86	5.69	5.81 ± 0.11
	MHB[e] pH 7.4	6.59	6.34	6.61	6.51 ± 0.15	6.06	6.04	5.83	5.97 ± 0.13
	Nutrient mixture F-12 Ham[f]	6.26	6.28	6.35	6.30 ± 0.05	5.96	6.00	6.00	5.99 ± 0.02
7.5	0.1 M tris-HCl[g] pH 7.5	6.27	6.41	6.12	6.27 ± 0.15	5.91	6.08	5.98	5.99 ± 0.09
	Moderately alkaline environment 8 ≤ pH ≤ 8.6								
8	0.1 M Sodium phosphate ($HPO_4^{2-}/H_2PO_4^-$) pH 8	7.25	7.06	6.96	7.09 ± 0.15	6.58	6.37	6.36	6.44 ± 0.12
	0.1 M HEPES pH 8	7.44	7.57	7.06	7.36 ± 0.27	6.68	6.60	6.50	6.59 ± 0.09
	0.1 M tris-HCl pH 8	6.66	6.93	6.60	6.73 ± 0.18	6.10	6.27	6.21	6.19 ± 0.09
8.5	0.1 M tris-HCl pH 8.5	7.76	8.00	7.80	7.85 ± 0.13	6.58	6.41	6.35	6.45 ± 0.12
8.6	0.1 M glycine-NaOH pH 8.6	6.56	6.41	5.89	6.29 ± 0.35	6.03	6.11	5.84	5.99 ± 0.14
	0.2 M glycine-NaOH pH 8.6	7.06	6.89	6.36	6.77 ± 0.36	6.27	6.28	6.03	6.19 ± 0.14
	0.5 M glycine-NaOH pH 8.6	7.94	7.52	6.80	7.42 ± 0.57	6.45	6.40	6.26	6.37 ± 0.10

[a]SD – standard deviation; [b]MES – 2-(N-morpholino)ethanesulfonic acid, $C_6H_{13}NO_4S$ (Figure S1); [c]HEPES – 4-(2-hydroxyethyl)-1-piperazineethanesulfonic acid, $C_8H_{18}N_2O_4S$ (Figure S1); [d]PBS – phosphate buffer saline; [e]MHB – Mueller-Hinton broth, for more detailed information about composition, see https://labmal.com/2019/11/20/mueller-hinton-agar-and-mueller-hinton-broth/; [f]Nutrient mixture F-12 Ham contains sodium pyruvate (0.11 g/L), phenol red, L-glutamine, and does not contain $NaHCO_3$ and HEPES, for more details please see https://www.sigmaaldrich.com/AT/en/technical-documents/technical-article/cell-culture-and-cell-culture-analysis/mammalian-cell-culture/f-12-ham; [g]tris – tris(hydroxymethyl)aminomethane, $C_4H_{11}NO_3$ (Figure S1).

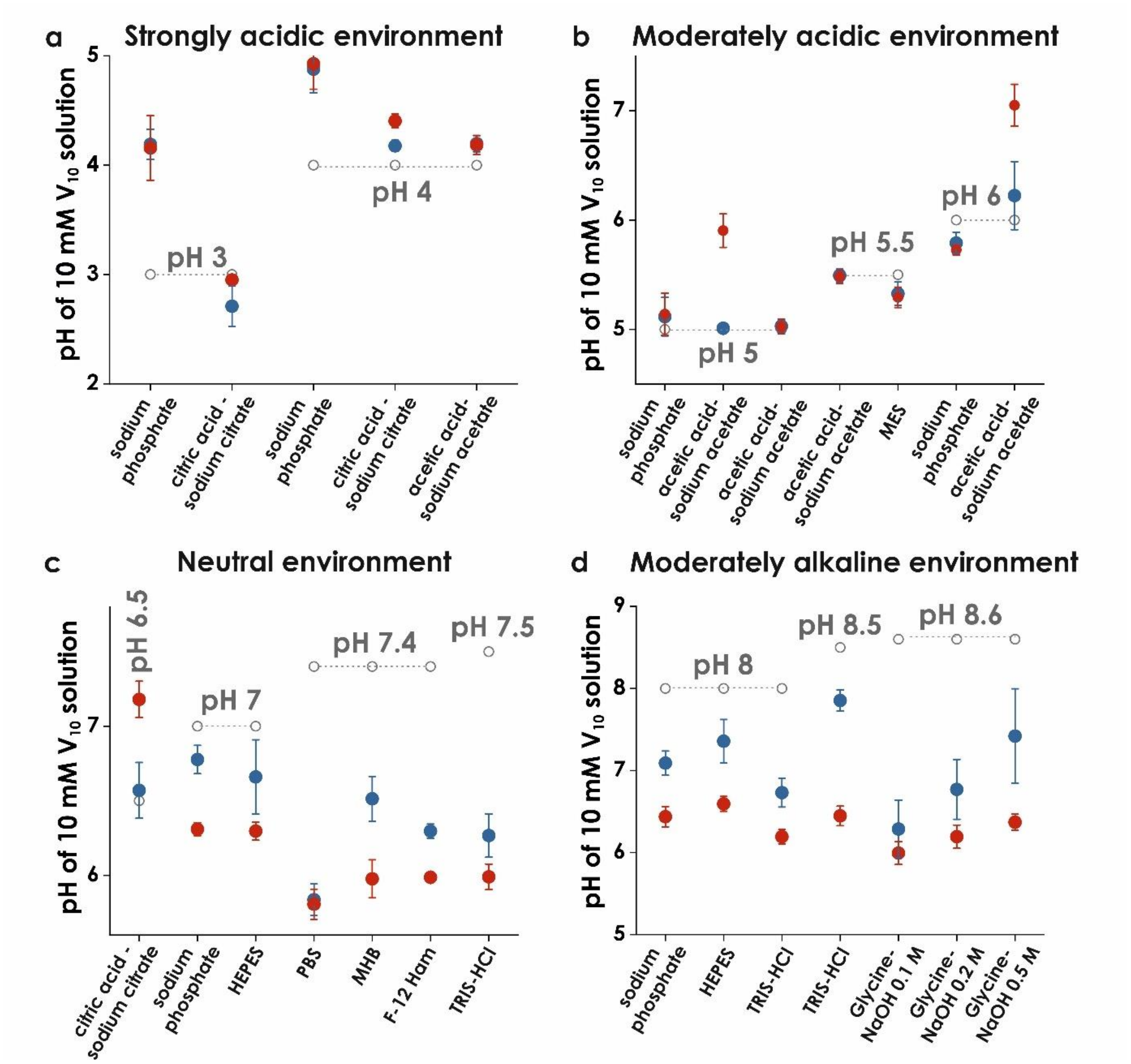

Fig. S132. pH in V_{10} solutions.

The pH dependence curves for solutions of **$Na_2K_4[V^V{}_{10}O_{28}]$** in different buffers in a) strongly acidic, b) moderately acidic, c) neutra, and d) moderately alkaline environment. The pH of the starting buffers is shown with a gray dashed line; the plots of the measured pH values immediately after preparation of the solutions are shown in blue and after 24 h incubation at 37 °C in red. The error bar shows standard deviation (**Table S26**).

12.2. ^{51}V NMR spectroscopic studies of decavanadate solutions

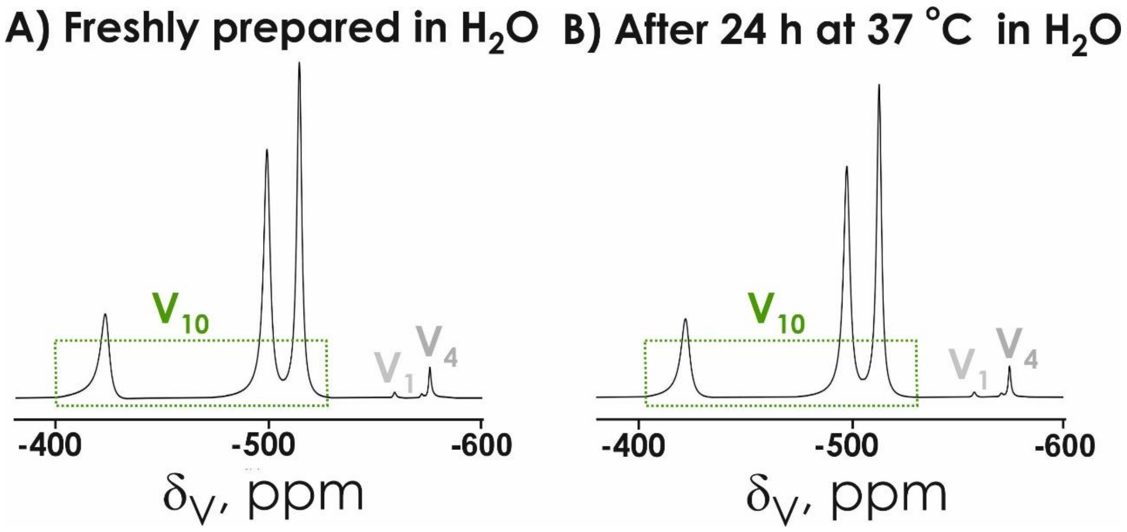

Fig. S133. ^{51}V NMR spectra of V_{10} in H_2O.

^{51}V NMR spectra for 10 mM solutions of **$Na_2K_4[V^V{}_{10}O_{28}]$** in D_2O that were recorded approximately one hour after preparation (**A**) and after incubation for 24 h at 37 °C (**B**). The chemical shifts and percentages of parent and formed species are given in **Tables S27** and **S28**. The structures of all POMs are shown in **Figure S131**. To identify the individual anions, they are shown in different colors, with the same color code being selected for a specific anion throughout all figures and tables in the main manuscript and the supporting information.

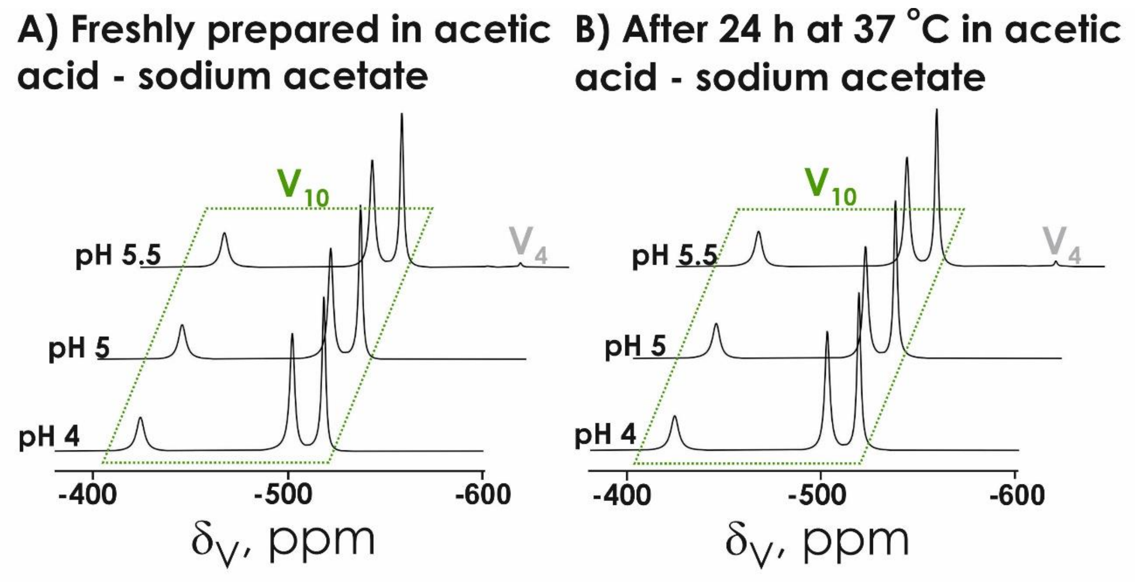

Fig. S134. ^{51}V NMR spectra of V_{10} in NaOAc/HOAc buffer.

^{51}V NMR spectra for 10 mM solutions of **$Na_2K_4[V^V{}_{10}O_{28}]$** in 0.1 M NaOAc/HOAc buffer (pH 4 – 5.5) that were recorded approximately one hour after preparation (**A**) and after incubation for 24 h at 37 °C (**B**). The chemical shifts and percentages of parent and formed species are given in **Tables S27** and **S28**. The structures of all POMs are shown in **Figure S131**. To identify the individual anions, they are shown in different colors, with the same color code being selected for a specific anion throughout all figures and tables in the main manuscript and the supporting information.

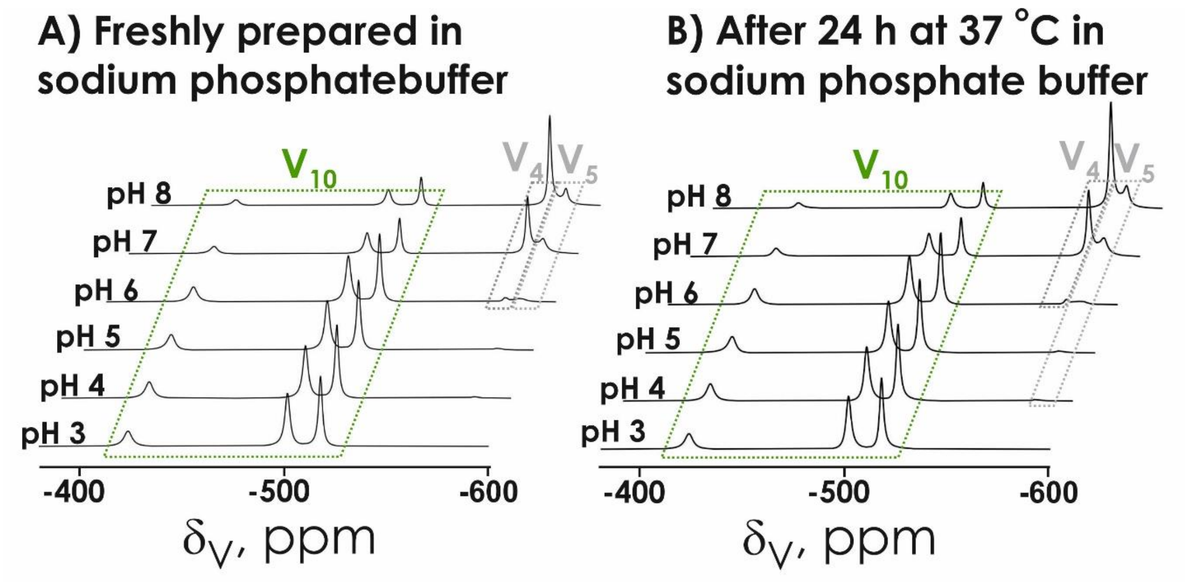

Fig. S135. [51]V NMR spectra of V_{10} in sodium phosphate buffer.

[51]V NMR spectra for 10 mM solutions of **$Na_2K_4[V^V_{10}O_{28}]$** in 0.1 M sodium phosphate buffer (pH 3 – 8) that were recorded approximately one hour after preparation (**A**) and after incubation for 24 h at 37 °C (**B**). The chemical shifts and percentages of parent and formed species are given in **Tables S27** and **S28**. The structures of all POMs are shown in **Figure S131**. To identify the individual anions, they are shown in different colors, with the same color code being selected for a specific anion throughout all figures and tables in the main manuscript and the supporting information.

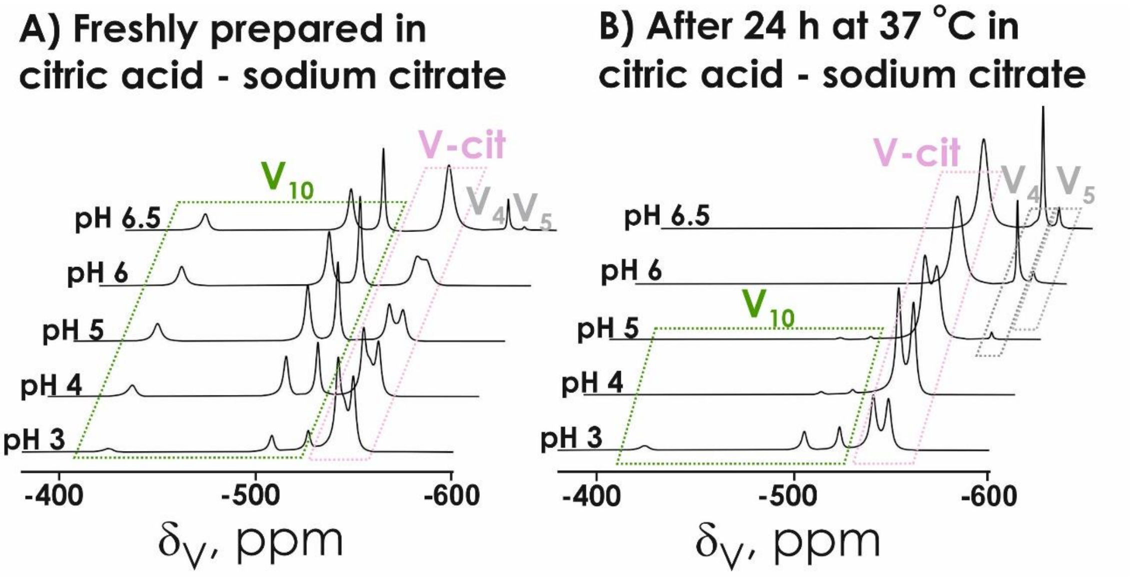

Fig. S136. ^{51}V NMR spectra of V_{10} in citric acid – sodium citrate buffer.

^{51}V NMR spectra for 10 mM solutions of **$Na_2K_4[V^V{}_{10}O_{28}]$** 0.1 M in citric acid – sodium citrate buffer (pH 3 – 6.5) that were recorded approximately one hour after preparation (**A**) and after incubation for 24 h at 37 °C (**B**). The chemical shifts and percentages of parent and formed species are given in **Tables S27** and **S28**. The structures of all POMs are shown in **Figure S131**. To identify the individual anions, they are shown in different colors, with the same color code being selected for a specific anion throughout all figures and tables in the main manuscript and the supporting information.

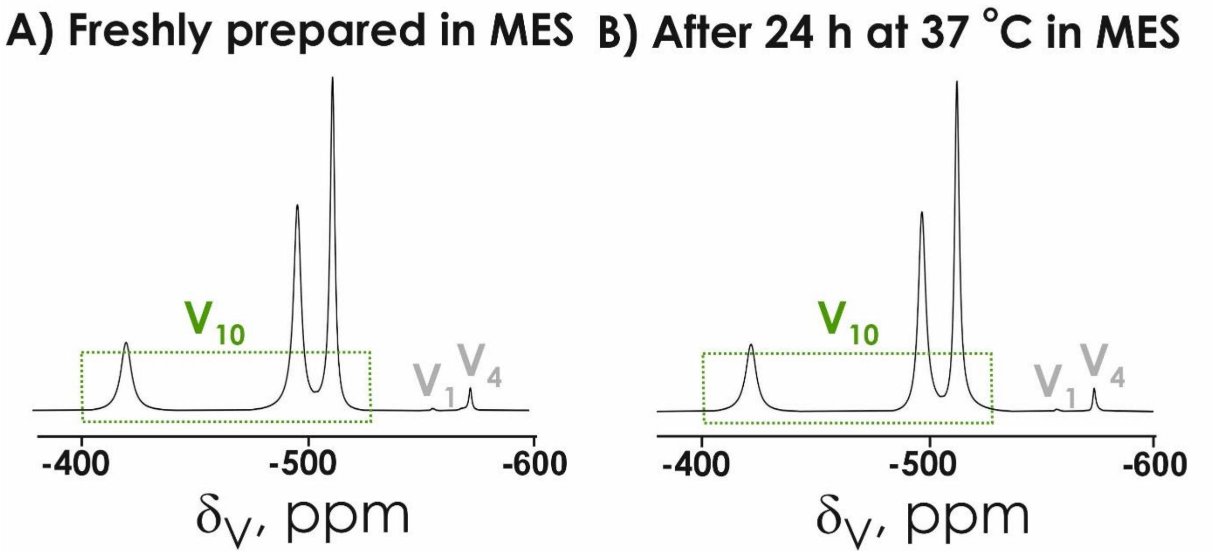

Fig. S137. ^{51}V NMR spectra of V_{10} in MES buffer.

^{51}V NMR spectra for 10 mM solutions of **$Na_2K_4[V^V_{10}O_{28}]$** in 0.1 M MES buffer pH 5.5 that were recorded approximately one hour after preparation (**A**) and after incubation for 24 h at 37 °C (**B**). The chemical shifts and percentages of parent and formed species are given in **Tables S27** and **S28**. The structures of all POMs are shown in **Figure S131**. To identify the individual anions, they are shown in different colors, with the same color code being selected for a specific anion throughout all figures and tables in the main manuscript and the supporting information.

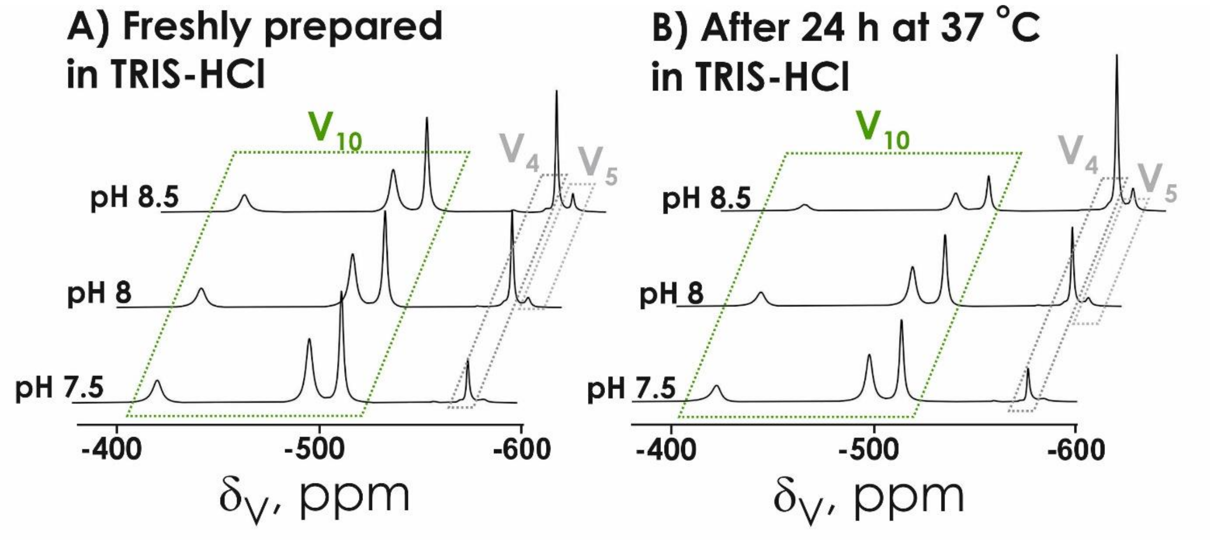

Fig. S138. ^{51}V NMR spectra of V_{10} in tris-HCl buffer.

^{51}V NMR spectra for 10 mM solutions of **$Na_2K_4[V^V_{10}O_{28}]$** in 0.1 M tris-HCl buffer (pH 7.5 – 8.5) that were recorded approximately one hour after preparation (**A**) and after incubation for 24 h at 37 °C (**B**). The chemical shifts and percentages of parent and formed species are given in **Tables S27** and **S28**. The structures of all POMs are shown in **Figure S131**. To identify the individual anions, they are shown in different colors, with the same color code being selected for a specific anion throughout all figures and tables in the main manuscript and the supporting information.

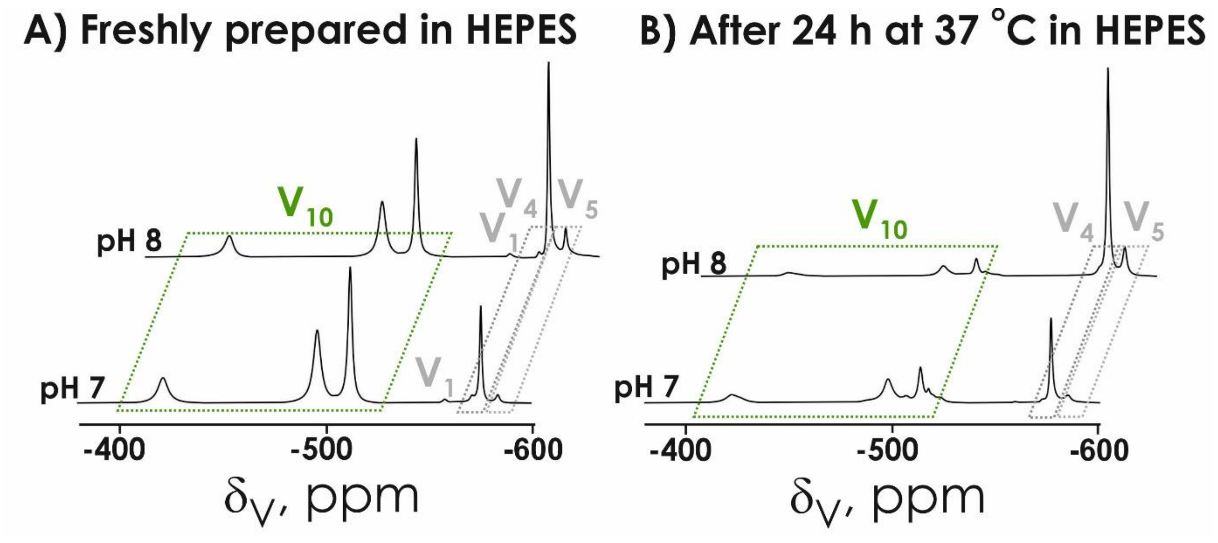

Fig. S139. [51]V NMR spectra of V_{10} in HEPES buffer.

[51]V NMR spectra for 10 mM solutions of **$Na_2K_4[V^V_{10}O_{28}]$** in 0.1 M HEPES buffer (pH 7 and 8) that were recorded approximately one hour after preparation (**A**) and after incubation for 24 h at 37 °C (**B**). The chemical shifts and percentages of parent and formed species are given in **Tables S27** and **S28**. The structures of all POMs are shown in **Figure S131**. To identify the individual anions, they are shown in different colors, with the same color code being selected for a specific anion throughout all figures and tables in the main manuscript and the supporting information.

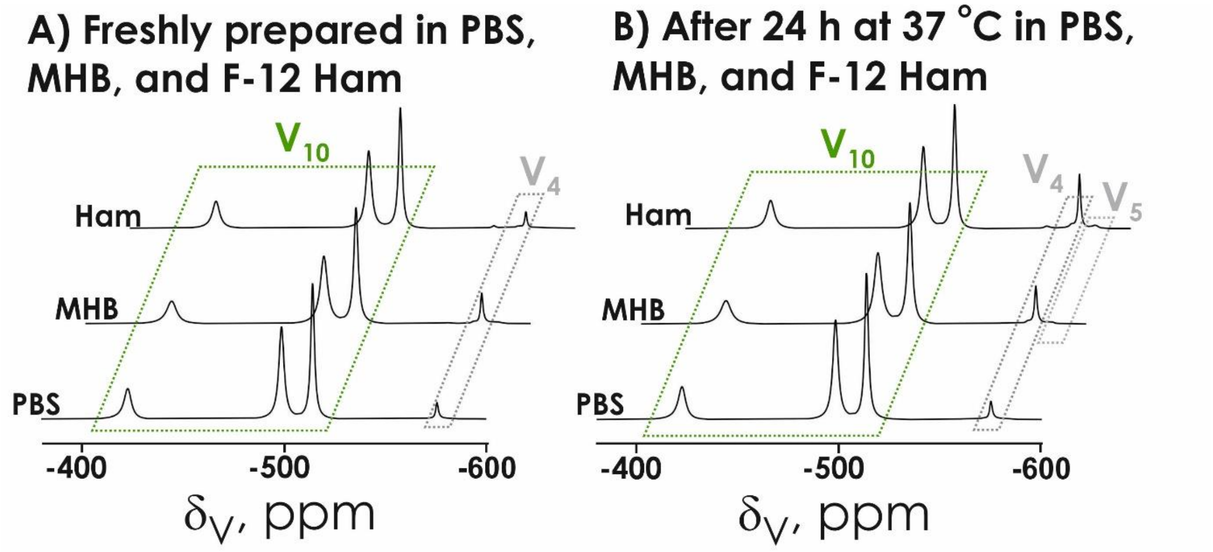

Fig. S140. ^{51}V NMR spectra of V_{10} in solutions with pH 7.4.

^{51}V NMR spectra for 10 mM solutions of **$Na_2K_4[V^V_{10}O_{28}]$** in 0.1 M PBS, MHB and F-12 Ham (pH 7.4) that were recorded approximately one hour after preparation (**A**) and after incubation for 24 h at 37 °C (**B**). The chemical shifts and percentages of parent and formed species are given in **Tables S27** and **S28**. The structures of all POMs are shown in **Figure S131**. To identify the individual anions, they are shown in different colors, with the same color code being selected for a specific anion throughout all figures and tables in the main manuscript and the supporting information.

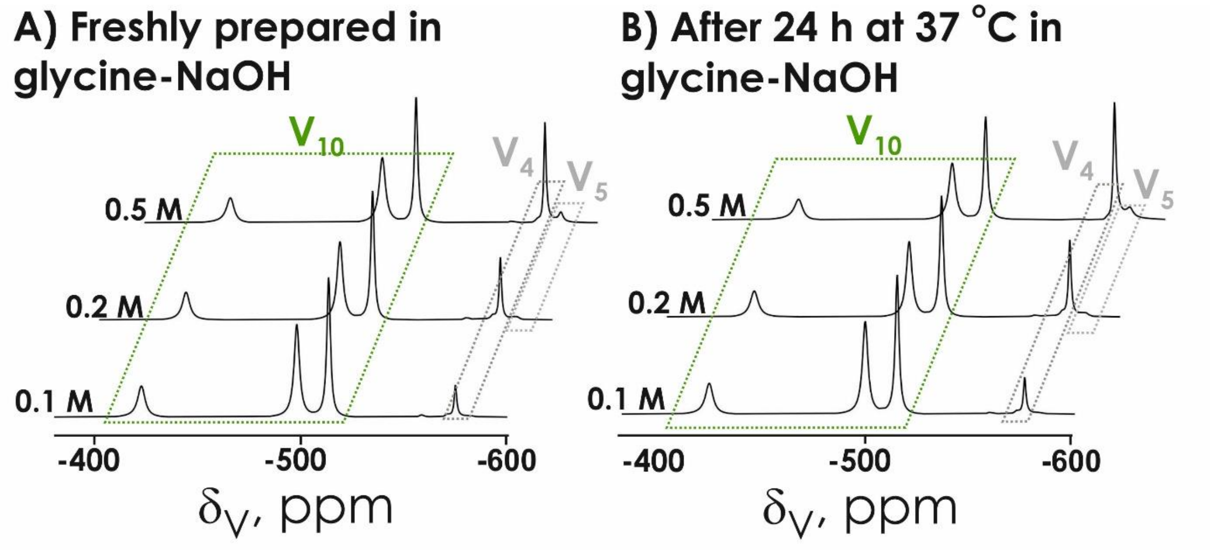

Fig. S141. ^{51}V NMR spectra of V_{10} in glycine-NaOH buffer.

^{51}V NMR spectra for 10 mM solutions of **$Na_2K_4[V^V_{10}O_{28}]$** in glycine-NaOH (pH 8.6) with concentration 0.1, 0.2 and 0.5 M that were recorded approximately one hour after preparation (**A**) and after incubation for 24 h at 37 °C (**B**). The chemical shifts and percentages of parent and formed species are given in **Tables S27** and **S28**. The structures of all POMs are shown in **Figure S131**. To identify the individual anions, they are shown in different colors, with the same color code being selected for a specific anion throughout all figures and tables in the main manuscript and the supporting information.

Table S27. Analysis of NMR spectroscopic data recorded in V_{10} solutions at room temperature.

Chemical shifts in ^{51}V NMR spectra measured in triplicate of **$Na_2K_4[V^V{}_{10}O_{28}]$** (10 mM) dissolved D_2O and 0.1 M buffers (acetic acid – sodium acetate pH 4 – 5.5; sodium phosphate pH 3 – 8 (while phosphate does not buffer at pH range from 3.5 – 5.5, experiments were conducted at this pH to provide comparisons to previously published studies (*29*)); citric acid – sodium citrate pH 3 – 6.5; MES pH 5.5; PBS pH 7.4; tris-HCl pH 7.5 – 8.5; HEPES pH 7 – 8; MHB pH 7.4; Nutrient mixture F-12 Ham pH 7.4 and glycine-NaOH pH 8.6) with concentration 0.1 M and investigated after 24 h incubation at 37 °C (**Figures S133 – S141**). The glycine-NaOH buffer was used in two additional concentrations of 0.2 and 0.5 M. The content of species was calculated based on the integration of ^{51}V signals. Signals were assigned based on the literature data summarized in **Table S4**.

pH	**$Na_2K_4[V_{10}O_{28}]$** (10 mM) in Solvent / Buffer / Medium	Chemical shifts δ ^{51}V [ppm]	% of V_{10} in 10 mM **$Na_2K_4[V^V{}_{10}O_{28}]$** at RT				% of Σ (V_1+V_2+V_4+V_5) in 10 mM **$Na_2K_4[V^V{}_{10}O_{28}]_0$** at RT				% of Σ V_{cit} in 10 mM **$Na_2K_4[V^V{}_{10}O_{28}]$** at RT			
			Sample			Mean of 1 to 3 ± SD[a]	Sample			Mean of 1 to 3 ± SD[a]	Sample			Mean of 1 to 3 ± SD[a]
			#1	#2	#3		#1	#2	#3		#1	#2	#3	
-	D_2O	**–422.7**, **–498.7**, **–511.0**, –558.9, –571.6, –575.5	99	95	99	98 +2	0	4	0	1 ± 2	0	0	0	0
Strongly acidic environment 3 ≤ pH ≤ 4														
3	0.1 M Sodium phosphate ($H_2PO_4^-/H_3PO_4$) pH 3	**–423.4**, **–501.4**, **–517.6**, –578.3	99	99	99	99 ± 0	0	0	1	0 ± 1	0	0	0	0
	0.1 M Citric acid – sodium citrate (H_3Cit/H_2Cit^-) pH 3	**–424.5**, **–507.1**, **–525.6**, –540.8, –548.5	18	19	17	18 ± 1	0	0	0	0	82	81	83	82 ± 1
4	0.1 M Sodium phosphate ($H_2PO_4^-/H_3PO_4$) pH 4	**–422.8**, **–499.3**, **–514.6**, –582.2	99	99	99	99 ± 0	0	1	1	1 ± 1	0	0	0	0
	0.1 M Citric acid – sodium citrate ($H_2Cit^-/HCit^{2-}$) pH 4	**–423.2**, **–501.2**, **–517.4**, –540.6, –543.8, –548.2	52	46	49	49 ± 3	0	0	0	0	48	54	51	51 ± 3
	0.1 M Acetic acid – sodium acetate (OAc^-/HOAc) pH 4	**–423.4**, **–501.6**, **–517.8**	100	100	100	100 ± 0	0	0	0	0	0	0	0	0
Moderately acidic environment 5 ≤ pH ≤ 6														
5	0.1 M Sodium phosphate ($H_2PO_4^-/H_3PO_4$) pH 5	**–422.6**, **–499.0**, **–514.2**, –575.3, –582.4	98	98	99	98 ± 1	0	2	1	1 ± 1	0	0	0	0
	0.1 M Citric acid – sodium citrate ($H_2Cit^-/HCit^{2-}$) pH 5	**–422.8**, **–499.1**, **–514.4**, –525.4, –540.4, –547.6	76	66	75	72 ± 6	0	0	0	0	24	34	25	28 ± 6
	0.1 M Acetic acid – sodium acetate (OAc^-/HOAc) pH 5	**–413.9**, **–499.3**, **–518.5**, –557.8, –574.9	100	100	100	100 ± 0	0	0	0	0	0	0	0	0
5.5	0.1 M Acetic acid – sodium acetate (OAc^-/HOAc) pH 5.5	**–422.6**, **–498.6**, **–513.9**, –558.1, –574.8	99	99	99	99 ± 0	1	1	1	1 ± 1	0	0	0	0
	0.1 M MES[b] pH 5.5	**–422.3**, **–498.0**, **–513.5**, –557.8, –570.6, –574.3	97	97	98	97 ± 1	2	2	2	2 ± 0	0	0	0	0

6	0.1 M Sodium phosphate ($HPO_4^{2-}/H_2PO_4^{-}$) pH 6	**–422.5, –498.3, –513.6**, –575.1, –582.8	92	90	94	92 ± 2	8	10	6	8 ± 2	0	0	0	0
	0.1 M Citric acid – sodium citrate ($HCit^{2-}/Cit^{3-}$) pH 6	**–421.9, –496.9, –512.6**, –541.2, –546.8	71	66	77	71 ± 6	0	0	0	0	29	34	23	29 ± 6
Neutral environment 6.5 ≤ pH ≤ 7.5														
6.5	0.1 M Citric acid – sodium citrate ($HCit^{2-}/Cit^{3-}$) pH 6.5	**–420.9, –494.8, –511.3**, –544.5, –570.5, –574.7, –582.8	60	55	62	59 ± 4	5	8	3	5 ± 3	35	37	36	36 ± 1
7	0.1 M Sodium phosphate ($HPO_4^{2-}/H_2PO_4^{-}$) pH 7	**–421.7, –496.5, –512.4**, –574.9, –582.8	63	52	61	59 ± 6	37	48	39	41 ± 6	0	0	0	0
	0.1 M HEPES[c] pH 7	**–421.2, –496.1, –512.0**, –557.8, –571.0, –575.3, –583.6	77	80	74	77 ± 3	23	20	26	23 ± 3	0	0	0	0
7.4	PBS[d] pH 7.4	**–422.3, –498.1, –513.5**, –574.8	93	96	95	95 ± 2	7	4	4	5 ± 2	0	0	0	0
	MHB[e] pH 7.4	**–422.2, –497.1, –512.9**, –559.0, –571.0, –574.9, –582.7	95	90	93	93 ± 3	5	9	6	7 ± 2	0	0	0	0
	Nutrient mixture F-12 Ham[f]	**–422.0, –497.3, –512.9**, –559.0, –570.8, –574.8, –582.8	95	94	94	94 ± 1	5	5	5	5	0	0	0	0
7.5	0.1 M tris-HCl[g] pH 7.5	**–421.9, –496.9, –512.7**, –558.2, –571.1, –575.0, –582.9	90	86	91	89 ± 3	9	13	8	10 ± 3	0	0	0	0
Moderately alkaline environment 8 ≤ pH ≤ 8.6														
8	0.1 M Sodium phosphate ($HPO_4^{2-}/H_2PO_4^{-}$) pH 8	**–421.4, –495.9, –512.0**, –574.8, –582.8	52	55	55	54 ± 2	48	48	45	47 ± 2	0	0	0	0
	0.1 M HEPES pH 8	**–420.7, –494.5, –511.0**, –556.4, –570.5, –575.1, –583.4, –593.5	41	44	40	42 ± 2	59	56	60	58 ± 2	0	0	0	0
	0.1 M tris-HCl pH 8	**–421.8, –496.4, –512.4**, –558.5, –570.9, –575.0, –582.9	81	76	79	79 ± 3	18	24	21	21 ± 3	0	0	0	0
8.5	0.1 M tris-HCl pH 8.5	**–421.1, –494.4, –511.0**, –553.6, –569.7, –575.0, –583.0	60	63	66	63 ± 3	40	36	34	37 ± 3	0	0	0	0

8.6	0.1 M glycine-NaOH pH 8.6	**–422.3**, **–497.8**, **–513.2**, –558.4, –570.9, –574.8, –582.6	93	92	95	93 ± 2	6	7	4	6 ± 2	0	0	0	0
	0.2 M glycine-NaOH pH 8.6	**–422.0**, **–496.8**, **–512.6**, –558.4, –570.9, –574.6, –582.4	88	84	84	85 ± 2	12	15	16	14 ± 2	0	0	0	0
	0.5 M glycine-NaOH pH 8.6	**–421.6**, **–495.3**, **–511.7**, –558.3, –570.6, –574.2, –582.0	77	75	71	74 ± 3	23	24	29	25 ± 3	0	0	0	0

[a]SD – standard deviation; [b]MES – 2-(N-morpholino)ethanesulfonic acid, $C_6H_{13}NO_4S$ (Figure S1); [c]HEPES – 4-(2-hydroxyethyl)-1-piperazineethanesulfonic acid, $C_8H_{18}N_2O_4S$ (Figure S1); [d]PBS – phosphate buffer saline; [e]MHB – Mueller-Hinton broth, for more detailed information about composition, see https://labmal.com/2019/11/20/mueller-hinton-agar-and-mueller-hinton-broth/; [f]Nutrient mixture F-12 Ham contains sodium pyruvate (0.11 g/L), phenol red, L-glutamine, and does not contain $NaHCO_3$ and HEPES, for more details please see https://www.sigmaaldrich.com/AT/en/technical-documents/technical-article/cell-culture-and-cell-culture-analysis/mammalian-cell-culture/f-12-ham; [g]tris – tris(hydroxymethyl)aminomethane, $C_4H_{11}NO_3$ (Figure S1).

Table S28. Analysis of NMR spectroscopic data of V_{10} solutions investigated after 24 h incubation at 37 °C

Chemical shifts in ^{51}V NMR spectra measured in triplicate of **$Na_2K_4[V^V{}_{10}O_{28}]$** (10 mM) dissolved D_2O and 0.1 M buffers (acetic acid – sodium acetate pH 4 – 5.5; sodium phosphate pH 3 – 8 (while phosphate does not buffer at pH range from 3.5 – 5.5, experiments were conducted at this pH to provide comparisons to previously published studies (*29*)); citric acid – sodium citrate pH 3 – 6.5; MES pH 5.5; PBS pH 7.4; tris-HCl pH 7.5 – 8.5; HEPES pH 7 – 8; MHB pH 7.4; Nutrient mixture F-12 Ham pH 7.4 and glycine-NaOH pH 8.6) and investigated after 24 h incubation at 37 °C (**Figures S133 – S141**). The glycine-NaOH buffer was used in two additional concentrations of 0.2 and 0.5 M. The content of species was calculated based on the integration of ^{51}V signals. Signals were assigned based on the literature data summarized in **Table S4**.

pH	**$Na_2K_4[V_{10}O_{28}]$** (10 mM) in Solvent / Buffer / Medium	Chemical shifts δ ^{31}P [ppm]	% of V_{10} in 10 mM **$Na_2K_4[V^V{}_{10}O_{28}]$** solution after 24 h incubation at 37 °C				% of Σ (V_1+V_2+V_4+V_5) in 10 mM **$Na_2K_4[V^V{}_{10}O_{28}]$** solution after 24 h incubation at 37 °C				% of Σ V_{cit} in 10 mM **$Na_2K_4[V^V{}_{10}O_{28}]$** solution after 24 h incubation at 37 °C			
			Sample			Mean of 1 to 3 ± SD[a]	Sample			Mean of 1 to 3 ± SD[a]	Sample			Mean of 1 to 3 ± SD[a]
			#1	#2	#3		#1	#2	#3		#1	#2	#3	
-	D_2O	**–422.8**, **–498.7**, **–513.9**, –558.9, –571.6, –575.5	99	96	98	98 ± 2	0	3	2	2 ± 2	0	0	0	0
Strongly acidic environment 3 ≤ pH ≤ 4														
3	0.1 M Sodium phosphate ($H_2PO_4^-$/H_3PO_4) pH 3	**–423.3**, **–501.4**, **–517.5**, –578.4	95	99	99	98 ± 2	0	0	1	0 ± 1	0	0	0	0
	0.1 M Citric acid – sodium citrate (H_3Cit/H_2Cit^-) pH 3	**–424.3**, **–505.6**, **–523.6**, –540.8, –548.5	25	29	29	28 ± 2	0	0	0	0	74	71	71	72 ± 2
4	0.1 M Sodium phosphate ($H_2PO_4^-$/H_3PO_4) pH 4	**–422.8**, **–499.3**, **–514.6**, –582.3	97	99	99	98 ± 1	0	1	1	1 ± 1	0	0	0	0
	0.1 M Citric acid – sodium citrate (H_2Cit^-/$HCit^{2-}$) pH 4	**–423.3**, **–501.1**, **–517.1**, –540.5, –548.2	0	4	0	1 ± 2	0	0	0	0	99	96	98	98 ± 2
	0.1 M Acetic acid – sodium acetate (OAc^-/HOAc) pH 4	**–423.4**, **–501.6**, **–517.8**	100	100	100	100 ± 0	0	0	0	0	0	0	0	0
Moderately acidic environment 5 ≤ pH ≤ 6														
5	0.1 M Sodium phosphate ($H_2PO_4^-$/H_3PO_4) pH 5	**–422.6**, **–499.0**, **–514.2**, –575.3, –582.5	98	98	99	98 ± 1	0	2	1	1 ± 1	0	0	0	0

	0.1 M Citric acid – sodium citrate (H_2Cit^-/$HCit^{2-}$) pH 5	**–422.0**, **–497.6**, **–513.1**, –540.6, –547.1, –558.7, –570.9, –574.7	2	2	2	2 ± 0	3	2	1	2 ± 1	95	95	97	96 ± 2
	0.1 M Acetic acid – sodium acetate (OAc^-/HOAc) pH 5	**–426.9**, **–487.9**, **–512.7**, –557.8, –574.9	100	98	100	99 ± 1	0	2	0	1 ± 1	0	0	0	0
5.5	0.1 M Acetic acid – sodium acetate (OAc^-/HOAc) pH 5.5	**–422.5**, **–498.6**, **–513.8,** –558.1, –574.8	99	98	99	99 ± 1	0	2	0	1 ± 1	0	0	0	0
	0.1 M MES[b] pH 5.5	**–422.2**, **–498.0**, **–513.5**, –557.9, –574.4	97	98	98	98 ± 1	3	2	2	2 ± 1	0	0	0	0
6	0.1 M Sodium phosphate (HPO_4^{2-}/$H_2PO_4^-$) pH 6	**–422.5**, **–498.3**, **–513.6**, –575.0, –582.6	92	92	94	93 ± 1	8	8	6	7 ± 1	0	0	0	0
	0.1 M Citric acid – sodium citrate ($HCit^{2-}$/Cit^{3-}) pH 6	–544.1, –570.7, –574.7, –582.8, –594.6	0	0	0	0	24	21	21	22 ± 2	76	78	79	78 ± 2
Neutral environment 6.5 ≤ pH ≤ 7.5														
6.5	0.1 M Citric acid – sodium citrate ($HCit^{2-}$/Cit^{3-}) pH 6.5	–544.3, –570.5, –574.7, –582.8	0	0	0	0	26	35	32	31 ± 5	64	65	68	66 ± 2
7	0.1 M Sodium phosphate (HPO_4^{2-}/$H_2PO_4^-$) pH 7	**–422.0**, **–496.8**, **–512.5**, –574.9, –582.7	43	42	50	45 ± 4	57	58	50	55 ± 4	0	0	0	0
	0.1 M HEPES[c] pH 7	**–421.5**, **–425.8**, **–496.8**, **–505.7**, **–512.5**, **–516.4**, **–518.6**, **–522.1**, –558.2, –571.1, –575.4, –583.6	49	61	73	61 ± 12	42	39	27	36 ± 8	0	0	0	0
7.4	PBS[d] pH 7.4	**–422.4**, **–498.1**, **–513.5**, –574.8	91	96	91	93 ± 3	9	4	8	7 ± 3	0	0	0	0
	MHB[e] pH 7.4	**–422.3**, **–497.2**, **–513.0**, –559.0, –571.1, –575.1, –582.6	86	89	94	90 ± 4	14	11	5	10 ± 5	0	0	0	0
	Nutrient mixture F-12 Ham[f]	**–422.2**, **–497.7**, **–513.1**, –558.7, –570.9, –574.8, –582.8	86	85	81	84 ± 3	14	14	19	16 ± 3	0	0	0	0

7.5	0.1 M tris-HCl[g] pH 7.5	**–421.9**, **–496.9**, **–512.7**, –558.2, –571.1, –575.0, –583.0	87	87	91	88 ± 2	13	12	8	11 ± 3	0	0	0	0
Moderately alkaline environment 8 ≤ pH ≤ 8.6															
	0.1 M Sodium phosphate (HPO_4^{2-}/$H_2PO_4^{-}$) pH 8	**–421.9**, **–496.5**, **–512.4**, –574.9, –582.7	35	30	33	33 ± 3	65	70	67	67 ± 3	0	0	0	0
8	0.1 M HEPES pH 8	**–421.2**, **–496.1**, **–512.0**, –558.3, –571.2, –575.5, –583.6	25	31	21	26 ± 5	74	69	79	74 ± 5	0	0	0	0
	0.1 M tris-HCl pH 8	**–421.7**, **–496.3**, **–512.4**, –558.2, –570.9, –575.0, –582.9	75	69	77	74 ± 4	25	31	22	26 ± 5	0	0	0	0
8.5	0.1 M tris-HCl pH 8.5	**–421.5**, **–495.7**, **–511.9**, –558.1, –570.9, –575.0, –583.0	34	39	48	40 ± 7	66	61	51	59 ± 8	0	0	0	0
	0.1 M glycine-NaOH pH 8.6	**–422.2**, **–497.8**, **–513.2**, –558.4, –571.1, –574.9, –582.5	91	91	95	92 ± 2	8	8	4	7 ± 2	0	0	0	0
8.6	0.2 M glycine-NaOH pH 8.6	**–422.0**, **–497.1**, **–512.8**, –558.5, –571.2, –574.9, –582.5	80	79	86	82 ± 4	20	20	13	18 ± 4	0	0	0	0
	0.5 M glycine-NaOH pH 8.6	**–421.9**, **–496.1**, **–512.2**, –558.5, –574.5, –582.0	67	68	70	68 ± 2	33	31	30	31 ± 2	0	0	0	0

[a]SD – standard deviation; [b]MES – 2-(N-morpholino)ethanesulfonic acid, $C_6H_{13}NO_4S$ (Figure S1); [c]HEPES – 4-(2-hydroxyethyl)-1-piperazineethanesulfonic acid, $C_8H_{18}N_2O_4S$ (Figure S1); [d]PBS – phosphate buffer saline; [e]MHB – Mueller-Hinton broth, for more detailed information about composition, see https://labmal.com/2019/11/20/mueller-hinton-agar-and-mueller-hinton-broth/; [f]Nutrient mixture F-12 Ham contains sodium pyruvate (0.11 g/L), phenol red, L-glutamine, and does not contain $NaHCO_3$ and HEPES, for more details please see https://www.sigmaaldrich.com/AT/en/technical-documents/technical-article/cell-culture-and-cell-culture-analysis/mammalian-cell-culture/f-12-ham; [g]tris – tris(hydroxymethyl)aminomethane, $C_4H_{11}NO_3$ (Figure S1).

12.3. Speciation in decavanadate solutions

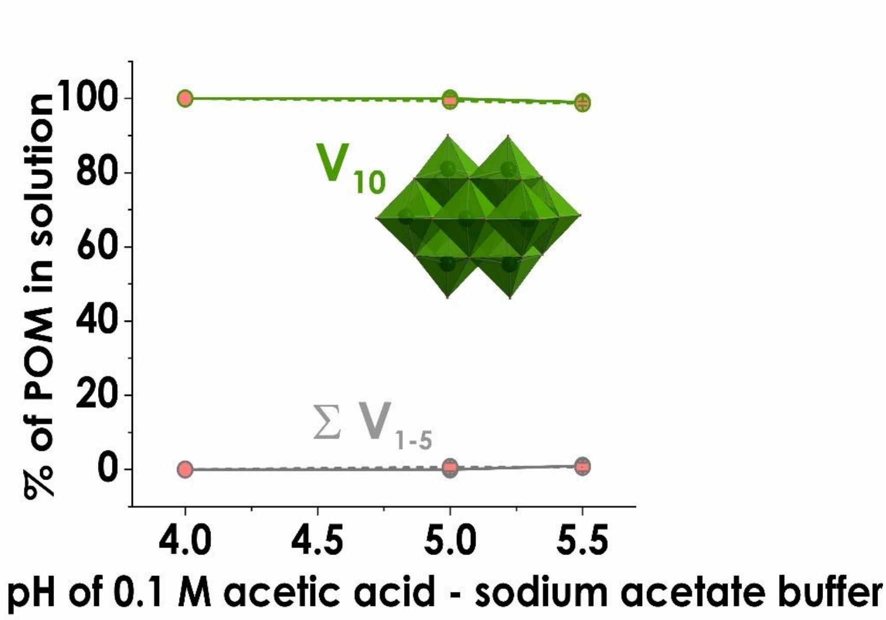

Fig. S142. Speciation of V_{10} in acetic acid – sodium acetate buffer.

POM concentration curves in **$Na_2K_4[V^V{}_{10}O_{28}]$** (10 mM) in 0.1 M acetic acid – sodium acetate buffer solutions before (solid line, blue dot in the middle) and after incubation (dash line, red dot in the middle) for 24 h at 37 °C. The exact percentage of all POM species present is given in **Tables S27** and **S28**.

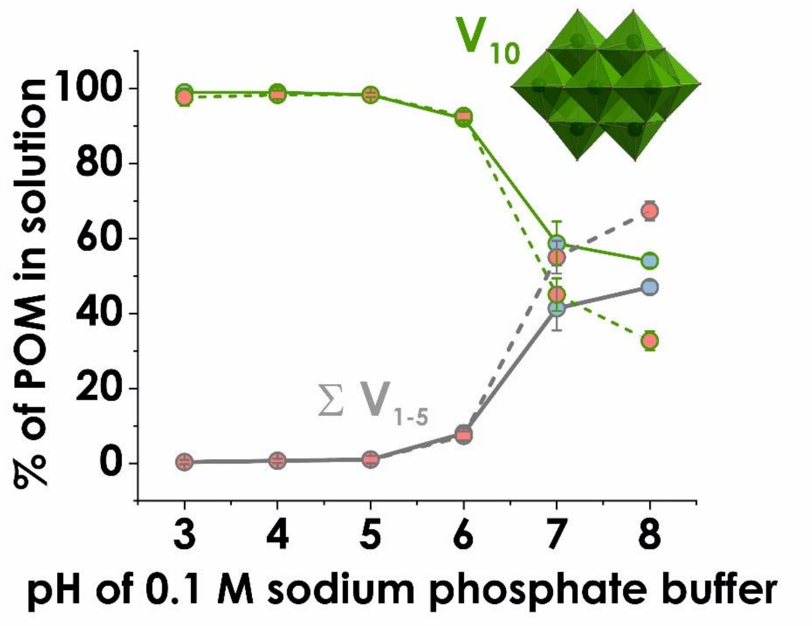

Fig. S143. Speciation of V_{10} in sodium phosphate buffer.

POM concentration curves in **$Na_2K_4[V^V{}_{10}O_{28}]$** (10 mM) in 0.1 M sodium phosphate buffer solutions before (solid line, blue dot in the middle) and after incubation (dash line, red dot in the middle) for 24 h at 37 °C. The exact percentage of all POM species present is given in **Tables S27** and **S28**.

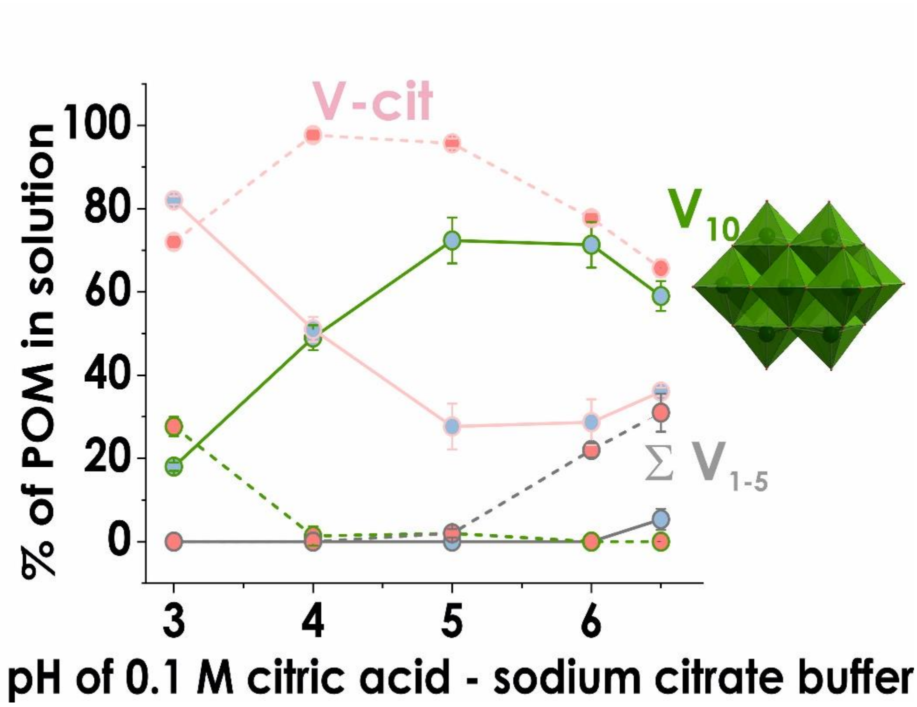

Fig. S144. Speciation of V_{10} in citric acid – sodium citrate buffer.

POM concentration curves in **$Na_2K_4[V^V_{10}O_{28}]$** (10 mM) in 0.1M citric acid – sodium citrate buffer solutions before (solid line, blue dot in the middle) and after incubation (dash line, red dot in the middle) for 24 h at 37 °C. The exact percentage of all POM species present is given in **Tables S27** and **S28**.

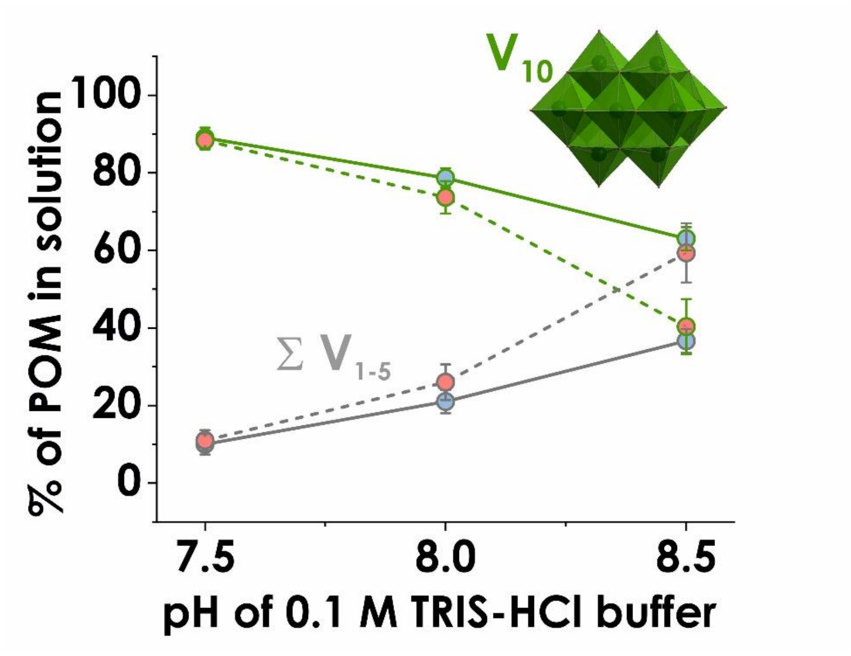

Fig. S145. Speciation of V_{10} in tris-HCl buffer.

POM concentration curves in **$Na_2K_4[V^V{}_{10}O_{28}]$** (10 mM) in 0.1 M tris-HCl buffer solutions before (solid line, blue dot in the middle) and after incubation (dash line, red dot in the middle) for 24 h at 37 °C. The exact percentage of all POM species present is given in **Tables S27** and **S28**.

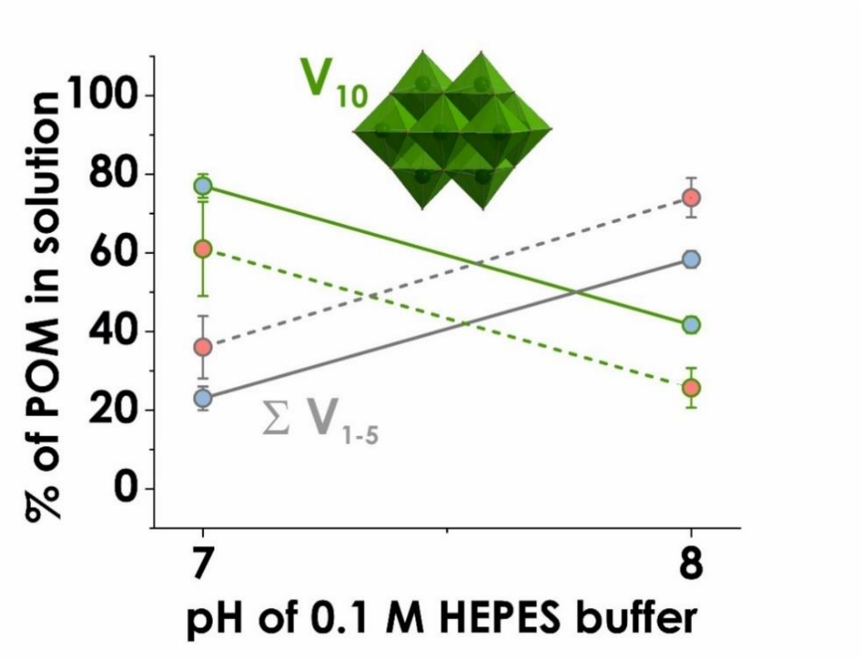

Fig. S146. Speciation of V_{10} in HEPES buffer.

POM concentration curves of **$Na_2K_4[V^V_{10}O_{28}]$** (10 mM) in 0.1 M HEPES buffer solutions before (solid line, blue dot in the middle) and after incubation (dash line, red dot in the middle) for 24 h at 37 °C. The exact percentage of all POM species present is given in **Tables S27** and **S28**.

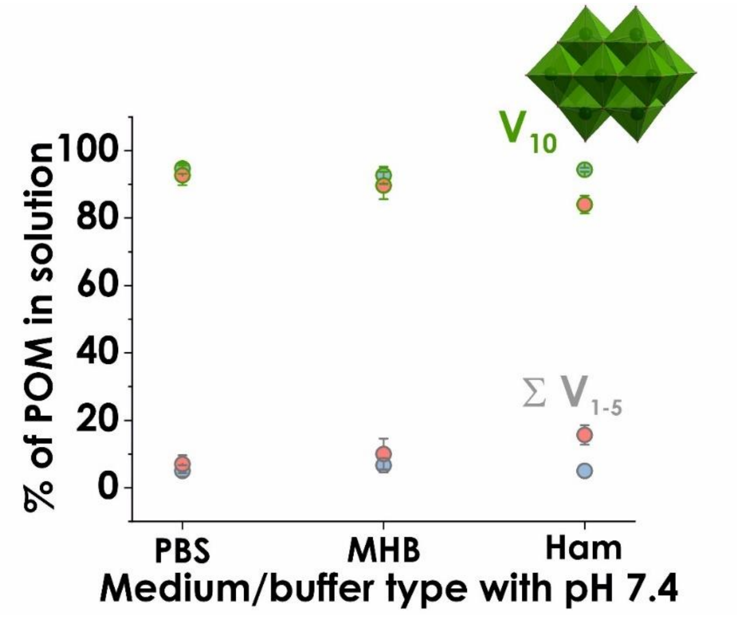

Fig. S147. Speciation of V_{10} in solutions with pH 7.4.

POM concentration curves of **$Na_2K_4[V^V{}_{10}O_{28}]$** (10 mM) in 0.1 M PBS, MHB and nutrient mixture F-12 Ham solutions before (blue dot in the middle) and after incubation (red dot in the middle) for 24 h at 37 °C. The exact percentage of all POM species present is given in **Tables S27** and **S28**.

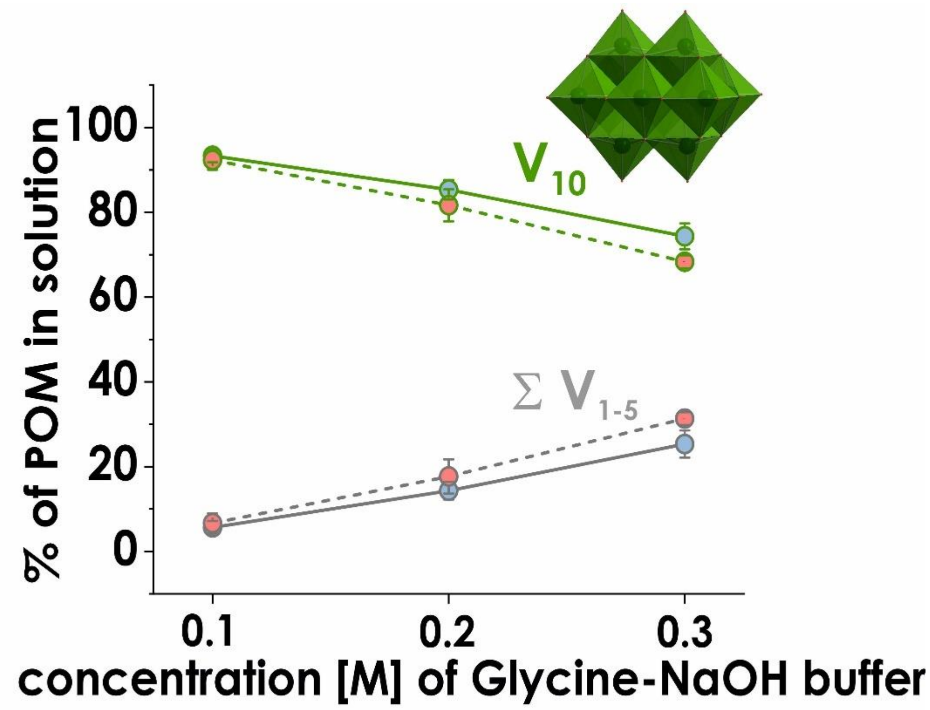

Fig. S148. Speciation of V_{10} in glycine-NaOH buffer.

POM concentration curves in **$Na_2K_4[V^V{}_{10}O_{28}]$** (10 mM) in glycine-NaOH (pH 8.6) with concentrations of 0.1, 0.2 and 0.5 M solutions before (solid line, blue dot in the middle) and after incubation (dash line, red dot in the middle) for 24 h at 37 °C. The exact percentage of all POM species present is given in **Tables S27** and **S28**.

13. The TeW_6 Anderson POT

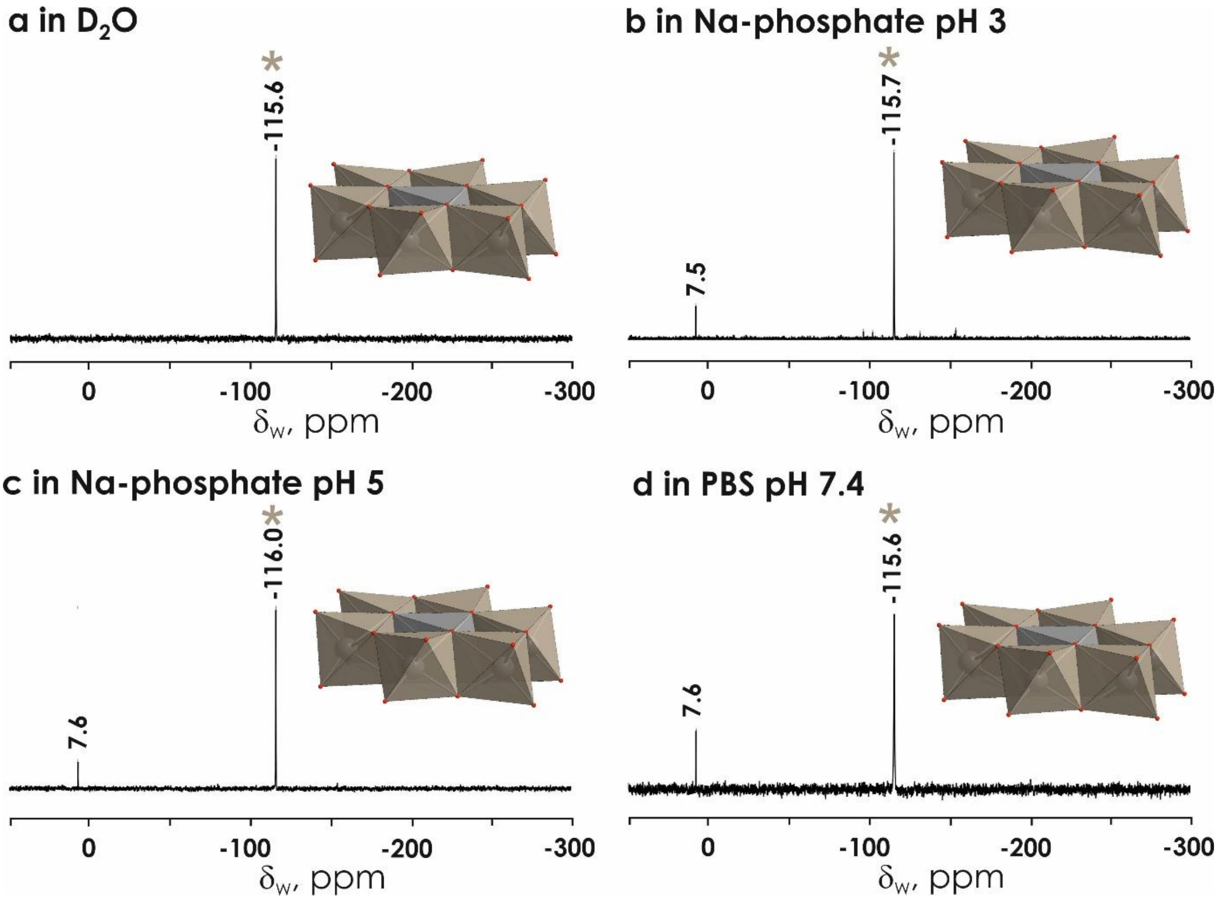

Fig. S149. ^{183}W NMR spectra of TeW_6 solutions.

^{183}W NMR spectra for 20 mM solutions of **$Na_6[Te^{VI}W^{VI}_6O_{24}]$** in (**a**) D_2O (pH after POM dissolving is 5.6); (**b**) in 0.2 M sodium phosphate pH 3 (pH after POM dissolving is 2.9); (**b**) in 0.2 M sodium phosphate pH 5 (pH after POM dissolving is 4.9); (**b**) in PBS pH 7.4 (pH after POM dissolving is 7.4). The chemical shift around –115 ppm corresponds to six equivalent W ions in $[Te^{VI}W^{VI}_6O_{24}]^{6-}$ (**Table S4**). Color code: $\{WO_6\}$, brown-grey; $\{TeO_6\}$, grey; O, red.

14. Ionic strength calculation

$$I = \frac{1}{2}\sum_{i=1}^{n}(c_i \cdot z_i^2) \quad (1),$$

where I – ionic strength, c_i – concentration of ionic species, z_i – charge of ionic species.

The ionic strength for 0.1 M tris-HCl buffer pH 8 and 0.1 M sodium phosphate were calculated using "CurTiPot − pH and Acid−Base Titration Curves: Analysis and Simulation freeware" (Gutz, I. G. R; http://www.iq.usp.br/gutz/Curtipot_.html).

Table S29. Analysis of NMR spectroscopic data of P_2Mo_{18} in tris-HCl with different ionic strength.

Chemical shifts in ^{31}P NMR spectra measured in triplicate of **$(NH_4)_6[\alpha\text{-}P^{V}_2Mo^{VI}_{18}O_{62}]$** (10 mM) tris-HCl pH 8 with and without added $NaNO_3$. The species content was calculated based on the integration of 31P signals considering only signals associated with POTs. Signals were assigned based on the literature data summarized in **Table S4**.

**$Na_6[P_2Mo_{18}O_{62}]$** (10 mM) in Solvent / Buffer / Medium	Chemical shifts δ ^{31}P [ppm]	% of P_2Mo_{18} in **$(NH_4)_6[\alpha\text{-}P^{V}_2Mo^{VI}_{18}O_{62}]$** (10 mM) at RT				% of PMo_{11} in **$(NH_4)_6[\alpha\text{-}P^{V}_2Mo^{VI}_{18}O_{62}]$** (10 mM) at RT				% of PMo_9 in **$(NH_4)_6[\alpha\text{-}P^{V}_2Mo^{VI}_{18}O_{62}]$** (10 mM) at RT				% of P_2Mo_5/PMo_6 in **$(NH_4)_6[\alpha\text{-}P^{V}_2Mo^{VI}_{18}O_{62}]$** (10 mM) at RT				Average % of other POMos present in solution
		Sample			Mean of 1 to 3 ± SD[a]	Sample			Mean of 1 to 3 ± SD[a]	Sample			Mean of 1 to 3 ± SD[a]	Sample			Mean of 1 to 3 ± SD[a]	
		#1	#2	#3		#1	#2	#3		#1	#2	#3		#1	#2	#3		
0.1 M tris-HCl pH 8	**1.2**; –0.3; –1.5; –2.0; –3.2	52	57	50	53 ± 4	24	23	24	24 ± 1	17	15	18	17 ± 2	7	5	8	7 ± 2	0
0.1 M tris-HCl pH 8 with an extra *0.15 M NaNO₃*	**1.2**; –0.3; –1.5; –2.0; –3.2	44	47	45	45 ± 2	29	26	28	28 ± 2	19	21	20	20 ± 1	8	6	7	7 ± 1	0

15. UV-Vis spectroscopic investigation for P_2W_{18} Wells-Dawson POT in MHB medium

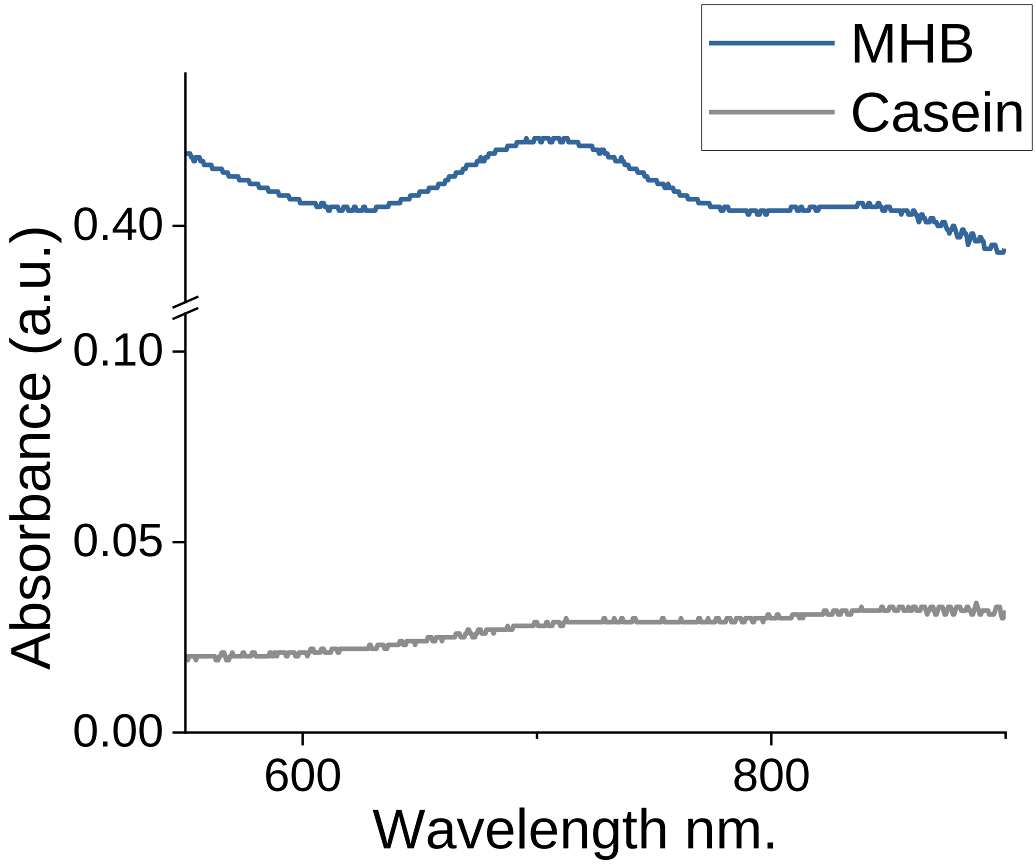

Fig. S150. UV-Vis-spectra of P_2W_{18} in MHB.

UV-Vis-spectra of 10 mM solutions of **$K_6[\alpha\text{-}P^{V}_2W^{VI}_{18}O_{62}]$** in MHB (blue) and in casein-hydrolysate (10 mg/mL) recorded 30 minutes after preparation at room temperature. The MHB spectrum clearly shows a band at ~700 nm, which indicates the reduction of W^{VI} to W^{V} (*9*).

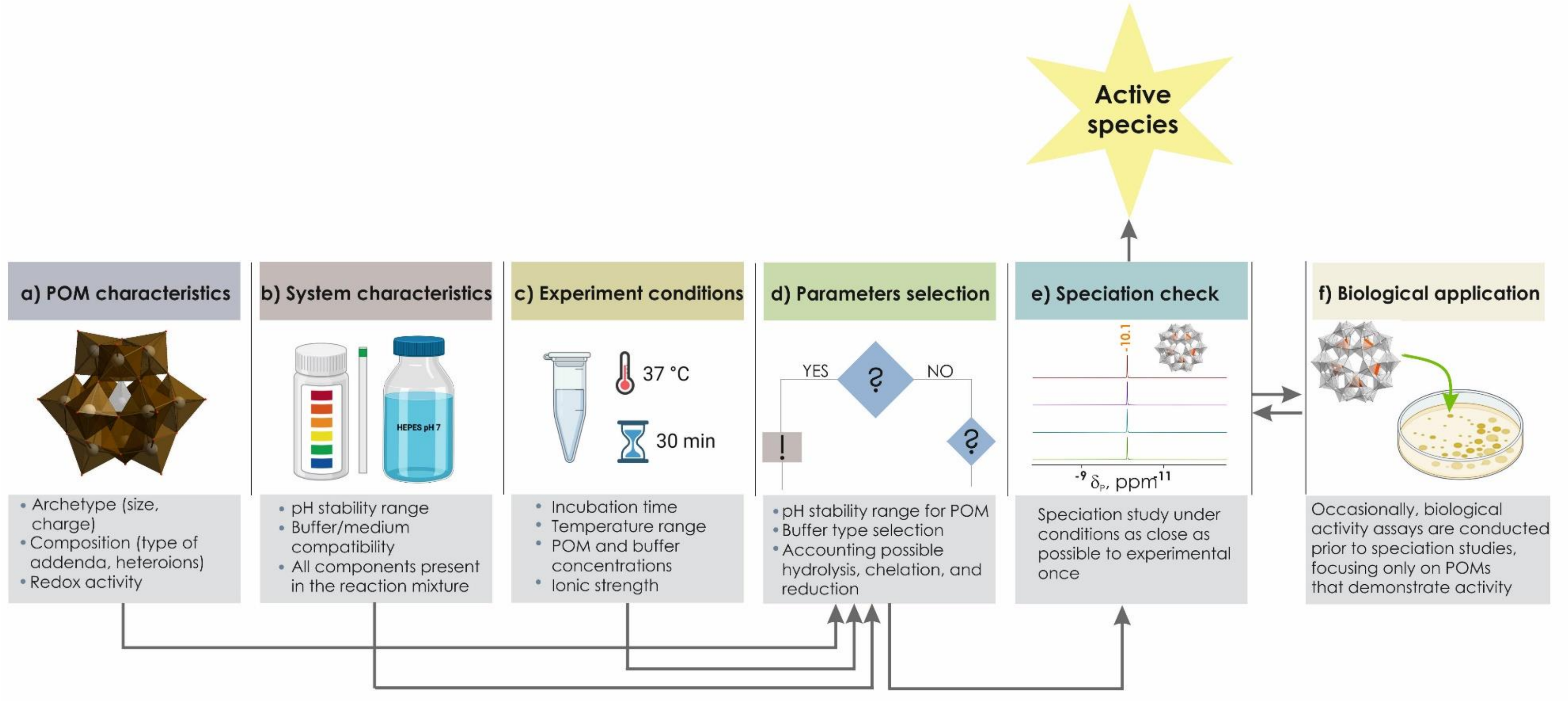

Fig. S151. A roadmap presenting the experiment design for POMs biological applications in aqueous solutions.

The key steps in experiment planning include the POM choice and characterization of the application system. Created with Biorender.com.

REFERENCES AND NOTES

1. M. T. Pope, *Heteropoly and Isopoly Oxometalates* (Springer-Verlag, 1983).

2. N. I. Gumerova, A. Rompel, Polyoxometalates in solution: Speciation under spotlight. *Chem. Soc. Rev.* **49**, 7568–7601 (2020).

3. S.-S. Wang, G.-Y. Yang, Recent advances in polyoxometalate-catalyzed reactions. *Chem. Rev.* **115**, 4893–4962 (2015).

4. I. V. Kozhevnikov, Catalysis by heteropoly acids and multicomponent polyoxometalates in liquid-phase reactions. *Chem. Rev.* **98**, 171–198 (1998).

5. M. Blasco-Ahicart, J. Soriano-López, J. J. Carbó, J. M. Poblet, J. R. Galan-Mascaros, Polyoxometalate electrocatalysts based on earth-abundant metals for efficient water oxidation in acidic media. *Nature Chem.* **10**, 24–30 (2018).

6. A. Bijelic, M. Aureliano, A. Rompel, The antibacterial activity of polyoxometalates: Structures, antibiotic effects and future perspectives. *Chem. Commun.* **54**, 1153–1169 (2018).

7. A. Bijelic, M. Aureliano, A. Rompel, Polyoxometalates as potential next-generation metallodrugs in the combat against cancer. *Angew. Chem. Int. Ed.* **58**, 2980–2999 (2019).

8. M. Aureliano, N. I. Gumerova, G. Sciortino, E. Garribba, A. Rompel, D. C. Crans, Polyoxovanadates with emerging biomedical activities. *Coord. Chem. Rev.* **447** 214143 (2021).

9. N. I. Gumerova, A. Rompel, Synthesis, structures and applications of electron-rich polyoxometalates. *Nature Rev. Chem.* **2**, 0112 (2018).

10. S. J. Folkman, J. T. Kirner, R. G. Finke, Cobalt polyoxometalate $Co_4V_2W_{18}O_{68}^{10-}$: A critical investigation of its synthesis, purity, and observed ^{51}V quadrupolar NMR. *Inorg. Chem.* **55**, 5343–5355 (2018).

11. J. F. Keggin, Structure of the molecule of 12-phosphotungstic acid. *Nature* **131**, 908–909 (1933).

12. C. R. Graham, R. G. Finke, The classic Wells−Dawson polyoxometalate, $K_6[\alpha\text{-}P_2W_{18}O_{62}]\cdot 14H_2O$. Answering an 88 year-old question: What is its preferred, optimum synthesis? *Inorg. Chem.* **47**, 3679–3686 (2008).

13. H. Wu, Contribution to the chemistry of phosphomolybdic acids, phosphotungstic acids, and allied substances. *J. Biol. Chem.* **43**, 189–220 (1920).

14. L. Chen, K. A. San, M. J. Turo, M. Gembicky, S. Fereidouni, M. Kalaj, A. M. Schimpf, Tunable metal oxide frameworks via coordination assembly of Preyssler-type molecular clusters. *J. Am. Chem. Soc.* **141**, 20261–20268 (2019).

15. H. G. T. Ly, G. Absillis, T. N. Parac-Vogt, Amide bond hydrolysis in peptides and cyclic peptides catalyzed by a dimeric Zr(IV)-substituted Keggin type polyoxometalate. *Dalton Trans.* **42**, 10929–10938 (2013).

16. J. C. A. Boeyens, G. J. McDougal, J. V. R. Smit, Crystallographic study of the ammonium/potassium 12-molybdophosphate ion-exchange system. *J. Solid State Chem.* **18**, 191–199 (1976).

17. E. Janusson, N. de Kler, L. Vilà-Nadal, D.-L. Long, L. Cronin, Synthesis of polyoxometalate clusters using carbohydrates as reducing agents leads to isomer-selection. *Chem. Commun.* **55**, 5797–5800 (2019).

18. K. J. Schmidt, G. J. Schrobilgen, J. F. Sawyer, Hexasodium hexatungstotellurate(VI) 22-hydrate. *Acta Cryst.*. **C42**, 1115–1118 (1986).

19. S. Zhao, Y. Jia, Y.-F. Song, Acetalization of aldehydes and ketones over $H_4[SiW_{12}O_{40}]$ and $H_4[SiW_{12}O_{40}]/SiO_2$. *Cat. Sci. Technol.* **4**, 2618–2625 (2014).

20. U. Lee, H.-C. Joo, Potassium–sodium double salt of decavanadate, $K_4Na_2[V_{10}O_{28}]\cdot 10H_2O$. *Acta Cryst.* **59**, i122–i124 (2003).

21. J. Geboers, S. Van de Vyver, K. Carpentier, K. de Blochouse, P. Jacobs, B. Sels, Efficient catalytic conversion of concentrated cellulose feeds to hexitols with heteropoly acids and Ru on carbon. *Chem. Commun.* **46**, 3577–3579 (2010).

22. F. de Azambuja, J. Moons, T. N. Parac-Vogt, The dawn of metal-oxo clusters as artificial proteases: From discovery to the present and beyond. *Acc. Chem. Res.* **54**, 1673–1684 (2021).

23. J. T. Rhule, C. L. Hill, D. A. Judd, R. F. Schinazi, Polyoxometalates in medicine. *Chem. Rev.* **98**, 327–358 (1998).

24. M. J. Pereira, E. Carvalho, J. W. Eriksson, D. C. Crans, M. Aureliano, Effects of decavanadate and insulin enhancing vanadium compounds on glucose uptake in isolated rat adipocytes. *J. Inorg. Biochem.* **103**, 1687–1692 (2009).

25. T. Yamase, Anti-tumor, -viral, and -bacterial activities of polyoxometalates for realizing an inorganic drug. *J. Mater. Chem.* **15**, 4773–4782 (2005).

26. A. Bijelic, A. Rompel, Ten good reasons for the use of the tellurium-centered Anderson-Evans polyoxotungstate in protein crystallography. *Acc. Chem. Res.* **50**, 1441–1448 (2017).

27. Y. Leng, J. Wang, D. Zhu, X. Ren, H. Ge, L. Shen, Heteropolyanion-based ionic liquids: Reaction-induced self-separation catalysts for esterification. *Angew. Chem. Int. Ed.* **48**, 168–171 (2008).

28. A. Tézé, G. Hervé, R. G. Finke, D. K. Lyon, α-, β-, and γ-Dodecatungstosilicic acids: Isomers and related lacunary compounds, in *Inorganic Syntheses*, A. P. Ginsberg, Ed. (Wiley, 1990), vol. 27.

29. D. L. Collins-Wildman, M. Kim, K. P. Sullivan, A. M. Plonka, A. I. Frenkel, D. G. Musaev, C. L. Hill, Buffer-induced acceleration and inhibition in polyoxometalate-catalyzed organophosphorus ester hydrolysis. *ACS Catal.* **8**, 7068–7076 (2018).

30. J. R. Casey, S. Grinstein, J. Orlowski, Sensors and regulators of intracellular pH. *Nat. Rev. Mol. Cell Biol.* **11**, 50–61 (2010).

31. N. E. Good, G. D. Winget, W. Winter, T. N. Connolly, S. Izawa, R. M. Singh, Hydrogen ion buffers for biological research. *Biochemistry* **5**, 467–477 (1966).

32. J. H. Mueller, J. Hinton, A protein-free medium for primary isolation of the Gonococcus and Meningococcus. *Exp. Biol. Med.* **48**, 330–333 (1941).

33. “Methods for dilution antimicrobial susceptibility tests for bacteria that grow aerobically” (Approved Standard M7-A5, National Committee for Clinical Laboratory Standards, 2000).

34. R. J. Beyon, J. S. Easterby, *Buffer Solutions: The Basics* (Oxford Univ. Press, 1996).

35. N. I. Gumerova, A. Rompel, Interweaving disciplines to advance chemistry: Applying polyoxometalates in biology. *Inorg. Chem.* **60**, 6109–6114 (2021).

36. A. Misra, K. Kozma, C. Streb, M. Nyman, Beyond charge balance: Counter-cations in polyoxometalate chemistry. *Angew. Chem. Int. Ed.* **59**, 596–612 (2020).

37. R. I. Maksimovskaya, G. M. Maksimov, ^{31}P NMR studies of hydrolytic conversions of 12-tungstophosphoric heteropolyacid. *Coord. Chem. Rev.* **385**, 81–99 (2019).

38. E. Stellwagen, J. D. Prantner, N. C. Stellwagen, Do zwitterions contribute to the ionic strength of a solution? *Anal. Biochem.* **373**, 407–409 (2008).

39. H. N. Po, N. M. Senozan, The Henderson-Hasselbalch equation: Its history and limitations. *J. Chem. Educ.* **78**, 1499–1503 (2001).

40. N. Samart, Z. Arhouma, S. Kumar, H. A. Murakami, D. C. Crick, D. C. Crans, Decavanadate inhibits mycobacterial growth more potently than other oxovanadates. *Front. Chem.* **6**:519 (2018).

41. J. J. Cruywagen, E. A. Rohwer, G. F. S. Wessels, Molybdenum(VI) complex formation—8. Equilibria and thermodynamic quantities for the reactions with citrate. *Polyhedron* **14**, 3481–3493 (1995).

42. J. J. Cruywagen, L. Krüger, E. A. Rohwer, Complexation of tungsten(VI) with citrate. *J. Chem. Soc. Dalton Trans.* 1727–1731 (1991).

43. M. Inoue, T. Suzuki, Y. Fujita, M. Oda, N. Matsumoto, T. Yamase, Enhancement of antibacterial activity of β-lactam antibiotics by $[P_2W_{18}O_{62}]^{6-}$, $[SiMo_{12}O_{40}]^{4-}$, and $[PTi_2W_{10}O_{40}]^{7-}$ against methicillin-resistant and vancomycin-resistant *Staphylococcus aureus*. *J. Inorg. Biochem.* **100**, 1225–1233 (2006).

44. J. N. Barrows, M. T. Pope, Stabilization and magnetic resonance characterization of the one-electron heteropoly blue derivative of the molybdophosphate $[P_2Mo_{18}O_{62}]^{6-}$. Slow intramolecular proton exchange of the two-electron blue in acetonitrile solution. *Inorg. Chim. Acta* **213**, 91–98 (1993).

45. D. W. Mulder, M. M. Phiri, A. Jordaan, B. C. Vorster, Modified HEPES one-pot synthetic strategy for gold nanostars. *R. Soc. Open Sci.* **6**, 190160 (2019).

46. N. I. Gumerova, E. Al-Sayed, L. Krivosudský, H. Čipčić-Paljetak, D. Verbanac, A. Rompel, Antibacterial activity of polyoxometalates against *Moraxella catarrhalis*. *Front. Chem.* **6**:336 (2018).

47. E. Tanuhadi, N. I. Gumerova, A. Prado-Roller, M. Galanski, H. Čipčić-Paljetak, D. Verbanac, A. Rompel, Aluminum-substituted Keggin germanotungstate $[HAl(H_2O)GeW_{11}O_{39}]^{4-}$: Synthesis, characterization and antibacterial activity. *Inorg. Chem.* **60**, 28–31 (2021).

48. N. I. Gumerova, T. Caldera Fraile, A. Roller, G. Giester, M. C. Pascual-Borràs, C. A. Ohlin, A. Rompel, Direct single- and double-side triol-functionalization of the mixed type Anderson polyoxotungstate $[Cr(OH)_3W_6O_{21}]^{6-}$. *Inorg. Chem.* **58**, 106–113 (2019).

49. N. I. Gumerova, A. Roller, G. Giester, J. Krzystek, J. Cano, A. Rompel, Incorporation of Cr^{III} into a Keggin polyoxometalate as a chemical strategy to stabilize a labile $\{Cr^{III}O_4\}$ tetrahedral conformation and promote unattended single-ion magnet properties. *J. Am. Chem. Soc.* **142**, 3336–3339 (2020).

50. S. Ramos, M. Manuel, T. Tiago, R. Duarte, J. Martins, C. Gutiérrez-Merino, J. J. G. Moura, M. Aureliano, Decavanadate interactions with actin: Inhibition of G-actin polymerization and stabilization of decameric vanadate. *J. Inorg. Biochem.* **100**, 1734–1743 (2006).

51. G. Sciortino, M. Aureliano, E. Garribba, Rationalizing the decavanadate(V) and oxidovanadium(IV) binding to G-actin and the competition with decaniobate(V) and ATP. *Inorg. Chem.* **60**, 334–344 (2021).

52. M. Aureliano, N. I. Gumerova, G. Sciortino, E. Garribba, C. C. McLauchlan, A. Rompel, D. C. Crans, Polyoxidovanadates' interactions with proteins: An overview. *Coord. Chem. Rev.* **454**, 214344 (2022).

53. H. Soria-Carrera, E. Atrián-Blasco, R. Martín-Rapún, S. G. Mitchell, Polyoxometalate–peptide hybrid materials: From structure–property relationships to applications. *Chem. Sci.* **14**, 10–28 (2022).

54. D. E. Salazar Marcano, N. D. Savić, S. A. M. Abdelhameed, F. de Azambuja, T. N. Parac-Vogt, Exploring the reactivity of polyoxometalates toward proteins: From interactions to mechanistic insights. *JACS Au.* **3**, 978–990 (2023).

55. E. Petrus, C. Bo, Unlocking phase diagrams for molybdenum and tungsten nanoclusters and prediction of their formation constants. *J. Phys. Chem. A* **125**, 5212–5219 (2021).

56. E. Petrus, M. Segado-Centellas, C. Bo, Computational prediction of speciation diagrams and nucleation mechanisms: Molecular vanadium, niobium, and tantalum oxide nanoclusters in solution. *Inorg. Chem.* **61**, 13708–13718 (2021).

57. H. S. Harned, *The Physical Chemistry of Electrolytic Solutions* (Reinhold Publishing Corporation, 1958).

58. R. Thouvenot, M. Fournier, R. Franck, C. Rocchiccioli-Deltcheff, Vibrational investigations of polyoxometalates. 3. Isomerism in molybdenum(VI) and tungsten(VI) compounds related to the Keggin structure. *Inorg. Chem.* **23**, 598–605 (1984).

59. K. Nomiya, Y. Saku, S. Yamada, W. Takahashi, H. Sekiya, A. Shinohara, M. Ishimaru, Y. Sakai, Synthesis and structure of dinuclear hafnium(IV) and zirconium(IV) complexes sandwiched between 2 mono-lacunary α-Keggin polyoxometalates. *Dalton Trans.* 5504–5511 (2009).

60. J. Liu, J. Peng, E. Wang, L. Bi, S. Guo, A novel amino acid salt of 18-molybdodiphosphate: Synthesis and structural characterization of $(Lys)_2H_6[P_2Mo_{18}O_{62}]\cdot 16H_2O$. *J. Mol. Struct.* **525**, 71–77 (2000).

61. L. R. Guilherme, A. C. Massabni, A. C. Dametto, R. de Souza Corrêa, A. S. de Araujo, Synthesis, infrared spectroscopy and crystal structure determination of a new decavanadate. *J. Chem. Cryst.* **40**, 897–901 (2010).

62. P. A. Lorenzo-Luis, P. Gili, A. Sánchez, E. Rodriguez-Castellón, J. Jiménez-Jiménez, C. Ruiz-Pérez, X. Solans, Tungstotellurates of the imidazolium and 4-methylimidazolium cations. *Trans. Metal Chem.* **24**, 686–692 (1999).

63. B. Gao, S.-X. Liu, C.-D. Zhang, L.-H. Xie, C.-Y. Sun, M. Yu, Hydrothermal assembly of pyrite-related framework: $(NH_4)_2\{[Ni(H_2O)_3]_2[TeW_6O_{24}]\}\cdot H_2O$. *J. Coord. Chem.* **60**, 911–918 (2007).

64. K. Nomiya, Y. Murara, Y. Iwasaki, H. Arai, T. Yoshida, N. C. Kasuga, T. Matsubara, Highly active, homogeneous catalysis by polyoxometalate-assisted N-heterocyclic carbene gold(I) complexes for hydration of diphenylacetylene. *Mol. Catal.* **469**, 144–154 (2009).

65. J. Zhang, X. Liu, M. Sun, X. Ma, Y. Han, Direct conversion of cellulose to glycolic acid with a phosphomolybdic acid catalyst in a water medium. *ACS Catal.* **2**, 1698–1702 (2012).

66. M. Nakamura, S. Islam, M. A. Rahman, R. N. Nahar, M. Fukuda, Y. Sekine, J. N. Beltramini, Y. Kim, S. Hayami, Microwave aided conversion of cellulose to glucose using polyoxometalate as catalyst. *RSC Adv.* **11**, 34558–34563 (2021).

67. M. S. Islam, M. Nakamura, N. N. Rabin, M. A. Rahman, M. Fukuda, Y. Sekine, J. N. Beltramini, Y. Kim, S. Hayami, Microwave-assisted catalytic conversion of chitin to 5-hydroxymethylfurfural using polyoxometalate as catalyst. *RSC Adv.* **12**, 406–412 (2021).

68. M. Yu, H. Zhu, S. Tian, R. Wang, I. V. Kozhevnikov, Experimental investigation of simultaneous removal of SO_2 and NO_x using a heteropoly compound. *Aerosol Air Qual. Res.* **21**, 210238 (2021).

69. J. Li, S. Zhao, Z. Li, D. Liu, Y. Chi, C. Hu, Efficient conversion of biomass-derived levulinic acid to γ-valerolactone over polyoxometalate@Zr-based metal–organic frameworks: The synergistic effect of Brønsted and Lewis acidic sites. *Inorg. Chem.* **60**, 7785–7793 (2021).

70. M. Singh, A. Yadav, C. P. Pradeep, Keggin cluster modulated photocatalytic activity of aryl sulfonium polyoxometalate hybrids toward dichromate reduction. *Langmuir* **38**, 16034–16045 (2022).

71. Y. Zhou, Q. Ji, C. Hu, H. Liu, J. Qu, A hybrid fuel cell for water purification and simultaneously electricity generation. *Front. Environ. Sci. Eng.* **17**, 11 (2023).

72. J. Wang, D. Han, X. Wang, B. Qi, M. Zhao, Polyoxometalates as peroxidase mimetics and their applications in H_2O_2 and glucose detection. *Biosens. Bioelectron.* **36**, 18–21 (2012).

73. J. Nayak, R. Chilivery, A. K. Kumar, G. Begum, R. K. Rana, A bioinspired assembly to simultaneously heterogenize polyoxometalates as nanozymes and encapsulate enzymes in a microstructure endowing efficient peroxidase-mimicking activity. *ACS Sustainable Chem. Eng.* **9**, 15819–15829 (2021).

74. L. S. van Rompuy, T. N. Parac-Vogt, Polyoxometalates as sialidase mimics: Selective and non-destructive removal of sialic acid from a glycoprotein promoted by phosphotungstic acid. *Chem. Commun.* **53**, 10600–10603 (2017).

75. J. Wang, Z. Tao, T. Tian, J. Qiu, H. Qian, Z. Zha, Z. Miao, Y. Ma, H. Wang, Polyoxometalate nanoclusters: A potential preventative and therapeutic drug for inflammatory bowel disease. *Chem. Eng. J.* **416**, 129137 (2021).

76. H. Zhang, M. Li, Z. Liu, X. Zhang, C. Du, Two Keggin-type polyoxometalates used as adsorbents with high efficiency and selectivity toward antibiotics and heavy metals. *J. Mol. Struc.* **1267**, 133604 (2022).

77. M. S. Petronek, B. G. Allen, G. Luthe, J. M. Stolwijk, Polyoxometalate nanoparticles as a potential glioblastoma therapeutic *via* lipid-mediated cell death. *Int. J. Mol. Sci.* **23**, 8263 (2022).

78. H. Guo, Y. Wang, L. Tian, W. Wei, T. Zhu, Y. Liu, Insight into the enhancing short-chain fatty acids (SCFAs) production from waste activated sludge via polyoxometalates pretreatment: Mechanisms and implications. *Sci. Total Environ.* **800**, 149392 (2021).

79. H. Guo, Y. Wang, L. Tian, W. Wei, T. Zhu, Y. Liu, Unveiling the mechanisms of a novel polyoxometalates (POMs)-based pretreatment technology for enhancing methane production from waste activated sludge. *Bioresource Technol.* **342**, 125934 (2021).

80. G. Chi, Y. Qi, J. Li, L. Wang, J. Hu, Polyoxomolybdates as α-glucosidase inhibitors: Kinetic and molecular modeling studies. *J. Inorg. Biochem.* **193**, 173–179 (2019).

81. M. Bazargan, M. Mirzaei, A. Amiri, J. T. Mague, Opioid drug detection in hair samples using polyoxometalate-based frameworks. *Inorg. Chem.* **62**, 56–65 (2023).

82. L. Faleiro, A. Marques, J. Martins, L. Jordão, I. Nogueira, N. I. Gumerova, A. Rompel, M. Aureliano, The Preyssler-type polyoxotungstate exhibits anti-quorum sensing, antibiofilm, and antiviral activities. *Biology* **11**, 994 (2022).

83. C. Pimpão, I. V. da Silva, A. F. Mósca, J. O. Pinho, M. M. Gaspar, N. I. Gumerova, A. Rompel, M. Aureliano, G. Soveral, The Aquaporin-3-inhibiting potential of polyoxotungstates. *Int. J. Mol. Sci.* **21**, 2467 (2020).

84. J.-J. Chen, L. Vilà-Nadal, A. Solé-Daura, G. Chisholm, T. Minato, C. Busche, T. Zhao, B. Kandasamy, A. Y. Ganin, R. M. Smith, I. Colliard, J. J. Carbó, J. M. Poblet, M. Nyman, L. Cronin, Effective storage of electrons in water by the formation of highly reduced polyoxometalate clusters. *J. Am. Chem. Soc.* **144**, 8951–8960 (2022).

85. J. Cao, X. Wang, Y. Zhang, X. Xie, Effect of the Wells–Dawson phosphomolybdic heteropolyacid on the conversion of glucose into glycolic acid. *React. Chem. Eng.* **7**, 691–698 (2022).

86. F. Zhou, D. Wang, J. Zhang, J. Li, D. Lai, S. Lin, J. Hu, Preparation and characterization of biodegradable κ-carrageenan based anti-bacterial film functionalized with Wells-Dawson polyoxometalate. *Foods* **11**, 586 (2022).

87. J. J. Hu, L. Wang, B.-N. Chen, G.-X. Chi, M. J. Zhao, Y. Li, Transition metal substituted polyoxometalates as α-glucosidase inhibitors. *Eur. J. Inorg. Chem.* **2019**, 3270–3276 (2019).

88. K. Chen, Q. Yu, Y. Liu, P. Yin, Bacterial hyperpolarization modulated by polyoxometalates for solutions of antibiotic resistance. *J. Inorg. Biochem.* **220**, 111463 (2021).

89. M. Aureliano, G. Fraqueza, M. Berrocal, J. J. Cordoba-Granados, N. I. Gumerova, A. Rompel, C. Gutierrez-Merino, A. M. Mata, Inhibition of SERCA and PMCA Ca^{2+}-ATPase activities by polyoxotungstates. *J. Inorg. Biochem.* **236**, 111952 (2022).

90. Y. W. Zhou, L. Zheng, F. Han, G. J. Zhang, Y. Mac, J. N. Yao, B. Keita, P. de Oliveira, L. Nadjo, Inhibition of amyloid-β protein fibrillization upon interaction with polyoxometalates nanoclusters. *Colloids Surf. A Physicochem. Eng. Aspects* **375**, 97–101 (2011).

91. A. Haider, K. Zarschler, S. A. Joshi, R. M. Smith, Z. Lin, A. S. Mougharbel, U. Herzog, C. E. Müller, H. Stephan, U. Kortz, Preyssler-Pope-Jeannin polyanions $[NaP_5W_{30}O_{110}]^{14-}$ and $[AgP_5W_{30}O_{110}]^{14-}$: Microwave-assisted synthesis, structure, and biological activity. *Z. Anorg. Allg. Chem.* **644**, 752–758 (2018).

92. S. F. Razavi, F. F. Bamoharram, T. Hashemi, K. Shahrokhabadi, A. Davoodnia, Nanolipid-loaded Preyssler polyoxometalate: Synthesis, characterization and *in vitro* inhibitory effects on HepG2 tumor cells. *Toxicol. In Vitro* **68**, 104917 (2020).

93. Z. Xu, K. Chen, M. Li, C. Hu, P. Yin, Sustained release of Ag^+ confined inside polyoxometalates for long-lasting bacterial resistance. *Chem. Commun.* **56**, 5287–5290 (2020).

94. S. Liu, J. Tian, L. Wang, Y. Zhang, Y. Luo, H. Li, A. M. Asiri, A. O. Al-Youbi, X. Sun, Fast and sensitive colorimetric detection of H_2O_2 and glucose: A strategy based on polyoxometalate clusters. *ChemPlusChem* **77**, 541–544 (2012).

95. H. G. T. Ly, G. Absillis, R. Janssens, P. Proost, T. N. Parac-Vogt, Highly amino acid selective hydrolysis of myoglobin at aspartate residues as promoted by zirconium(IV)-substituted polyoxometalates. *Angew. Chem. Int. Ed.* **54**, 7391–7394 (2015).

96. T. K. N. Luong, P. Shestakova, T. T. Mihaylov, G. Absillis, K. Pierloot, T. N. Parac-Vogt, Multinuclear diffusion NMR spectroscopy and DFT modeling: A powerful combination for unraveling the mechanism of phosphoester bond hydrolysis catalyzed by metal-substituted polyoxometalates. *Chem. A Eur. J.* **21**, 4428–4439 (2015).

97. H. Giang, T. Ly, T. N. Parac-Vogt, Spectroscopic study of the interaction between horse heart myoglobin and zirconium(IV)-substituted polyoxometalates as artificial proteases. *ChemPhysChem* **18**, 2451–2458 (2017).

98. T. K. N. Luong, I. Govaerts, J. Robben, P. Shestakova, T. N. Parac-Vogt, Polyoxometalates as artificial nucleases: Hydrolytic cleavage of DNA promoted by a highly negatively charged Zr^{IV}-substituted Keggin polyanion. *Chem. Commun.* **53**, 617–620 (2017).

99. H. G. T. Ly, T. T. Mihaylov, P. Proost, K. Pierloot, J. N. Harvey, T. N. Parac-Vogt, Chemical mimics of aspartate-directed proteases: Predictive and strictly specific hydrolysis of a globular protein at Asp−X sequence promoted by polyoxometalate complexes rationalized by a combined experimental and theoretical approach. *Chem. A Eur. J.* **25**, 14370–14381 (2019).

100. M. Gil-Moles, S. Türck, U. Basu, A. Pettenuzzo, S. Bhattacharya, A. Rajan, X. Ma, R. Büssing, J. Wölker, H. Burmeister, H. Hoffmeister, P. Schneeberg, A. Prause, P. Lippmann, J. Kusi-Nimarko, S. Hassell-Hart, A. McGown, D. Guest, Y. Lin, A. Notaro, R. Vinck, J. Karges, K. Cariou, K. Peng, X. Qin, X. Wang, J. Skiba, Ł. Szczupak, K. Kowalski, U. Schatzschneider, C. Hemmert, H. Gornitzka, E. R. Milaeva, A. A. Nazarov, G. Gasser, J. Spencer, L. Ronconi, U. Kortz, J. Cinatl, D. Bojkova, I. Ott, Metallodrug profiling against SARS-CoV-2 target proteins identifies highly potent inhibitors of the S/ACE2 interaction and the papain-like protease PL^{pro}. *Chem. A Eur. J.* **27**, 17928–17940 (2021).

101. S. G. Mauracher, C. Molitor, R. Al-Oweini, U. Kortz, A. Rompel, Crystallization and preliminary x-ray crystallographic analysis of latent isoform PPO4 mushroom (*Agaricus bisporus*) tyrosinase. *Acta Cryst.* **70**, 263–266 (2014).

102. S. G. Mauracher, C. Molitor, R. Al-Oweini, U. Kortz, A. Rompel, Latent and active $abPPO_4$ mushroom tyrosinase cocrystallized with hexatungstotellurate(VI) in a single crystal. *Acta Crystallogr. D Biol. Crystallogr.* **70**, 2301–2315 (2014).

103. A. Bijelic, C. Molitor, S. G. Mauracher, R. Al-Oweini, U. Kortz, A. Rompel, Hen egg-white lysozyme crystallisation: Protein stacking and structure stability enhanced by a tellurium(VI)-centred polyoxotungstate. *Chembiochem* **16**, 233–241 (2015).

104. C. Molitor, S. G. Mauracher, A. Crystallization and preliminary crystallographic analysis of latent, active and recombinantly expressed aurone synthase, a polyphenol oxidase, from *Coreopsis grandiflora*. *Acta Cryst.* **71** 746–751 (2015).

105. C. Molitor, A. Bijelic, A. Rompel, *In situ* formation of the first proteinogenically functionalized $[TeW_6O_{24}O_2(Glu)]^{7-}$ structure reveals unprecedented chemical and geometrical features of the Anderson-type cluster. *Chem. Commun.* **52**, 12286–12289, (2016).

106. A. Mac Sweeney, A. Chambovey, M. Wicki, M. Müller, N. Artico, R. Lange, A. Bijelic, J. Breibeck, A. Rompel, The crystallization additive hexatungstotellurate promotes the crystallization of the HSP70 nucleotide binding domain into two different crystal forms. *PLOS ONE* **13**, e0199639 (2018).

107. J. D. Somasundaram, A. Ebrahimi, S. P. Nandan, A. Cherevan, D. Eder, M. Šupolíková, E. Nováková, R. Gyepes, L. Krivosudský, Functionalization of decavanadate anion by coordination to cobalt(II): Binding to proteins, cytotoxicity, and water oxidation catalysis. *J. Inorg. Biochem.* **239**, 112067 (2023).

108. T. Aissa, R. Ksiksi, I. Elbini-Dhouib, R. Doghri, N. Srairi-Abid, M. F. Zid, Synthesis of a new vanadium complex (V), hexa [4-methylimidazolium] decavanadate trihydrate $(C_4H_7N_2)_6V_{10}O_{28}\cdot 3H_2O$: Physico-chemical and biological characterizations. *J. Mol. Struc.* **1236**, 130331 (2021).

109. A. L. De Sousa-Coelho, M. Aureliano, G. Fraqueza, G. Serrão, J. Gonçalves, I. Sánchez-Lombardo, W. B. Link, I. Ferreira, Decavanadate and metformin-decavanadate effects in human melanoma cells. *J. Inorg. Biochem.* **235**, 111915 (2022).

110. D. Favre, J. F. Harmon, A. Zhang, M. S. Miller, I. A. Kaltashov, Decavanadate interactions with the elements of the SARS-CoV-2 spike protein highlight the potential role of electrostatics in disrupting the infectivity cycle. *J. Inorg. Biochem.* **234**, 111899 (2022).

111. R. Ksiksi, A. Essis, S. Kouka, F. Boujelbane, M. Daoudi, N. Srairi-Abid, M. F. Zid, Synthesis and characterization of a tetra-(benzylammonium) dihydrogen decavanadate dihydrate compound inhibiting MDA-MB-231 human breast cancer cells proliferation and migration. *J. Mol. Struct.* **1250**, 131929 (2022).

112. R. Massart, R. Contant, J. M. Fruchart, J. P. Ciabrini, M. Fournier, Phosphorus-31 NMR studies on molybdic and tungstic heteropolyanions. Correlation between structure and chemical shift. *Inorg. Chem.* **16**, 2916–2921 (1977).

113. M. Kozik, C. F. Hammer, L. C. W. Baker, NMR of phosphorus-31 heteroatoms in paramagnetic 1-electron heteropoly blues. Rates of intra- and intercomplex electron transfers. Factors affecting line widths. *J. Am. Chem. Soc.* **108**, 7627–7630 (1986).

114. M. A. Fedotov, R. I. Maksimovskaya, NMR structural aspects of the chemistry of V, Mo, W polyoxometalates. *J. Struct. Chem.* **47**, 952–978 (2006).

115. S. R. Bajpe, E. Breynaert, K. Robeyns, K. Houthoofd, G. Absillis, D. Mustafa, T. N. Parac-Vogt, A. Maes, J. A. Martens, C. E. A. Kirschhock, Chromate-mediated one-step quantitative transformation of PW_{12} into P_2W_{20} polyoxometalates. *EurJIC* **24**, 3852–3858 (2012).

116. R. Contant, M. Abbessi, R. Thouvenot, G. Hervé, Dawson type heteropolyanions. 3. Syntheses and ^{31}P, ^{51}V, and ^{183}W NMR structural investigation of octadeca(molybdo−tungsto−vanado)diphosphates related to the $[H_2P_2W_{12}O_{48}]^{12-}$ anion. *Inorg. Chem.* **43**, 3597–3604 (2004).

117. T. K. Nga Luong, G. Absillis, P. Shestakova, T. N. Parac-Vogt, Hydrolysis of the RNA model substrate catalyzed by a binuclear Zr^{IV}-substituted Keggin polyoxometalate. *Dalton Trans.* **44**, 15690–15696 (2015).

118. I. M. Krukovsky, A. M. Sheloumov, O. V. Golubev, A. N. Loginova, V. V. Fadeev, Optimization of phosphomolybdic acid synthesis using ^{31}P NMR data. *J Appl Spectrosc.* **87**, 267–274 (2020).

119. L. Pettersson, I. Andersson, L. O. Oehman, Multicomponent polyanions. 39. Speciation in the aqueous hydrogen ion-molybdate(MoO_4^{2-})-hydrogenphosphate(HPO_4^{2-}) system as deduced from a combined Emf-phosphorus-31 NMR study. *Inorg. Chem.* **25**, 4726–4733 (1986).

120. J. A. R. van Veen, O. Sudmeijer, C. A. Emeis, H. de Wit, On the identification of molybdophosphate complexes in aqueous solution. *J. Chem. Soc. Dalton Trans.* 1825–1831 (1986).

121. L. P. Kazansky, M. A. Fedotov, Phosphorus-31 and oxygen-17 n.m.r. evidence of trapped electrons in reduced 18-molybdodiphosphate(V), $P_2Mo_{18}O_{62}^{8-}$. *J. Chem. Soc. Chem. Commun.* 644–646 (1980).

122. D. C. Crans, B. Baruah, N. E. Levinger, Oxovanadates: A novel probe for studying lipid-water interfaces. *Biomed. Pharmacother.* **60**, 174–181 (2006).

123. E. Heath, O. W. Howarth, Vanadium-51 and oxygen-17 nuclear magnetic resonance study of vanadate(V) equilibria and kinetics. *J. Chem. Soc. Dalton Trans.* 1105–1110 (1981).

124. R. Acerete, C. F. Hammer, L. C. W. Baker, Tungsten-183 NMR of heteropoly and isopolytungstates. Explanations of chemical shifts and band assignments and theoretical considerations. *J. Am. Chem. Soc.* **104**, 5384–5390 (1982).

125. Y. G. Chen, J. Gong, L.-Y. Qu, Tungsten-183 nuclear magnetic resonance spectroscopy in the study of polyoxometalates. *Coord. Chem. Rev.* **248**, 245–260 (2004).
